# Reactivity of *δ*‐Functionalized *Para*‐Quinone Methides in Nucleophilic Addition Reactions

**DOI:** 10.1002/chem.202501224

**Published:** 2025-06-25

**Authors:** Christoph Gross, Andreas Eitzinger, Peter Mayer, Armin R. Ofial

**Affiliations:** ^1^ Department Chemie Ludwig‐Maximilians‐Universität München Butenandtstr. 5–13, 81377 München Germany; ^2^ Institute of Organic Chemistry Johannes Kepler University Linz Altenberger Straße 69, 4040 Linz Austria

**Keywords:** buried volumes, electrophilicity, kinetics, linear free energy relationships, quinone methides

## Abstract

The electrophilic reactivities of *para*‐quinone methides (*p*QMs) with functional groups (FG) at the exocyclic polarized carbon–carbon double bond were determined by photometrically monitoring the kinetics of their reactions with carbanions in dimethyl sulfoxide (DMSO) at 20 °C. The experimental second‐order rate constants *k*_2_ were evaluated by the Mayr‐Patz equation, that is, the linear free energy relationship lg *k*_2_ = *s*_N_(*N *+ *E*), which was leveraged to determine the electrophilicity descriptors *E* of the *p*QMs. These electrophilicity parameters *E* were subsequently used to successfully predict the scope of the *p*QM reactions with C‐, H‐, N‐, O‐, and S‐centered nucleophiles. Moreover, the electrophilicity parameters *E* correlate linearly with a linear combination of quantum‐chemically calculated methyl anion affinities (MAAs) and buried volumes (%*V*_bur_). While MAA values mainly reflect the thermodynamic driving force of the carbon–carbon bond formation, %*V*_bur_ values take account of the variable steric effects of substituents at the electrophilic *δ*‐position of the *p*QMs. Knowledge of MAA and %*V*_bur_ thus enables chemists to tailor novel *p*QMs with predictable reactivity properties.

## Graphical abstract

The reactivities of *δ*‐functionalized *para*‐quinone methides (*p*QMs) were characterized by kinetic studies of their reactions with carbanions in DMSO. Evaluating the second‐order rate constants (*k*_2_) by the Mayr‐Patz equation gave the electrophilicities *E* of *p*QMs. Reactivities of unseen *δ*‐functionalized *p*QMs can be predicted by using the linear relationship of *E* with a combination of quantum chemically calculated methyl anion affinities (MAA) and buried volumes (%*V*_bur_).

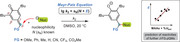

## 1 Introduction

**Scheme 1:**
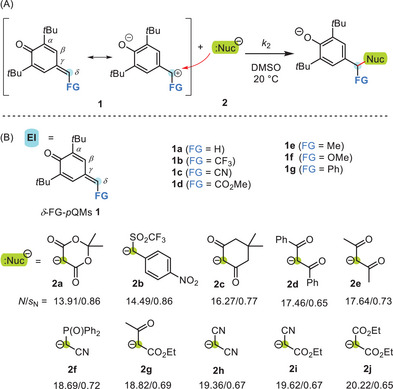
a) Nucleophiles attack *δ*‐functionalized *para*‐quinone methides (*δ*‐FG‐*p*QMs) at the polarized exocyclic π‐bond. b) Structures of *δ*‐FG‐*p*QM electrophiles and reference nucleophiles used in this work (counterion: K^+^ or K^+^/18‐crown‐6). Nucleophilicity parameters *N* (and *s*_N_) refer to reactivities in DMSO; refs. [20, 26].

*Para*‐quinone methides (*p*QMs) are a subclass of cyclic Michael acceptors, in which an exocyclic methylene group is in conjugation with an *α*,*β*‐unsaturated ketone.^[^ ^1^ ^]^ This combination of functionalities results in a strong polarization of the exocyclic π‐bond and a high reactivity toward nucleophiles at the *δ*‐position (Scheme 1a).^[^ ^2^ ^]^ Besides the significant role of *p*QMs in biological processes^[^ ^3^, ^4^, ^5^ ^]^ they are frequently utilized in synthetic transformations where *p*QMs undergo 1,6‐conjugate nucleophilic additions^[^ ^6^, ^7^, ^8^, ^9^ ^]^ or a multitude of ring‐forming reactions.^[^ ^10^, ^11^, ^12^, ^13^ ^]^

Owing to their relevance as vinylogous Michael acceptors, several attempts were made to characterize their electrophilicity, in particular by pH‐dependent kinetic measurements in aqueous solution.^[^ ^14^, ^15^, ^16^, ^17^, ^18^ ^]^ Furthermore, *δ*‐aryl‐substituted *p*QMs (**1** with FG = aryl, such as **1** **g** in Scheme 1b) resemble structural analogues of benzhydrylium ions and were, therefore, used by H. Mayr and coworkers as reference electrophiles to extend their comprehensive reactivity scales toward highly reactive nucleophiles.^[^ ^19^, ^20^, ^21^ ^]^

The Mayr reactivity scales are based on the linear free energy relationship in Equation (1), that uses three parameters to calculate the second‐order rate constant *k*_2_ of a given electrophile‐nucleophile reaction at 20 °C.^[^ ^22^, ^23^, ^24^, ^25^ ^]^

(1) $$\mathrm{lg}\;k_{2}=s_{N}\left(N+E\right)$$

Nucleophiles are characterized by two solvent‐dependent parameters, *s*_N_ and *N*. The reactivity of electrophiles is described by a single electrophilicity parameter *E*. So far, Equation (1) has been utilized to characterize the reactivity of > 1300 nucleophiles and 350 electrophiles, including aryl‐substituted *ortho*‐ and *para*‐quinone methides.^[^ ^26^ ^]^

Though syntheses and spectroscopic characterization of simple *δ*‐functional group‐substituted *para‐*quinone methides (*δ*‐FG‐*p*QMs) **1** (Scheme 1) have been reported since the 1960s,^[^ ^27^, ^28^, ^29^, ^30^ ^]^ systematic reactivity studies of these versatile electrophiles with synthetically relevant nucleophiles in organic solvents are still scarce.^[^ ^18^, ^27^, ^31^, ^32^ ^]^

This lack of knowledge is currently retarding a more comprehensive exploitation of the synthetic potential of *δ*‐FG‐*p*QMs and motivated us to quantify their electrophilicity by studying the kinetics of their reactions with carbanions **2** as reference nucleophiles. The experimentally determined rate constants *k*_2_ of these model reactions provide the fundament for embedding *δ*‐FG‐*p*QMs **1** in Mayr's reactivity scales. The location of *δ*‐FG‐*p*QMs **1** in the electrophilicity scale will then provide a powerful tool to enhance the synthetic space of these electrophiles by the straightforward identification of novel nucleophilic reaction partners. Additionally, we will show that DFT calculations can be efficiently used to reliably predict the electrophilicities of further *δ*‐FG‐*p*QM derivatives.

## 2 Results and Discussion

### 2.1 Product Studies

**Scheme 2:**
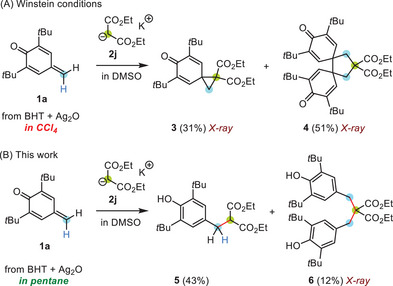
Reactions of **1a** with the nucleophile **2j**: depending on the solvent used to preform *p*QM **1a** either a) cyclic products **3** and **4** from Michael addition/oxidative radical cyclization sequences or b) Michael adducts **5** and **6** were isolated (conditions: 1 hour, r.t.).

**Figure 1:**
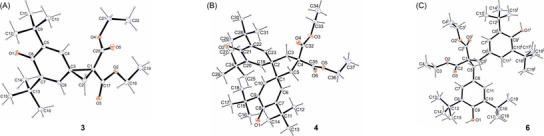
Crystalline products from the reactions of the *p*QM **1a** with potassium diethyl malonate (**2j**): scXRD structures of a) the cyclopropane **3**, b) the cyclopentane **4**, and c) the double Michael adduct **6**. Thermal ellipsoids are shown on the 25% probability level (at 173 K).^[^ ^34^ ^].^

First, we studied the products of the reactions between *p*QMs **1** and the potassium salts of the carbanions **2** that we selected as potential reference nucleophiles for the kinetic measurements. To do so, we generated the parent *δ*‐FG‐*p*QM **1a** (FG = H) by oxidation of 3,5‐di‐*tert*‐butyl‐4‐hydroxytoluene (BHT) with silver(I) oxide in tetrachloromethane as originally reported by Winstein.^[^ ^27^, ^30^ ^]^ The NMR spectroscopic analysis showed that BHT was quantitatively converted into the *p*QM **1a** within 20 minutes under Winstein's conditions.^[^ ^33^ ^]^ After filtration from solids, the thus obtained CCl_4_ solution of **1a** was mixed with a DMSO solution of potassium diethyl malonate (**2j**). These reaction conditions did not furnish simple Michael adducts, however, but a mixture of the spirocyclopropane **3** and the bis‐spiro cyclopentane **4**. After aqueous workup and separation by chromatography, **3** and **4** were isolated in yields of 31% and 51%, respectively (Scheme 2a). Analysis of **3** and **4** by single crystal X‐ray diffraction (scXRD) confirmed the structural assignments (Figure 1a,b),^[^ ^34^ ^]^ which were derived from the NMR spectra for both reaction products. We rationalized the formation of both **3** and **4** by initial Michael additions followed by ring‐forming reactions involving radical intermediates.^[^ ^35^, ^36^ ^]^ The ring systems in the solid‐state structures of both **3** and **4** are characterized by one C─C bond which is significantly longer than the average of the other C─C bond lengths in the same ring. In the three‐membered ring of **3**, *d*(C1‐C3) = 1.5711(13) Å is longer than *d*(C1‐C2) = 1.4913(13) Å or *d*(C2‐C3) = 1.5217(13) Å.^[^ ^34^ ^]^ Even more obvious, *d*(C1‐C2) = 1.6044(17) Å in the cyclopentane ring of **4** deviates from the lengths of the other C─C bonds in the same ring, which are in a narrow range from 1.5479(19) to 1.5552(19) Å.^[^ ^34^ ^]^

A screening of conditions for the generation of *p*QM **1a** by oxidation of BHT with silver(I) oxide identified pentane as a solvent, which made it possible to carry out the Michael reaction of **1a** and **2j** without competing intramolecular radical cyclizations. As shown in Scheme 2b, the 1:1 Michael adduct **5** as well as the 2:1 Michael adduct **6** were isolated (Figure 1c).^[^ ^36^ ^]^ Thus, polar reactions of the parent **1a** with nucleophiles could be carried out without being disturbed by electron transfer reactions, presumably induced by traces of oxidizing metal ions.

**Scheme 3:**
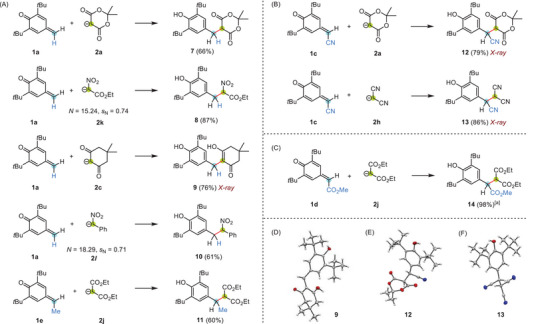
Michael additions of carbanionic nucleophiles **2** (counterion: K^+^) to *δ*‐substituted *p*QMs **1a** and **1e** (a), **1c** (b), and **1d** (c) in DMSO or pentane/DMSO mixtures (1 hour at r.t.). The quinone methides **1** were generated from the corresponding phenols by oxidation with silver(I) oxide in *n*‐pentane.^[^ ^33^ ^]^ Yields refer to isolated products after aqueous workup. Single crystal structures with thermal ellipsoids on the 50% probability level (at 173 K) are shown for **9** (d), **12** (e), and **13** (f).^[^ ^34^ ^]^ [a] Owing to a high degree of disorder in crystalline **14** only low‐quality scXRD data were obtained.^[^ ^33^, ^34^ ^].^

A rapid fading of the colored *p*QMs **1** ^[^ ^33^, ^37^ ^]^ was observed when they were mixed with the carbanions **2** (counterion: K^+^) in DMSO or *n*‐pentane/DMSO solvent mixtures. The reaction mixtures were worked up and purified by chromatography to isolate the Michael adducts in unoptimized yields of 60–98% (Scheme 3). For example, *p*QM **1a** (FG = H) and **1e** (FG = Me) reacted with carbanions **2** to furnish after aqueous workup in good yields the simple 1,6‐addition products **7**–**11**, which were spectroscopically characterized. In CDCl_3_ solution, **9** was characterized as a 1:1 keto‐enol mixture. In crystalline state, however, the scXRD analysis of **9** (Scheme 3d) showed exclusively the enol form of the 1,3‐dicarbonyl groups of the dimedone moiety.

Also, the reactions of *p*QM **1c** (FG = CN) with deprotonated Meldrum's acid **2a** as well as with the malononitrile‐derived carbanion **2h** generated the Michael adducts **12** and **13**, respectively, in decent yields of 79% and 86%. In agreement with the structural data for **7**, NMR spectroscopic data and scXRD analysis of **12** (Scheme 3e) showed the bis‐lactone structure of the Meldrum's acid moiety. Indications for the alternative enol forms were not found. Phenol **13** carries three adjacent nitrile groups in the side chain at the 4‐position (Scheme 3b,f). The analogous reaction of *p*QM **1d** (FG = CO_2_Me) with the diethyl malonate‐derived nucleophile **2j** yielded a 1,6‐addition product with three ester groups at the side chain of the phenol **14** (Scheme 3c).

**Scheme 4:**
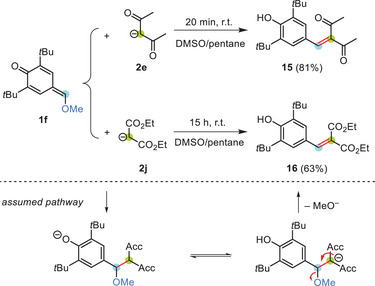
The *δ*‐methoxy‐substituted *p*QM **1f** reacted with the carbanions **2e** and **2j** (counterion: K^+^) in an addition‐elimination sequence to form **15** and **16**, respectively. Yields refer to isolated products after aqueous workup.

Scheme 4 illustrates that reactions of the *δ*‐methoxy‐*p*QM **1f** with carbanions took another course. Additions of the C‐nucleophiles **2e** and **2j** to **1f** were accompanied by subsequent methanol eliminations to give the benzylidene pentan‐2,4‐dione **15** and the benzylidene malonate **16**, respectively. Nevertheless, formation of both **15** and **16** is rationalized by an initial σ‐bond formation through an electrophile‐nucleophile combination. The similar Brønsted basicities of the phenolate oxygen (p*K*_a_ = 16.8 for 2,6‐di‐*tert*‐butylphenol in DMSO) and the acceptor‐stabilized carbanions which emerge from the acetylacetone or malonate part of the adducts (p*K*_a_ = 15.1 for 2‐methylacetylacetone; p*K*_a_ = 18.0 for dimethyl 2‐methylmalonate in DMSO)^[^ ^38^ ^]^ facilitate a subsequent proton shift from the CH acid to the phenolate oxygen to give carbanions, which then eliminate methoxide ions to furnish the isolated benzylidenes **15** and **16**, respectively. An analogous course was described by Tsuri and colleagues for the reaction of **1f** with α‐lithiated *N*‐ethyl‐γ‐sultam, which gave the corresponding benzylidene compound considered as a drug candidate for the treatment of arthritis.^[^ ^39^ ^]^

### 2.2 Kinetics

The product studies showed that formation of all isolated products can be rationalized by electrophile‐nucleophile combinations of **1** and **2**, in which one new σ‐bond is formed in the first step of the reaction. Thus, a crucial prerequisite for the application of the Mayr‐Patz equation (Equation (1)) was fulfilled and we, therefore, set out to characterize the electrophilicity of the *δ*‐FG‐*p*QMs **1** by kinetic methods.

**Figure 2:**
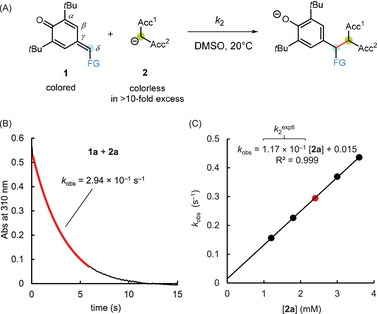
a) Carbon–carbon bond‐forming reaction of an electrophilic *p*QM **1** with a C‐centered nucleophile **2** in DMSO at 20 °C. b) The time‐dependent decay of the absorbance of **1a** ([**1a**]_0 _= 2.50 × 10^−5^ M) at 310 nm in the course of the reaction with **2a** ([**2a**]_0 _= 2.40 × 10^−3^ M) was used to determine the first‐order rate constant *k*_obs_ (s^−1^). c) The slope of the linear correlation of *k*_obs_ with [**2a**] corresponds to the second‐order rate constant *k*_2_^exp^ (M^−1^ s^−1^) for the **1a **+ **2a** addition reaction.

Figure 2 exemplifies the general procedure for determining the kinetics of the **1** + **2** reactions. The kinetics of the reactions of *p*QMs **1** with carbanions **2** in DMSO at 20 °C were monitored with stopped‐flow and conventional UV‐Vis spectroscopy by following the decay of the absorbance of the colored *p*QMs **1**. Initial concentrations of the colorless carbanions **2** were at least 10‐fold higher than the initial concentrations of the *p*QMs **1** to fulfill pseudo first‐order reaction conditions (Figure 2a). Thus, first‐order rate constants *k*_obs_ (s^−1^) were determined by least squares fitting of the mono‐exponential decay function *A_t_* = *A*_0_ exp(−*k*_obs_ *t*) + *C* to the time‐dependent absorbances *A_t_* during the reaction of **1** with **2** (Figure 2b). The second‐order rate constants *k*_2_^exptl^ (M^−1^ s^−1^) were then calculated as the slopes of the linear correlations of *k*_obs_ with the carbanion concentrations, as exemplified in Figure 2c. Table 1 lists the second‐order rate constants *k*_2_ of all investigated electrophile‐nucleophile combinations (see Supporting Information and ref. [40] for details of the kinetic measurements).

**Table 1:** Experimental and calculated second‐order rate constants *k*_2_ of the reactions of *p*QMs **1** with the reference nucleophiles **2** (DMSO, 20 °C).

*δ*‐FG‐*p*QM	2	*k*_2_^exptl^ [M^−1^ s^−1^]	*k*_2_^Eq.(1)^ ^[a]^ [M^−1^ s^−1^]	*k*_2_^exptl^/*k*_2_^Eq.(1)^
**1a** (FG = H)	**2a**	1.17 × 10^2^	2.27 × 10^2^	0.51
*E* = −11.17	**2b**	1.73 × 10^3^	7.16 × 10^2^	2.4
	**2c**	6.78 × 10^3^	8.45 × 10^3^	0.80
**1b** (FG = CF_3_)	**2f**	7.92 × 10^4^ ^[b]^	1.11 × 10^5^	0.71
*E* = −11.68^[b]^	**2h**	1.03 × 10^5^ ^[b]^	1.40 × 10^5^	0.74
	**2i**	2.73 × 10^5^ ^[b]^	2.09 × 10^5^	1.3
	**2j**	5.38 × 10^5^ ^[b]^	3.56 × 10^5^	1.5
**1c** (FG = CN)	**2a**	2.29 × 10^1^	5.57 × 10^1^	0.41
*E* = −11.88	**2c**	4.98 × 10^3^	2.40 × 10^3^	2.1
	**2d**	2.68 × 10^3^	4.24 × 10^3^	0.63
	**2h**	8.90 × 10^4^	1.03 × 10^5^	0.87
	**2i**	3.71 × 10^5^	1.53 × 10^5^	2.4
**1d** (FG = CO_2_Me)	**2c**	2.22 × 10^3^	1.91 × 10^3^	1.2
*E* = −12.01	**2e**	9.55 × 10^3^	1.29 × 10^4^	0.74
	**2g**	7.58 × 10^4^	5.00 × 10^4^	1.5
	**2h**	2.53 × 10^4^	8.40 × 10^4^	0.30
	**2i**	1.04 × 10^5^	1.26 × 10^5^	0.83
	**2j**	6.68 × 10^5^	2.17 × 10^5^	3.1
**1e** (FG = Me)	**2c**	2.73 × 10^2^	9.87 × 10^1^	2.8
*E* = −13.68	**2g**	3.52 × 10^3^	3.52 × 10^3^	1.0
	**2h**	4.55 × 10^3^	6.39 × 10^3^	0.71
	**2i**	5.05 × 10^3^	9.55 × 10^3^	0.53
	**2j**	1.48 × 10^4^	1.78 × 10^4^	0.83
**1f** (FG = OMe)	**2e**	4.85 × 10^1^	7.15 × 10^1^	0.68
*E* = −15.10	**2g**	4.29 × 10^2^	3.69 × 10^2^	1.2
	**2h**	1.04 × 10^3^	7.15 × 10^2^	1.5
	**2j**	1.89 × 10^3^	2.13 × 10^3^	0.89
**1g** (FG = Ph)	**2c**	2.63	3.40	0.77
*E* = −15.58	**2e**	3.26 × 10^1^	3.19 × 10^1^	1.0
	**2g**	1.93 × 10^2^	1.72 × 10^2^	1.1
	**2h**	2.70 × 10^2^	3.41 × 10^2^	0.79
	**2i**	5.56 × 10^2^	5.09 × 10^2^	1.1
	**2j**	1.37 × 10^3^	1.04 × 10^3^	1.3

^[a]^ The second‐order rate constants *k*_2_^Eq.(1)^ were calculated by using Equation (1), reported nucleophile‐specific parameters *N* and *s*_N_ (from ref. [26]) as well as the electrophilicity parameter *E* of the *p*QMs **1** (this work).

^[b]^ From ref. [32].

### 2.3 Correlation Analysis

To determine the Mayr electrophilicity descriptors *E* of the *δ*‐functionalized *p*QMs **1** we used the second‐order rate constants *k*_2_^exptl^ (Table 1) and the previously reported nucleophile‐specific reactivity parameters (*N* and *s*_N_) of the reference nucleophiles **2** (see Scheme 1). A least‐squares analysis to minimize Δ^2^ as defined in Equation (2)^[^ ^21^ ^]^ by adjusting *E* as the only variable gave the electrophilicity *E* for each *p*QM **1**.

(2) $$\Delta^{2}=\Sigma {\left(\mathrm{lg}\;{k_{2}}^{\mathrm{exptl}}-s_{N}\left(N+E\right)\right)}^{2}$$

The ratios *k*_2_^exptl^/*k*_2_^Eq.(1)^, which are listed in the 5^th^ column of Table 1, show that using Equation (1) and the reported *N* and *s*_N_ parameters along with the electrophilicities *E* determined in this work, results in a deviation of *k*_2_^Eq.(1)^ from *k*_2_^exptl^ lower than a factor of 3.3. For practical applications in organic synthesis (see below), this error margin is of sufficient precision, in particular if one considers that both electrophilicity (*E*) and nucleophilicity (*N*) scales currently cover 40 logarithmic orders of magnitude.^[^ ^26^ ^]^ Usually, an accuracy of 1 kcal mol^−1^ (= 4.18 kJ mol^−1^) is targeted in high‐level quantum‐chemical calculations for polar reactions in solution.^[^ ^41^ ^]^ It is noteworthy, therefore, that the Gibbs energies of activation (Δ*G* ^‡^) calculated by using the three‐parameter Equation (1) agree within ± 2.9 kJ mol^−1^ with the experimentally determined energetic barriers for the electrophile‐nucleophile additions in Table 1.

**Figure 3:**
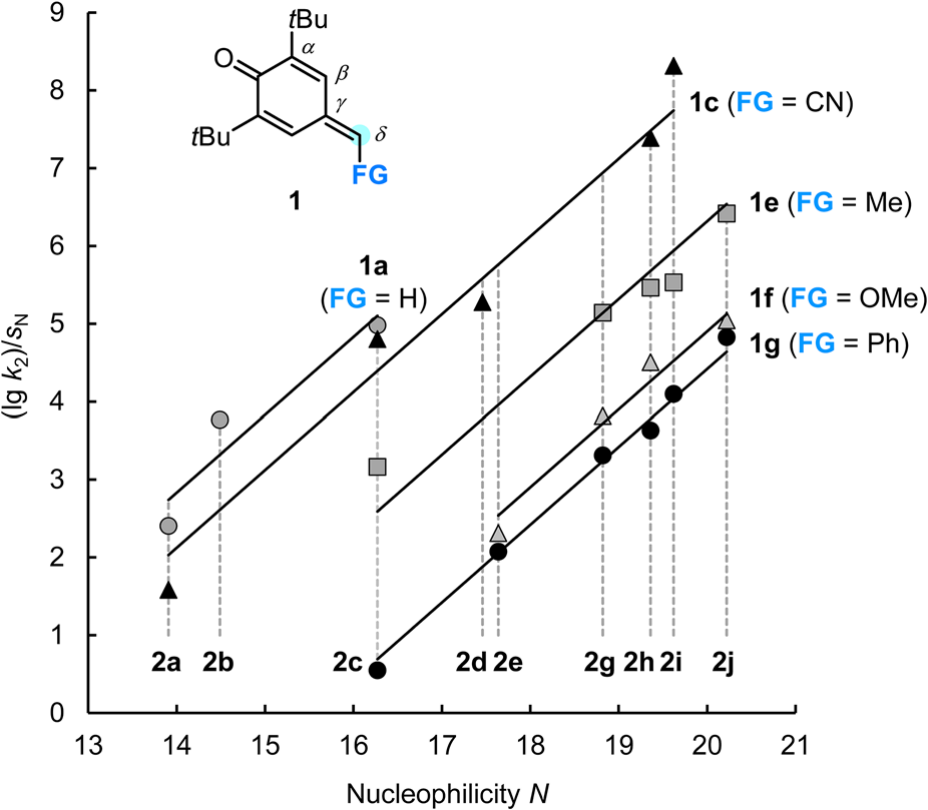
Plot of (lg *k*_2_)/*s*_N_ for the reactions of the *p*QMs **1a**, **1c**, and **1e**−**1g** with the reference nucleophiles **2** against the nucleophilicity parameters *N* of **2** (DMSO, 20 °C). The slopes of the correlation lines were enforced to unity as required by Equation (1). The correlation for **1d** is shown in Figure S4 (Supporting Information).

Figure 3 visualizes the results of the kinetic data evaluation by Equation (1) and illustrates that the experimentally determined second‐order rate constants (lg *k*_2_^exptl^)/*s*_N_ correlate linearly with the nucleophilicity parameters *N* of the C‐centered nucleophiles **2**. The slopes of these lines are enforced to unity, as required by Equation (1), and their intercepts with the abscissa [that is, (lg *k*_2_^exptl^)/*s*_N_ = 0] correspond to *E* = −*N*.

**Figure 4:**
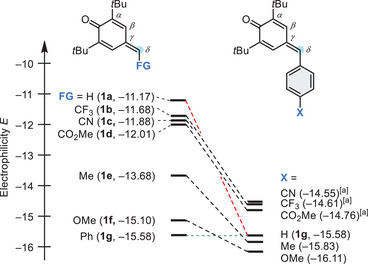
Comparison of the electrophilicities *E* of *δ*‐FG‐*p*QMs **1** with those of phenylogous *p*QMs. [a] The *E* values for X = CN, CF_3_, and CO_2_Me were extrapolated by a Hammett correlation with data from ref. [21].

On the basis of the Mayr *E* parameters, we compared the reactivity of the *δ*‐FG‐*p*QMs **1** with those of analogously substituted phenylogous *p*QMs. Figure 4 highlights that the electrophilicity of the simpler *δ*‐FG‐*p*QMs **1** is generally 1 to 4 orders of magnitude higher than that of the *δ*‐aryl‐substituted *p*QMs. The electronic substituent effects are obviously much stronger if the functional group is directly bound to the electrophilic center than being attached at a more remote position of the phenyl ring. For methyl‐ and methoxy‐substituted *p*QMs **1e** and **1f**, moderate increases in electrophilic reactivity by two and one orders of magnitude, respectively, are observed. The *p*QMs **1c** and **1d** carrying electron‐withdrawing groups, as well as the previously characterized *δ*‐trifluoromethylated **1b** ^[^ ^32^ ^]^ are by roughly three units on the *E* scale more reactive than analogously aryl‐substituted *p*QMs.

Additionally, the position of *p*QM **1a** in Figure 4 is interesting, as it outperformed the *δ*‐methoxy‐*p*QM **1f** by four orders of magnitude on the *E* scale. Moreover, **1a** is an even stronger electrophile than the *δ*‐acceptor‐substituted *p*QMs **1b** (FG = CF_3_), **1c** (FG = CN), and **1d** (FG = CO_2_Me). The sequence of *δ*‐FG‐*p*QMs **1** in Figure 4 clearly demonstrates, therefore, that electronic effects cannot be the only decisive factors that determine the reactivity of these electrophiles. In order to develop a model that reliably predicts the reactivity of *δ*‐FG‐*p*QMs **1** further dimensions have to be considered, as will be discussed below in the section on quantum‐chemical calculations.

### 2.4 Scope of *p*QM Reactions with Further Types of Nucleophiles

**Figure 5:**
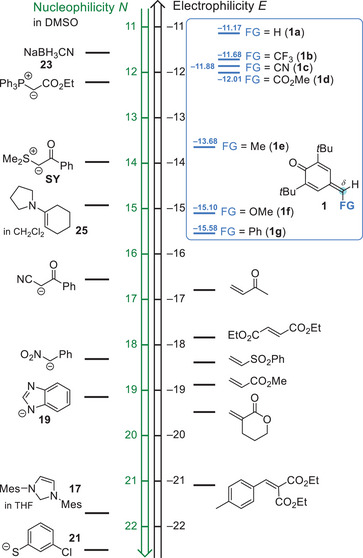
The *δ*‐FG‐*p*QMs **1** studied in this work and further Michael acceptors are ranked according to their electrophilicity parameters *E* in the right‐hand part of the combined reactivity scales for nucleophiles (left‐hand side) and electrophiles.^[^ ^26^ ^].^

After quantifying the reactivity of *δ*‐FG‐*p*QMs **1** by their Mayr electrophilicities *E*, these reactivity descriptors can now be used to rationalize reported reactions and to predict new reactions. Figure 5 shows a combination of electrophilicity and nucleophilicity scales, in which nucleophiles and electrophiles located on the same horizontal level (*E* + *N* = 0) combine with rate constants of *k* ≈ 1 M^−1^ s^−1^ at 20 °C, corresponding to half‐reaction times of 10 seconds for 0.1 M solutions. The *δ*‐FG‐*p*QMs **1** are located in a reactivity range of −11.1 < *E* < −15.6. It can, therefore, be predicted that the *δ*‐FG‐*p*QMs **1** form products easily with nucleophiles that exceed a nucleophilicity of *N* > 7 to 10.

It is in accord with these predictions that reactions of *p*QMs **1** with diazomethane (*N*/*s*_N_ = 10.48/0.78),^[^ ^42^ ^]^ sulfonium ylides^[^ ^43^ ^]^ (for example, **SY** with *N*/*s*_N_ = 13.95/0.69), the anion of α‐bromo‐malonate^[^ ^44^ ^]^ (*N*/*s*_N_ = 18.19/0.74 for α‐chloro malonate), deprotonated α‐bromo Meldrum's acid^[^ ^45^ ^]^ (*N*/*s*_N_ = 13.91/0.86 for the parent **2a**), phosphorus nucleophiles,^[^ ^46^ ^]^ such as triphenylphosphine (*N*/*s*_N_ = 14.33/0.65 in CH_2_Cl_2_) and trimethyl phosphite (*N*/*s*_N_ = 9.04/0.70 in MeOH/MeCN), have been reported in the literature.^[^ ^26^ ^]^

On a more quantitative basis, the unexpectedly high electrophilicity *E* of **1a** is corroborated by reported first‐order rate constants for the hydrolysis^[^ ^31^ ^]^ and methanolysis^[^ ^27^ ^]^ of **1a**. Table 2 shows that using the solvent nucleophilicity parameters *N*_1_ (and *s*_N_) for 50/50 (v/v) water/acetonitrile and methanol^[^ ^47^ ^]^ along with *E*(**1a**) = −11.17 from this work in Equation (1) gives calculated rate constants, which agree within one order of magnitude with the experimentally determined first‐order rate constants (*k* ^exptl^/*k* ^Eq.(1)^ = 4.2 for hydrolysis; *k* ^exptl^/*k* ^Eq.(1)^ = 0.20 for methanolysis). In addition, also the reactivity of **1a** toward hydroxide ions,^[^ ^31^, ^48^ ^]^ that is, a negatively charged O‐nucleophile, is excellently reflected by the Mayr reactivity parameters *E*, *N*, and *s*_N_ in Equation (1) (entry 3 in Table 2).

**Table 2:** Comparison of reported and predicted rate constants for reactions of O‐nucleophiles with the *p*QM **1a** (*E* = −11.17).

Entry	Nucleophile	*k* ^exptl^ ^[a]^	*N* (*s*_N_)	*k* ^Eq.(1)^ ^[b]^	*k* ^exptl^/*k* ^Eq.(1)^
1	50W50AN^[c]^	1.5 × 10^−5^ s^−1^ ^[d]^	5.05 (0.89)^[e]^	3.6 × 10^−6^ s^−1^	4.2^[f]^
2	MeOH	9.4 × 10^−5^ s^−1^ ^[g]^	7.54 (0.92)^[e]^	4.6 × 10^−4^ s^−1^	0.20^[f]^
3	HO^‐^ (in 50W50AN^[c]^)	0.18 M^−1^ s^−1^ ^[d]^	10.19 (0.62)^[h]^	0.25 M^−1^ s^−1^	0.72^[f]^

^[a]^ Experimentally determined first‐order (s^−1^) or second‐order rate constants (M^−1^ s^−1^) at 25 °C.

^[b]^ First‐order (s^−1^) or second‐order rate constants (M^−1^ s^−1^) at 20 °C as predicted by Equation (1) from *E*, *N*, and *s*_N_.

^[c]^ 50W50AN = 50/50 (v/v) water/acetonitrile.

^[d]^ At 25 °C, from ref. [31].

^[e]^ Reactivity parameters *N*_1_ and *s*_N_ as reported in ref. [47].

^[f]^ We considered the temperature difference, that is, 25 °C in the experiments and 20 °C as standard in Equation (1), to be negligible for this comparison.

^[g]^ At 25 °C, from ref. [27].

^[h]^ Reactivity parameters *N* and *s*_N_ as reported in ref. [48].

**Scheme 5:**
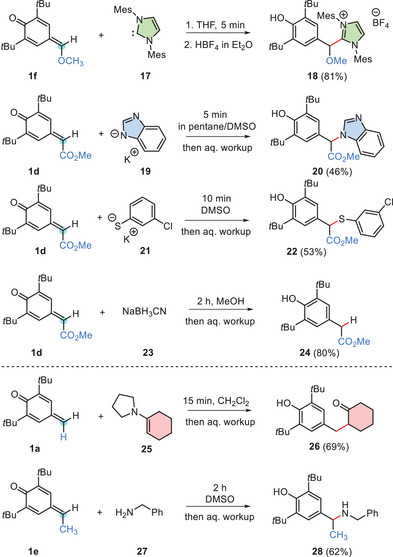
Extending the scope of reactions between *δ*‐FG‐*p*QMs **1** and carbon‐ or heteroatom‐centered nucleophiles.

To enhance the scope of nucleophilic reaction partners for the *δ*‐FG‐*p*QMs **1**, we explored further carbon‐ or heteroatom‐centered nucleophiles of different reactivity in the range from *N* = 11 to *N* = 22 (Scheme 5).

The reactions of *p*QMs **1d** and **1f** with strong nucleophiles such as the N‐heterocyclic carbene IMes (**17**, *N*/*s*_N_ = 21.72/0.45 in THF),^[^ ^49^ ^]^ the anion of benzimidazole **19** (*N*/*s*_N_ = 19.13/0.55 in DMSO),^[^ ^50^ ^]^ or the thiophenolate **21** (*N*/*s*_N_ = 22.50/0.78 in DMSO)^[^ ^51^ ^]^ gave rapidly the Michael adducts **18**, **20**, and **22**, respectively. Also, the enamine **25** (*N*/*s*_N_ = 14.91/0.86)^[^ ^26^ ^]^ and the primary amine **27** (*N*/*s*_N_ = 15.28/0.65)^[^ ^26^ ^]^ reacted with *p*QMs **1** within short times to give after aqueous workup the ketone **26** and the secondary amine **28**, respectively. Due to the relatively high reactivity of *δ*‐FG‐*p*QMs **1**, which are significantly more reactive than typical Michael acceptors such as methyl vinyl ketone, methyl acrylate, or exo‐methylene *δ*‐valerolactone (Figure 5), it was also possible to observe product formations from rather weak nucleophiles, such as the mild hydride donor sodium cyanoborohydride **23** (*N*/*s*_N_ = 11.52/0.67).^[^ ^52^ ^]^ It can therefore be concluded that the characterized electrophilicities *E* of *δ*‐FG‐*p*QMs **1** have proven useful to select in an informed way novel nucleophilic reaction partners for the *p*QMs **1**.

### 2.5 Quantum‐Chemical Calculations

**Figure 6:**
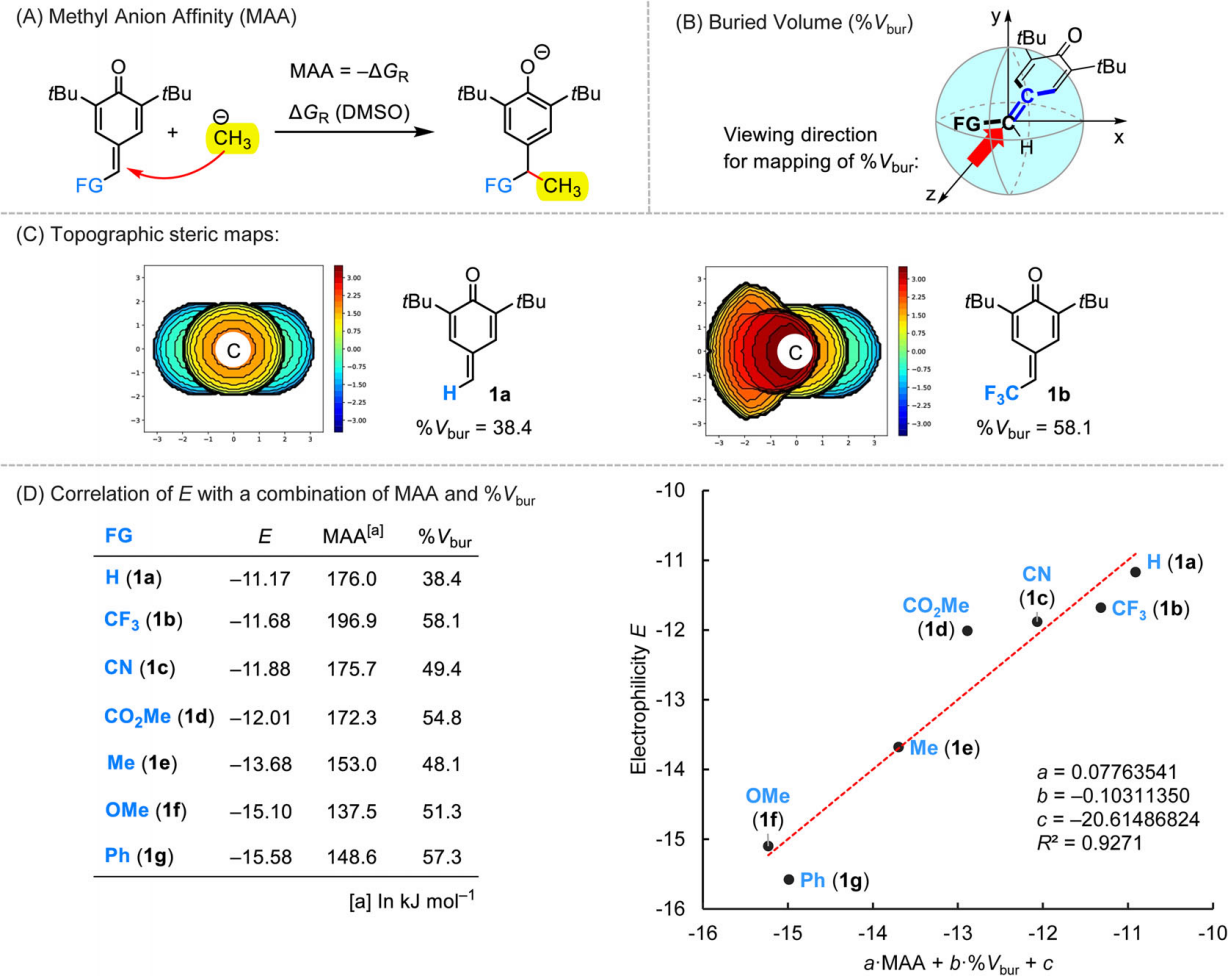
a) Definition reaction for the quantum‐chemical calculation of MAAs of *p*QMs **1** at the SMD(DMSO)/B3LYP/6–311++G(3df,2pd)//B3LYP/6–31G(d,p) level of theory. b) Definition of the viewing direction for the determination of buried volumes (%*V*_bur_). c) Topographic steric maps for **1a** and **1b** indicate the variable steric demand at the electrophilic position in *δ*‐FG‐*p*QMs **1**. d) The electrophilicity *E* of **1** correlates with a linear combination of MAA (in kJ mol^−1^) and %*V*_bur_.

We used quantum‐chemical calculations to unravel the seemingly unsystematic *δ*‐FG effects on the electrophilicity of *p*QMs **1**. Previously, the linear correlation of Mayr electrophilicities *E* with methyl anion affinities (MAAs)^[^ ^53^, ^54^, ^55^ ^]^ was shown to enable the prediction of electrophilic reactivities of typical Michael acceptors, including a series of *δ*‐aryl‐*p*QMs and *ortho*‐quinone methides when involving the continuum solvation model (SMD) in the MAA calculations.^[^ ^56^, ^57^ ^]^ Analogously, we calculated^[^ ^58^, ^59^ ^]^ the MAAs as the Gibbs reaction energies Δ*G*_R_ for the 1,6‐Michael addition of a methyl anion to the *δ*‐FG‐*p*QMs **1a**–**1g** at the SMD(DMSO)^[^ ^60^ ^]^/B3LYP/6–311++G(3df,2pd)//B3LYP/6–31G(d,p) level of theory^[^ ^61^, ^62^, ^63^ ^]^ (Figure 6a).

The calculated MAAs (in kJ mol^−1^) are tabulated in Figure 6d along with the electrophilicity parameters *E* of the *δ*‐FG‐*p*QMs **1**. Attempts to establish a linear correlation of Mayr *E* with the MAAs of the *p*QMs **1** led, however, only to a relationship of inferior quality (*R* ^2^ = 0.7725).^[^ ^33^ ^]^ This weak correlation deviates from previous results for a total of 52 Michael acceptors whose electrophilicities *E* were strongly correlated with MAA (*R* ^2^ = 0.8890).^[^ ^57^ ^]^ Already the entries for the *δ*‐FG‐*p*QMs **1a**–**1c** indicate that the experimentally determined reactivities (Mayr *E*), which differ by less than one order of magnitude, do not follow the trend of the thermodynamic driving force for the carbon–carbon bond formation that is reflected by the quantum‐chemically calculated MAAs, which differ by more than 20 kJ mol^−1^ for **1a**–**1c**.

Obviously, the steric demand of the *δ*‐FG at the *p*QMs **1** significantly affects their reactivity. We, therefore, used the optimized geometries from the quantum‐chemical MAA calculations as input data to assess buried volumes (%*V*_bur_, Figure 6b,c), from which we expected that they are a useful measure of steric effects at the reaction centers of the *δ*‐FG‐*p*QMs **1**.^[^ ^64^, ^65^, ^66^ ^]^ Comparison of the %*V*_bur_ of **1a** with that of **1b** shows that the change from a *δ*‐H‐ to a *δ*‐(trifluoromethyl)‐substituted *p*QM enhances the buried volume from 38% to 58%, which counteracts the Lewis acidity (MAA) that is significantly higher for **1b** than for **1a** (Figure 6d).

Thus, electronic and steric effects as expressed by MAA and %*V*_bur_ allow the qualitative interpretation of the electrophilic reactivity ordering of *δ*‐FG‐*p*QMs **1**. Also, quantitative predictions become possible though we have to admit that the use of only two parameters might be an oversimplification. Yet, experimentally determined electrophilicity parameters *E* for the 2,6‐di‐*tert*.‐butyl‐substituted *p*QMs showed an excellent linear relationship (*R* ^2^ = 0.9271) with a linear combination of MAAs and %*V*_bur_. The graph in Figure 6d will, therefore, also be significantly helpful for predicting the reactivity of further *δ*‐FG‐*p*QMs, for which kinetic data are currently not available.

## 3 Conclusion

In summary, we quantified the Mayr electrophilicity parameters *E* of *δ*‐functional group substituted *para‐*quinone methides through following the kinetics of their reactions with carbanions as reference nucleophiles in DMSO. Product studies support that simple electrophile‐nucleophile reactions gave rise to the photometrically observed decay of the absorbance of the quinone methides. We demonstrate that embedding and locating *δ*‐FG‐*p*QMs in Mayr's reactivity scales facilitates the informed selection of novel nucleophilic reaction partners. Thus, the data from this work will make it possible to systematically enhance the synthetic scope of *δ*‐FG‐*p*QMs toward currently unseen electrophile‐nucleophile combinations.

Variation of FG within the series of *δ*‐FG‐*p*QMs gave rise, however, to a relative reactivity ordering that could not be predicted straightforwardly. Counterintuitively, the simple 2,6‐di‐*tert*.‐butyl quinone methide **1a** (FG = H) was found to be a stronger electrophile than structurally analogous quinone methides with electron‐withdrawing groups at the electrophilic center (*δ*‐position). Quantum‐chemically calculated methyl anion affinities and buried volumes (%*V*_bur_) were, therefore, used to rationalize the relative reactivities of the studied *δ*‐FG‐*p*QMs. We show that the electrophilicities *E* of *δ*‐FG‐*p*QMs correlate linearly with a linear combination of methyl anion affinities and buried volumes (%*V*_bur_). Thus, we established a simple relationship that will support the tailored development of additional novel *p*QMs with predictable properties.

## 4 Experimental Section

### Chemicals

Supporting Information contains procedures for the preparation of *δ*‐FG‐*p*QMs **1** and the details for the reactions of **1** with anionic and neutral nucleophiles, which led to the isolated products **3**–**16**, **18**, **20**, **22**, **24**, **26**, and **28**.^[^ ^34^ ^]^

### Kinetics

The kinetics of the reactions of the *δ*‐FG‐*p*QMs **1** with the (reference) nucleophiles **2** in DMSO at 20 °C were followed by using conventional photometric or UV‐Vis stopped‐flow techniques. Details of the kinetic experiments are given in the Supporting Information and in ref. [40].

### Quantum‐Chemical Calculations

Details are reported in the Supporting Information.

## Supporting Information

The authors have cited additional references within the Supporting Information.^[^ ^67^, ^68^, ^69^, ^70^, ^71^, ^72^, ^73^, ^74^, ^75^, ^76^, ^77^, ^78^, ^79^, ^80^ ^]^

## Conflict of Interest

The authors declare no conflict of interest.

## Supporting information



Supporting Information

## Data Availability

The raw data of kinetic measurements that support the findings of this study are openly available in Open Data LMU at DOI: 10.5282/ubm/data.582, ref. [40]. Further data available in article supplementary information.
