# Supplementary material for: Reactivity of δ‐Functionalized Para‐Quinone Methides in Nucleophilic Addition Reactions
Source: Chemistry. 2025 Jun 25;31(40):e202501224. doi: 10.1002/chem.202501224 (PMC12272005; doi:10.1002/chem.202501224)
Supplement: Supplementary file 1 — Supporting Information [file CHEM-31-e202501224-s001.pdf]

– Supporting Information –

Table of Contents

1. General S2

2. Synthesis of *para*-Quinone Methides (*p*QMs) S3

3. Products of Reactions of *p*QMs with C-Nucleophiles S6

4. Products of Reactions of *p*QMs with Further Classes of Nucleophiles S17

5. UV/Vis Spectra and Molar Absorption Coefficients of *p*QMs S21

6. Cyclic Voltammetry: Reduction Potentials of *p*QMs S27

7. Single Crystal X-Ray Structure Determinations S30

8. Kinetics of the Reactions of *p*QMs with Carbanions S37

9. Electrophilicities *E* of *p*QMs **1a**–**1g** S52

10. Copies of ^{1}H and ^{13}C{^{1}H} NMR Spectra S55

11. Quantum-Chemical Calculations S83

1. General

Commercial reagents and dry solvents (stored over molecular sieves) were used without further purification as purchased from Sigma-Aldrich or Acros Organics. For thin-layer chromatography, silica gel plates with F-254 fluorescence indicator (Merck) were used. Purification by flash column chromatography was performed using Merck silica gel 60 (0.040–0.063 mm) with freshly distilled solvents.

Melting points were acquired using Büchi Melting Point B-560 devices and are not corrected.

Nuclear magnetic resonance (NMR) spectra were recorded on 400 or 600 MHz spectrometers. NMR signals were assigned based on information from additional 2D NMR experiments (COSY, gHSQC, gHMBC). Internal reference was set to the residual solvent signals (δ_H = 7.26 ppm, δ_C = 77.16 ppm for $CDCl_3$; δ_H = 2.50 ppm, δ_C = 39.52 ppm for DMSO-d_6; δ_H = 5.32 ppm, δ_C = 53.84 ppm for CD_2Cl_2; δ_H = 1.94 ppm, δ_C = 1.32 ppm for CD_3CN).

Infrared (IR) spectra were recorded on a Perkin Elmer Spectrum BX-59343 instrument with a Smiths Detection DuraSamplIR II Diamond ATR sensor or a Bruker Tensor 27 FT-IR instrument with a "Platinum" Diamond ATR sensor for detection in the range 4500–600 cm^{-1} as a film for liquids or neat for solids.

High resolution (HRMS) mass spectra were recorded on a Finnigan MAT 90, a Finnigan MAT 95, a JEOL MStation JMS 700, a Thermo Finnigan LTQ FT Ultra Fourier Transform ion cyclotron resonance, a Q Exactive GC Orbitrap GC/MS or a Thermo Fisher Scientific LTQ Orbitrap XL. For ionization of the samples, either electron-impact ionization (EI) or electrospray ionization (ESI) was applied.

UV/Vis measurements were carried out using a J&M TIDAS diode array spectrophotometer, which was controlled by TIDASDAQ3 (v3) software and connected to a Hellma 661.502-QX quartz Suprasil immersion probe (light path d = 5 mm) via fiber optic cables and standard SMA connectors.

Cyclovoltammetry was performed on a CH Instruments 630E electrochemical analyzer.

Kinetic measurements were performed by using UV/Vis photometry on AppliedPhotophysics SX.20 stopped-flow instruments as well as on a conventional J&M TIDAS diode array spectrophotometer, which was controlled by TIDASDAQ3 (v3) software and connected to a Hellma 661.502-QX quartz Suprasil immersion probe (light path d = 5 mm) via fiber optic cables and standard SMA connectors. The temperature (20.0 ± 0.2 °C) was maintained constant by using circulating bath cryostats.

2. Synthesis of *para*-Quinone Methides (*p*QMs)

2,6-Di-*tert*-butyl-4-methylenecyclohexa-2,5-dien-1-one (1a) was synthesized by oxidation of 3,5-di-*tert*-butyl-4-hydroxytoluene (BHT) as described in ref. [30] but with slight modifications. For NMR analysis the oxidation was performed in two ways:

(a) In a dry N_2-atmosphere, BHT (20.4 mg, 0.093mmol) was stirred with Ag_2O (300 mg, 1.30 mmol) in $CDCl_3$ (2 mL) or CCl_4 (2 mL) under the exclusion of light for 20 min. Then solids were filtered off (0.2 µm PTFE filter) before NMR spectroscopic analysis of the filtrate.

(b) BHT (21.6 mg, 0.098 mmol) was stirred with Ag_2O (300 mg, 1.30 mmol) in *n*-pentane (2 mL) under the exclusion of light for 20 min under N_2-atmosphere. Then solids were filtered off (0.2 µm PTFE filter). The thus generated pentane solution of *p*QM **1a** (1 mL) was mixed with DMSO-d_6 (1 mL). The *n*-pentane was evaporated under vacuum. The *p*QM **1a** remained dissolved in DMSO-d_6 and was used without further purification for NMR spectroscopic analysis.

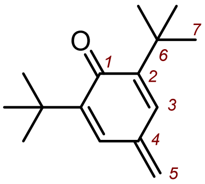

1a (CG446_CDCl3)

^{1}H NMR (400 MHz, $CDCl_3$): δ = 6.94 (s, 2 H, 3-H), 5.77 (s, 2 H, 5-H), 1.28 (s, 18 H, 7-H).

^{13}C{^{1}H} NMR (101 MHz, $CDCl_3$): δ = 186.5, 148.7, 139.0, 132.8, 128.3, 35.1, 29.6.

^{1}H NMR (400 MHz, DMSO-d_6): δ = 7.12 (s, 2 H, 3-H), 6.09 (s, 2 H, 5-H), 1.23 (s, 18 H, 7-H).

^{13}C{^{1}H} NMR (101 MHz, DMSO-d_6): δ = 185.8, 147.3, 137.9, 133.2, 131.8, 34.7, 29.2.

^{1}H NMR (400 MHz, CCl_4):[1] δ = 6.76 (s, 2 H), 5.58 (s, 2 H), 1.19 (s, 18 H).

^{13}C{^{1}H} NMR (101 MHz, CCl_4): δ = 185.4, 149.0, 139.4, 132.2, 126.8, 35.4, 29.9.

HRMS (EI): *m/z* calcd for $C_{15}H_{22}O^{\bullet+}$ $[M]^{\bullet+}$ 218.1665; found: 218.1663.

For photometric experiments, *p*QM **1a** was generated under the exclusion of light by stirring BHT (18.6 mg, 0.084 mmol) with Ag_2O (100 mg, 0.432 mmol) in *n*-pentane (3 mL) for 20 minutes. Then, solids were removed by filtration (0.2 µm PTFE filter) and an aliquot of the solution (0.5 mL) was mixed with DMSO (1.0 mL). The *n*-pentane was evaporated under vacuum. The *p*QM **1a** remained dissolved in DMSO. The thus prepared stock solution of **1a** in DMSO was used for kinetic experiments and for determining the extinction coefficient of **1a**. According to the NMR analysis in (b), *p*QM **1a** formed quantitatively from the BHT. Consequently, the molar amount of **1a** used in photometric experiments was calculated assuming a complete conversion of BHT to *p*QM **1a**.

(1) For NMR spectroscopic analysis in CCl_4, a capillary tube filled with $CDCl_3$ was added into the NMR tube to ensure proper shimming. Chemical shifts of NMR resonances were calibrated according to the residual solvent signal of $CDCl_3$.

2-(3,5-Di-*tert*-butyl-4-oxocyclohexa-2,5-dien-1-ylidene)acetonitrile (1c) was synthesized according to ref. [67] NMR spectroscopic data in $CDCl_3$ agree with those described in ref. [67].

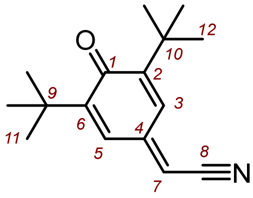

1c (CG067)

^{1}H NMR (400 MHz, $CDCl_3$): δ = 7.32 (d, J = 2.5 Hz, 1 H, 5-H), 6.85 (d, J = 2.5 Hz, 1 H, 3-H), 5.66 (s, 1 H, 7-H), 1.31 (s, 9 H, 11-H), 1.28 (s, 9 H, 12-H).

^{13}C{^{1}H} NMR (101 MHz, $CDCl_3$): δ = 186.1 (C_q, C-1), 152.8 (C_q, C-6), 152.1 (C_q, C-2), 147.5 (C_q, C-4), 130.6 (CH, C-3), 127.3 (CH, C-5), 116.4 (C-8), 103.6 (CH, C-7), 35.9 (C_q, C-9), 35.7 (C_q, C-10), 29.6 (CH_3, C-11), 29.5 (CH_3, C-12).

HRMS (EI): m/z calcd for $C_{16}H_{21}NO^{\bullet+}$ $[M]^{\bullet+}$ 243.1618; found: 243.1618.

IR (neat, ATR): $\tilde{\nu}$ = 2959, 2207, 1630, 1617, 1582, 1363, 1253, 1084, 914, 879, 817, 743 cm^{-1}.

Methyl 2-(3,5-di-*tert*-butyl-4-oxocyclohexa-2,5-dien-1-ylidene)acetate (1d) was synthesized according to ref. [67] NMR spectroscopic data in $CDCl_3$ agree with those described in ref. [67].

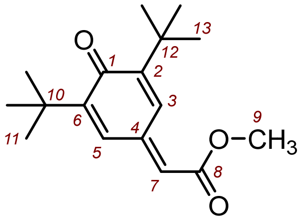

1d (CG475)

^{1}H NMR (400 MHz, $CDCl_3$): δ = 8.30 (d, J = 3.1 Hz, 1 H), 6.78 (d, J = 2.4 Hz, 1 H), 6.14 (s, 1 H, 7-H), 3.81 (s, 3 H, 9-H), 1.30 (s, 9 H), 1.28 (s, 9 H).

^{13}C{^{1}H} NMR (101 MHz, $CDCl_3$): δ = 186.8 (C_q, C-1), 166.4, 151.6, 151.4, 142.8, 133.6, 127.2, 125.1, 52.0, 35.8, 35.4, 29.7, 29.6.

HRMS (EI): m/z calcd for $C_{17}H_{24}O_3^{\bullet+}$ $[M]^{\bullet+}$ 276.1720; found: 276.1718.

IR (neat, ATR): $\tilde{\nu}$ = 2953, 1706, 1627, 1431, 1278, 1240, 1171, 1013, 932, 921, 860, 821 cm^{-1}.

2,6-Di-tert-butyl-4-ethylidenecyclohexa-2,5-dien-1-one (1e) was synthesized according to ref. [68] NMR spectroscopic data in $CDCl_3$ agree with those described in ref. [68].

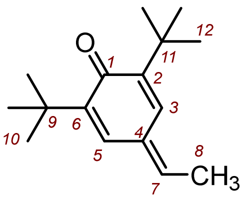

1e (TR01)

^{1}H NMR (600 MHz, $CDCl_3$): δ = 7.30 (d, J = 2.5 Hz, 1 H), 6.84 (d, J = 2.6 Hz, 1 H), 6.41 (q, J = 7.7 Hz, 1 H, 7-H), 2.12 (d, J = 7.6 Hz, 3 H, 8-H), 1.31 (s, 9 H), 1.28 (s, 9 H).

^{13}C{^{1}H} NMR (151 MHz, $CDCl_3$): δ = 186.9 (C_q, C-1), 148.2 (C_q), 146.4 (C_q), 142.8 (CH, C-7), 135.1 (CH), 132.8 (C_q, C-4), 125.9 (CH), 35.5 (C_q), 34.9 (C_q), 29.60 (CH_3), 29.56 (CH_3), 15.2 (CH_3, C-8).

HRMS (EI): *m/z* calcd for $C_{16}H_{24}O^{•+}$ $[M]^{•+}$ 232.1822; found: 232.1823.

IR (neat, ATR): $\tilde{\nu}$ = 2950, 1613, 1586, 1572, 1452, 1434, 1358, 1250, 902, 883, 815, 746 cm^{-1}.

2,6-Di-*tert*-butyl-4-(methoxymethylene)cyclohexa-2,5-dien-1-one (1f) was synthesized according to ref. [67] NMR spectroscopic data in $CDCl_3$ agree with those described in ref. [67].

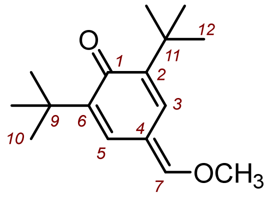

1f (CG476_CDCl3)

^{1}H NMR (600 MHz, $CDCl_3$): δ = 7.39 (d, *J* = 3.2 Hz, 1 H), 6.88 (s, 1 H, 7-H), 6.82 (d, *J* = 2.4 Hz, 1 H), 3.98 (s, 3 H, 7-OCH_3), 1.30 (s, 9 H), 1.28 (s, 9 H).

^{13}C{^{1}H} NMR (151 MHz, $CDCl_3$): δ = 186.2 (C_q, C-1), 159.4 (CH, C-7), 147.3 (C_q), 145.6 (C_q), 131.1 (CH), 124.0 (CH), 114.6 (C_q, C-4), 62.4 (CH_3, 7-OCH_3), 35.4 (C_q), 35.0 (C_q), 29.6 (CH_3), 29.5 (CH_3).

HRMS (EI): *m/z* calcd for $C_{16}H_{24}O_2^{•+}$ $[M]^{•+}$ 248.1771; found: 248.1772.

IR (neat, ATR): $\tilde{\nu}$ = 2951, 1635, 1552, 1357, 1254, 1151, 1018, 987, 948, 915, 883, 818 cm^{-1}.

4-Benzylidene-2,6-di-*tert*-butylcyclohexa-2,5-dien-1-one (1g) was synthesized analogously to a reported procedure for aryl substituted *p*QMs.[69] NMR spectroscopic data in $CDCl_3$ agree with those described in ref. [70].

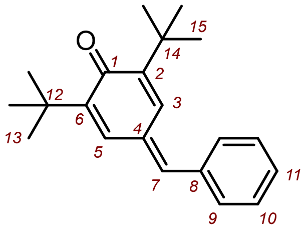

1g (CG095)

^{1}H NMR (400 MHz, $CDCl_3$): δ = 7.53 (d, *J* = 3.3 Hz, 1 H), 7.49–7.35 (m, 5 H, 9-H, 10-H, and 11-H), 7.20 (s, 1 H, 7-H), 7.02 (d, *J* = 2.6 Hz, 1 H), 1.34 (s, 9 H), 1.31 (s, 9 H).

^{13}C{^{1}H} NMR (101 MHz, $CDCl_3$): δ = 186.7 (C_q, C-1), 149.5, 147.9, 142.7, 136.1, 135.3, 132.1, 130.5, 129.2, 128.9, 127.9, 35.6, 35.2, 29.68, 29.65.

HRMS (EI): *m/z* calcd for $C_{21}H_{26}O^{•+}$ $[M]^{•+}$ 294.1978; found: 294.1976.

3. Products of Reactions of *p*QMs with C-Nucleophiles

General Procedure 1 (GP1) for Reactions of *p*QMs **1** with C-Nucleophiles **2**

A solution of nucleophile **2** (1.0 to 1.3 equiv.) in DMSO-d_6 (1.0 mmol) was transferred to the quinone methide **1** in a standard GC vial. The reaction solution was mixed using a sonicator. Then the reaction mixture was left for up to 4 h, depending on the nucleophile used. The mixture was quenched with saturated ammonium chloride solution (1 mL), diluted with water (5 mL) and extracted with diethyl ether (4 × 10 mL). The combined organic phases were washed with water (4 × 10 mL) and dried over $MgSO_4$. The solvent was removed under reduced pressure and the crude residue was either purified by silica gel chromatography or recrystallized to afford the reaction product.

Reactions of *p*QM 1a and Carbanion 2j

(A) Isolation of cyclized products 3 and 4 from the reaction in CCl_4/DMSO solution (CG536)

2,6-Di-*tert*-butyl-4-methylphenol (BHT) (60.0 mg, 0.272 mmol) was dissolved in CCl_4 (2 mL) and Ag_2O (315 mg, 1.36 mmol) was added. The mixture was stirred for 20 min at room temperature under the exclusion of light. Solids were removed by filtration (0.2 µm PTFE filter) and a DMSO solution of **2j** (59.4 mg, 0.300 mmol in 10 mL) was added. The mixture was stirred for 1 h at room temperature under nitrogen atmosphere. The reaction was quenched with sat. NH_4Cl solution (5 mL), diluted with water (5 mL), and extracted with diethyl ether (4 × 10 mL). The combined organic phases were washed with water (4 × 10 mL) and dried over $MgSO_4$. After filtration, the solvent was removed under reduced pressure yielding a residue that was purified by preparative TLC (silica gel, eluent: *n*-pentane:EtOAc 97:3) to isolate **3** (31.5 mg, 31%) as a colorless solid (m.p. 95 °C) and **4** (41.0 mg, 51%) as a colorless solid (m.p. 187 °C).

(B) Isolation of Michael adducts 5 and 6 from the reaction in *n*-pentane/DMSO solution (CG536_4)

2,6-Di-*tert*-butyl-4-methylphenol (BHT) (65.0 mg, 0.295 mmol) was dissolved in *n*-pentane (2 mL) and Ag_2O (335 mg, 1.45 mmol) was added. The mixture was stirred for 20 min at room temperature under the exclusion of light. Solids were removed by filtration (0.2 µm PTFE filter) and a DMSO solution of **2j** (66.0 mg, 0.333 mmol in 10 mL) was added. The mixture was stirred for 1 h at room temperature under nitrogen atmosphere. The reaction was quenched with sat. NH_4Cl solution (5 mL), diluted with water (5 mL) and extracted with diethyl ether (4 × 10 mL). The combined organic phases were washed with water (4 × 10 mL) and dried over $MgSO_4$. After filtration, the solvent was removed under reduced pressure yielding a residue that was purified by preparative TLC (silica gel, *n*-pentane:EtOAc 95:5) to give **5** (48.0 mg, 43%) as a white solid (m.p. 156°C) and **6** (10.4 mg, 12%) as a white solid (m.p. 162°C).

(C) Conversion of 6 to 4 under the conditions of reaction (A) (CG646)

The 2,2-alkylated diethyl malonate **6** (20.0 mg, 0.034 mmol) was dissolved in CCl_4 (2 mL) and silver(I) oxide (78 mg, 0.34 mmol) was added. The mixture was stirred for 20 min at room temperature under the exclusion of light. Solids were removed by filtration (0.2 µm PTFE filter) and DMSO (5 mL) was added. The mixture was stirred for 2 h at room temperature under nitrogen atmosphere. The reaction

was quenched with sat. NH_4Cl solution (5 mL), diluted with water (5 mL), and extracted with diethyl ether (4 × 10 mL). The combined organic phases were washed with water (4 × 10 mL) and dried over $MgSO_4$. After filtration, the solvent was removed under reduced pressure to give the cyclic **4** (19.0 mg, 94%) as a colorless solid, which was analyzed by ^{1}H NMR spectroscopy (Figure S1).

^{1}H NMR (400 MHz, $CDCl_3$): δ = 6.46 (s, 4 H), 4.28 (q, *J* = 7.2 Hz, 4 H), 2.79 (s, 4 H), 1.29 (t, *J* = 7.1 Hz, 6 H), 1.15 ppm (s, 36 H). Identical to the ^{1}H NMR spectrum of **4** isolated by following the procedure of the reaction described in (A).

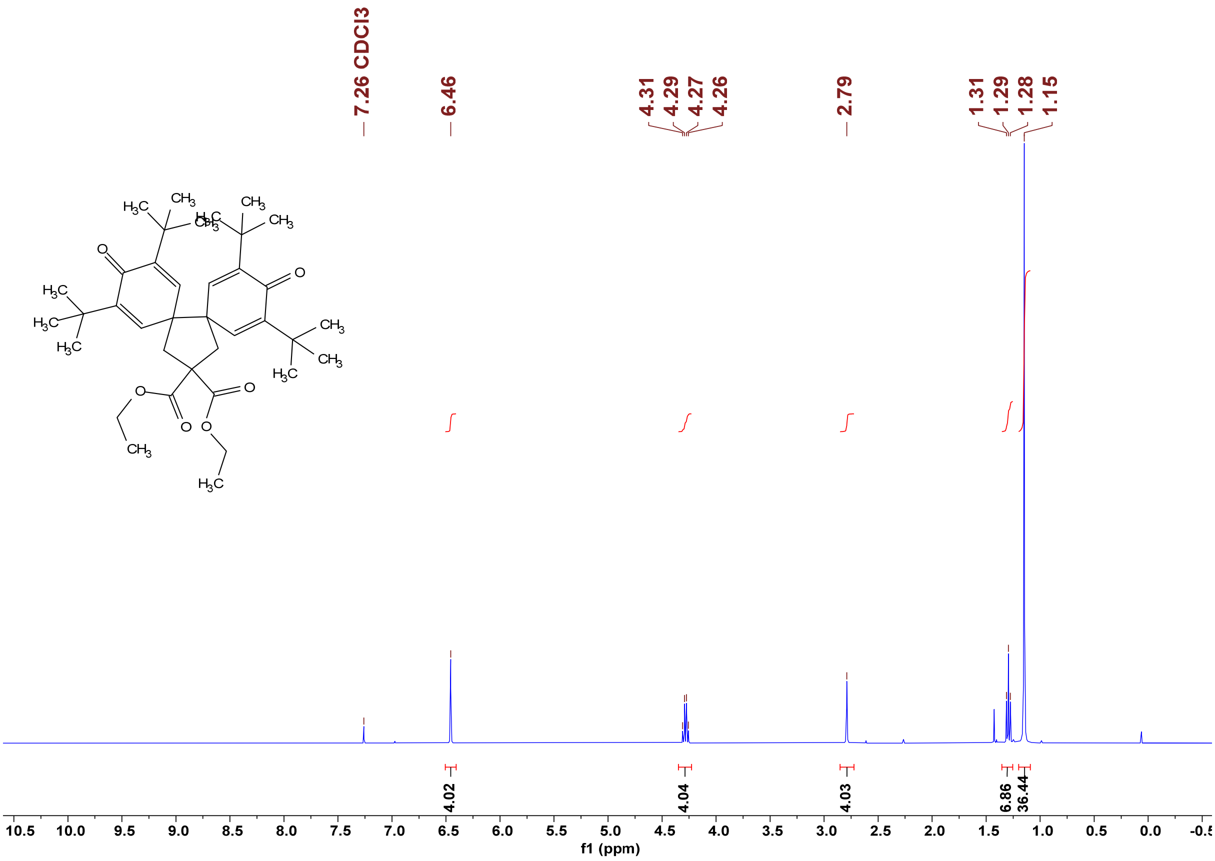

Figure S1. ^{1}H NMR spectrum of **4** in $CDCl_3$ (400 MHz) CG646

Diethyl 5,7-di-*tert*-butyl-6-oxospiro[2.5]octa-4,7-diene-1,1-dicarboxylate (3)

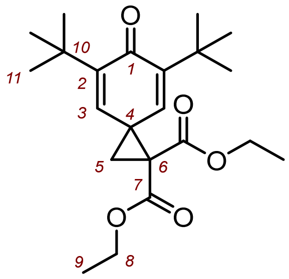
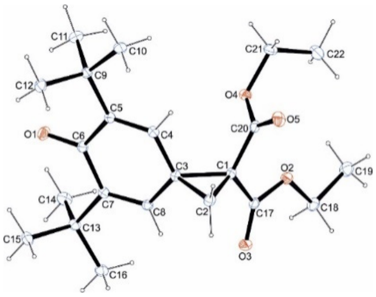

3 (CG536_1F20-29)

R_f (*n*-pentane/EtOAc 97:3, silica, UV) = 0.40.

^{1}H NMR (400 MHz, $CDCl_3$): δ = 6.37 (s, 2 H, 3-H), 4.31–4.19 (m, 4 H, 8-H), 2.29 (s, 2 H, 5-H), 1.30 (t, *J* = 7.1 Hz, 6 H, 9-H), 1.22 (s, 18 H, 11-H).

^{13}C{^{1}H} NMR (101 MHz, $CDCl_3$): δ = 185.7 (C_q, C-1), 167.1 (C_q, C-7), 150.3 (C_q, C-2), 137.0 (CH, C-3), 62.5 (CH_2, C-8), 45.0 (C_q, C-6), 36.2 (C_q, C-4), 35.4 (C_q, C-10), 29.4 (CH_3, C-11), 27.7 (CH_2, C-5), 14.3 (CH_3, C-9).

HRMS (pos. ESI): *m/z* calcd for $C_{22}H_{32}NaO_5^+$ [M + Na^+]: 399.2142; found: 399.2161.

IR (neat, ATR): $\tilde{\nu}$ = 2958, 1729, 1652, 1632, 1459, 1371, 1310, 1265, 1210, 1107, 1022, 1002 cm^{-1}.

Diethyl 2,4,9,11-tetra-*tert*-butyl-3,10-dioxodispiro[5.0.5^7.3^6]pentadeca-1,4,8,11-tetraene-14,14-dicarboxylate (4)

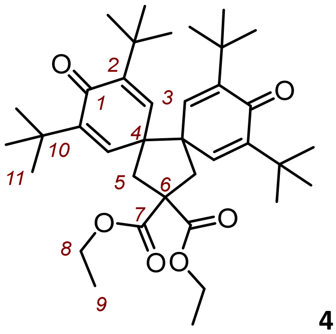
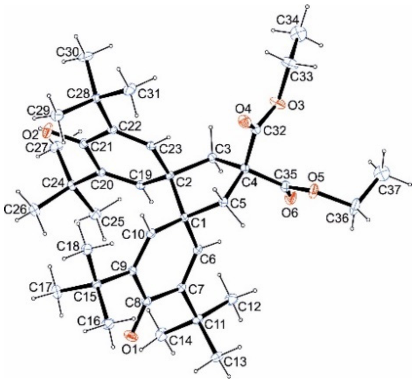

4 (CG536_1F10-16)

R_f (*n*-pentane/EtOAc 97:3, silica, UV) = 0.45.

^{1}H NMR (400 MHz, $CDCl_3$): δ = 6.46 (s, 4 H, 3-H), 4.28 (q, *J* = 7.1 Hz, 4 H, 8-H), 2.79 (s, 4 H, 5-H), 1.29 (t, *J* = 7.1 Hz, 6 H, 9-H), 1.15 (s, 36 H, 11-H).

^{13}C{^{1}H} NMR (101 MHz, $CDCl_3$): δ = 185.9 (C_q, C-1), 172.2 (C_q, C-7), 148.8 (C_q, C-2), 139.9 (CH, C-3), 62.5 (CH_2, C-8), 59.7 (C_q, C-6), 56.6 (C_q, C-4), 43.8 (CH_2, C-5), 35.1 (C_q, C-10), 29.6 (CH_3, C-11), 14.2 (CH_3, C-9).

HRMS (EI): *m/z* calcd for $[C_{37}H_{54}O_6]^{\bullet+}$ [$M^{\bullet+}$] 594.3915; found: 594.3911.

IR (neat, ATR): $\tilde{\nu}$ = 2956, 1728, 1660, 1641, 1459, 1368, 1255, 1181, 1161, 1097, 1075, 881 cm^{-1}.

Diethyl 2-(3,5-di-*tert*-butyl-4-hydroxybenzyl)malonate (5)

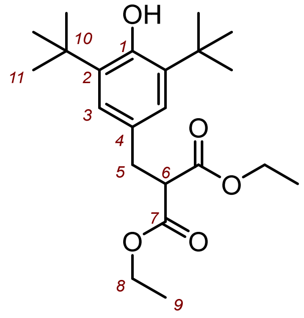

5 (CG536_4_2)

^{1}H NMR (600 MHz, $CDCl_3$): δ = 6.98 (s, 2 H, 3-H), 5.09 (s, 1 H, 1-OH), 4.16 (q, J = 7.1 Hz, 4 H, 8-H), 3.61 (t, J = 7.8 Hz, 1 H, 6-H), 3.14 (d, J = 7.9 Hz, 2 H, 5-H), 1.42 (s, 18 H, 11-H), 1.21 (t, J = 7.1 Hz, 6 H, 9-H).

^{13}C{^{1}H} NMR (151 MHz, $CDCl_3$): δ = 169.1 (C_q, C-7), 152.5 (C_q, C-1), 135.8 (C_q, C-2), 128.4 (C_q, C-4), 125.3 (CH, C-3), 61.3 (CH_2, C-8), 54.2 (CH, C-6), 34.7 (CH_2, C-5), 34.3 (C_q, C-10), 30.3 (CH_3, C-11), 14.0 (CH_3, C-9).

HRMS (EI): *m/z* calcd for $C_{22}H_{34}O_5^{\bullet+}$ [$M^{\bullet+}$] 378.2401; found: 378.2401.

IR (neat, ATR): $\tilde{\nu}$ = 3641, 2957, 1728, 1435, 1368, 1232, 1214, 1146, 1119, 1034, 865, 770 cm^{-1}.

Diethyl 2,2-bis(3,5-di-*tert*-butyl-4-hydroxybenzyl)malonate (6)

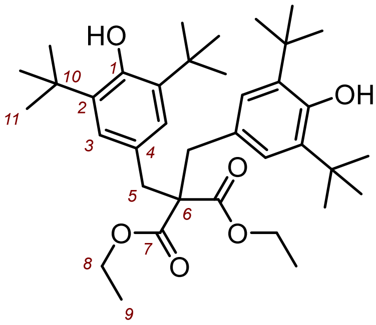

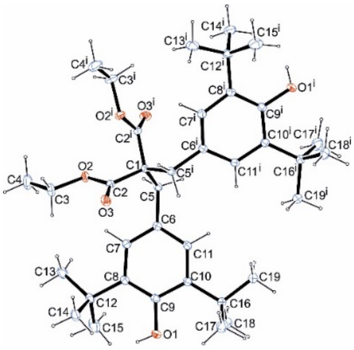

6 (CG536_4_1)

^{1}H NMR (600 MHz, $CDCl_3$): δ = 7.02 (s, 4 H, 3-H), 5.09 (s, 2 H, 1-OH), 4.03 (q, J = 7.1 Hz, 4 H, 8-H), 3.17 (s, 4 H, 5-H), 1.41 (s, 36 H, 11-H), 1.10 (t, J = 7.2 Hz, 6 H, 9-H).

^{13}C{^{1}H} NMR (151 MHz, $CDCl_3$): δ = 171.3 (C_q, C-7), 152.7 (C_q, C-1), 135.5 (C_q, C-2), 127.3 (C_q, C-4), 126.8 (CH, C-3), 61.1 (CH_2, C-8), 60.8 (C_q, C-6), 39.1 (CH_2, C-5), 34.4 (C_q, C-10), 30.5 (CH_3, C-11), 14.0 (CH_3, C-9).

HRMS (pos. ESI): *m/z* calcd for $C_{37}H_{57}O_6^{+}$ [$M + H^{+}$] 597.4150; found: 597.4139.

IR (neat, ATR): $\tilde{\nu}$ = 3587, 2954, 1728, 1434, 1305, 1232, 1195, 1141, 1065, 982, 884, 771 cm^{-1}.

5-(3,5-Di-*tert*-butyl-4-hydroxybenzyl)-2,2-dimethyl-1,3-dioxane-4,6-dione (7)

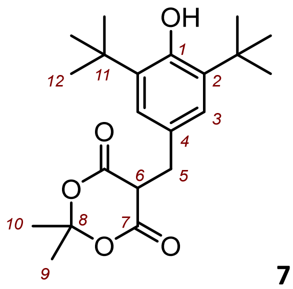

7 (CG542)

BHT (64.0 mg, 0.29 mmol) was dissolved in *n*-pentane (2 mL) and silver(I) oxide (337 mg, 1.45 mmol) was added. The mixture was stirred for 20 min at room temperature under the exclusion of light. Solids were removed by filtration (0.2 µm PTFE filter) and a DMSO solution of **2a** (61.9 mg, 0.34 mmol in 10 mL) was added. The mixture was stirred for 1 h at room temperature under nitrogen atmosphere. The reaction was quenched with aq. sat. NH_4Cl solution (5 mL), diluted with water (5 mL) and extracted with diethyl ether (4 × 10 mL). The combined organic phases were washed with water (4 × 10 mL) and dried over $MgSO_4$. The solvent was removed under reduced pressure yielding a crude product that was purified by preparative TLC (silica gel, *n*-pentane:EtOAc 9:1→8:2→7:3) to give **7** as a white solid (69.0 mg, 66%); m.p. 175 °C.

R_f (*n*-pentane/EtOAc 9:1, silica, UV) = 0.15.

^{1}H NMR (400 MHz, $CDCl_3$): δ = 7.10 (s, 2 H, 3-H), 5.15 (s, 1 H, 1-OH), 3.72 (t, J = 4.7 Hz, 1 H, 6-H), 3.43 (d, J = 4.8 Hz, 2 H, 5-H), 1.70 (s, 3 H, 9-H or 10-H), 1.41 (s, 18 H, 12-H), 1.28 (s, 3 H, 9-H or 10-H).

^{13}C{^{1}H} NMR (101 MHz, $CDCl_3$): δ = 165.9 (C_q, C-7), 153.1 (C_q, C-1), 136.3 (C_q, C-2), 127.8 (C_q, C-4), 126.6 (CH, C-3), 105.4 (C_q, C-8), 48.6 (CH, C-6), 34.4 (C_q, C-11), 32.9 (CH_2, C-5), 30.4 (CH_3, C-12), 28.6 (CH_3, C-9 or C-10), 27.7 (CH_3, C-9 or C-10).

HRMS (EI): m/z calcd for $C_{21}H_{30}O_5^{\bullet+}$ [$M^{\bullet+}$]: 362.2088; found: 362.2090.

IR (neat, ATR): $\tilde{\nu}$ = 3638, 2957, 1744, 1435, 1393, 1344, 1281, 1200, 1088, 1016, 948, 909, 730 cm^{-1}.

Ethyl 3-(3,5-di-*tert*-butyl-4-hydroxyphenyl)-2-nitropropanoate (8)

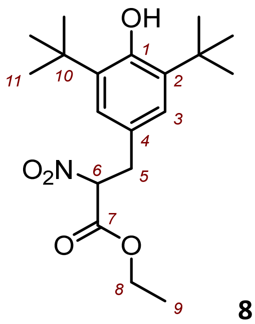

8 (CG543)

BHT (66.1 mg, 0.30 mmol) was dissolved in *n*-pentane (2 mL) and silver(I) oxide (348 mg, 1.50 mmol) was added. The mixture was stirred for 20 min at room temperature under the exclusion of light. Solids were removed by filtration (0.2 µm PTFE filter) and DMSO solution of **2k** was added [generated by mixing KO*t*Bu (37.0 mg, 0.33 mmol) and ethyl 2-nitroacetate (45.9 mg, 0.34 mmol) in 10 mL DMSO]. The mixture was vigorously stirred for 1 h at room temperature under nitrogen atmosphere. The reaction was quenched with aq. sat. NH_4Cl solution (5 mL), diluted with water (5 mL) and extracted with diethyl ether (4 × 10 mL). The combined organic phases were washed with water (4 × 10 mL) and dried over $MgSO_4$. The solvent was removed under reduced pressure to furnish a residue that was

purified by preparative TLC (silica gel, *n*-pentane:EtOAc 95:5→9:1) to give **8** as a white solid (92.0 mg, 87%); m.p. 67 °C.

R_f (*n*-pentane/EtOAc 95:5, silica, UV) = 0.40.

^{1}H NMR (600 MHz, $CDCl_3$): δ = 6.97 (s, 2 H, 3-H), 5.30 (dd, *J* = 9.5, 5.8 Hz, 1 H, 6-H), 5.18 (s, 1 H, 1-OH), 4.29–4.25 (m, 2 H, 8-H), 3.48 (dd, *J* = 14.7, 9.5 Hz, 1 H, 5-H^a), 3.39 (d, *J* = 14.7, 5.9 Hz, 1 H, 5-H^b), 1.41 (s, 18 H, 11-H), 1.27 (t, *J* = 7.1 Hz, 3 H, 9-H).

^{13}C{^{1}H} NMR (151 MHz, $CDCl_3$): δ = 164.5 (C_q, C-7), 153.4 (C_q, C-1), 136.5 (C_q, C-2), 125.6 (CH, C-3), 124.8 (C_q, C-4), 89.7 (CH, C-6), 63.2 (CH_2, C-8), 36.4 (CH_2, C-5), 34.4 (C_q, C-10), 30.3 (CH_3, C-11), 14.0 (CH_3, C-9).

HRMS (EI): *m/z* calcd for $C_{19}H_{29}NO_5^{\bullet+}$ [$M^{\bullet+}$] 351.2040; found: 351.2038.

IR (neat, ATR): $\tilde{\nu}$ = 3627, 2957, 1746, 1566, 1434, 1368, 1317, 1270, 1211, 1147, 1117, 1028 cm^{-1}.

2-(3,5-Di-*tert*-butyl-4-hydroxybenzyl)-5,5-dimethylcyclohexane-1,3-dione (9)

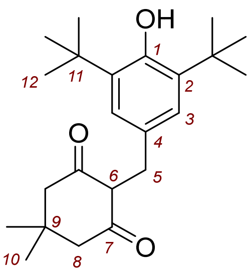

9^{Keto}

+

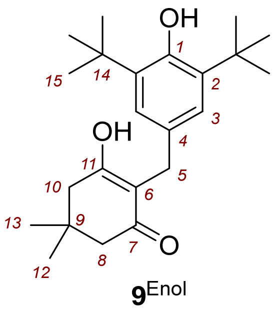

9^{Enol}

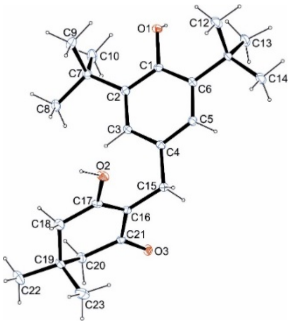

9 (CG538_1F2)

BHT (62.0 mg, 0.28 mmol) was dissolved in *n*-pentane (2 mL) and Ag_2O (326 mg, 1.41 mmol) was added. The mixture was stirred for 20 min at room temperature under the exclusion of light. Solids were removed by filtration (0.2 µm PTFE filter) and a DMSO solution of **2c** (55.2 mg, 0.31 mmol in 10 mL) was added. The mixture was stirred for 1 h at room temperature under nitrogen atmosphere. Then, the reaction was quenched with aq. sat. NH_4Cl solution (5 mL), diluted with water (5 mL) and extracted with diethyl ether (4 × 10 mL). The combined organic phases were washed with water (4 × 10 mL) and dried over $MgSO_4$. The solvent was removed under reduced pressure yielding a residue that was purified by preparative TLC (silica gel, *n*-pentane:EtOAc 95:5→9:1→6:4) to give **9** as a white solid (77.0 mg, 76%); m.p. 202 °C.

According to the integrals in the ^{1}H NMR spectrum, **9** was analyzed as a 46/54 mixture of keto and enol tautomers in $CDCl_3$.

^{1}H NMR (600 MHz, $CDCl_3$): δ = 7.04 (s, 2 H, 3-H^{Keto}), 7.00 (s, 2 H, 3-H^{Enol}), 5.81 (s, 1 H, 11-OH^{Enol}), 5.10 (s, 1 H, 1-OH^{Enol}), 5.07 (s, 1 H, 1-OH^{Keto}), 3.61 (s, 2 H, 5-H^{Enol}), 3.56 (t, *J* = 5.6 Hz, 1 H, 6-H^{Keto}), 3.10 (d, *J* = 5.7 Hz, 2 H, 5-H^{Keto}), 2.65 (d, *J* = 13 Hz, 2 H, 8-H^{Keto}), 2.42 (d, *J* = 13 Hz, 2 H, 8-H^{Keto}), 2.33 (s, 2 H, 10-H^{Enol}), 2.30 (s, 2 H, 8-H^{Enol}), 1.41 (s, 18 H, 12-H^{Keto}), 1.40 (s, 18 H, 15-H^{Enol}), 1.15 (s, 3 H, CH_3^{Enol}), 1.10 (s, 6 H, 10-H^{Keto}), 0.82 (s, 3 H, CH_3^{Enol}).

^{13}C{^{1}H} NMR (151 MHz, $CDCl_3$): δ = 204.1 (C_q, C-7^{Keto}), 198.0 (C_q, C-7^{Enol}), 169.9 (C_q, C-11^{Enol}), 152.7, 152.3, 136.6, 135.8, 130.8, 129.2, 126.2, 124.9, 114.0, 69.6 (CH, C-6^{Keto}), 54.7, 50.6, 42.8, 34.5, 34.4, 32.1, 31.1, 30.4 (CH_3, C-12^{Keto}), 30.4 (CH_3, C-15^{Enol}), 30.4 (CH_3), 28.5 (CH_3), 28.2 (CH_2, C-5^{Keto}), 27.4 (CH_2, C-5^{Enol}), 26.5 (CH_3).

HRMS (pos. ESI): *m/z* calcd for $C_{23}H_{34}NaO_3^+$ [M + Na^+]: 381.2400; found: 381.2407.

IR (neat, ATR): $\tilde{\nu}$ = 3397, 2957, 1562, 1423, 1390, 1316, 1260, 1196, 1149, 1036, 1019, 766 cm^{-1}.

2,6-Di-*tert*-butyl-4-(2-nitro-2-phenylethyl)phenol (10)

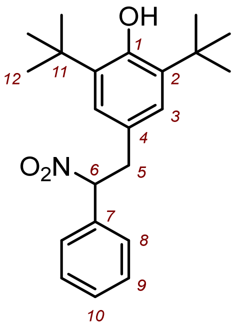

10 (CG654)

BHT (51.5 mg, 0.234 mmol) was dissolved in *n*-pentane (2 mL) and Ag_2O (271 mg, 1.17 mmol) was added. The mixture was stirred for 20 min under the exclusion of light at room temperature. Solids were removed by filtration (0.2 µm PTFE filter) and **2j** was added [generated in situ by mixing KO*t*Bu (31.5 mg, 0.281 mmol) and phenylnitromethane (49.7 mg, 0.362 mmol) in 5 mL DMSO]. The mixture was vigorously stirred for 1 h at room temperature under nitrogen atmosphere. The reaction was quenched with aq. sat. NH_4Cl solution (5 mL), diluted with water (5 mL) and extracted with diethyl ether (4 × 10 mL). The combined organic phases were washed with water (4 × 10 mL) and dried over $MgSO_4$. The solvent was removed under reduced pressure yielding a residue that was purified by preparative TLC (silica gel, *n*-pentane:EtOAc 97:3) to give **10** as an white solid (51.0 mg, 61%); m.p. 107 °C

R_f (*n*-pentane/EtOAc 97:3, silica, UV) = 0.50.

^{1}H NMR (400 MHz, $CDCl_3$): δ = 7.50–7.47 (m, 2 H, 8-H), 7.42–7.39 (m, 3 H, 9-H and 10-H), 6.86 (s, 2 H, 3-H), 5.61 (dd, J = 8.7, 6.3 Hz, 1 H, 6-H), 5.12 (s, 1 H, 1-OH), 3.69 (dd, J = 14.3, 8.7 Hz, 1 H, 5-H^a), 3.28 (dd, J = 14.3, 6.4 Hz, 1 H, 5-H^b), 1.38 (s, 18 H, 12-H).

^{13}C{^{1}H} NMR (101 MHz, $CDCl_3$): δ = 153.1 (C_q, C-1), 136.3 (C_q, C-2), 134.8 (C_q, C-7), 129.9 (CH, C-10), 129.1 (CH, C-9), 128.0 (CH, C-8), 126.1 (C_q, C-4), 125.7 (CH, C-3), 93.0 (CH, C-6), 40.5 (CH_2, C-5), 34.4 (C_q, C-11), 30.4 (CH_3, C-12).

HRMS (EI): *m/z* calcd for $C_{22}H_{29}NO_3^{\bullet+}$ [$M^{\bullet+}$]: 355.2142; found: 355.2142.

IR (neat, ATR): $\tilde{\nu}$ = 3624, 2956, 1549, 1433, 1367, 1231, 1211, 1142, 1117, 873, 790, 767, 723, 694 cm^{-1}.

Diethyl 2-(1-(3,5-di-*tert*-butyl-4-hydroxyphenyl)ethyl)malonate (11) was synthesized according to GP1 (aqueous workup after 30 min) from the *p*QM **1e** (15.0 mg, 0.065 mmol) and **2j** (13.4 mg, 0.068 mmol) and yielding a residue that was purified by preparative TLC (silica gel, *n*-pentane:EtOAc 97:3) to give **11** as a colorless oil (15.3 mg, 60%).

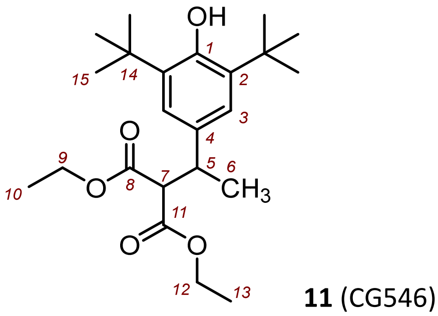

11 (CG546)

R_f (*n*-pentane/EtOAc 97:3, silica, UV) = 0.15.

^{1}H NMR (600 MHz, $CDCl_3$): δ = 6.99 (s, 2 H, 3-H), 5.07 (s, 1 H, 1-OH), 4.25–4.21 (m, 2 H, 9-H or 12-H), 3.90–3.85 (m, 2 H, 9-H or 12-H), 3.53 (d, J = 10.7 Hz, 1 H, 7-H), 3.47–3.41 (m, 1 H, 5-H), 1.41 (s, 18 H, 15-H), 1.31 (d, J = 6.7 Hz, 3 H, 6-H), 1.29 (t, J = 7.2 Hz, 3 H, 10-H or 13-H), 0.91 (t, J = 7.1 Hz, 3 H, 10-H or 13-H).

^{13}C{^{1}H} NMR (151 MHz, $CDCl_3$): δ = 168.8 (C_q, C-8 or C-11), 168.4 (C_q, C-8 or C-11), 152.6 (C_q, C-1), 135.7 (C_q, C-2), 133.5 (C_q, C-4), 124.1 (CH, C-3), 61.5 (CH_2, C-9 or C-12), 61.1 (CH_2, C-9 or C-12), 60.0 (CH, C-7), 40.5 (CH, C-5), 34.5 (C_q, C-14), 30.4 (CH_3, C-15), 20.5 (CH_3, C-6), 14.3 (CH_3, C-10 or C-13), 13.9 (CH_3, C-10 or C-13).

HRMS (EI): *m/z* calcd for $C_{23}H_{36}O_5^{\bullet+}$ [$M^{\bullet+}$]: 392.2557; found: 392.2556.

IR (neat, ATR): $\tilde{\nu}$ = 3643, 2958, 1753, 1728, 1435, 1367, 1234, 1176, 1151, 1120, 1029, 880, 770 cm^{-1}.

2-(3,5-Di-tert-butyl-4-hydroxyphenyl)-2-(2,2-dimethyl-4,6-dioxo-1,3-dioxan-5-yl)acetonitrile (12)

The *p*QM **1c** (15.2 mg, 0.062 mmol) was dissolved in *n*-pentane (2 mL) under nitrogen atmosphere and mixed with a DMSO solution of **2a** (14.8 mg, 0.081 mmol in 2 mL). The mixture was vigorously stirred for 20 min at room temperature under nitrogen atmosphere. Then, the reaction was quenched with 2 M hydrochloric acid (5 mL), diluted with water (5 mL) and extracted with diethyl ether (4 × 10 mL). The combined organic phases were washed with water (4 × 10 mL) and dried over $MgSO_4$. The solvent was removed under reduced pressure yielding a residue that was crystallized from *n*-pentane/CH_2Cl_2 to give **12** as colorless crystals (19.0 mg, 79%); m.p. >130 °C (dec.).

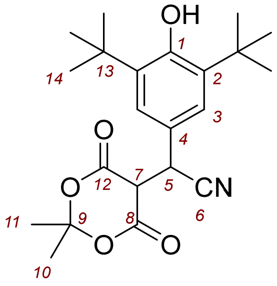

12 (CG651_1)

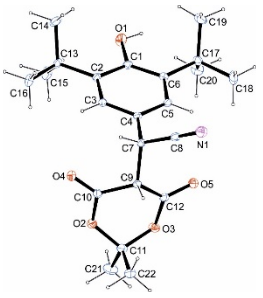

^{1}H NMR (400 MHz, CD_3CN): δ = 7.32 (s, 2 H, 3-H), 5.60 (s, 1 H, 1-H), 4.95 (d, J = 2.8 Hz, 1 H, 5-H), 4.56 (d, J = 3.0 Hz, 1 H, 7-H), 1.78 (s, 3 H), 1.58 (s, 3 H), 1.40 ppm (s, 18 H, 14-H).

^{13}C{^{1}H} NMR (101 MHz, CD_3CN): δ = 164.2 (C_q, C-8 or C-12), 163.8 (C_q, C-8 or C-12), 154.9 (C_q, C-1), 138.4 (C_q, C-2), 126.7 (CH, C-3), 124.0 (C_q, C-4), 119.7 (C_q, C-6), 107.3 (C_q, C-9), 51.4 (CH, C-7), 35.3 (C_q, C-13), 34.7 (CH, C-5), 30.4 (CH_3, C-14), 28.6 (CH_3, C-10 or C11), 26.9 ppm (CH_3, C-10 or C-11).

HRMS (pos. ESI): *m/z* calcd for $C_{22}H_{29}NNaO_5^+$ [M + Na^+] 410.1938; found: 410.1932.

IR (neat, ATR): $\tilde{\nu}$ = 3633, 2958, 2865, 2251, 1785, 1748, 1436, 1395, 1387, 1334, 1323, 1292, 1270, 1237, 1200, 1159, 1080, 1059, 1019, 994, 923, 900, 877, 860, 774, 735 cm^{-1}.

2-(3,5-Di-*tert*-butyl-4-hydroxyphenyl)ethane-1,1,2-tricarbonitrile (13)

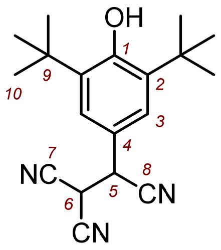

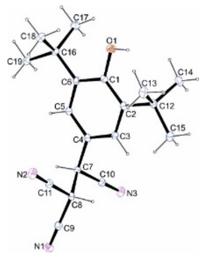

13 (CG553)

The *p*QM **1c** (15.0 mg, 0.062 mmol) was dissolved in *n*-pentane (2 mL) under nitrogen atmosphere and mixed with a DMSO solution of **2h** (7.1 mg, 0.068 mmol in 2 mL). The mixture was vigorously stirred for 30 min at room temperature. The reaction was quenched with aq. sat. NH_4Cl solution (2 mL), diluted with water (5 mL) and extracted with diethyl ether (3 × 10 mL). The combined organic phases were washed with water (3 × 10 mL) and dried over $MgSO_4$. The solvent was removed under reduced pressure yielding a residue that was purified by preparative TLC (silica gel, *n*-pentane:EtOAc 9:1) to give product 1**3** as an off-white solid (16.5 mg, 86%); m.p. 188 °C.

R_f (*n*-pentane/EtOAc 9:1, silica, UV) = 0.50.

^{1}H NMR (400 MHz, $CDCl_3$): δ = 7.26 (s, 2 H, 3-H, superimposed by residual $CHCl_3$), 5.52 (s, 1 H, 1-OH), 4.38 (d, J = 5.6 Hz, 1 H, 5-H), 4.17 (d, J = 5.6 Hz, 1 H, 6-H), 1.46 (s, 18 H, 10-H).

^{13}C{^{1}H} NMR (101 MHz, $CDCl_3$): δ = 156.0 (C_q, C-1), 137.7 (C_q, C-2), 125.1 (CH, C-3), 118.9 (C_q, C-4), 115.4 (C_q, C-8), 109.7 (C_q, C-7), 39.3 (CH, C-5), 34.7 (C_q, C-9), 30.1$_8$ (CH, C-6), 30.1$_5$ (CH_3, C-10).

HRMS (EI): m/z calcd for $C_{19}H_{23}N_3O^{\bullet+}$ [$M^{\bullet+}$]: 309.1836; found: 309.1836.

IR (neat, ATR): $\tilde{\nu}$ = 3628, 2959, 2258, 1435, 1240, 1157, 1123, 909, 884, 733 cm^{-1}.

1,1-Diethyl 2-methyl 2-(3,5-di-*tert*-butyl-4-hydroxyphenyl)ethane-1,1,2-tricarboxylate (14) was synthesized according to GP1 (aqueous workup after 30 min) from **1d** (7.3 mg, 0.026 mmol) and **2j** (5.5 mg, 0.028 mmol) and yielding **14** as a white solid (11.1 mg, 98%); m.p. 77 °C.

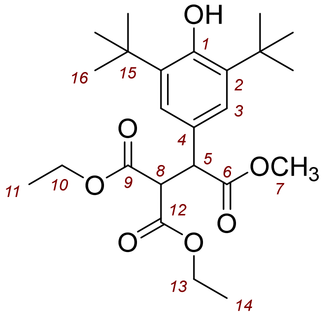

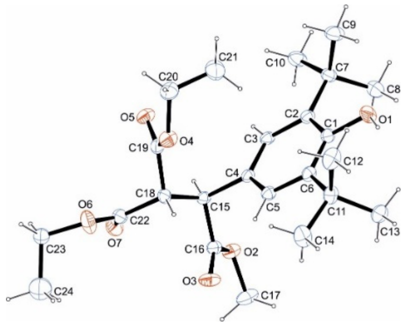

14 (CG524_1)

^{1}H NMR (600 MHz, $CDCl_3$): δ = 7.04 (s, 2 H, 3-H), 5.18 (s, 1 H, 1-OH), 4.24–4.15 (m, 4 H, 5-H, 8-H and 13-H), 3.93–3.84 (m, 2 H, 10-H), 3.68 (s, 3 H, 7-H), 1.40 (s, 18 H, 16-H), 1.27 (t, J = 7.2 Hz, 3 H, 11-H or 14-H), 0.89 (t, J = 7.1 Hz, 3 H, 11-H or 14-H).

^{13}C{^{1}H} NMR (151 MHz, $CDCl_3$): δ = 173.0 (C_q, C-6), 168.2 (C_q, C-9 or C-12), 167.5 (C_q, C-9 or C-12), 153.7 (C_q, C-1), 136.1 (C_q, C-2), 125.3 (C_q, C-4), 125.2 (CH, C-3), 62.0 (CH_2, C-10 or C-13), 61.4 (CH_2, C-10 or C-

13), 55.9 (CH, C-8), 52.6 (CH_3, C-7), 50.6 (CH, C-5), 34.5 (C_q, C-15), 30.3 (CH_3, C-16), 14.1 (CH_3, C-11 or C-14), 13.9 (CH_3, C-11 or C-14).

HRMS (pos. ESI): *m/z* calcd for $C_{24}H_{36}NaO_7^+$ [M + Na^+] 459.2353; found: 459.2354.

IR (neat, ATR): $\tilde{\nu}$ = 3602, 2957, 1731, 1435, 1368, 1299, 1236, 1159, 1121, 1023 cm^{-1}.

3-(3,5-Di-*tert*-butyl-4-hydroxybenzylidene)pentane-2,4-dione (15)

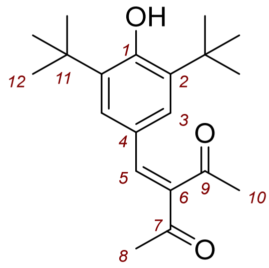

15 (CG652)

The *p*QM **1f** (24.0 mg, 0.097 mmol) was dissolved in *n*-pentane (2 mL) under nitrogen atmosphere and mixed with a DMSO solution of **2e** (15.2 mg, 0.11 mmol in 2 mL). The mixture was vigorously stirred under nitrogen atmosphere for 20 min at room temperature. The reaction was quenched with aq. sat. NH_4Cl solution (5 mL), diluted with water (5 mL) and extracted with diethyl ether (4 × 10 mL). The combined organic phases were washed with water (4 × 10 mL) and dried over $MgSO_4$. The solvent was removed under reduced pressure yielding a residue that was purified by preparative TLC (silica gel, *n*-pentane:EtOAc 85:15) to give **15** as a white solid (25.0 mg, 81%); m.p. 124 °C.

R_f (*n*-pentane/EtOAc 85:15, silica, UV) = 0.60.

^{1}H NMR (600 MHz, $CDCl_3$): δ = 7.41 (s, 1 H, 5-H), 7.24 (s, 2 H, 3-H), 5.63 (s, 1 H, 1-OH), 2.41 (s, 3 H, 8-H or 10-H), 2.35 (s, 3 H, 8-H or 10-H), 1.42 (s, 18 H, 12-H).

^{13}C{^{1}H} NMR (151 MHz, $CDCl_3$): δ = 206.7 (C_q, C-7 or C-9), 196.8 (C_q, C-7 or C-9), 156.7 (C_q, C-1), 141.5 (CH, C-5), 139.9 (C_q, C-6), 136.6 (C_q, C-2), 128.0 (CH, C-3), 124.1 (C_q, C-4), 34.6 (C_q, C-11), 31.8 (CH_3, C-8 or C-10), 30.2 (CH_3, C-12), 26.5 (CH_3, C-8 or C-10).

HRMS (EI): *m/z* calcd for $C_{20}H_{28}O_3^{\bullet+}$ $[M]^{\bullet+}$ 316.2033; found: 316.2015.

IR (neat, ATR): $\tilde{\nu}$ = 3573, 2957, 1701, 1640, 1607, 1592, 1428, 1381, 1262, 1240, 1212, 1174, 1115, 918 cm^{-1}.

Diethyl 2-(3,5-di-*tert*-butyl-4-hydroxybenzylidene)malonate (16)

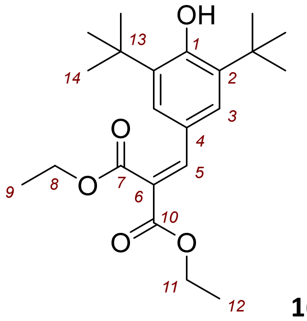

16 (CG547_1)

The *p*QM **1f** (22.8 mg, 0.092 mmol) was dissolved in *n*-pentane (2 mL) under nitrogen atmosphere and mixed with a DMSO solution of **2j** (20.0 mg, 0.10 mmol in 2 mL). The mixture was vigorously stirred for

15 h at room temperature under nitrogen atmosphere. The reaction was quenched with aq. sat. NH_4Cl solution (5 mL), diluted with water (5 mL) and extracted with diethyl ether (4 × 10 mL). The combined organic phases were washed with water (4 × 10 mL) and dried over $MgSO_4$. The solvent was removed under reduced pressure yielding a residue that was purified by preparative TLC (silica gel, *n*-pentane:EtOAc 97:3) to give **16** as a white solid (22.0 mg, 63%); m.p. 112 °C.

R_f (*n*-pentane/EtOAc 97:3, silica, UV) = 0.15.

^{1}H NMR (400 MHz, $CDCl_3$): δ = 7.65 (s, 1 H, 5-H), 7.34 (s, 2 H, 3-H), 5.57 (s, 1 H, 1-OH), 4.36 (q, *J* = 7.1 Hz, 2 H, 8-H or 11-H), 4.29 (q, *J* = 7.1 Hz, 2 H, 8-H or 11-H), 1.42 (s, 18 H, 14-H), 1.32 (t, *J* = 7.1 Hz, 6 H, 9-H and 12-H).

^{13}C{^{1}H} NMR (101 MHz, $CDCl_3$): δ = 167.7 (C_q, C-7 or C-10), 164.9 (C_q, C-7 or C-10), 156.6 (C_q, C-1), 143.2 (CH, C-5), 136.4 (C_q, C-2), 127.7 (CH, C-3), 124.3 (C_q, C-4), 122.7 (C_q, C-6), 61.7 (CH_2, C-8 or C-10), 61.4 (CH_2, C-8 or C-10), 34.5 (C_q, C-13), 30.3 (CH_3, C-14), 14.3 (CH_3, C-9 or C12), 14.2 (CH_3, C-9 or C12).

HRMS (EI): *m*/*z* calcd for $C_{22}H_{32}O_5^{\bullet+}$ $[M]^{\bullet+}$ 376.2244; found: 379.2244.

IR (neat, ATR): $\tilde{\nu}$ = 3594, 2952, 1722, 1688, 1622, 1430, 1384, 1259, 1207, 1141, 1067, 765 cm^{-1}.

4. Products of Reactions of *p*QMs with Further Classes of Nucleophiles

2-((3,5-Di-*tert*-butyl-4-hydroxyphenyl)(methoxy)methyl)-1,3-dimesityl-1*H*-imidazol-3-ium tetra-fluoroborate (18)

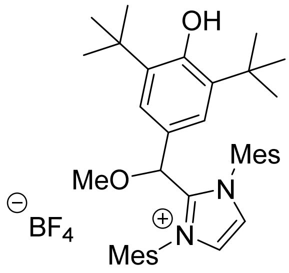

18 (CG688)

The *p*QM **1f** (18.0 mg, 0.073 mmol) and the *N*-heterocyclic carbene **17** (21.0 mg, 0.069 mmol) were dissolved in dry THF (5mL) under nitrogen atmosphere. The reaction mixture was stirred for 5 min at room temperature under nitrogen atmosphere. Then, the reaction was quenched with ethereal HBF_4 (10 µL, 11.1 mg, 0.069 mmol). The solvent was removed under reduced pressure. Subsequently, the crude material was dissolved in a minimum volume of DCM/Et_2O (ratio 1:4) and precipitated by addition of *n*-pentane to yield **18** as a white solid (36.0 mg, 81%); m.p. 199 °C.

^{1}H NMR (600 MHz, $CDCl_3$): δ = 7.64 (s, 2 H), 7.09 (s, 2 H), 6.91 (s, 2 H), 6.41 (s, 2 H), 5.26 (s, 1 H), 5.02 (s, 1 H), 3.13 (s, 3 H, OMe), 2.36 (s, 6 H, 2 × CH_3), 2.19 (s, 6 H, 2 × CH_3), 1.73 (s, 6 H, 2 × CH_3), 1.24 (s, 18 H, 2 × $C(CH_3)_3$).

^{13}C{^{1}H} NMR (151 MHz, $CDCl_3$): δ = 154.7 (C_q), 143.7 (C_q), 141.8 (C_q), 136.5 (C_q), 135.1 (C_q), 135.0 (C_q), 130.3 (C_q), 130.1 (CH), 130.0 (CH), 125.9 (CH), 123.1 (CH), 121.1 (C_q), 74.9 (CH), 58.5 (OCH_3), 34.2 (C_q), 29.8 (CH_3), 21.3 (CH_3), 17.8 (CH_3), 16.9 (CH_3).

HRMS (pos. ESI): *m/z* calcd for $C_{37}H_{49}N_2O_2^+$ [M – BF_4^-]: 553.3789; found: 553.3779.

IR (neat, ATR): $\tilde{\nu}$ = 3532, 2964, 1503, 1432, 1230, 1196, 1098, 1049, 1035, 983, 851, 782, 774 cm^{-1}.

Methyl 2-(1*H*-benzo[*d*]imidazol-1-yl)-2-(3,5-di-*tert*-butyl-4-hydroxyphenyl)acetate (20)

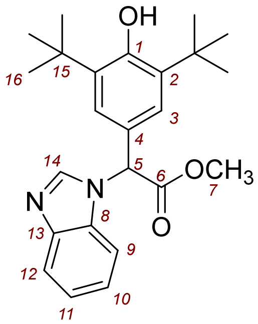

20 (CG690)

The *p*QM **1d** (20.0 mg, 0.072 mmol) was dissolved in *n*-pentane (2 mL) under nitrogen atmosphere and **19** [generated by mixing KO*t*Bu (8.5 mg, 0.076 mmol) with benzimidazole (10.3 mg, 0.087 mmol) in 2 mL DMSO] was added to the *p*QM solution. The reaction mixture was vigorously stirred for 5 min at room temperature under nitrogen atmosphere. The reaction was quenched with aq. sat. NH_4Cl solution (2 mL), diluted with water (5 mL) and extracted with diethyl ether (4 × 10 mL). The combined organic phases were washed with water (4 × 10 mL) and dried over $MgSO_4$. The solvent was removed

under reduced pressure yielding a residue that was purified by preparative TLC (silica gel, *n*-pentane:EtOAc:NEt_3 50:48:2) to give **20** as a colorless solid (13.0 mg, 46%); m.p. 182 °C.

R_f (*n*-pentane/EtOAc/NEt_3 50:48:2, silica, UV) = 0.55.

^{1}H NMR (600 MHz, $CDCl_3$): δ = 7.83–7.81 (m, 2 H, 14-H, 12-H), 7.37–7.35 (m, 1 H, 9-H), 7.33–7.29 (m, 2 H, 10-H, 11-H), 7.22 (s, 2 H, 3-H), 6.04 (s, 1 H, 5-H), 5.45 (s, 1 H, 1-OH), 3.82 (s, 3 H, 7-H), 1.42 (s, 18 H, 16-H).

^{13}C{^{1}H} NMR (151 MHz, $CDCl_3$): δ = 169.8 (C_q, C-6), 155.1 (C_q, C-1), 143.9 (C_q, C-13), 142.4 (CH, C-14), 137.2 (C_q, C-2), 134.0 (C_q, C-8), 125.4 (CH, C-3), 123.3 (CH, C-11), 123.2 (C_q, C-4), 122.7 (CH, C-10), 120.7 (CH, C-12), 109.5 (CH, C-9), 62.1 (CH, C-5), 53.2 (CH_3, C-7), 34.6 (C_q, C-15), 30.2 (CH_3, C-16).

HRMS (EI): *m/z* calcd for $C_{24}H_{30}N_2O_3^{\bullet+}$ [$M^{\bullet+}$]: 394.2251; found: 394.2253.

IR (neat, ATR): $\tilde{\nu}$ = 3625, 2955, 1748, 1484, 1457, 1434, 1281, 1200, 1173, 1009, 909, 766, 738 cm^{-1}.

Methyl 2-((3-chlorophenyl)thio)-2-(3,5-di-*tert*-butyl-4-hydroxyphenyl)acetate (22)

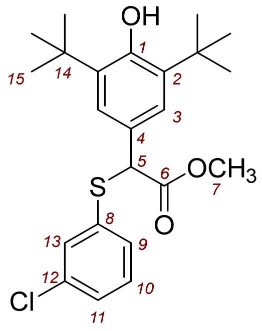

22 (CG693F2)

The *p*QM **1d** (20.0 mg, 0.072 mmol) was dissolved in DMSO (0.5 mL) under nitrogen atmosphere and **21** [generated by mixing KO*t*Bu (8.5 mg, 0.076 mmol) with 3-chlorothiophenol (18.8 mg, 0.130 mmol) in 0.5 ml DMSO] was added to the *p*QM solution. The reaction mixture was stirred for 10 min at room temperature under nitrogen atmosphere. The reaction was quenched with aq. sat. NH_4Cl solution (2 mL), diluted with water (5 mL) and extracted with diethyl ether (4 × 10 mL). The combined organic phases were washed with water (4 × 10 mL) and dried over $MgSO_4$. The solvent was removed under reduced pressure yielding a residue that was purified by preparative TLC (silica gel, *n*-pentane:EtOAc 97:3) to give the thioether **22** as a colorless oil (16.0 mg, 53%).

R_f (*n*-pentane/EtOAc 97:3, silica, UV) = 0.65.

^{1}H NMR (600 MHz, $CDCl_3$): δ = 7.29–7.29 (m, 1 H, 10-H), 7.24–7.17 (m, 3 H, 9-H, 11-H and 13-H), 7.16–7.15 (m, 2 H, 3-H), 5.26 (s, 1 H, 1-OH), 4.87 (s, 1 H, 5-H), 3.70 (s, 3 H, 7-H), 1.41 (s, 18 H, 15-H).

^{13}C{^{1}H} NMR (151 MHz, $CDCl_3$): δ = 171.2 (C_q, C-6), 154.1 (C_q, C-1), 136.3 (C_q, C-2), 136.1 (C_q, C-8), 134.5 (C_q, C-12), 132.4 (CH, C-10), 130.8 (CH), 130.0 (CH), 128.0 (CH), 125.5 (CH, C-3), 125.4 (C_q, C-4), 56.0 (CH, C-5), 52.8 (CH_3, C-7), 34.5 (C_q, C-14), 30.3 (CH_3, C-15).

HRMS (EI): *m/z* calcd for $C_{23}H_{29}ClO_3S^{\bullet+}$ [$M^{\bullet+}$]: 420.1520; found: 420.1511.

IR (neat, ATR): $\tilde{\nu}$ = 3634, 2955, 1737, 1576, 1564, 1461, 1433, 1238, 1210, 1149, 1121, 779, 681 cm^{-1}.

Methyl 2-(3,5-di-*tert*-butyl-4-hydroxyphenyl)acetate (24)

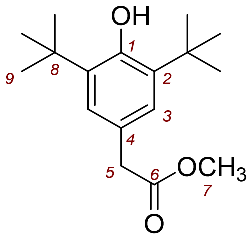

24 (CG699)

The *p*QM **1d** (25.0 mg, 0.090 mmol) was dissolved in methanol (2 mL) under nitrogen atmosphere and sodium cyanoborohydride **23** (12.5 mg, 0.20 mmol) was added portionwise over 10 min at room temperature. The mixture was stirred for 2 h at room temperature. The reaction was quenched by addition of aq. sat. ammonium chloride solution (2 mL), diluted with water (5 mL) and extracted with diethyl ether (3 × 10 mL). The combined organic phases were washed with water (3 × 10 mL) and dried over $MgSO_4$. The solvent was removed under reduced pressure yielding a residue that was purified by preparative TLC (silica gel, *n*-pentane:EtOAc 98:2) to give product **24** as a white solid (20.0 mg, 80%); m.p. 85 °C.

R_f (*n*-pentane/EtOAc 98:2, silica, UV) = 0.35.

^{1}H NMR (600 MHz, $CDCl_3$): δ = 7.08 (s, 2 H, 3-H), 5.15 (s, 1 H, 1-OH), 3.70 (s, 3 H, 7-H), 3.54 (s, 2 H, 5-H), 1.44 (s, 18 H, 9-H).

^{13}C{^{1}H} NMR (151 MHz, $CDCl_3$): δ = 172.9 (C_q, C-6), 153.0 (C_q, C-1), 136.1 (C_q, C-2), 126.0 (CH, C-3), 124.7 (C_q, C-4), 52.1 (CH_3, C-7), 41.1 (CH_2, C-5), 34.4 (C_q, C-8), 30.4 (CH_3, C-9).

HRMS (EI): *m/z* calcd for $C_{17}H_{26}O_3^{\bullet+}$ [$M^{\bullet+}$]: 278.1876; found: 278.1874.

IR (neat, ATR): $\tilde{\nu}$ = 3630, 2954, 1735, 1434, 1361, 1316, 1234, 1156, 1121, 1016 cm^{-1}.

2-(3,5-Di-*tert*-butyl-4-hydroxybenzyl)cyclohexan-1-one (26)

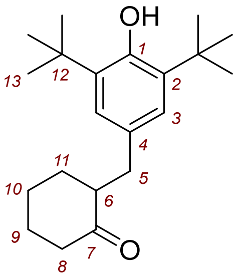

26 (CG695_DMSO)

BHT (33.0 mg, 0.150 mmol) was dissolved in *n*-pentane (2 mL) and Ag_2O (174 mg, 0.750 mmol) was added. The mixture was stirred for 20 min at room temperature under the exclusion of light. Solids were removed by filtration (0.2 µm PTFE filter) and a dichloromethane solution of enamine **25** (22.7 mg, 0.150 mmol in 2 mL) was added. The mixture was stirred for 15 min at room temperature under nitrogen atmosphere. The reaction was quenched with hydrochloric acid (2 M, 2 mL), diluted with water (5 mL) and extracted with dichloromethane (3 × 10 mL). The combined organic phases were washed with aq. sat. $NaHCO_3$ solution (10 mL) and water (10 mL) and dried over $MgSO_4$. The solvent was removed under reduced pressure yielding a residue that was purified by preparative TLC (silica gel, *n*-pentane:EtOAc 97:3) to give **26** as a light yellow oil (33.0 mg, 69%).

R_f (*n*-pentane/EtOAc 97:3, silica, UV) = 0.50.

^{1}H NMR (400 MHz, DMSO-d_6): δ = 6.86 (s, 2 H, 3-H), 6.67 (s, 1 H, 1-OH), 2.96 (dd, J = 13.9, 5.1 Hz, 1 H, 5-H^a), 2.57–2.53 (m, 1 H, 6-H), 2.45–2.33 (m, 1 H), 2.27–2.23 (m, 1 H), 2.23–2.17 (m, 1 H, 5-H^b), 1.98–1.89 (m, 2 H), 1.78–1.69 (m, 1 H), 1.63–1.51 (m, 2 H), 1.35 (s, 18 H, 13-H), 1.30–1.19 (m, 1 H).

^{13}C{^{1}H} NMR (101 MHz, DMSO-d_6): δ = 211.9 (C_q, C-7), 151.8 (C_q, C-1), 138.9 (C_q, C-2), 131.0 (C_q, C-4), 125.0 (CH, C-3), 51.6 (CH, C-6), 41.4 (CH_2), 35.0 (CH_2, C-5), 34.4 (C_q, C-12), 32.9 (CH_2), 30.4 (CH_3, C-13), 27.5(CH_2), 24.2 (CH_2).

HRMS (EI): m/z calcd for $C_{21}H_{32}O_2^{\bullet+}$ [M$^{\bullet+}$]: 316.2397; found: 316.2394.

IR (neat, ATR): $\tilde{\nu}$ = 3642, 2950, 2863, 1706, 1434, 1360, 1313, 1233, 1153, 1122, 883, 768 cm^{-1}.

4-(1-(Benzylamino)ethyl)-2,6-di-*tert*-butylphenol (28)

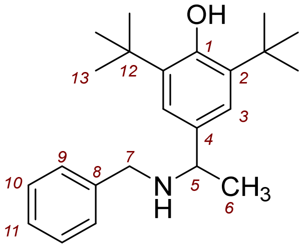

28 (CG698)

The *p*QM **1e** (29.0 mg, 0.12 mmol) was dissolved in DMSO (4 mL) under nitrogen atmosphere and a DMSO solution of benzylamine **27** (17.4 mg, 0.16 mmol in 1 mL) was added. The reaction mixture was stirred for 2 h at room temperature. The reaction was quenched by addition of water (10 mL) and extracted with diethyl ether (4 × 10 mL). The combined organic phases were washed with water (4 × 10 mL) and dried over $MgSO_4$. The solvent was removed under reduced pressure yielding a residue that was purified by preparative TLC (silica gel, *n*-pentane:EtOAc:NEt_3 96:3:1) to give product **28** as a colorless oil (25.0 mg, 61%).

R_f (*n*-pentane/EtOAc/NEt_3 96:3:1, silica, UV) = 0.40.

^{1}H NMR (400 MHz, CD_2Cl_2): δ = 7.31–7.30 (m, 4 H, 9-H and 10-H), 7.26–7.20 (m, 1 H, 11-H), 7.15 (s, 2 H, 3-H), 5.13 (s, 1 H, 1-OH), 3.72 (q, J = 6.6 Hz, 1 H, 5-H), 3.65 (d, J = 13.1 Hz, 1 H, 7-H^a), 3.60 (d, J = 13.1 Hz, 1 H, 7-H^b), 1.45 (s, 18 H, 13-H), 1.32 (d, J = 6.6 Hz, 3 H, 6-H).

^{13}C{^{1}H} NMR (101 MHz, CD_2Cl_2): δ = 152.9 (C_q, C-1), 141.7 (C_q, C-8), 136.7 (C_q, C-4), 136.2 (C_q, C-2), 128.61 (CH, C-9), 128.58 (CH, C-10), 127.0 (CH, C-11), 123.6 (CH, C-3), 58.1 (CH, C-5), 52.1 (CH_2, C-7), 34.7 (C_q, C-12), 30.6 (CH_3, C-13), 24.8 (CH_3, C-6).

HRMS (pos. ESI): m/z calcd for $C_{23}H_{34}NO^+$ [M + H$^+$]: 340.2635; found: 340.2634.

5. UV/Vis Spectra and Molar Absorption Coefficients of *p*QMs

A solution of the quinone methide (3 mg *p*QM per mL DMSO) was added stepwise to a known volume of DMSO. The absorbances *A* of the *p*QM solutions were detected by using a *J&M* TIDAS diode array spectrophotometer (connected to a Hellma quartz probe with a path length d = 0.5 cm).

Molar absorption coefficients ε (M^{-1} cm^{-1}) were determined from the slopes of linear correlations of absorbance with *p*QM concentrations by assuming the validity of the Beer-Lambert law [Equation (S1)].

$$\lg (I_0/I) = A = \varepsilon\, d\, c \qquad \text{(S1)}$$

Solutions of *p*QM **1a** in DMSO: Concentration-dependent absorbance *A* at λ_{max}.

V (L)	[**1a**] (M)	*A* (292 nm)
0.02400	0	0.000
0.02405	2.95×10^{-5}	0.326
0.02407	4.13×10^{-5}	0.454
0.02409	5.30×10^{-5}	0.600
0.02411	6.48×10^{-5}	0.722
0.02413	7.65×10^{-5}	0.866
0.02415	8.82×10^{-5}	1.000
0.02417	9.99×10^{-5}	1.130

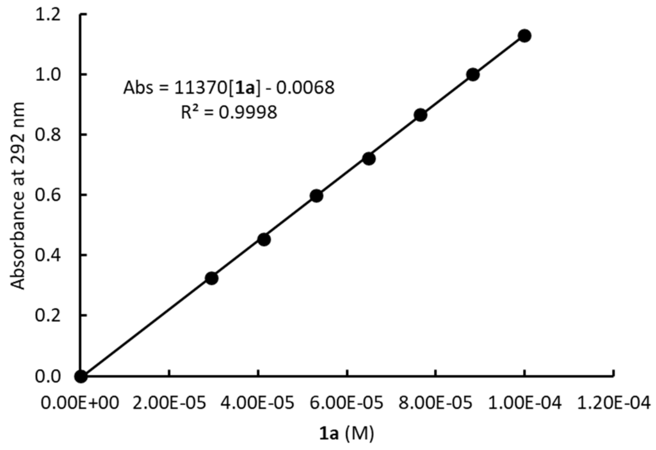

Concentration-dependent absorbance of *p*QM **1a** used to determine ε (at λ_{max} = 292 nm, d = 0.5 cm).

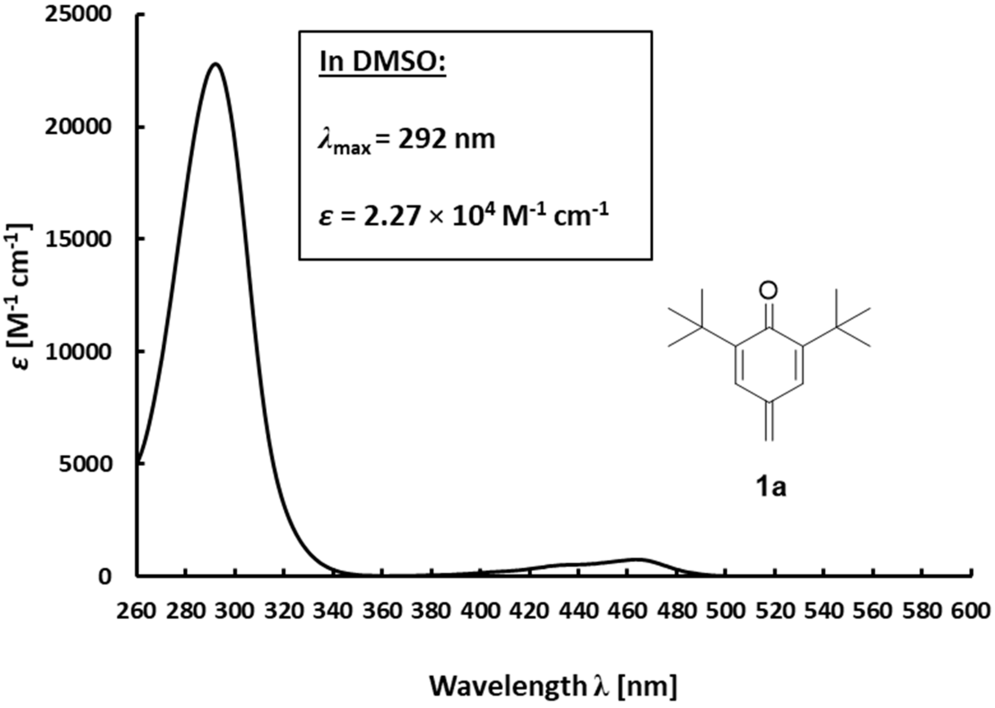

Solutions of *p*QM **1c** in DMSO: Concentration-dependent absorbance *A* at λ_{max}.

V (L)	[**1c**] (M)	*A* (318 nm)
0.02400	0	0.000
0.02402	1.15×10^{-5}	0.198
0.02404	2.29×10^{-5}	0.388
0.02406	3.43×10^{-5}	0.585
0.02408	4.57×10^{-5}	0.720
0.02410	5.71×10^{-5}	0.915
0.02412	6.85×10^{-5}	1.095
0.02414	7.98×10^{-5}	1.240

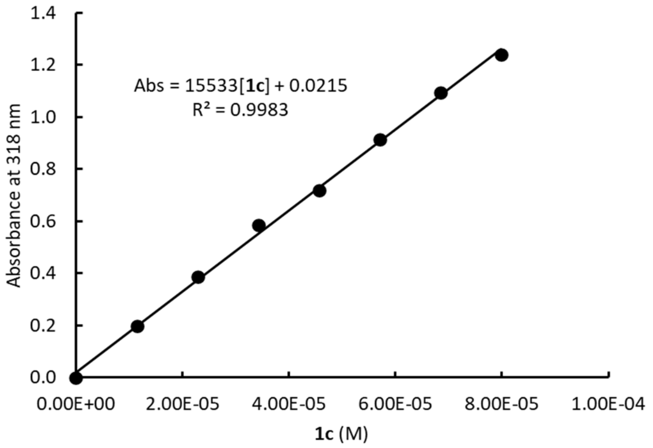

Concentration-dependent absorbance of *p*QM **1c** used to determine ε (at λ_{max} = 318 nm, *d* = 0.5 cm).

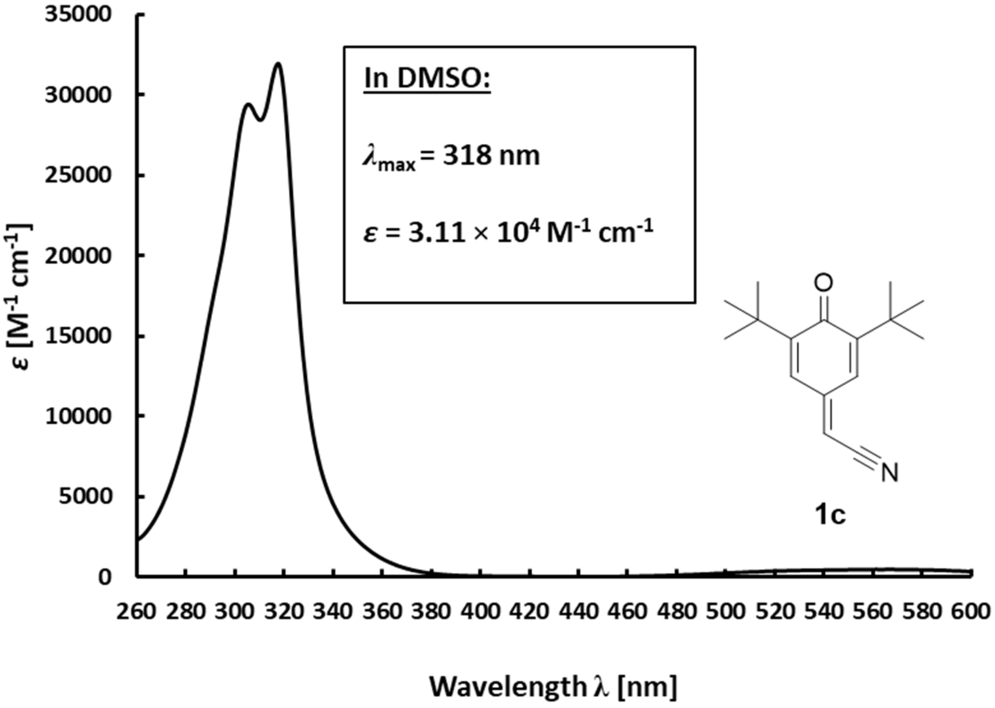

Solutions of *p*QM **1d** in DMSO: Concentration-dependent absorbance *A* at λ_{max}.

V (L)	[**1d**] (M)	*A* (309 nm)
0.02400	0	0.000
0.02405	2.26×10^{-5}	0.379
0.02407	3.16×10^{-5}	0.509
0.02409	4.06×10^{-5}	0.639
0.02411	4.95×10^{-5}	0.807
0.02413	5.85×10^{-5}	0.950
0.02415	6.74×10^{-5}	1.093

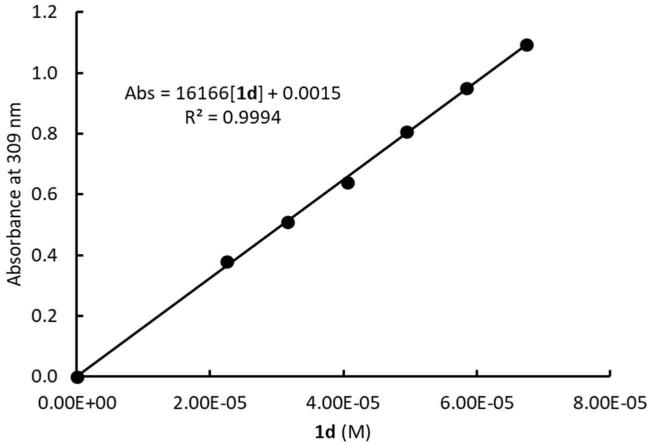

Concentration-dependent absorbance of *p*QM **1d** used to determine ε (at λ_{max} = 309 nm, *d* = 0.5 cm).

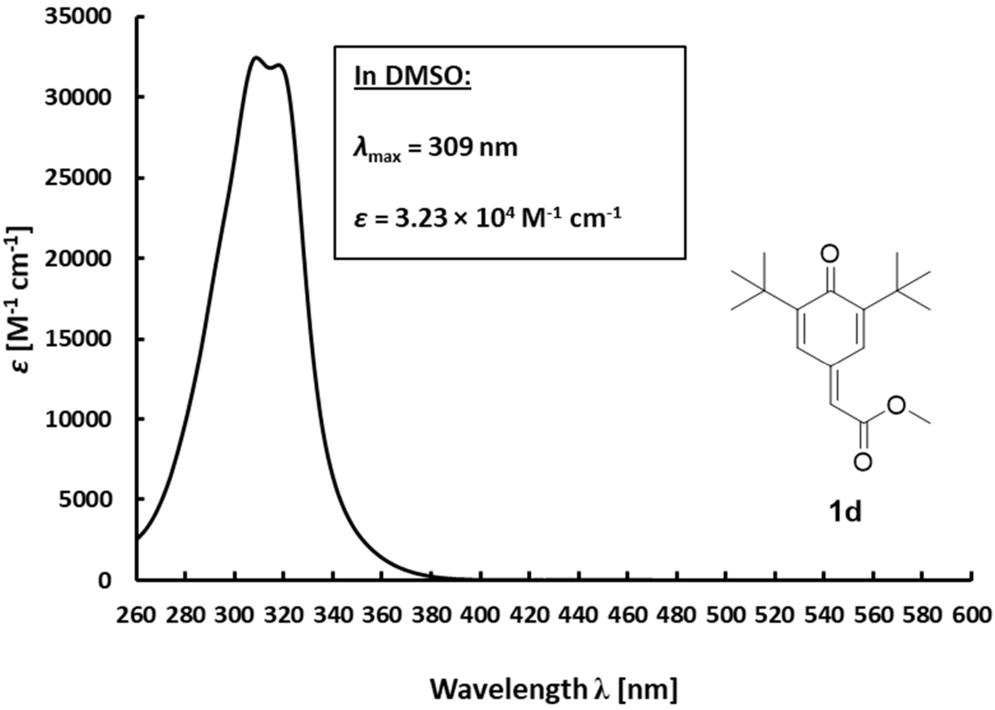

Solutions of *p*QM **1e** in DMSO: Concentration-dependent absorbance *A* at λ_{max}.

V (L)	[**1e**] (M)	*A* (309 nm)
0.02400	0	0.000
0.02405	3.04×10^{-5}	0.359
0.02407	4.26×10^{-5}	0.473
0.02409	5.47×10^{-5}	0.632
0.02411	6.68×10^{-5}	0.774
0.02413	7.88×10^{-5}	0.933
0.02415	9.09×10^{-5}	1.092

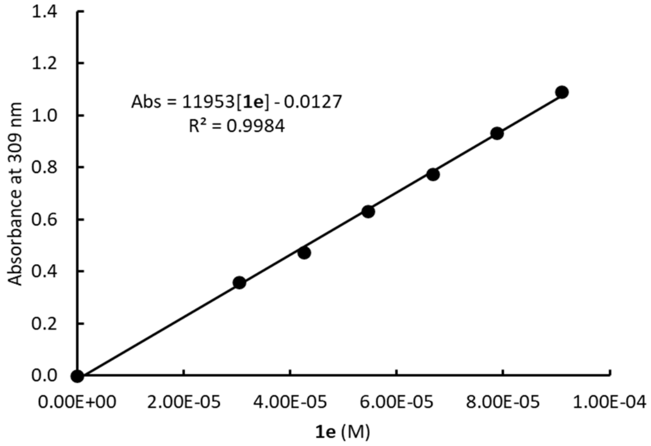

Concentration-dependent absorbance of *p*QM **1e** used to determine ε (at λ_{max} = 309 nm, *d* = 0.5 cm).

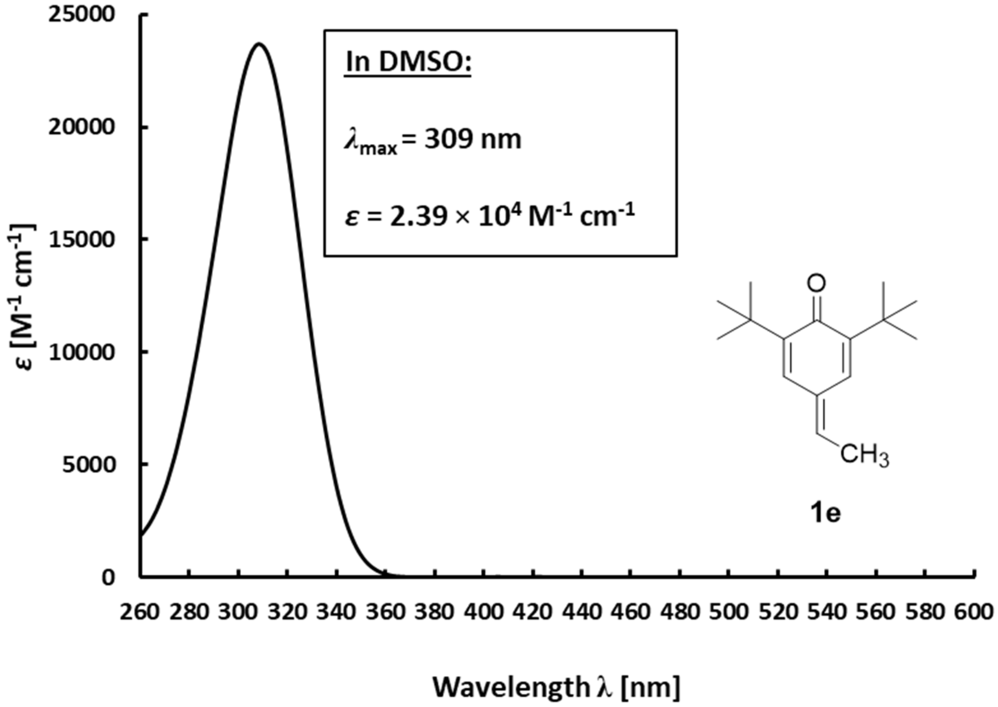

Solutions of *p*QM **1f** in DMSO: Concentration-dependent absorbance *A* at λ_{max}.

V (L)	[**1f**] (M)	*A* (345 nm)
0.02400	0	0.000
0.02402	1.11×10^{-5}	0.165
0.02404	2.21×10^{-5}	0.314
0.02406	3.31×10^{-5}	0.467
0.02408	4.41×10^{-5}	0.622
0.02410	5.51×10^{-5}	0.799
0.02412	6.61×10^{-5}	0.939
0.02414	7.71×10^{-5}	1.100
0.02416	8.80×10^{-5}	1.240

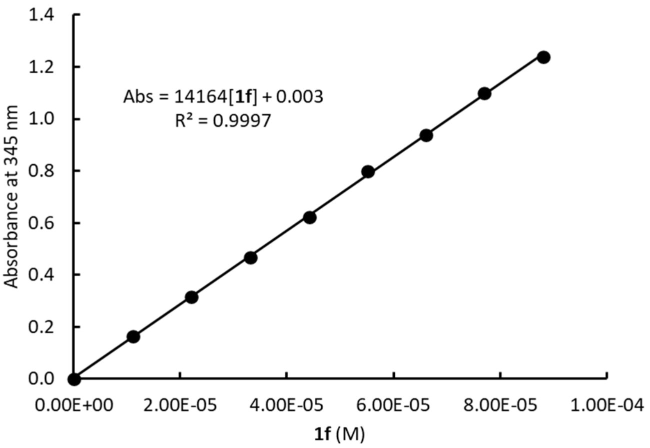

Concentration-dependent absorbance of *p*QM **1f** used to determine ε (at λ_{max} = 345 nm, *d* = 0.5 cm).

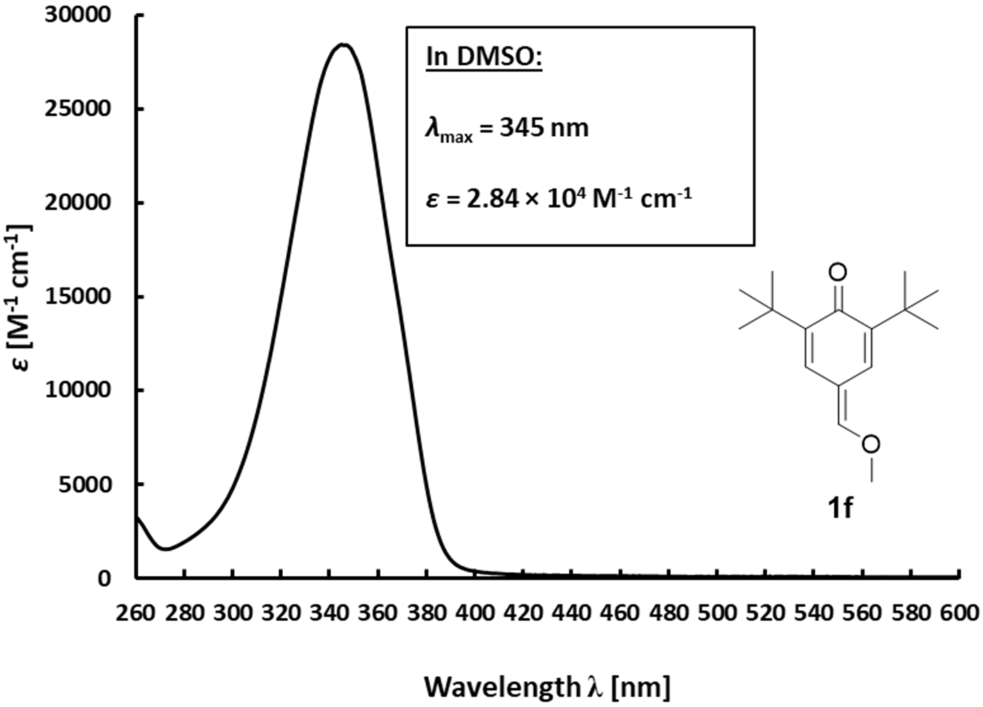

Solutions of *p*QM **1g** in DMSO: Concentration-dependent absorbance *A* at λ_{max}.

V (L)	[**1g**] (M)	*A* (359 nm)
0.02400	0	0.000
0.02402	9.33×10^{-6}	0.135
0.02404	1.86×10^{-5}	0.303
0.02406	2.79×10^{-5}	0.437
0.02408	3.72×10^{-5}	0.586
0.02410	4.65×10^{-5}	0.730
0.02412	5.58×10^{-5}	0.880
0.02414	6.50×10^{-5}	1.033

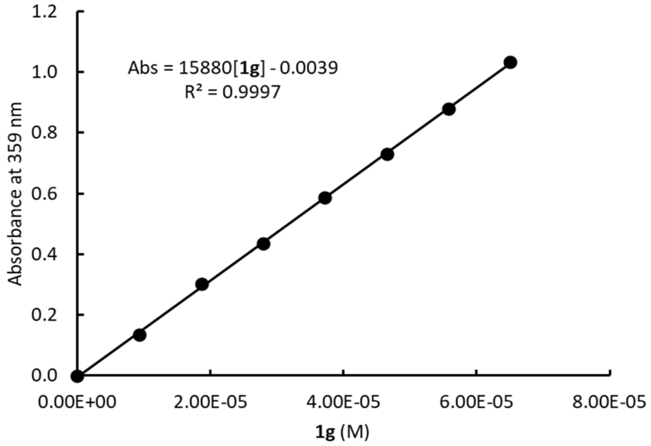

Concentration-dependent absorbance of *p*QM **1g** used to determine ε (at λ_{max} = 359 nm, *d* = 0.5 cm).

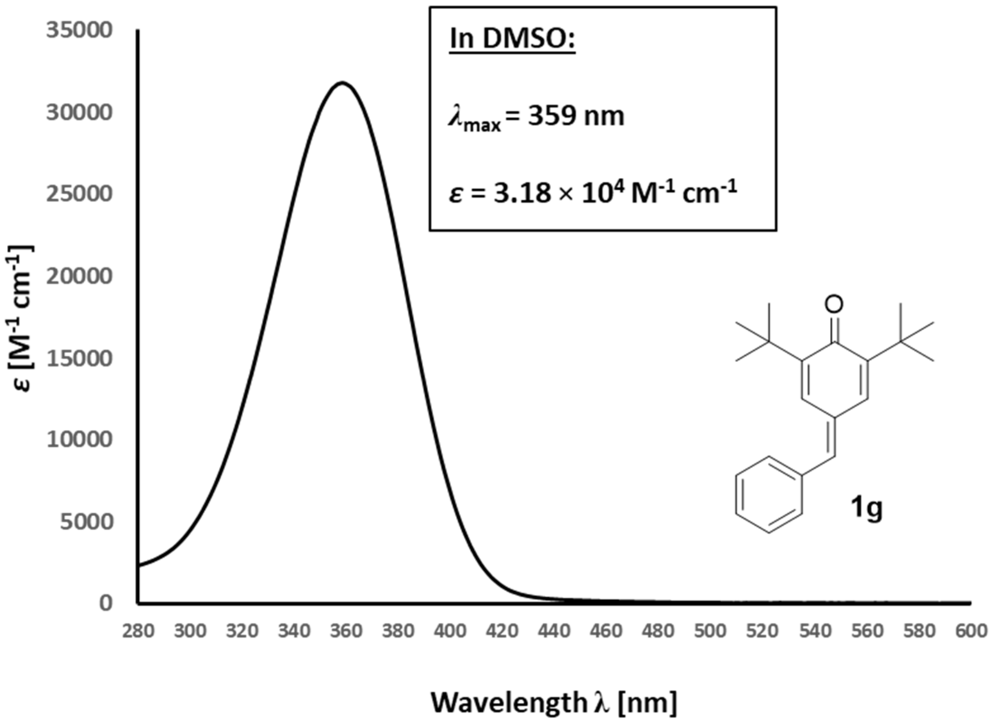

6. Cyclic Voltammetry: Reduction Potentials of *p*QMs

Cyclic voltammetry (CH Instruments 630E electrochemical analyzer, 2 mm diameter platinum working electrode, platinum wire counter electrode and Ag wire pseudo-reference electrode, scan rate 0.1 V/s) was performed in a 0.1 M tetra-n-butyl ammonium perchlorate solution of deaerated acetonitrile with the *p*QM **1** ($c \approx 1 \times 10^{-3}$ M) and ferrocene ($c = 7.5 \times 10^{-4}$ M, as an internal standard). The $E_{1/2}$(fc$^+$/fc in MeCN) = +0.382 V was used to calibrate E_p^{red} (**1** in MeCN) vs SCE.[71] Only peak potentials E_p^{red} could be determined due to the non-reversibility of the *p*QM reduction.

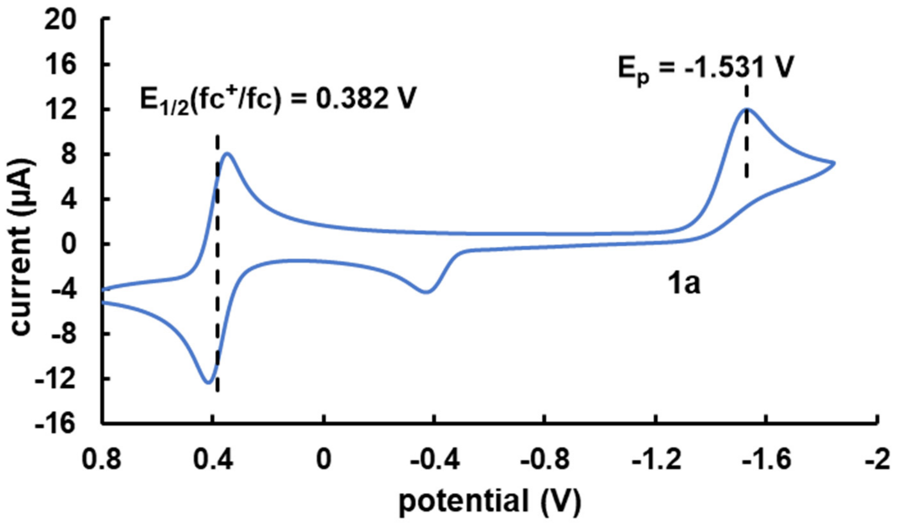

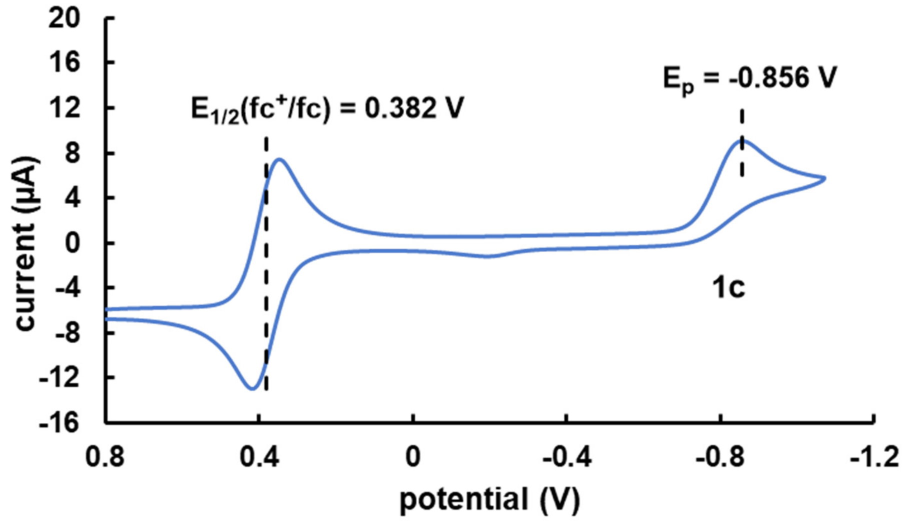

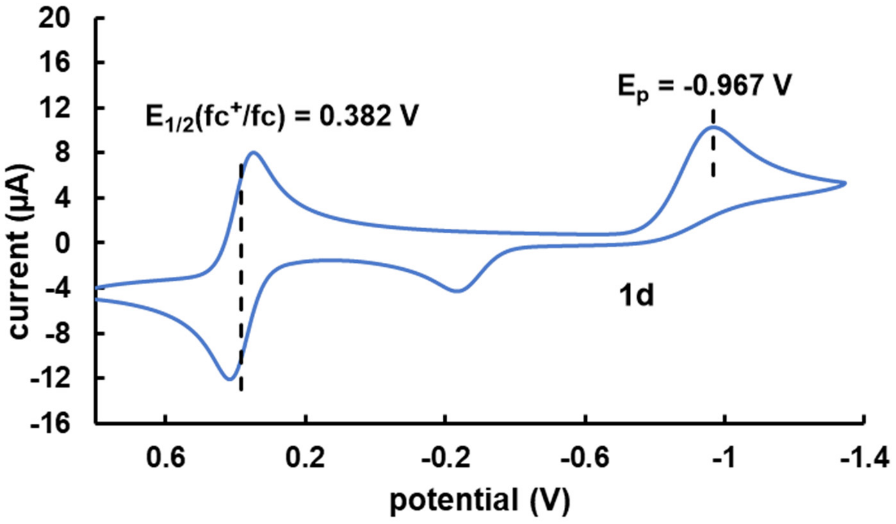

E1/2(fc+/fc) = 0.382 V
Ep = -0.967 V
1d
current (μA)
potential (V)

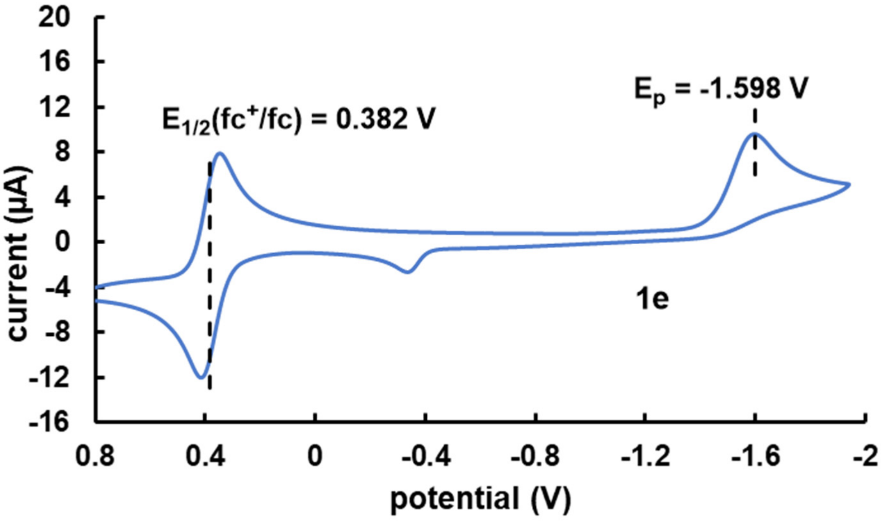

E1/2(fc+/fc) = 0.382 V
Ep = -1.598 V
1e
current (μA)
potential (V)

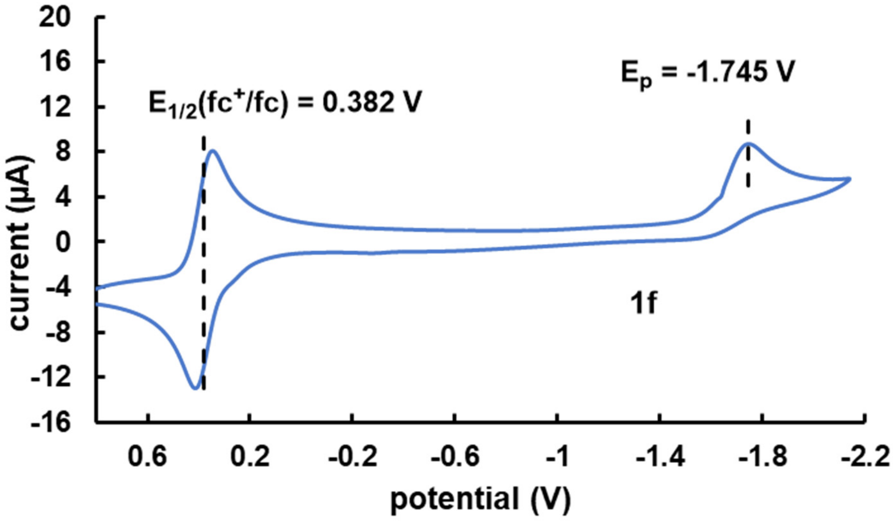

E1/2(fc+/fc) = 0.382 V
Ep = -1.745 V
1f
current (μA)
potential (V)

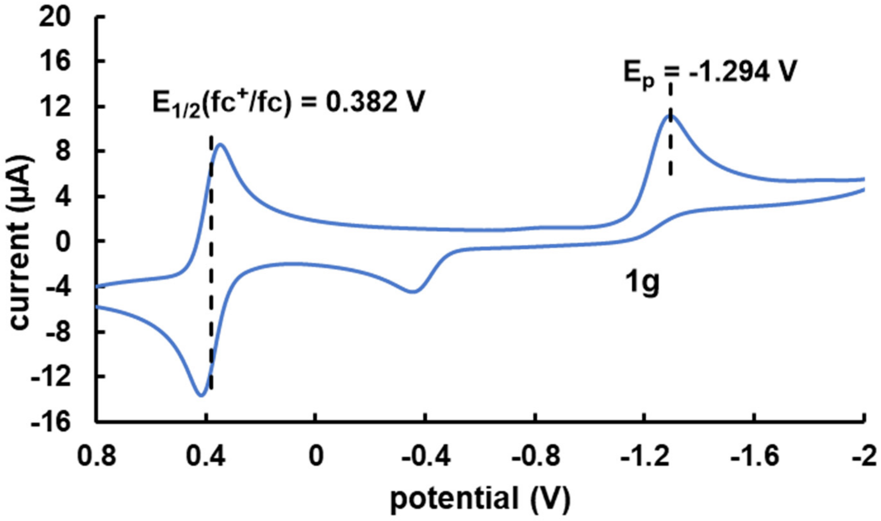

E1/2(fc+/fc) = 0.382 V
Ep = -1.294 V
1g
current (μA)
potential (V)
20
16
12
8
4
0
-4
-8
-12
-16
0.8
0.4
0
-0.4
-0.8
-1.2
-1.6
-2

7. Single Crystal X-Ray Structure Determinations

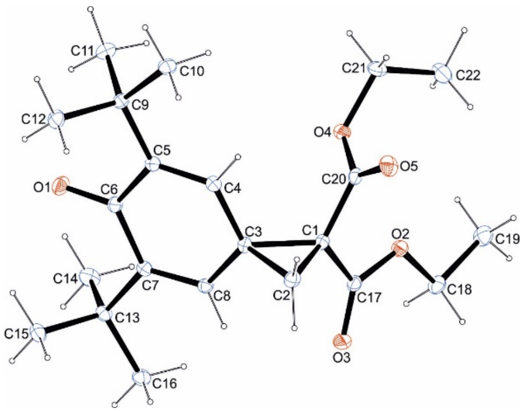

CCDC 2431023

3 (CG536_1F20-29_br006)

The X-ray intensity data of **3** (br006) were measured on a XtaLAB Synergy R, HyPix-Arc 150 system equipped with a mirror monochromator and a Cu Kα rotating-anode X-ray tube (λ = 1.54184 Å). The frames were integrated with the CrysAlisPro software package.[72] Data were corrected for absorption effects using the Multi-Scan method (SCALE3 ABSPACK) integrated in CrysAlisPro. The structure was solved with OLEX2.SOLVE[73] and refined using the SHELXLE Software Package.[74] All C-bound hydrogen atoms have been calculated in ideal geometry riding on their parent atoms. The figures have been drawn at the 25% ellipsoid probability level.[75]

net formula	$C_{22}H_{32}O_5$
M_r/g mol^{-1}	376.47
crystal size/mm	0.180 × 0.140 × 0.090
T/K	100.00(10)
radiation	CuKα
diffractometer	'XtaLAB Synergy R, HyPix-Arc 150'
crystal system	monoclinic
space group	'P 1 21/c 1'
a/Å	15.68849(14)
b/Å	10.57193(8)
c/Å	12.90346(12)
α/°	90
β/°	99.1414(8)
γ/°	90
V/Å^3	2112.96(3)
Z	4
calc. density/g cm^{-3}	1.183
μ/mm^{-1}	0.666
absorption correction	multi-scan
transmission factor range	0.86773–1.00000
refls. measured	22888
R_{int}	0.0141
mean σ(I)/I	0.0111
θ range	2.853–75.555
observed refls.	4012
x, y (weighting scheme)	0.0410, 0.6133
hydrogen refinement	constr
Flack parameter	?
refls in refinement	4325
parameters	253
restraints	0
$R(F_{obs})$	0.0322
$R_w(F^2)$	0.0853
S	1.050
shift/error$_{max}$	0.001
max electron density/e Å^{-3}	0.294
min electron density/e Å^{-3}	−0.171

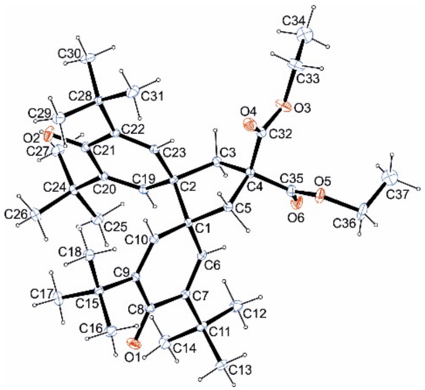

CCDC 2431024

4 (CG536_1F10-16_br007)

The X-ray intensity data of **4** (br007) were measured on a XtaLAB Synergy R, HyPix-Arc 150 system equipped with a mirror monochromator and a Cu Kα rotating-anode X-ray tube (λ = 1.54184 Å). The frames were integrated with the CrysAlisPro software package.[72] Data were corrected for absorption effects using the Multi-Scan method (SCALE3 ABSPACK) integrated in CrysAlisPro. The structure was solved with OLEX2.SOLVE[73] and refined using the SHELXLE Software Package.[74] All C-bound hydrogen atoms have been calculated in ideal geometry riding on their parent atoms. The disorder of two side chains has been described by split models. Split atoms have been refined partly isotropically (anisotropic refinement create new split suggestion). The figures have been drawn at the 25% ellipsoid probability level.[75]

net formula	$C_{37}H_{54}O_6$
M_r/g mol^{-1}	594.80
crystal size/mm	0.150 × 0.120 × 0.080
T/K	100(2)
radiation	CuKα
diffractometer	'XtaLAB Synergy R, HyPix-Arc 150'
crystal system	monoclinic
space group	'P 21/c'
a/Å	9.63184(5)
b/Å	19.83515(9)
c/Å	18.85208(9)
α/°	90
β/°	90.3018(4)
γ/°	90
V/Å^3	3601.62(3)
Z	4
calc. density/g cm^{-3}	1.097
μ/mm^{-1}	0.576
absorption correction	multi-scan
transmission factor range	0.88082–1.00000
refls. measured	43861
R_{int}	0.0151
mean σ(I)/I	0.0082
θ range	2.2274–75.6685
observed refls.	7005
x, y (weighting scheme)	0.0766, 1.9217
hydrogen refinement	constr
Flack parameter	?
refls in refinement	7441
parameters	411
restraints	0
$R(F_{obs})$	0.0553
$R_w(F^2)$	0.1539
S	1.076
shift/error$_{max}$	0.001
max electron density/e Å^{-3}	0.590
min electron density/e Å^{-3}	−0.624

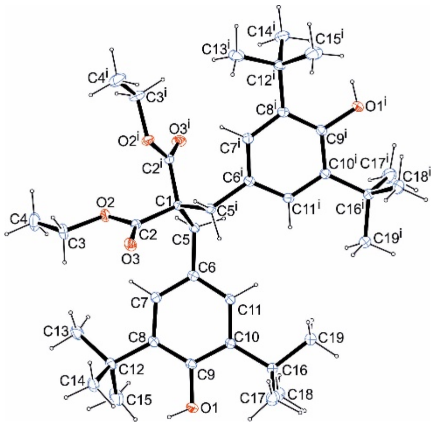

CCDC 2431025

6 (CG536_4_1_bv303)

The X-ray intensity data of **6** (bv303) were measured on a Bruker D8 Venture TXS system equipped with a multilayer mirror monochromator and a Mo Kα rotating anode X-ray tube (λ = 0.71073 Å). The frames were integrated with the Bruker SAINT software package.[76] Data were corrected for absorption effects using the Multi-Scan method (SADABS).[77] The structure was solved and refined using the Bruker SHELXTL Software Package.[78] All C-bound hydrogen atoms have been calculated in ideal geometry riding on their parent atoms, the O-bound hydrogen atom has been refined freely. The figures have been drawn at the 25% ellipsoid probability level.[75] Symmetry code for figure above: i = 1–*x*, *y*, 1.5–*z*.

net formula	$C_{37}H_{56}O_6$
M_r/g mol^{-1}	596.81
crystal size/mm	0.160 × 0.150 × 0.120
T/K	173.(2)
radiation	MoKα
diffractometer	'Bruker D8 Venture TXS'
crystal system	monoclinic
space group	'C 1 2/c 1'
a/Å	19.8525(4)
b/Å	13.4348(3)
c/Å	14.2279(3)
α/°	90
β/°	110.8130(10)
γ/°	90
V/Å^3	3547.16(13)
Z	4
calc. density/g cm^{-3}	1.118
μ/mm^{-1}	0.074
absorption correction	Multi-Scan
transmission factor range	0.96–0.99
refls. measured	30935
R_{int}	0.0392
mean σ(*I*)/*I*	0.0237
θ range	3.032–27.101
observed refls.	3233
x, y (weighting scheme)	0.0447, 2.9421
hydrogen refinement	mixed
Flack parameter	?
refls in refinement	3909
parameters	206
restraints	0
$R(F_{obs})$	0.0418
$R_w(F^2)$	0.1125
S	1.045
shift/error$_{max}$	0.001
max electron density/e Å^{-3}	0.256
min electron density/e Å^{-3}	−0.183

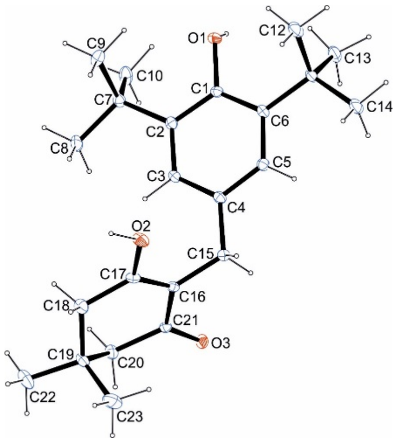

CCDC 2431026

9 (CG538_2_cv106)

The X-ray intensity data of **9** (cv106) were measured on a Bruker D8 Venture TXS system equipped with a multilayer mirror monochromator and a Mo Kα rotating anode X-ray tube (λ = 0.71073 Å). The frames were integrated with the Bruker SAINT software package.[76] Data were corrected for absorption effects using the Multi-Scan method (SADABS).[77] The structure was solved and refined using the Bruker SHELXTL Software Package.[78] All C-bound hydrogen atoms have been calculated in ideal geometry riding on their parent atoms whereas the O-bound hydrogen atoms have been refined freely. The structure has been refined as a 2-component perfect inversion twin. The figures have been drawn at the 25% ellipsoid probability level.[75]

net formula	$C_{23}H_{34}O_3$
M_r/g mol^{-1}	358.50
crystal size/mm	0.110 × 0.020 × 0.020
T/K	173.(2)
radiation	MoKα
diffractometer	'Bruker D8 Venture TXS'
crystal system	orthorhombic
space group	'P n a 21'
a/Å	13.3840(8)
b/Å	17.7258(11)
c/Å	8.9518(5)
α/°	90
β/°	90
γ/°	90
V/Å^3	2123.7(2)
Z	4
calc. density/g cm^{-3}	1.121
μ/mm^{-1}	0.072
absorption correction	Multi-Scan
transmission factor range	0.96–1.00
refls. measured	34319
R_{int}	0.0734
mean σ(*I*)/*I*	0.0388
θ range	3.044–25.350
observed refls.	3599
x, y (weighting scheme)	0.0411, 0.3076
hydrogen refinement	mixed
Flack parameter	0.5
refls in refinement	3839
parameters	251
restraints	1
$R(F_{obs})$	0.0361
$R_w(F^2)$	0.0860
S	1.086
shift/error$_{max}$	0.001
max electron density/e Å^{-3}	0.134
min electron density/e Å^{-3}	−0.149

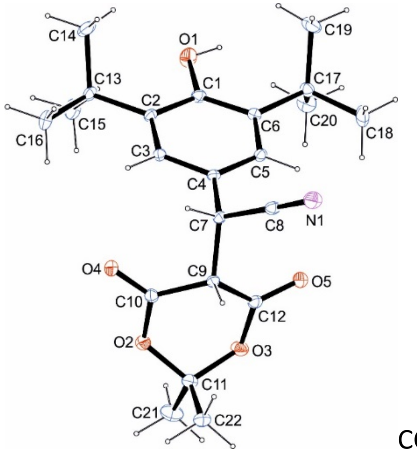

CCDC 2432306

12 (CG651_1)

The X-ray intensity data of **12** (dv077) were measured on a Bruker D8 Venture TXS system equipped with a multilayer mirror monochromator and a Mo Kα rotating anode X-ray tube (λ = 0.71073 Å). The frames were integrated with the Bruker SAINT software package.[76] Data were corrected for absorption effects using the Multi-Scan method (SADABS).[77] The structure was solved and refined using the Bruker SHELXTL Software Package.[78] All hydrogen atoms have been calculated in ideal geometry riding on their parent atoms. Free refinement of the H1 leads to a too short O1-H1 bond. The figures have been drawn at the 25% ellipsoid probability level.[75]

net formula	$C_{22}H_{29}NO_5$
M_r/g mol^{-1}	387.46
crystal size/mm	0.110 × 0.080 × 0.040
T/K	173.(2)
radiation	MoKα
diffractometer	'Bruker D8 Venture TXS'
crystal system	triclinic
space group	'P -1'
a/Å	9.9324(8)
b/Å	10.1092(9)
c/Å	11.4597(9)
α/°	70.997(3)
β/°	74.677(3)
γ/°	89.897(3)
V/Å^3	1044.78(15)
Z	2
calc. density/g cm^{-3}	1.232
μ/mm^{-1}	0.087
absorption correction	Multi-Scan
transmission factor range	0.92–1.00
refls. measured	18053
R_{int}	0.0471
mean σ(I)/I	0.0437
θ range	3.275–27.483
observed refls.	4014
x, y (weighting scheme)	0.0383, 0.7441
hydrogen refinement	constr
Flack parameter	?
refls in refinement	4745
parameters	262
restraints	3
$R(F_{obs})$	0.0501
$R_w(F^2)$	0.1276
S	1.053
shift/error$_{max}$	0.001
max electron density/e Å^{-3}	0.376
min electron density/e Å^{-3}	−0.226

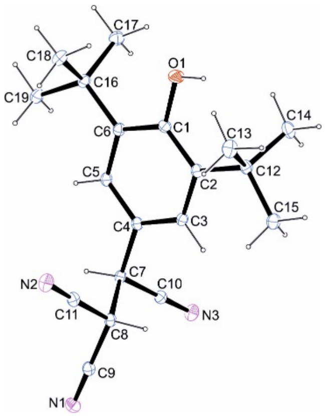

CCDC 2431027

13 (CG553_br015)

The X-ray intensity data of **13** (br015) were measured on a XtaLAB Synergy R, HyPix-Arc 150 system equipped with a mirror monochromator and a Cu Kα rotating-anode X-ray tube (λ = 1.54184 Å). The frames were integrated with the CrysAlisPro software package.[72] Data were corrected for absorption effects using the Multi-Scan method (SCALE3 ABSPACK) integrated in CrysAlisPro. The structure was solved with SHELXT[78] and refined using the SHELXLE software package.[74] All hydrogen atoms have been calculated in ideal geometry riding on their parent atoms. Free refinement of H1 leads to a wrong O1-H1 distance of 0.68 Å. The figures have been drawn at the 25% ellipsoid probability level.[75]

net formula	$C_{19}H_{23}N_3O$
M_r/g mol^{-1}	309.40
crystal size/mm	0.16 × 0.09 × 0.08
T/K	100(2)
radiation	CuKα
diffractometer	'XtaLAB Synergy R, HyPix-Arc 150'
crystal system	triclinic
space group	'P -1'
a/Å	5.94490(10)
b/Å	9.27980(10)
c/Å	16.32000(10)
α/°	93.5500(10)
β/°	95.9450(10)
γ/°	93.8890(10)
V/Å^3	891.270(19)
Z	2
calc. density/g cm^{-3}	1.153
μ/mm^{-1}	0.571
absorption correction	multi-scan
transmission factor range	0.96944–1.00000
refls. measured	30400
R_{int}	0.0227
mean σ(I)/I	0.0130
θ range	2.729–75.325
observed refls.	3258
x, y (weighting scheme)	0.0453, 0.1892
hydrogen refinement	constr
Flack parameter	_refine_ls_abs_structure_Flack
refls in refinement	3578
parameters	216
restraints	2
$R(F_{obs})$	0.0336
$R_w(F^2)$	0.0931
S	1.095
shift/error$_{max}$	0.001
max electron density/e Å^{-3}	0.242
min electron density/e Å^{-3}	−0.160

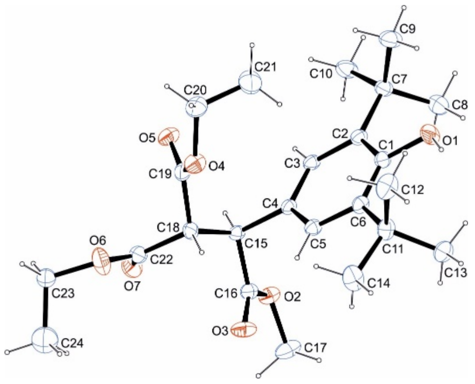

CCDC 2431028

14 (CG524_cv127)

The X-ray intensity data of **14** (cv127) were measured on a Bruker D8 Venture TXS system equipped with a multilayer mirror monochromator and a Mo Kα rotating anode X-ray tube (λ = 0.71073 Å). The frames were integrated with the Bruker SAINT software package.[76] Data were corrected for absorption effects using the Multi-Scan method (SADABS).[77] The structure was solved and refined using the Bruker SHELXTL Software Package.[78] All hydrogen atoms have been calculated in ideal geometry riding on their parent atoms. The asymmetric unit contains two formula units. One of the molecules shows only a disorder of an ethyl group. The other molecule is almost completely disordered. The disorders have been described by split models. Disordered atoms have been refined isotropically. The figures have been drawn at the 25% ellipsoid probability level.[75] In the case of disorder, the less-occupied parts have been neglected for the figures. In the figure above only one of the two molecules in the asymmetric unit has been depicted.

net formula	$C_{24}H_{36}O_7$
M_r/g mol^{-1}	436.53
crystal size/mm	0.130 × 0.090 × 0.070
T/K	173.(2)
radiation	MoKα
diffractometer	'Bruker D8 Venture TXS'
crystal system	triclinic
space group	'P -1'
a/Å	13.2864(6)
b/Å	15.0339(7)
c/Å	15.1617(7)
α/°	67.8270(10)
β/°	65.0750(10)
γ/°	85.373(2)
V/Å^3	2532.2(2)
Z	4
calc. density/g cm^{-3}	1.145
μ/mm^{-1}	0.083
absorption correction	Multi-Scan
transmission factor range	0.94–0.99
refls. measured	41669
R_{int}	0.0374
mean σ(I)/I	0.0318
θ range	2.574–25.350
observed refls.	6857
x, y (weighting scheme)	0.0354, 4.2812
hydrogen refinement	constr
Flack parameter	?
refls in refinement	9251
parameters	584
restraints	4
$R(F_{obs})$	0.0776
$R_w(F^2)$	0.1834
S	1.087
shift/error$_{max}$	0.001
max electron density/e Å^{-3}	0.445
min electron density/e Å^{-3}	−0.571

8. Kinetics of the Reactions of *p*QMs with Carbanions

All solutions were prepared by using dry DMSO (ThermoScientific, DMSO 99.7+%, extra dry, over molecular sieve, AcroSeal) and kept under an atmosphere of dry nitrogen. The kinetic measurements for each *p*QM/nucleophile combination **1** + **2** were performed with or without added 18-crown-6 ether (18-c-6) and in some cases with addition of the corresponding CH-acid (**2**-H). Uncertainty of reactant concentrations is assumed to be in the range of ±5%, and uncertainty of k_{obs} values is generally ±10%.

The raw data of kinetic measurements that support the findings of this study are openly available in Open Data LMU at DOI: 10.5282/ubm/data.582.

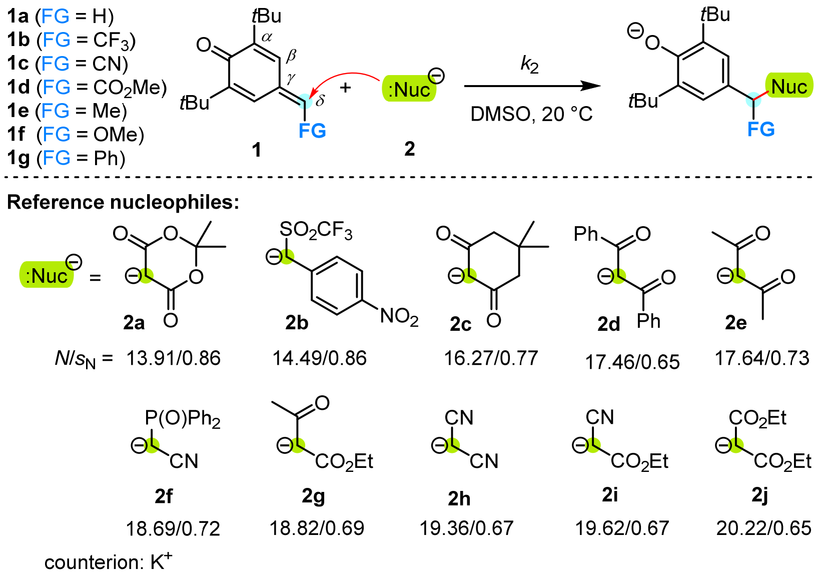

1a + **2a** in DMSO (stopped-flow, detection at 310 nm) CG544

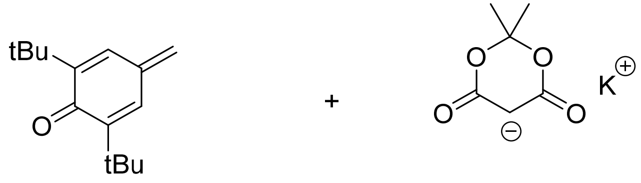

1a **2a**

[**1a**]$_0$ (M)	[**2a**]$_0$ (M)	[**2a**-H]$_0$ (M)	[18-c-6]$_0$ (M)	k_{obs} (s^{-1})
2.50×10^{-5}	1.20×10^{-3}	1.20×10^{-3}		1.56×10^{-1}
2.50×10^{-5}	1.80×10^{-3}	1.80×10^{-3}	1.98×10^{-3}	2.26×10^{-1}
2.50×10^{-5}	2.40×10^{-3}	2.40×10^{-3}		2.94×10^{-1}
2.50×10^{-5}	3.00×10^{-3}	3.00×10^{-3}	3.30×10^{-3}	3.69×10^{-1}
2.50×10^{-5}	3.60×10^{-3}	3.60×10^{-3}		4.36×10^{-1}

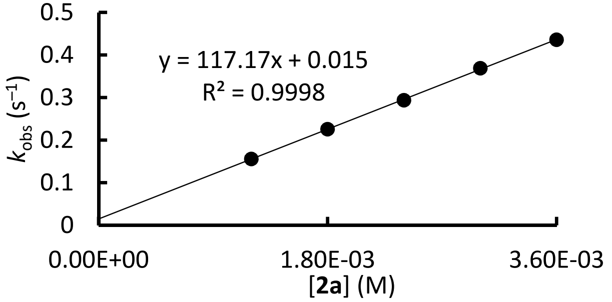

$k_2 = (1.17 \pm 0.01) \times 10^2$ M^{-1} s^{-1}

1a + **2b** in DMSO (stopped-flow, detection at 320 nm) CG708

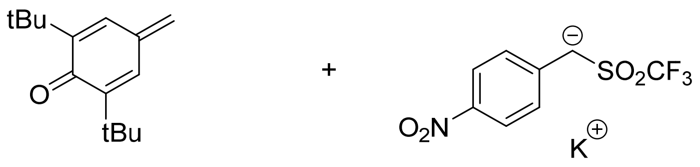

1a **2b**

[**1a**]$_0$ (M)	[**2b**]$_0$ (M)	[18-c-6]$_0$ (M)	k_{obs} (s^{-1})
5.50×10^{-5}	3.00×10^{-4}		5.46×10^{-1}
5.50×10^{-5}	4.50×10^{-4}	4.95×10^{-4}	7.98×10^{-1}
5.50×10^{-5}	6.00×10^{-4}		1.06
5.50×10^{-5}	7.50×10^{-4}	8.25×10^{-4}	1.30
5.50×10^{-5}	9.00×10^{-4}		1.59

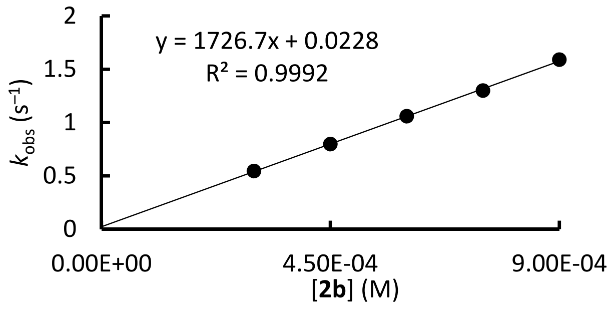

k_2 = (1.73 ± 0.03) × 10^3 M^{-1} s^{-1}

1a+ **2c** in DMSO (stopped-flow, detection at 310 nm) CG541

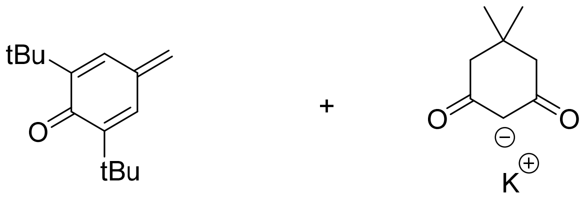

1a **2c**

[**1a**]$_0$ (M)	[**2c**]$_0$ (M)	[**2c**-H]$_0$ (M)	[18-c-6]$_0$ (M)	k_{obs} (s^{-1})
2.18×10^{-5}	6.00×10^{-4}	6.00×10^{-4}		5.79
2.18×10^{-5}	9.00×10^{-4}	9.00×10^{-4}	9.90×10^{-4}	7.91
2.18×10^{-5}	1.20×10^{-3}	1.20×10^{-3}		9.92
2.18×10^{-5}	1.50×10^{-3}	1.50×10^{-3}	1.65×10^{-3}	11.9

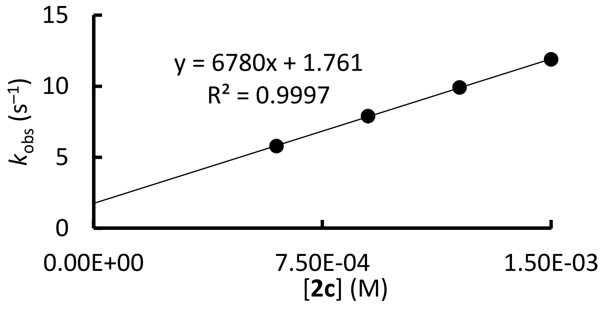

k_2 = (6.78 ± 0.08) × 10^3 M^{-1} s^{-1}

1c + **2a** in DMSO (stopped-flow, detection at 318 nm) CG081

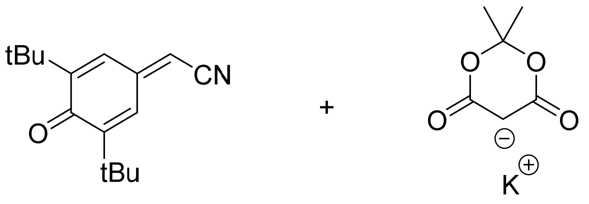

1c **2a**

$[\mathbf{1c}]_0$ (M)	$[\mathbf{2a}]_0$ (M)	$[\text{18-c-6}]_0$ (M)	k_{obs} (s^{-1})
1.90×10^{-5}	1.75×10^{-4}		3.32×10^{-3}
1.90×10^{-5}	2.63×10^{-4}	2.89×10^{-4}	5.59×10^{-3}
1.90×10^{-5}	3.50×10^{-4}		7.39×10^{-3}
1.90×10^{-5}	4.38×10^{-4}	4.82×10^{-4}	9.25×10^{-3}
1.90×10^{-5}	5.25×10^{-4}		1.15×10^{-2}

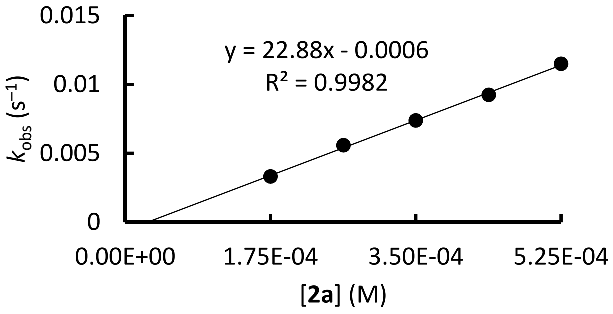

k_2 **= (2.29 ± 0.06) × 10**^{1} **M**$^{-1}$ **s**$^{-1}$

1c + **2c** in DMSO (stopped-flow, detection at 318 nm) CG071

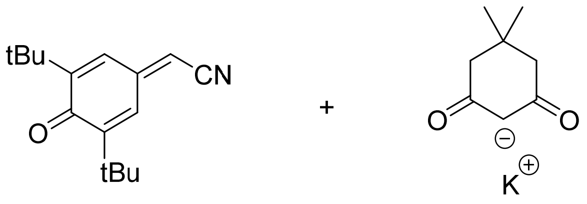

1c **2c**

$[\mathbf{1c}]_0$ (M)	$[\mathbf{2c}]_0$ (M)	$[\mathbf{2c}\text{-H}]_0$ (M)	$[\text{18-c-6}]_0$ (M)	k_{obs} (s^{-1})
1.90×10^{-5}	1.75×10^{-4}	1.75×10^{-4}		8.97×10^{-1}
1.90×10^{-5}	2.63×10^{-4}	2.63×10^{-4}	2.89×10^{-4}	1.34
1.90×10^{-5}	3.50×10^{-4}	3.50×10^{-4}		1.76
1.90×10^{-5}	4.38×10^{-4}	4.38×10^{-4}	4.82×10^{-4}	2.19
1.90×10^{-5}	5.25×10^{-4}	5.25×10^{-4}		2.65

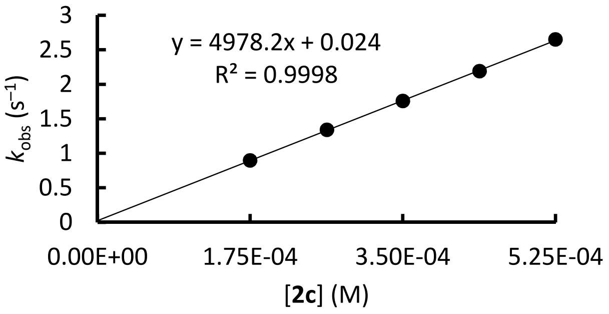

k_2 **= (4.98 ± 0.04) × 10**^{3} **M**$^{-1}$ **s**$^{-1}$

1c + **2d** in DMSO (stopped-flow, detection at 318 nm) CG083

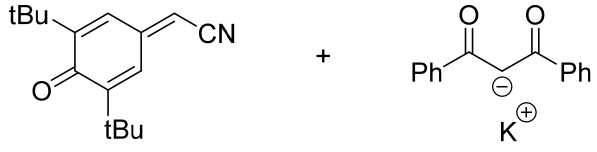

1c **2d**

[**1c**]$_0$ (M)	[**2d**]$_0$ (M)	[18-c-6]$_0$ (M)	k_{obs} (s^{-1})
1.13×10^{-5}	1.13×10^{-4}		2.95×10^{-1}
1.13×10^{-5}	1.69×10^{-4}	1.86×10^{-4}	4.58×10^{-1}
1.13×10^{-5}	2.25×10^{-4}		5.83×10^{-1}
1.13×10^{-5}	2.82×10^{-4}	3.10×10^{-4}	7.57×10^{-1}

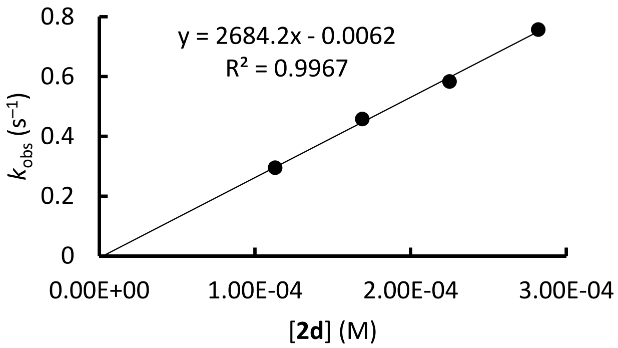

$\boldsymbol{k_2 = (2.68 \pm 0.11) \times 10^3\ M^{-1}\ s^{-1}}$

1c + **2h** in DMSO (stopped-flow, detection at 318 nm) CG074

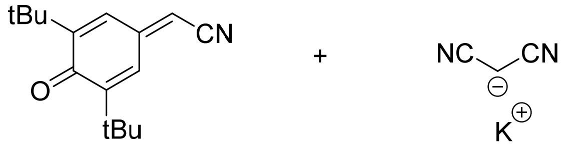

1c **2h**

[**1c**]$_0$ (M)	[**2h**]$_0$ (M)	[18-c-6]$_0$ (M)	k_{obs} (s^{-1})
1.75×10^{-5}	1.75×10^{-4}		1.62×10^{1}
1.75×10^{-5}	2.63×10^{-4}	2.89×10^{-4}	2.37×10^{1}
1.75×10^{-5}	3.50×10^{-4}		3.21×10^{1}
1.75×10^{-5}	4.38×10^{-4}	4.82×10^{-4}	3.98×10^{1}
1.75×10^{-5}	5.25×10^{-4}		4.71×10^{1}

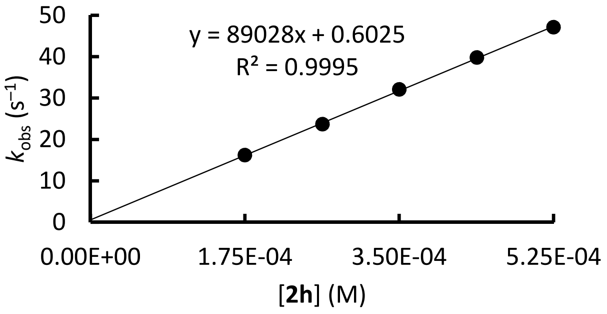

$\boldsymbol{k_2 = (8.90 \pm 0.12) \times 10^4\ M^{-1}\ s^{-1}}$

1c + **2i** in DMSO (stopped-flow, detection at 318 nm) CG186

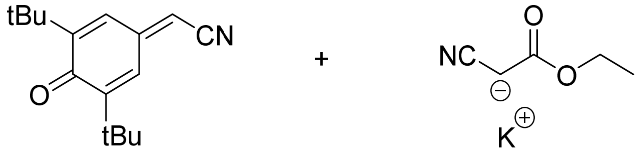

1c **2i**

[**1c**]$_0$ (M)	[**2i**]$_0$ (M)	[**2i**-H]$_0$ (M)	[18-c-6]$_0$ (M)	k_{obs} (s^{-1})
2.03×10^{-5}	1.75×10^{-4}	1.75×10^{-4}		6.36×10^{1}
2.03×10^{-5}	2.63×10^{-4}	2.63×10^{-4}	2.89×10^{-4}	9.48×10^{1}
2.03×10^{-5}	3.50×10^{-4}	3.50×10^{-4}		1.31×10^{2}
2.03×10^{-5}	4.38×10^{-4}	4.38×10^{-4}	4.82×10^{-4}	1.60×10^{2}

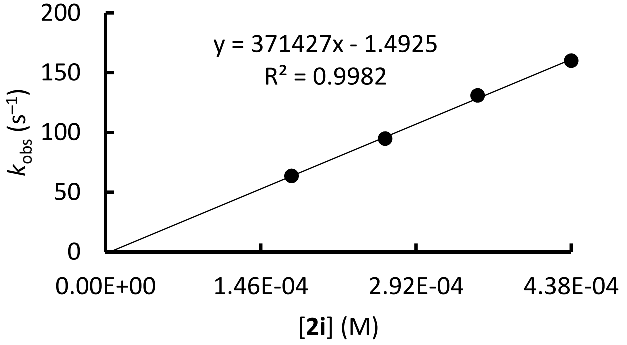

$k_2 = (3.71 \pm 0.11) \times 10^{5}\ M^{-1}\ s^{-1}$

1d + **2c** in DMSO (stopped-flow, detection at 309 nm) CG534

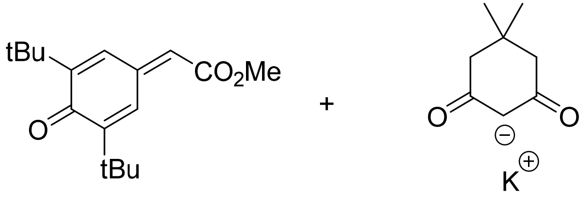

1d **2c**

[**1d**]$_0$ (M)	[**2c**]$_0$ (M)	[**2c**-H]$_0$ (M)	[18-c-6]$_0$ (M)	k_{obs} (s^{-1})
2.22×10^{-5}	2.00×10^{-4}	2.00×10^{-4}		4.95×10^{-1}
2.22×10^{-5}	3.00×10^{-4}	3.00×10^{-4}	3.30×10^{-4}	7.07×10^{-1}
2.22×10^{-5}	4.00×10^{-4}	4.00×10^{-4}		9.39×10^{-1}
2.22×10^{-5}	5.00×10^{-4}	5.00×10^{-4}	5.50×10^{-4}	1.16
2.22×10^{-5}	6.00×10^{-4}	6.00×10^{-4}		1.38

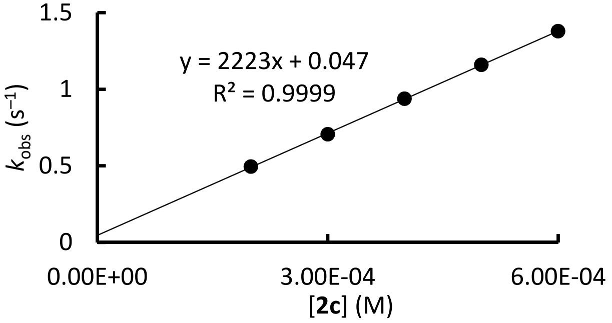

$k_2 = (2.22 \pm 0.02) \times 10^{3}\ M^{-1}\ s^{-1}$

1d + **2e** in DMSO (stopped-flow, detection at 340 nm) CG533

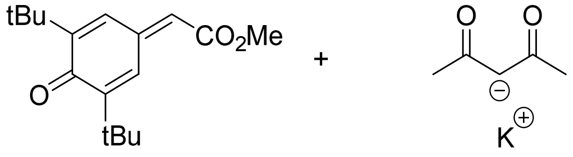

1d **2e**

[**1d**]$_0$ (M)	[**2e**]$_0$ (M)	[**2e**-H]$_0$ (M)	[18-c-6]$_0$ (M)	k_{obs} (s^{-1})
9.78×10^{-5}	9.50×10^{-4}	9.50×10^{-4}		9.42
9.78×10^{-5}	1.43×10^{-3}	1.43×10^{-3}	1.57×10^{-3}	1.38×10^{1}
9.78×10^{-5}	1.90×10^{-3}	1.90×10^{-3}		1.83×10^{1}
9.78×10^{-5}	2.38×10^{-3}	2.38×10^{-3}	2.61×10^{-3}	2.34×10^{1}
9.78×10^{-5}	2.85×10^{-3}	2.85×10^{-3}		2.73×10^{1}

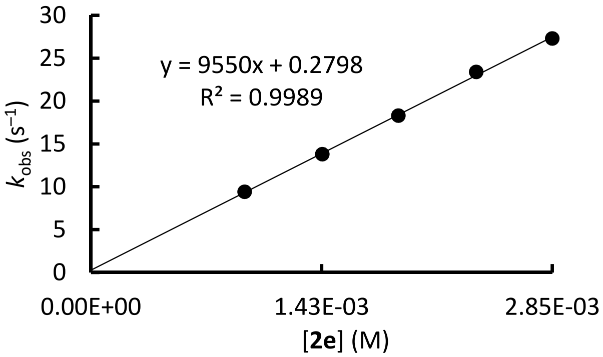

$\boldsymbol{k_2 = (9.55 \pm 0.18) \times 10^3\ M^{-1}\ s^{-1}}$

1d + **2g** in DMSO (stopped-flow, detection at 309 nm) CG531

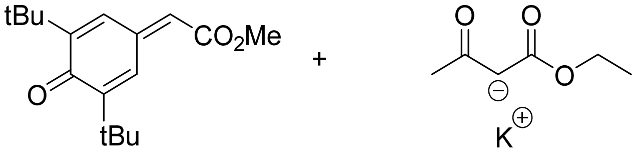

1d **2g**

[**1d**]$_0$ (M)	[**2g**]$_0$ (M)	[**2g**-H]$_0$ (M)	[18-c-6]$_0$ (M)	k_{obs} (s^{-1})
1.54×10^{-5}	2.00×10^{-4}	2.00×10^{-4}		1.56×10^{1}
1.54×10^{-5}	3.00×10^{-4}	3.00×10^{-4}	3.30×10^{-4}	2.34×10^{1}
1.54×10^{-5}	4.00×10^{-4}	4.00×10^{-4}		3.09×10^{1}
1.54×10^{-5}	5.00×10^{-4}	5.00×10^{-4}	5.50×10^{-4}	3.82×10^{1}
1.54×10^{-5}	6.00×10^{-4}	6.00×10^{-4}		4.61×10^{1}

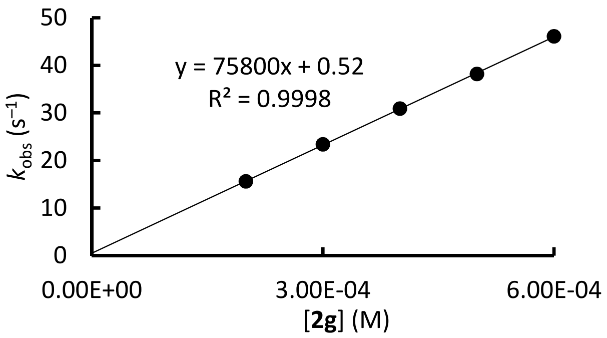

$\boldsymbol{k_2 = (7.58 \pm 0.05) \times 10^4\ M^{-1}\ s^{-1}}$

1d + **2h** in DMSO (stopped-flow, detection at 309 nm) CG530

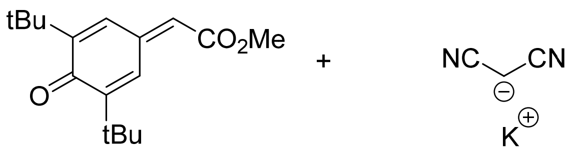

1d **2h**

[**1d**]$_0$ (M)	[**2h**]$_0$ (M)	[18-c-6]$_0$ (M)	k_{obs} (s^{-1})
1.70×10^{-5}	1.00×10^{-4}		2.75
1.70×10^{-5}	1.50×10^{-4}	1.65×10^{-4}	3.83
1.70×10^{-5}	2.00×10^{-4}		5.46
1.70×10^{-5}	2.50×10^{-4}	2.75×10^{-4}	6.53
1.70×10^{-5}	3.00×10^{-4}		7.72

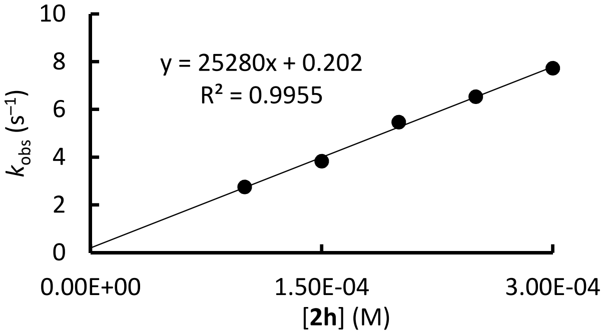

k_2 = (2.53 ± 0.10) × 10^4 M^{-1} s^{-1}

1d + **2i** in DMSO (stopped-flow, detection at 309 nm) CG532

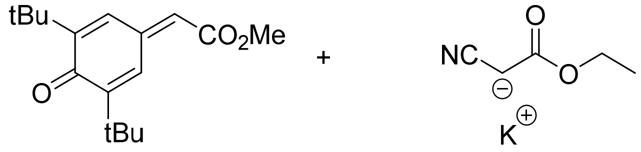

1d **2i**

[**1d**]$_0$ (M)	[**2i**]$_0$ (M)	[**2i**-H]$_0$ (M)	[18-c-6]$_0$ (M)	k_{obs} (s^{-1})
1.75×10^{-5}	1.00×10^{-4}	1.00×10^{-4}		1.06×10^{1}
1.75×10^{-5}	1.50×10^{-4}	1.50×10^{-4}	1.65×10^{-4}	1.59×10^{1}
1.75×10^{-5}	2.00×10^{-4}	2.00×10^{-4}		2.13×10^{1}
1.75×10^{-5}	2.50×10^{-4}	2.50×10^{-4}	2.75×10^{-4}	2.60×10^{1}
1.75×10^{-5}	3.00×10^{-4}	3.00×10^{-4}		3.16×10^{1}

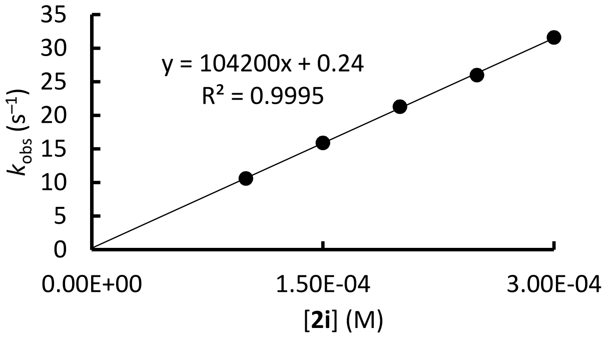

k_2 = (1.04 ± 0.01) × 10^5 M^{-1} s^{-1}

1d + **2j** in DMSO (stopped-flow, detection at 309 nm) CG523_1

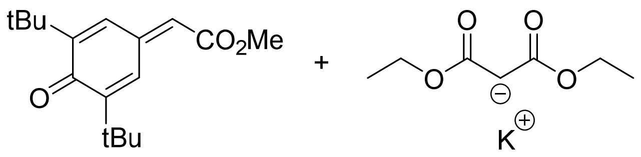

1d **2j**

[**1d**]$_0$ (M)	[**2j**]$_0$ (M)	[18-c-6]$_0$ (M)	k_{obs} (s^{-1})
1.38×10^{-5}	1.00×10^{-4}		6.42×10^{1}
1.38×10^{-5}	1.50×10^{-4}	1.65×10^{-4}	9.74×10^{1}
1.38×10^{-5}	2.00×10^{-4}		1.31×10^{2}
1.38×10^{-5}	2.50×10^{-4}	2.75×10^{-4}	1.64×10^{2}
1.38×10^{-5}	3.00×10^{-4}		1.98×10^{2}

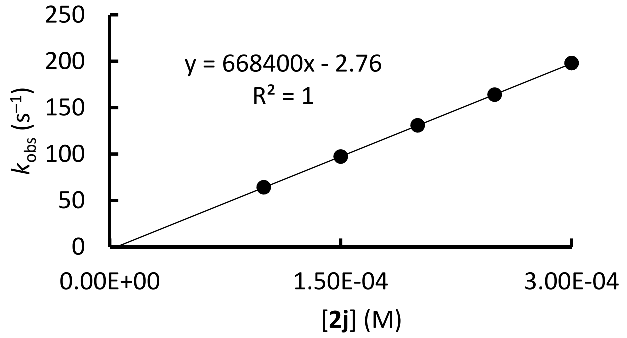

$k_2 = (6.68 \pm 0.02) \times 10^5\ M^{-1}\ s^{-1}$

1e+ **2c** in DMSO (stopped-flow, detection at 310 nm) CG445_1

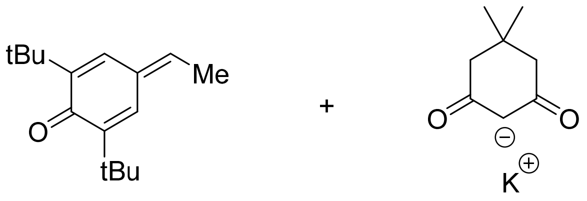

1e **2c**

[**1e**]$_0$ (M)	[**2c**]$_0$ (M)	[**2c**-H]$_0$ (M)	[18-c-6]$_0$ (M)	k_{obs} (s^{-1})
2.18×10^{-5}	3.00×10^{-4}	3.00×10^{-4}		9.45×10^{-2}
2.18×10^{-5}	4.50×10^{-4}	4.50×10^{-4}	4.95×10^{-4}	1.41×10^{-1}
2.18×10^{-5}	6.00×10^{-4}	6.00×10^{-4}		1.79×10^{-1}
2.18×10^{-5}	7.50×10^{-4}	7.50×10^{-4}	8.25×10^{-4}	2.22×10^{-1}
	9.00×10^{-4}	9.00×10^{-4}		2.59×10^{-1}

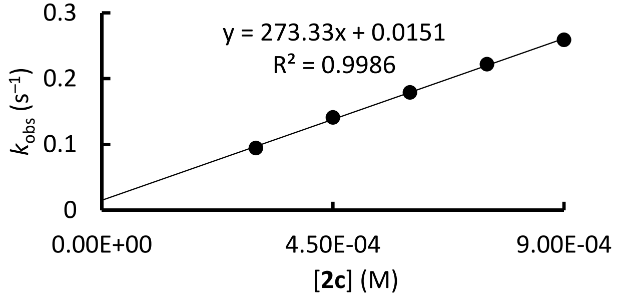

$k_2 = (2.73 \pm 0.06) \times 10^2\ M^{-1}\ s^{-1}$

1e + **2g** in DMSO (stopped-flow, detection at 320 nm) CG444

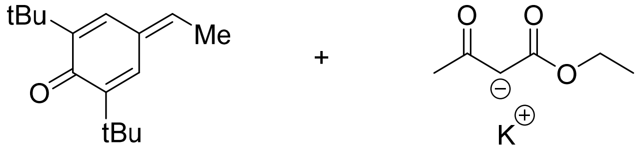

1e **2g**

[**1e**]$_0$ (M)	[**2g**]$_0$ (M)	[18-c-6]$_0$ (M)	k_{obs} (s^{-1})
1.97×10^{-5}	6.00×10^{-4}		2.07
1.97×10^{-5}	9.00×10^{-4}	9.90×10^{-4}	3.46
1.97×10^{-5}	1.20×10^{-3}		4.38
1.97×10^{-5}	1.50×10^{-3}	1.65×10^{-3}	5.33
1.97×10^{-5}	1.80×10^{-3}		6.41

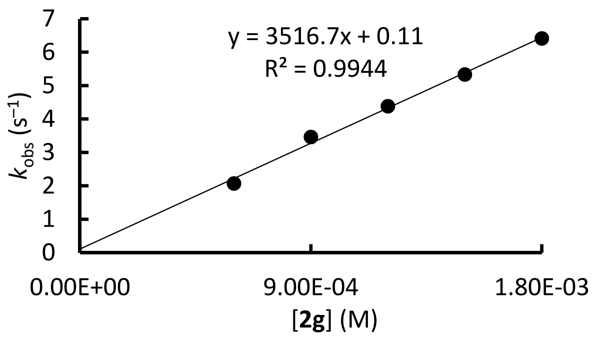

k_2 = (3.52 ± 0.15) × 10^3 M^{-1} s^{-1}

1e + **2h** in DMSO (stopped-flow, detection at 320 nm) CG441

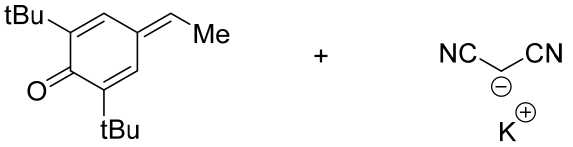

1e **2h**

[**1e**]$_0$ (M)	[**2h**]$_0$ (M)	[18-c-6]$_0$ (M)	k_{obs} (s^{-1})
1.97×10^{-5}	3.00×10^{-4}		1.59
1.97×10^{-5}	4.50×10^{-4}	4.95×10^{-4}	2.24
1.97×10^{-5}	6.00×10^{-4}		3.00
1.97×10^{-5}	7.50×10^{-4}	8.25×10^{-4}	3.70
1.97×10^{-5}	9.00×10^{-4}		4.27

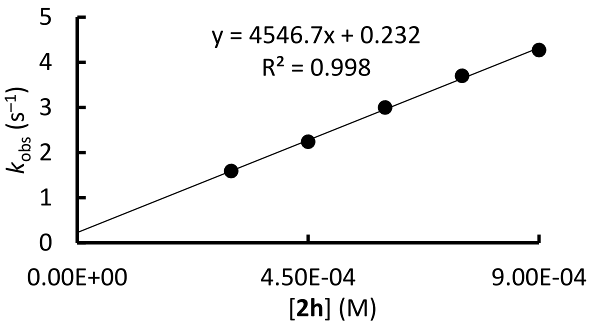

k_2 = (4.55 ± 0.12) × 10^3 M^{-1} s^{-1}

1e + **2i** in DMSO (stopped-flow, detection at 320 nm) CG443

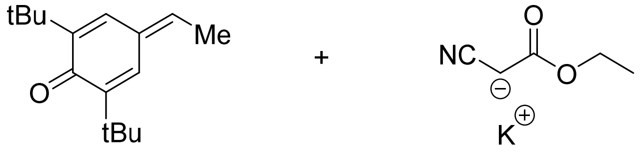

1e **2i**

[**1e**]$_0$ (M)	[**2i**]$_0$ (M)	[18-c-6]$_0$ (M)	k_{obs} (s^{-1})
1.97×10^{-5}	3.00×10^{-4}		1.44
1.97×10^{-5}	4.50×10^{-4}	4.95×10^{-4}	2.29
1.97×10^{-5}	6.00×10^{-4}		2.87
1.97×10^{-5}	7.50×10^{-4}	8.25×10^{-4}	3.83
1.97×10^{-5}	9.00×10^{-4}		4.46

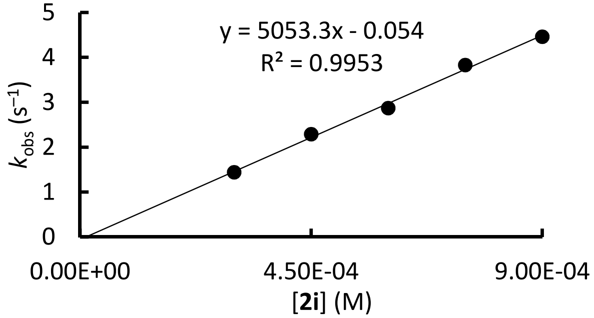

$\boldsymbol{k_2 = (5.05 \pm 0.20) \times 10^3\ M^{-1}\ s^{-1}}$

1e + **2j** in DMSO (stopped-flow, detection at 320 nm) CG442

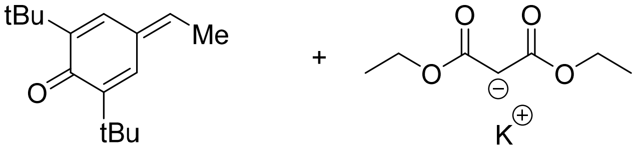

1e **2j**

[**1e**]$_0$ (M)	[**2j**]$_0$ (M)	[18-c-6]$_0$ (M)	k_{obs} (s^{-1})
1.97×10^{-5}	3.00×10^{-4}		4.28
1.97×10^{-5}	4.50×10^{-4}	4.95×10^{-4}	6.47
1.97×10^{-5}	6.00×10^{-4}		8.75
1.97×10^{-5}	7.50×10^{-4}	8.25×10^{-4}	1.12×10^1
1.97×10^{-5}	9.00×10^{-4}		1.30×10^1

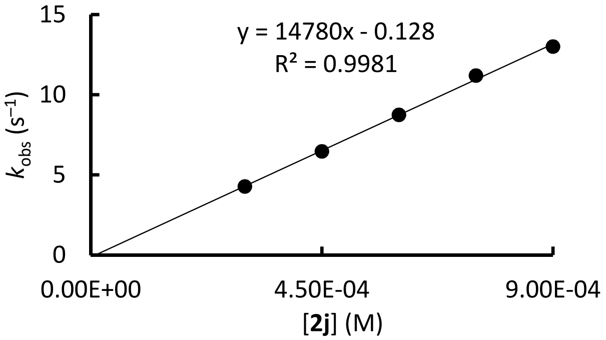

$\boldsymbol{k_2 = (1.48 \pm 0.04) \times 10^4\ M^{-1}\ s^{-1}}$

1f + **2e** in DMSO (stopped-flow, detection at 345 nm) CG529

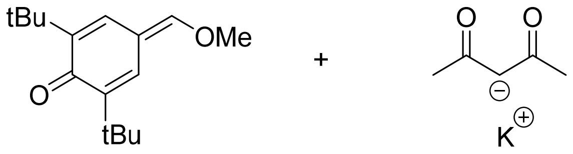

1f **2e**

[**1f**]$_0$ (M)	[**2e**]$_0$ (M)	[**2e**-H]$_0$, (M)	[18-c-6]$_0$ (M)	k_{obs} (s^{-1})
1.80×10^{-5}	4.40×10^{-4}	4.40×10^{-4}		2.27×10^{-2}
1.80×10^{-5}	6.60×10^{-4}	6.60×10^{-4}	7.26×10^{-4}	3.55×10^{-2}
1.80×10^{-5}	8.80×10^{-4}	8.80×10^{-4}		4.48×10^{-2}
1.80×10^{-5}	1.10×10^{-3}	1.10×10^{-3}	1.21×10^{-3}	5.59×10^{-2}
1.80×10^{-5}	1.32×10^{-3}	1.32×10^{-3}		6.58×10^{-2}

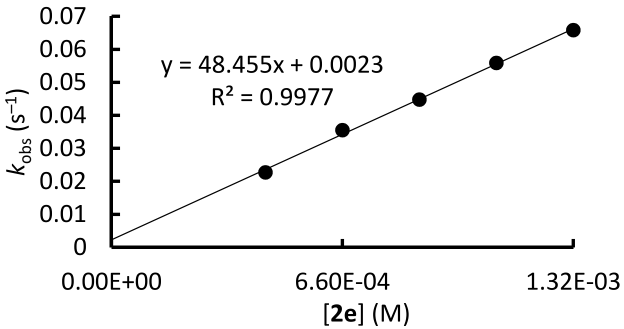

k_2 **= (4.85 ± 0.14) × 10**^{1} **M**$^{-1}$ **s**$^{-1}$

1f + **2g** in DMSO (stopped-flow, detection at 345 nm) CG528

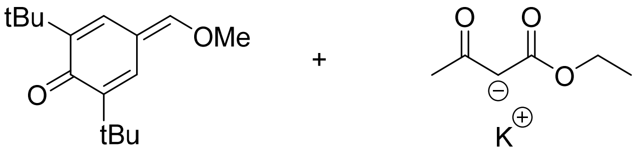

1f **2g**

[**1f**]$_0$ (M)	[**2g**]$_0$ (M)	[18-c-6]$_0$ (M)	k_{obs} (s^{-1})
1.97×10^{-5}	2.20×10^{-4}		1.53×10^{-1}
1.97×10^{-5}	3.30×10^{-4}	3.63×10^{-4}	1.99×10^{-1}
1.97×10^{-5}	4.40×10^{-4}		2.44×10^{-1}
1.97×10^{-5}	5.50×10^{-4}	6.05×10^{-4}	2.85×10^{-1}
1.97×10^{-5}	6.60×10^{-4}		3.46×10^{-1}

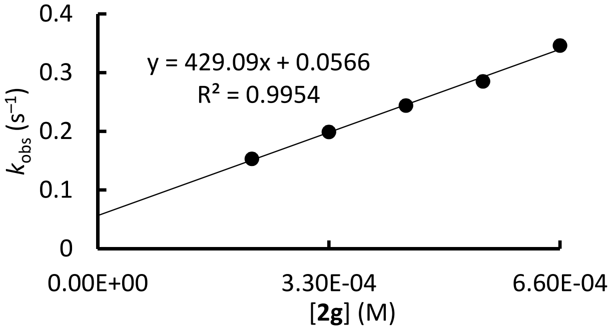

k_2 **= (4.29 ± 0.17) × 10**^{2} **M**$^{-1}$ **s**$^{-1}$

1f + **2h** in DMSO (stopped-flow, detection at 345 nm) CG527

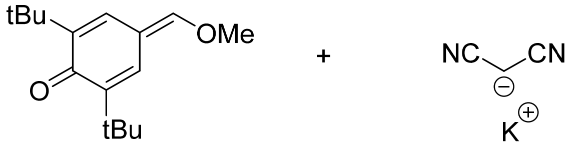

1f **2h**

[**1f**]$_0$ (M)	[**2h**]$_0$ (M)	[18-c-6]$_0$ (M)	k_{obs} (s^{-1})
2.04×10^{-5}	2.20×10^{-4}		2.28×10^{-1}
2.04×10^{-5}	3.30×10^{-4}	3.63×10^{-4}	3.45×10^{-1}
2.04×10^{-5}	4.40×10^{-4}		4.49×10^{-1}
2.04×10^{-5}	5.50×10^{-4}	6.05×10^{-4}	5.77×10^{-1}
2.04×10^{-5}	6.60×10^{-4}		6.86×10^{-1}

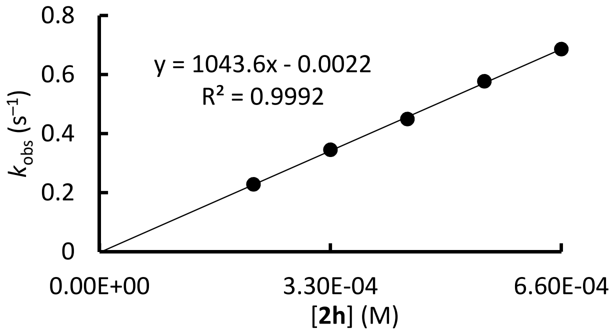

k_2 = **(1.04 ± 0.02) × 10^3 M^{-1} s^{-1}**

1f + **2j** in DMSO (stopped-flow, detection at 345 nm) CG526

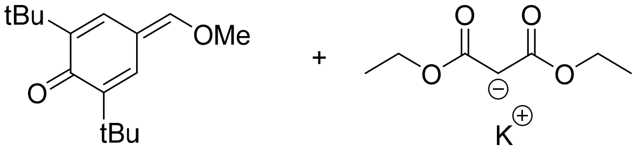

1f **2j**

[**1f**]$_0$ (M)	[**2j**]$_0$ (M)	[18-c-6]$_0$ (M)	k_{obs} (s^{-1})
2.08×10^{-5}	2.20×10^{-4}		4.31×10^{-1}
2.08×10^{-5}	3.30×10^{-4}	3.63×10^{-4}	6.72×10^{-1}
2.08×10^{-5}	4.40×10^{-4}		8.55×10^{-1}
2.08×10^{-5}	5.50×10^{-4}	6.05×10^{-4}	1.09
2.08×10^{-5}	6.60×10^{-4}		1.26

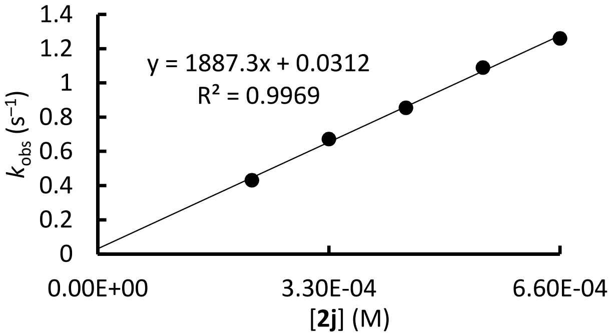

k_2 = **(1.89 ± 0.06) × 10^3 M^{-1} s^{-1}**

1g + **2c** in DMSO (conventional photometry, detection at 359 nm) CG116

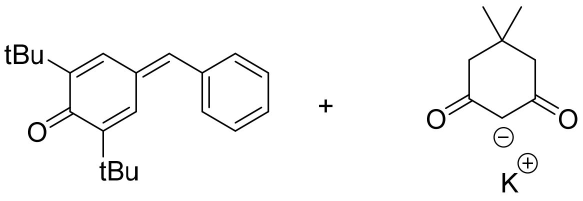

1g **2c**

$[\mathbf{1g}]_0$ (M)	$[\mathbf{2c}]_0$ (M)	$[\mathbf{2c}\text{-H}]_0$ (M)	$[18\text{-c-}6]_0$ (M)	k_{obs} (s^{-1})
3.82×10^{-5}	3.80×10^{-4}	3.80×10^{-4}		1.15×10^{-3}
3.65×10^{-5}	5.50×10^{-4}	5.50×10^{-4}	6.04×10^{-4}	1.51×10^{-3}
3.68×10^{-5}	7.40×10^{-4}	7.40×10^{-4}		2.06×10^{-3}
3.58×10^{-5}	9.00×10^{-4}	9.00×10^{-4}		2.50×10^{-3}

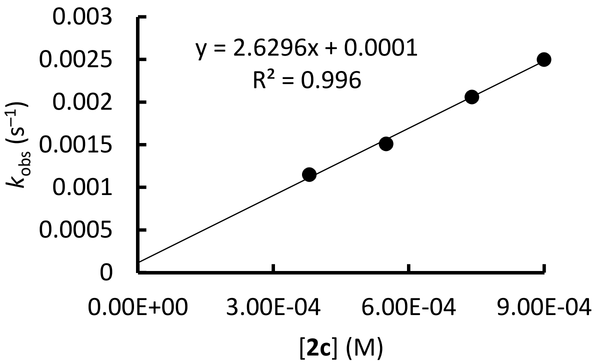

$\boldsymbol{k_2 = (2.63 \pm 0.12)\ M^{-1}\ s^{-1}}$

1g + **2e** in DMSO (stopped-flow, detection at 359 nm) CG115

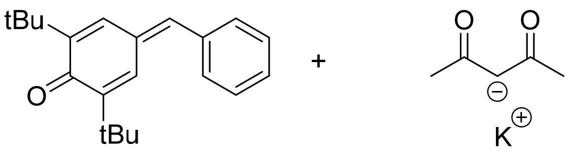

1g **2e**

$[\mathbf{1g}]_0$ (M)	$[\mathbf{2e}]_0$ (M)	$[\mathbf{2e}\text{-H}]_0$ (M)	$[18\text{-c-}6]_0$ (M)	k_{obs} (s^{-1})
2.20×10^{-5}	2.25×10^{-4}	2.25×10^{-4}		6.91×10^{-3}
2.20×10^{-5}	3.38×10^{-4}	3.38×10^{-4}	3.72×10^{-4}	1.06×10^{-2}
2.20×10^{-5}	4.50×10^{-4}	4.50×10^{-4}		1.48×10^{-2}
2.20×10^{-5}	5.65×10^{-4}	5.65×10^{-4}	6.20×10^{-4}	1.78×10^{-2}
2.20×10^{-5}	6.75×10^{-4}	6.75×10^{-4}		2.17×10^{-2}

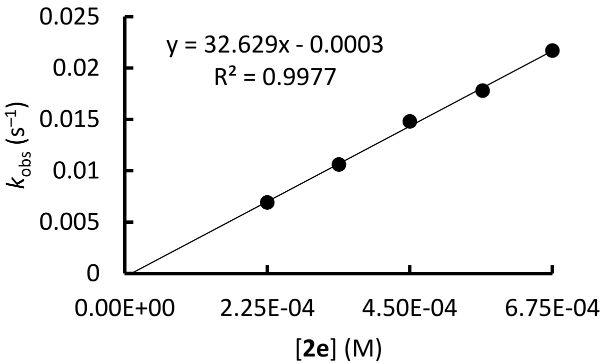

$\boldsymbol{k_2 = (3.26 \pm 0.09) \times 10^{1}\ M^{-1}\ s^{-1}}$

1g + **2g** in DMSO (stopped-flow, detection at 359 nm) CG113

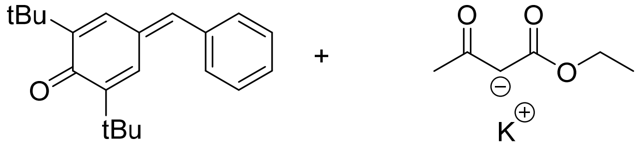

1g **2g**

[**1g**]$_0$ (M)	[**2g**]$_0$ (M)	[**2g**-H]$_0$ (M)	[18-c-6]$_0$ (M)	k_{obs} (s^{-1})
1.54×10^{-5}	2.25×10^{-4}	2.25×10^{-4}		4.50×10^{-2}
1.54×10^{-5}	3.38×10^{-4}	3.38×10^{-4}	3.72×10^{-4}	6.54×10^{-2}
1.54×10^{-5}	4.50×10^{-4}	4.50×10^{-4}		8.98×10^{-2}
1.54×10^{-5}	5.65×10^{-4}	5.65×10^{-4}	6.20×10^{-4}	1.09×10^{-1}
1.54×10^{-5}	6.75×10^{-4}	6.75×10^{-4}		1.32×10^{-1}

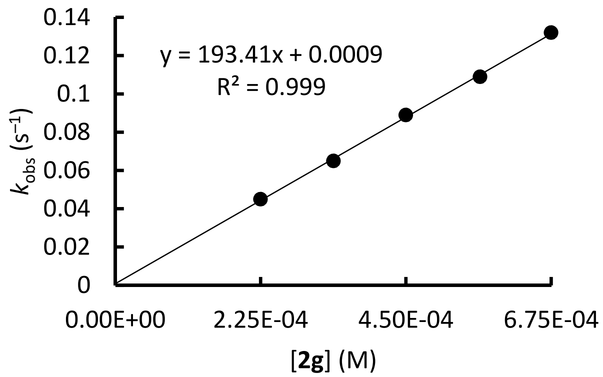

$\boldsymbol{k_2}$ **= (1.93 ± 0.04) × 10^2 M^{-1} s^{-1}**

1g + **2h** in DMSO (stopped-flow, detection at 359 nm) CG109

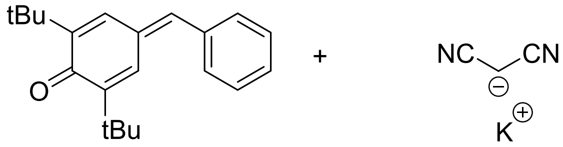

1g **2h**

[**1g**]$_0$ (M)	[**2h**] (M)	[**2h**-H]$_0$ (M)	[18-c-6]$_0$ (M)	k_{obs} (s^{-1})
1.79×10^{-5}	2.25×10^{-4}	2.25×10^{-4}		5.65×10^{-2}
1.79×10^{-5}	3.38×10^{-4}	3.38×10^{-4}	3.72×10^{-4}	7.99×10^{-2}
1.79×10^{-5}	4.50×10^{-4}	4.50×10^{-4}		1.13×10^{-1}
1.79×10^{-5}	6.75×10^{-4}	6.75×10^{-4}		1.76×10^{-1}

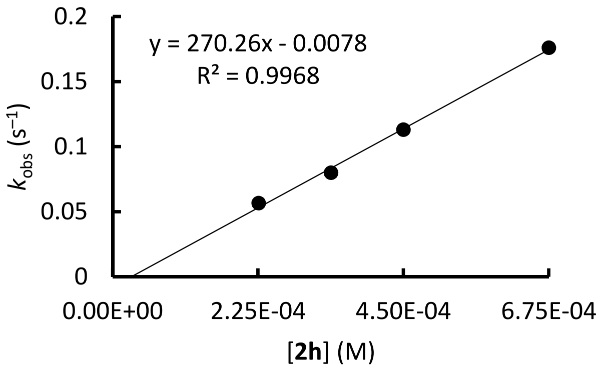

$\boldsymbol{k_2}$ **= (2.70 ± 0.11) × 10^2 M^{-1} s^{-1}**

1g + **2i** in DMSO (stopped-flow, detection at 359 nm) CG111

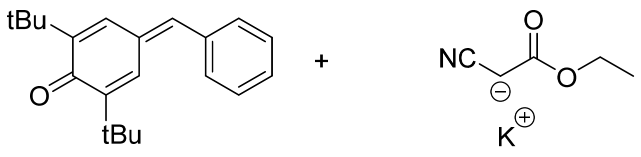

1g **2i**

[**1g**]$_0$ (M)	[**2i**]$_0$ (M)	[**2i**-H]$_0$ (M)	[18-c-6]$_0$ (M)	k_{obs} (s^{-1})
1.82×10^{-5}	2.25×10^{-4}	2.25×10^{-4}		1.24×10^{-1}
1.82×10^{-5}	3.38×10^{-4}	3.38×10^{-4}	3.72×10^{-4}	1.80×10^{-1}
1.82×10^{-5}	4.50×10^{-4}	4.50×10^{-4}		2.49×10^{-1}
1.82×10^{-5}	5.65×10^{-4}	5.65×10^{-4}	6.20×10^{-4}	3.01×10^{-1}
1.82×10^{-5}	6.75×10^{-4}	6.75×10^{-4}		3.77×10^{-1}

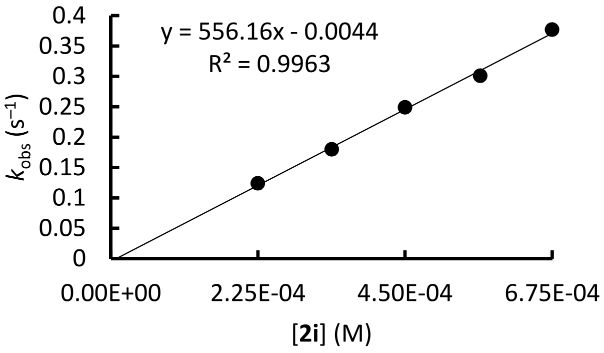

$k_2 = (5.56 \pm 0.20) \times 10^2\ M^{-1}\ s^{-1}$

1g + **2j** in DMSO (stopped-flow, detection at 359 nm) CG112

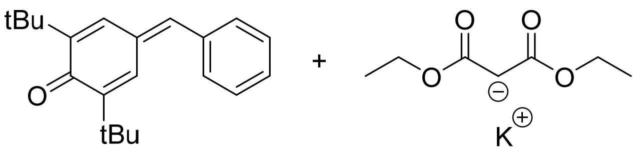

1g **2j**

[**1g**]$_0$ (M)	[**2j**]$_0$ (M)	[**2j**-H]$_0$ (M)	[18-c-6]$_0$ (M)	k_{obs} (s^{-1})
1.73×10^{-5}	2.25×10^{-4}	2.25×10^{-4}		3.51×10^{-1}
1.73×10^{-5}	3.38×10^{-4}	3.38×10^{-4}	3.72×10^{-4}	4.87×10^{-1}
1.73×10^{-5}	4.50×10^{-4}	4.50×10^{-4}		6.62×10^{-1}
1.73×10^{-5}	5.65×10^{-4}	5.65×10^{-4}	6.20×10^{-4}	8.03×10^{-1}
1.73×10^{-5}	6.75×10^{-4}	6.75×10^{-4}		9.67×10^{-1}

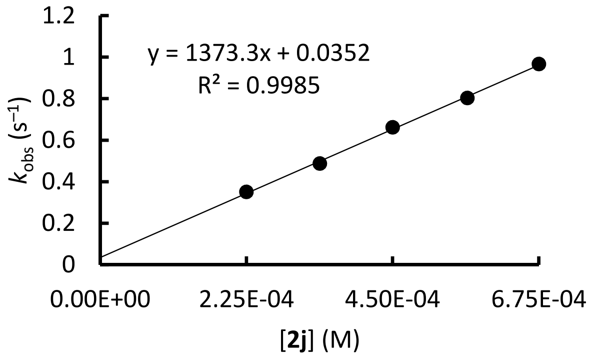

$k_2 = (1.37 \pm 0.03) \times 10^3\ M^{-1}\ s^{-1}$

9. Electrophilicities *E* of *p*QMs **1a–1g**

The slopes of the correlation lines in Figs. S2–S7 are set to unity as required by Equation (1) and illustrate the results from least-square minimizations $\Delta^2 = (\lg k_2^{exp} - s_N(N + E))^2$ to calculate the electrophilicity *E* of the *p*QMs. The least-squares minimizations used the experimental second-order rate constants k_2^{exp} in DMSO at 20 °C (this Supporting Information, Section 8) and the reported nucleophile-specific parameters *N* and s_N of the reference nucleophiles **2a**–**2i** from ref. [20] as input in an MS Excel spreadsheet. The *E* parameter for each individual *o*QM was defined as an adjustable variable and optimized by using the MS Excel Solver (GRG algorithm).

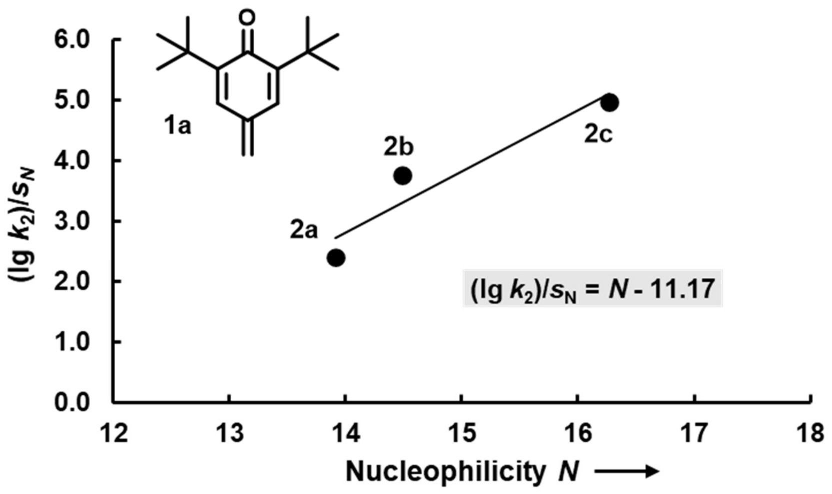

Figure S2. Correlation of lg k_2^{exp}/s_N vs. *N* for *p*QM **1a**.

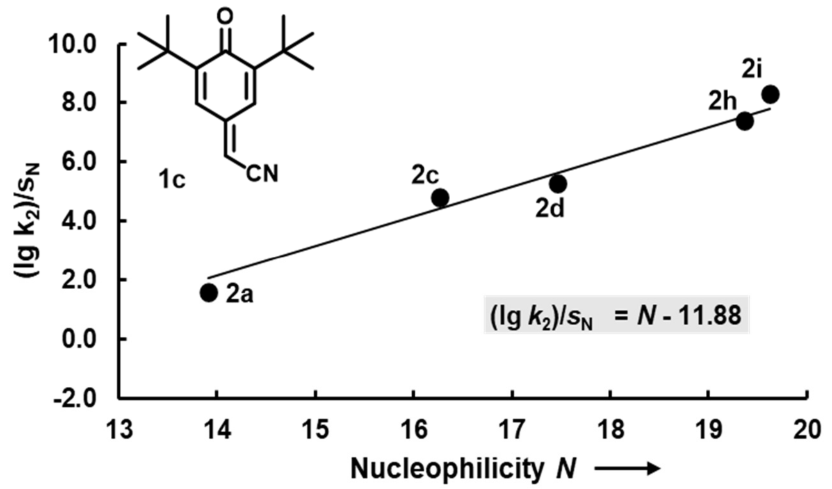

Figure S3. Correlation of lg k_2^{exp}/s_N vs. *N* for *p*QM **1c**.

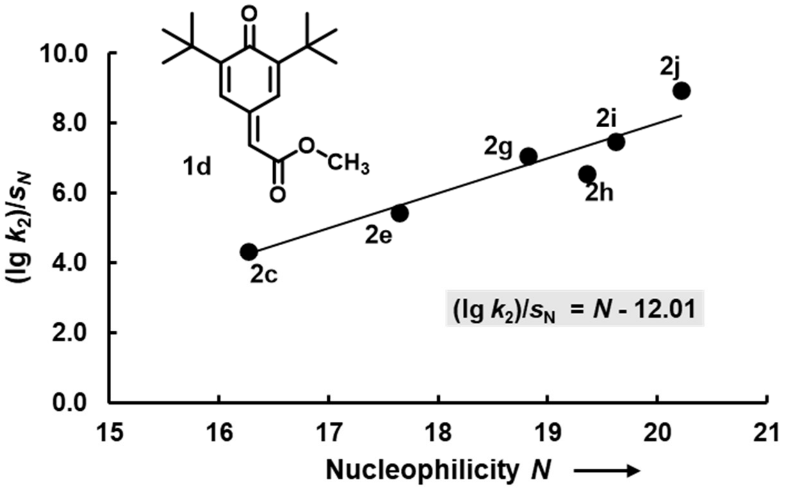

Figure S4. Correlation of lg k_2^{exp}/s_N vs. N for pQM **1d**.

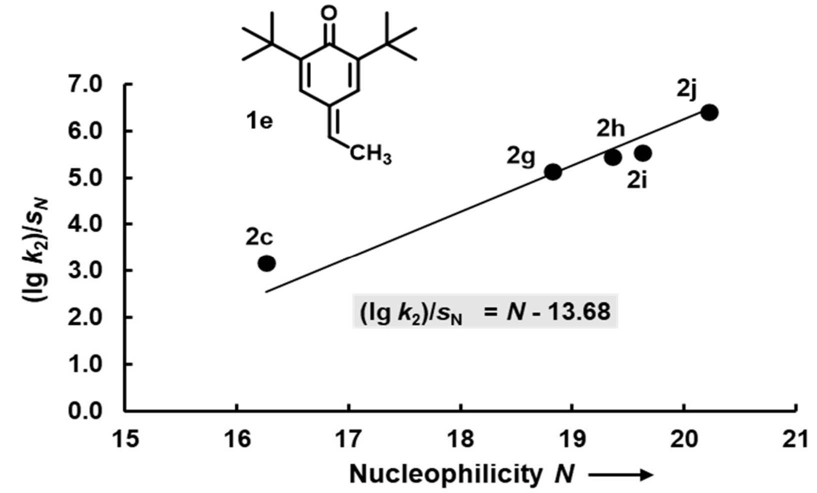

Figure S5. Correlation of lg k_2^{exp}/s_N vs. N for pQM **1e**.

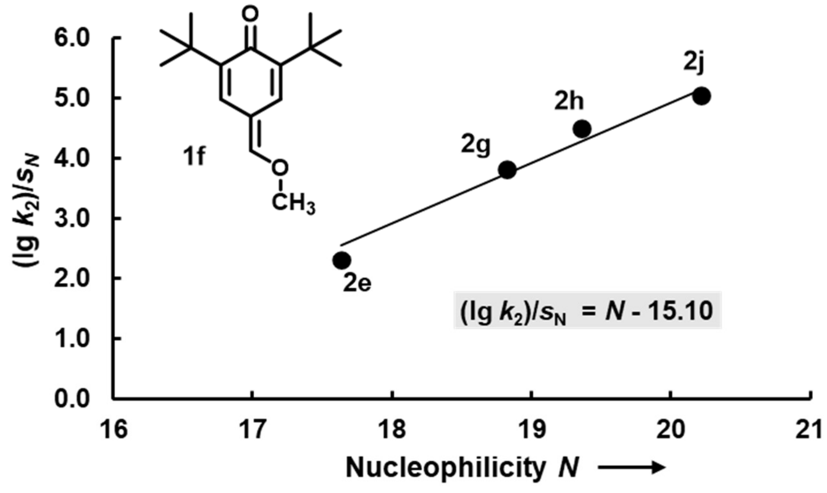

Figure S6. Correlation of lg k_2^{exp}/s_N vs. N for pQM **1f**.

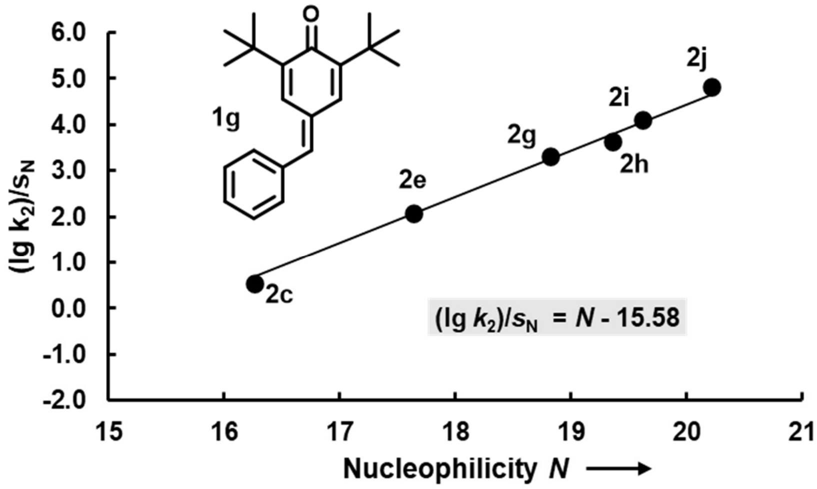

Figure S7. Correlation of lg k_2^{exp}/s_N vs. N for *p*QM **1g**.

10. Copies of ^{1}H and $^{13}C\{^{1}H\}$ NMR Spectra

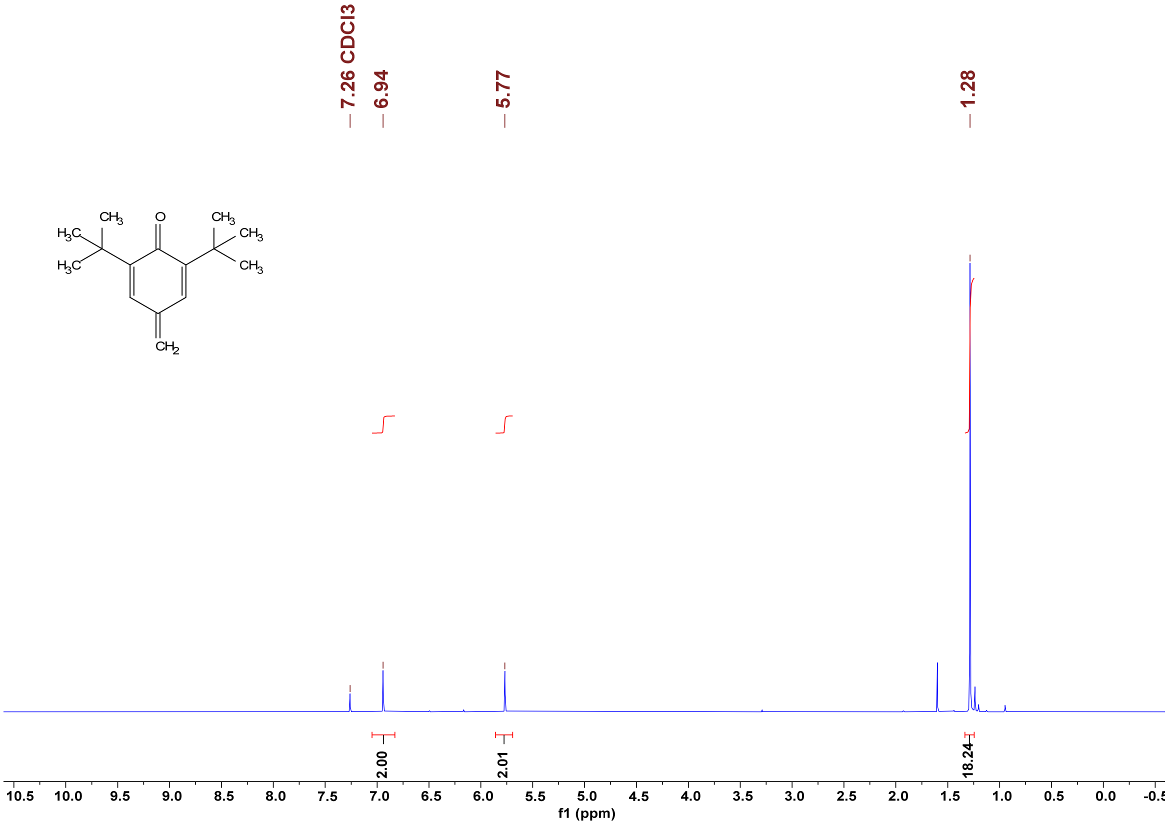

Figure S8. ^{1}H NMR spectrum of **1a** in $CDCl_3$ (400 MHz) CG446_CDCl3

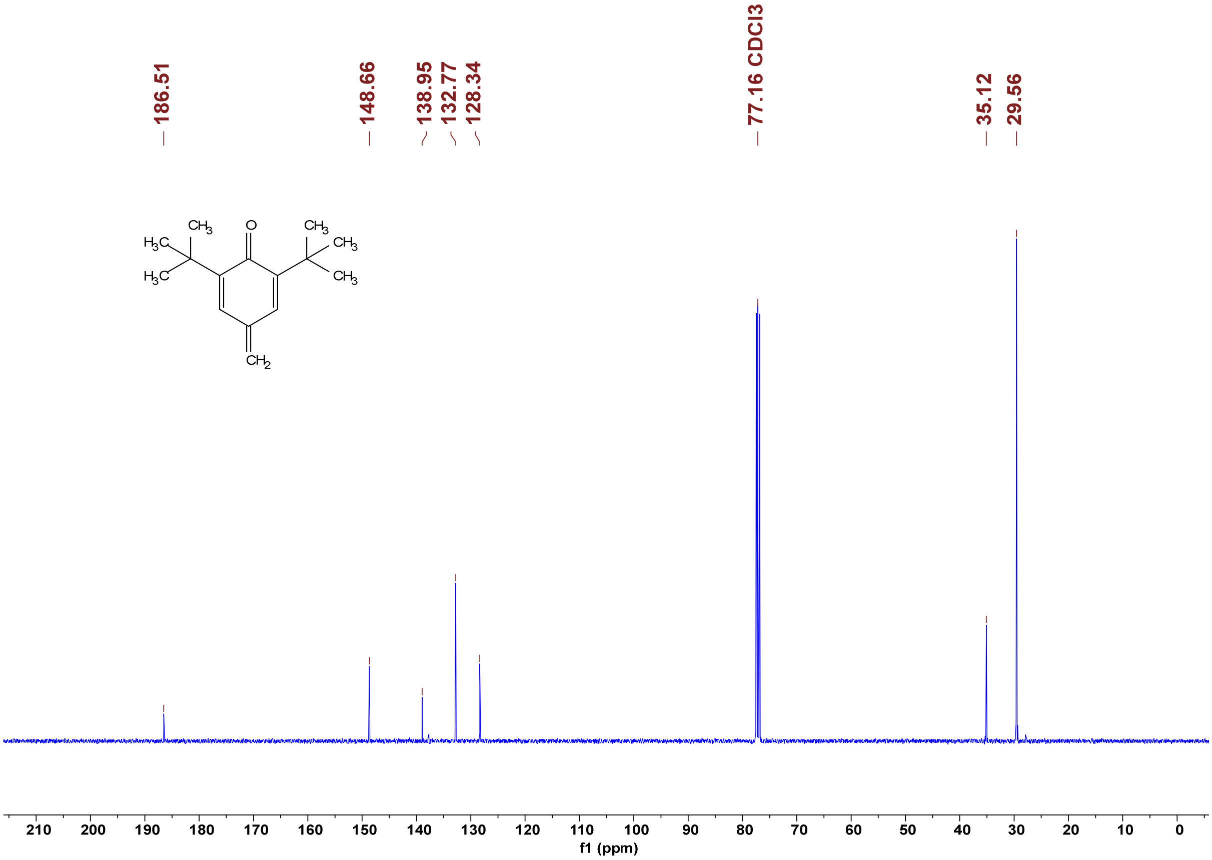

Figure S9. ^{13}C NMR spectrum of **1a** in $CDCl_3$ (101 MHz) CG446_CDCl3

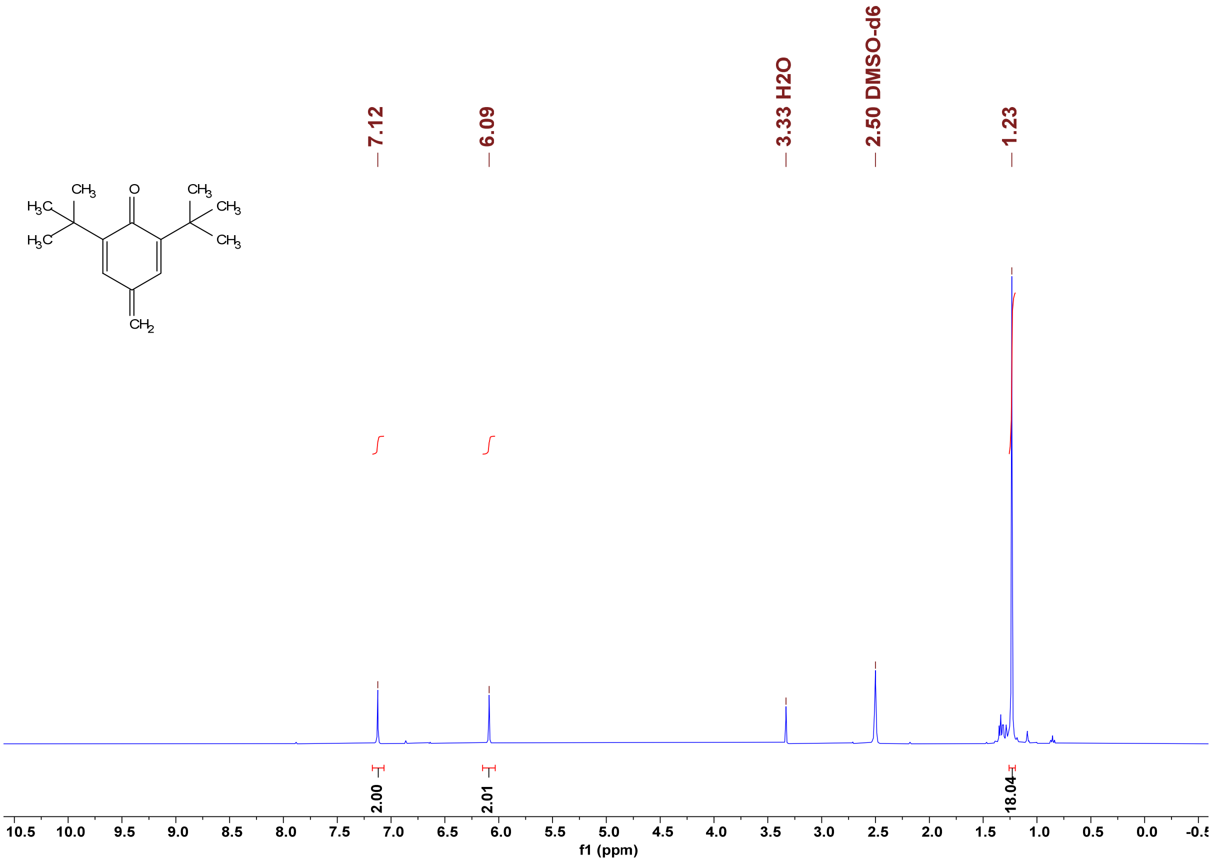

Figure S10. ^{1}H NMR spectrum of **1a** in DMSO-d_6 (400 MHz) CG446_n_Pentan

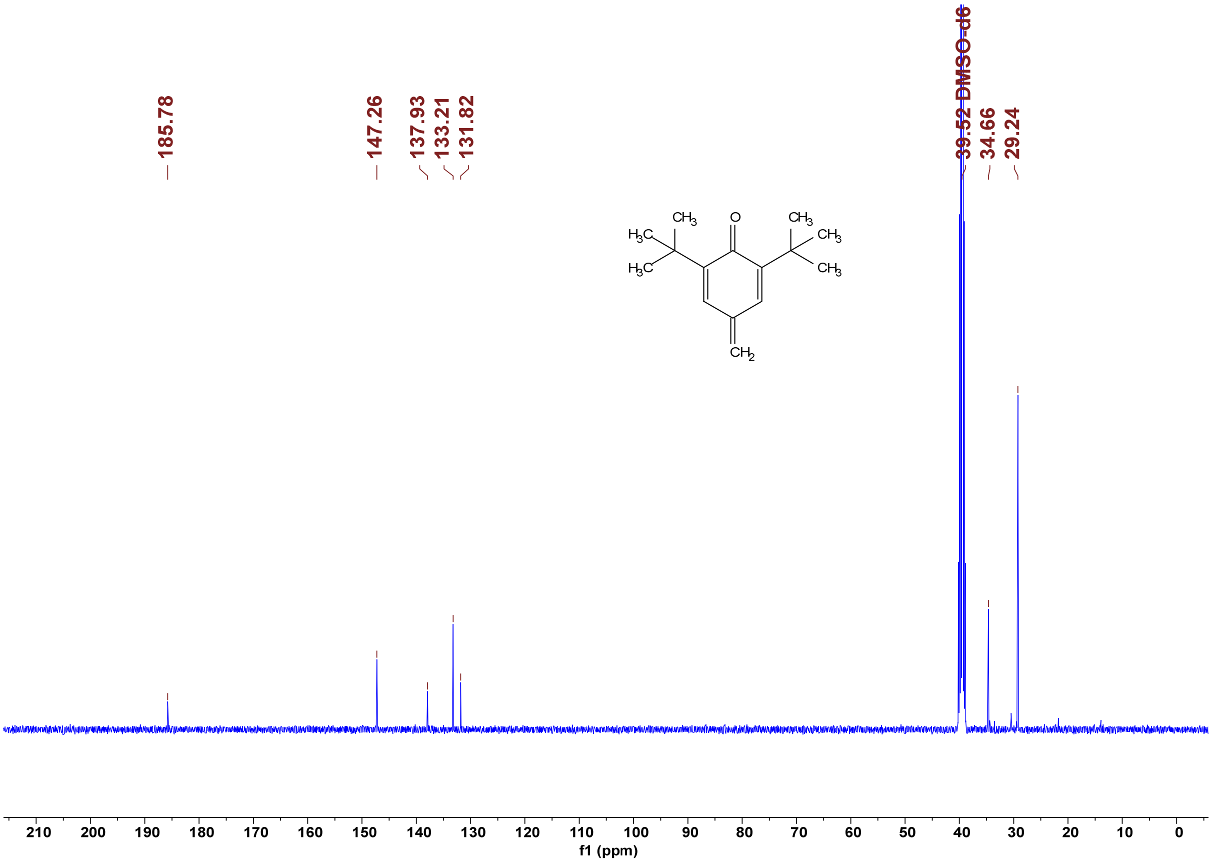

Figure S11. ^{13}C NMR spectrum of **1a** in DMSO-d_6 (101 MHz) CG446_n_Pentan

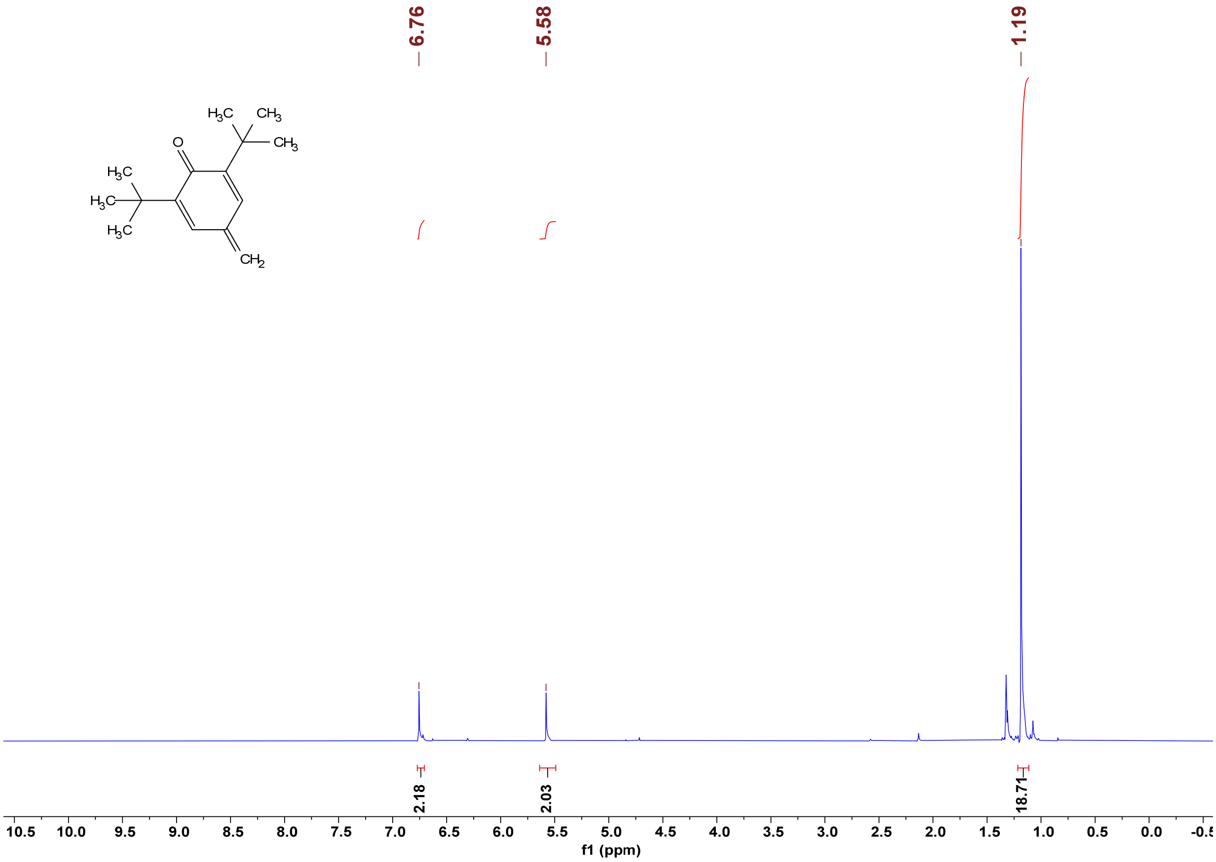

Figure S12. ^{1}H NMR spectrum of **1a** in CCl_4 (400 MHz) CG446_1

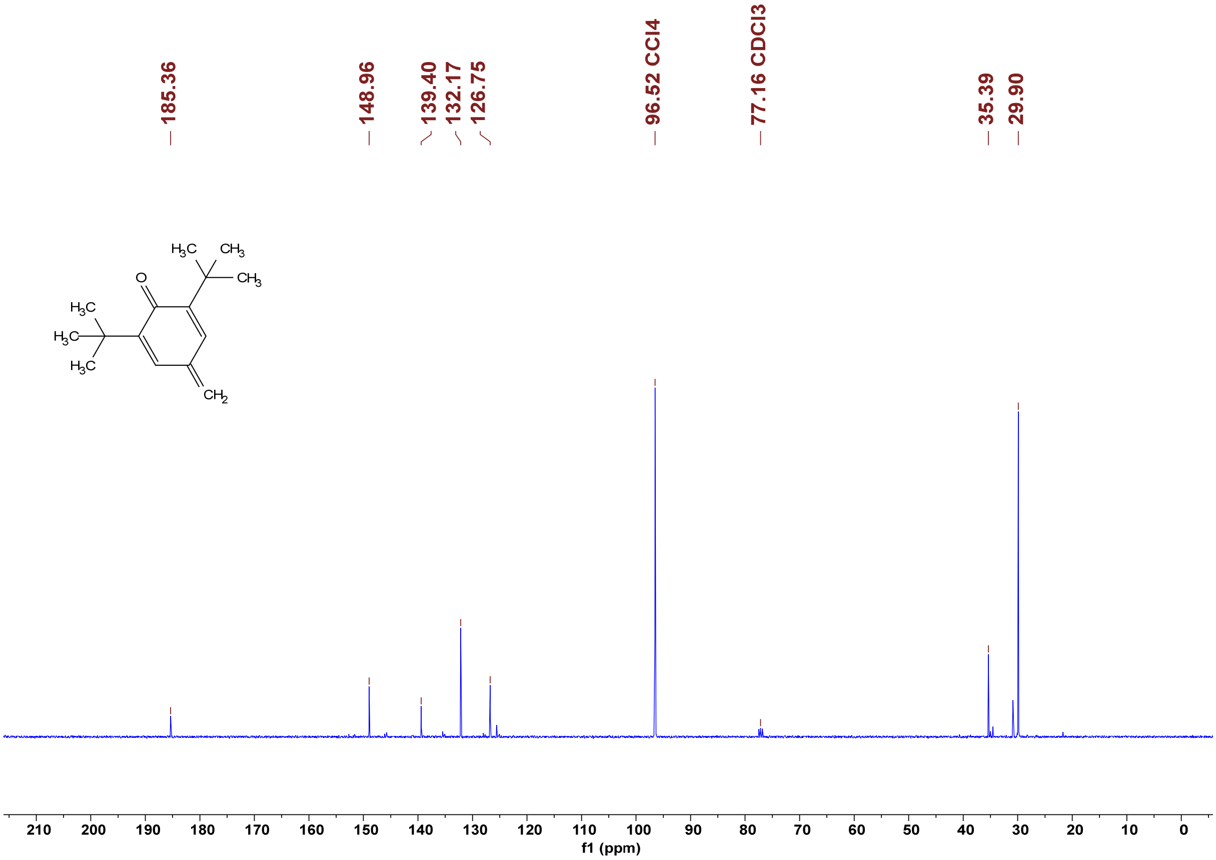

Figure S13. ^{13}C NMR spectrum of **1a** in $CDCl_3$ (101 MHz) CG446_1

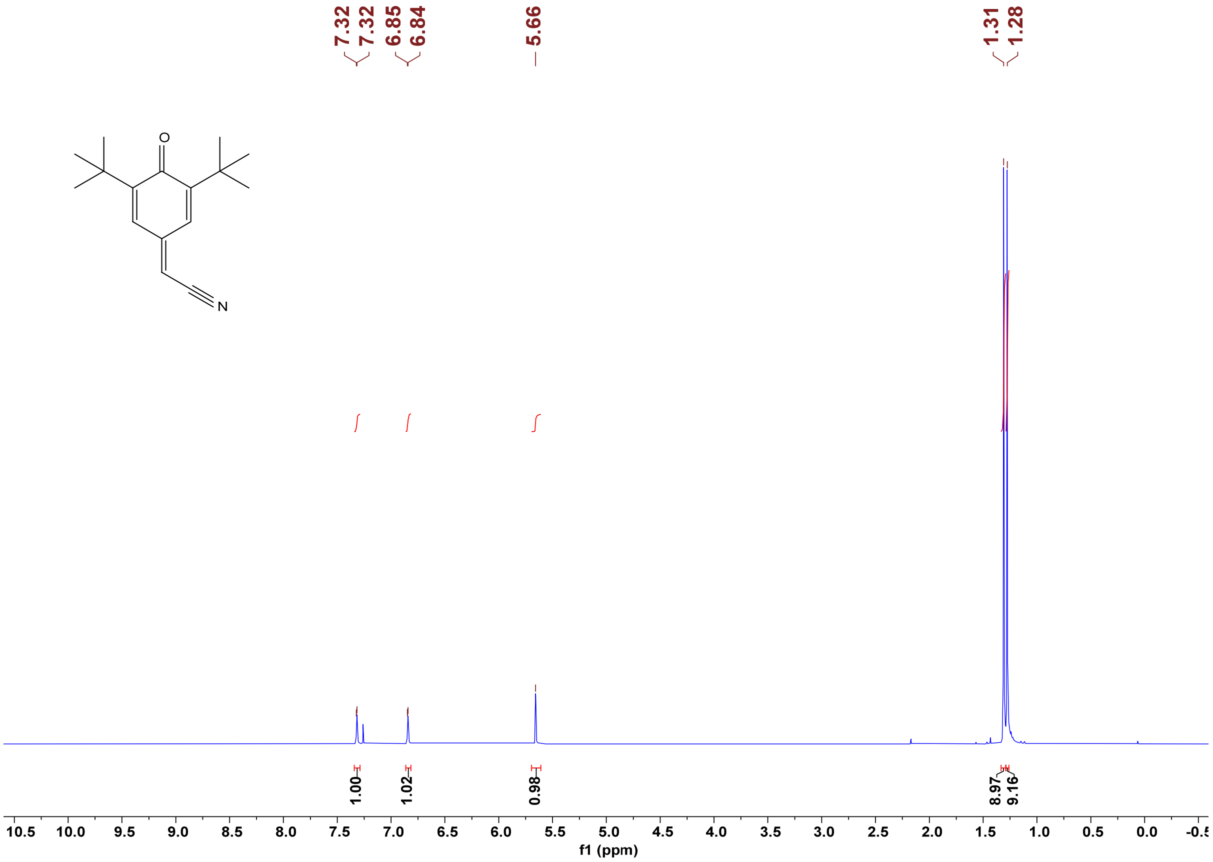

Figure S14. ^{1}H NMR spectrum of **1c** in $CDCl_3$ (400 MHz) CG067

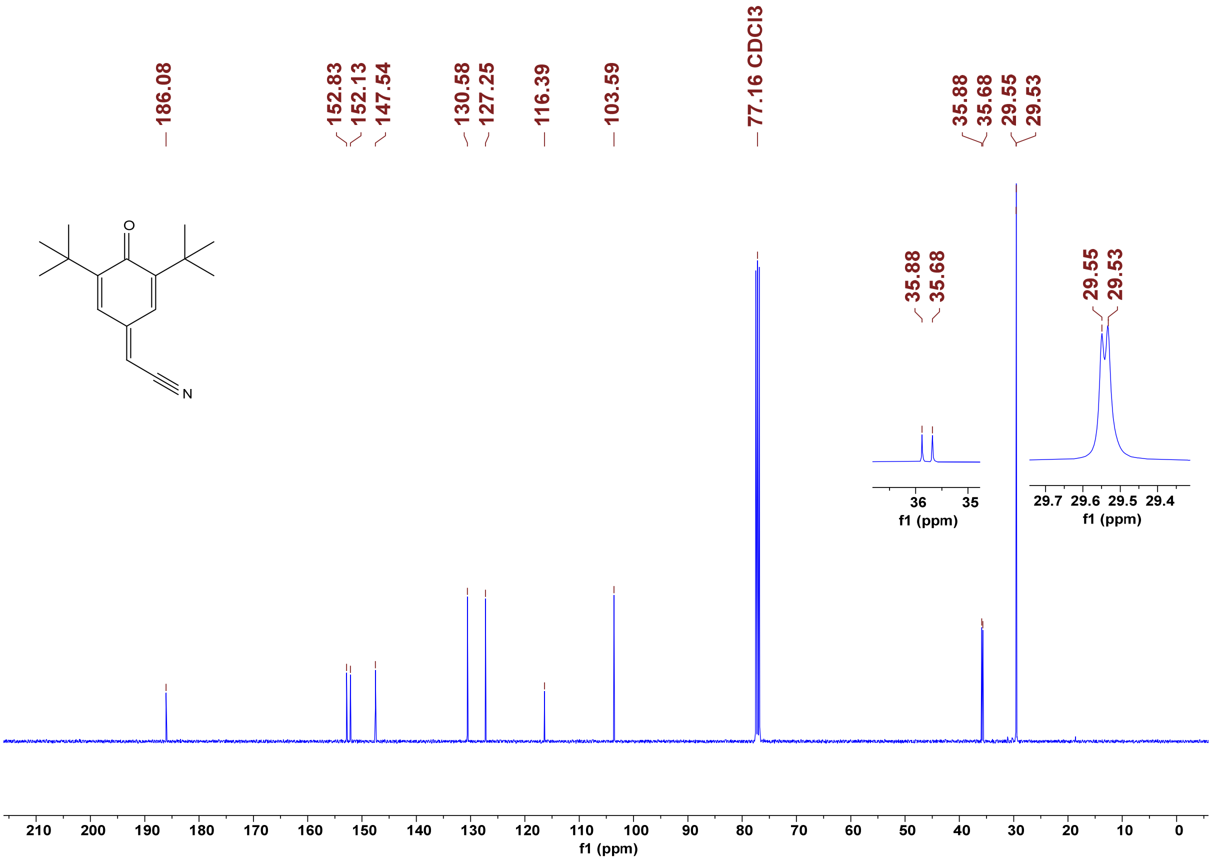

Figure S15. ^{13}C NMR spectrum of **1c** in $CDCl_3$ (101 MHz) CG067

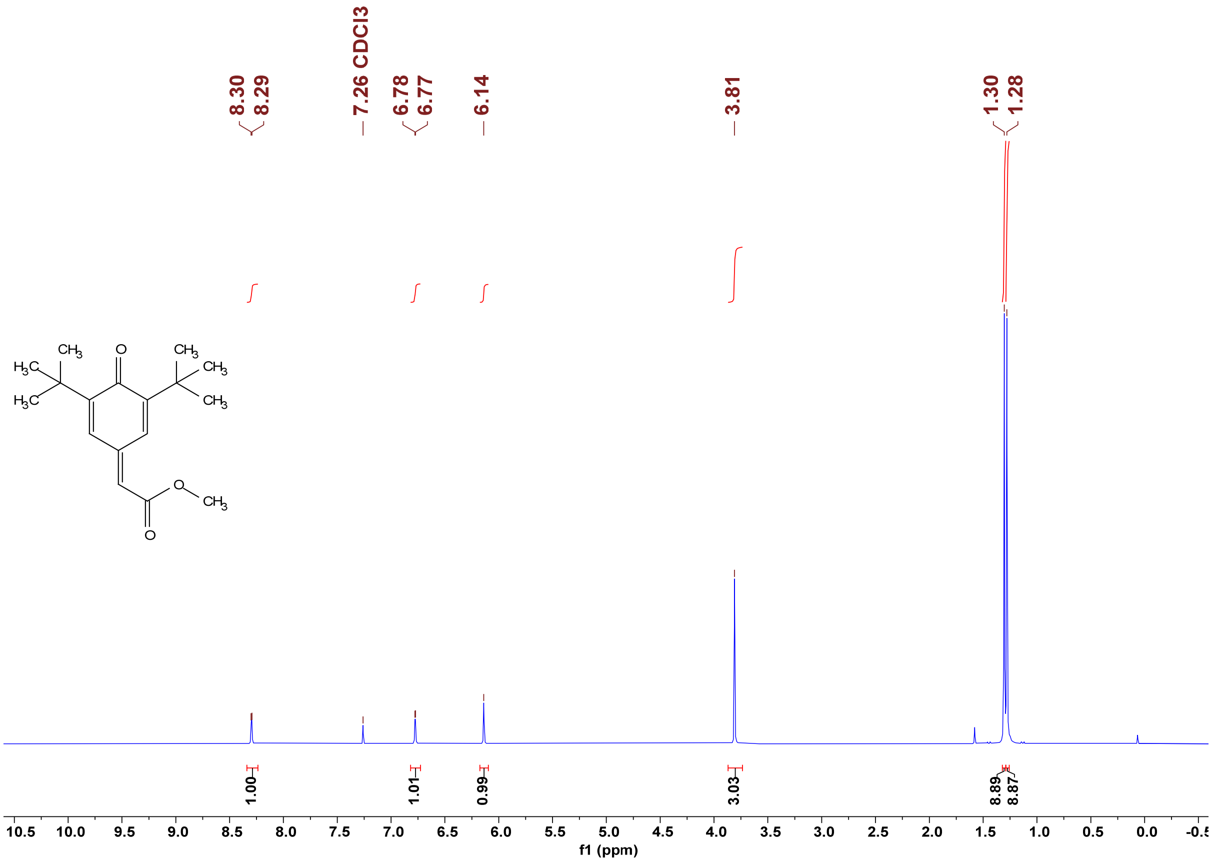

Figure S16. ^{1}H NMR spectrum of **1d** in $CDCl_3$ (400 MHz) CG475F1

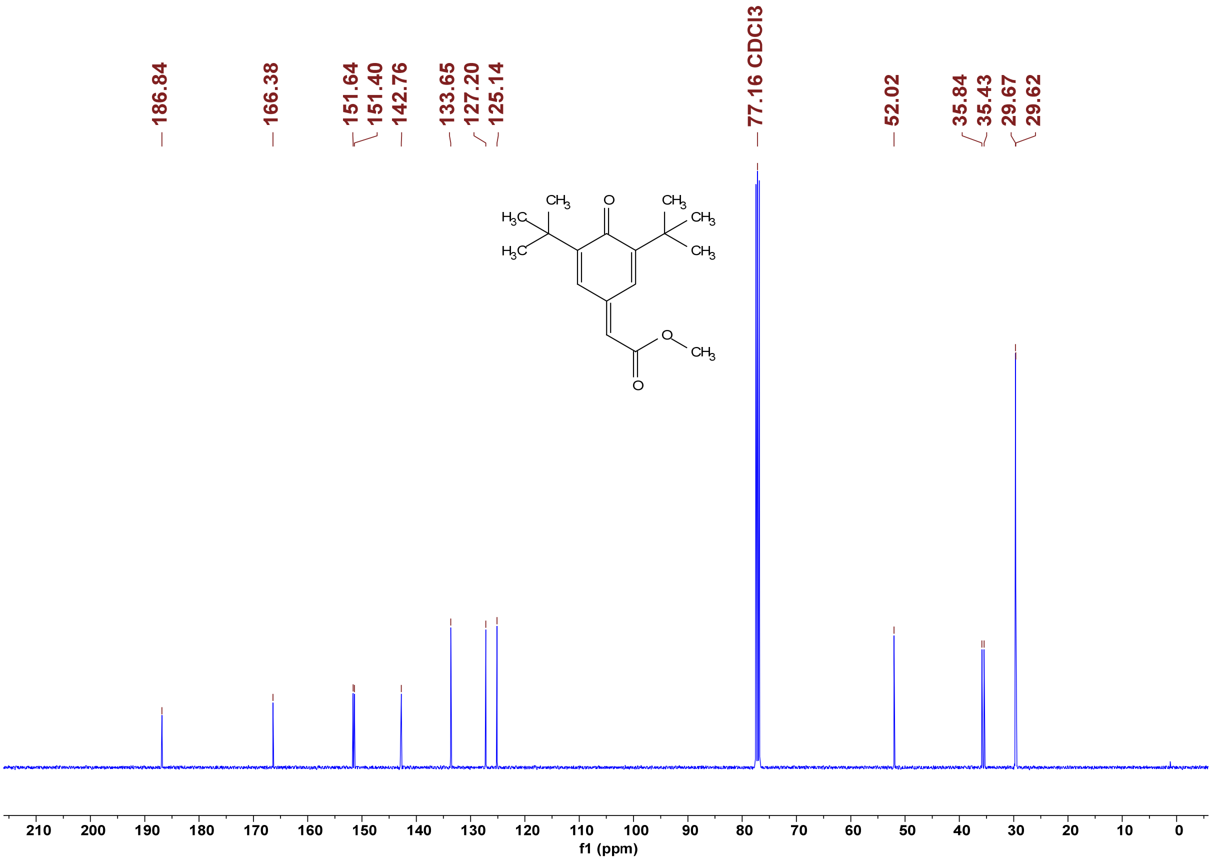

Figure S17. ^{13}C NMR spectrum of **1d** in $CDCl_3$ (101 MHz) CG475F1

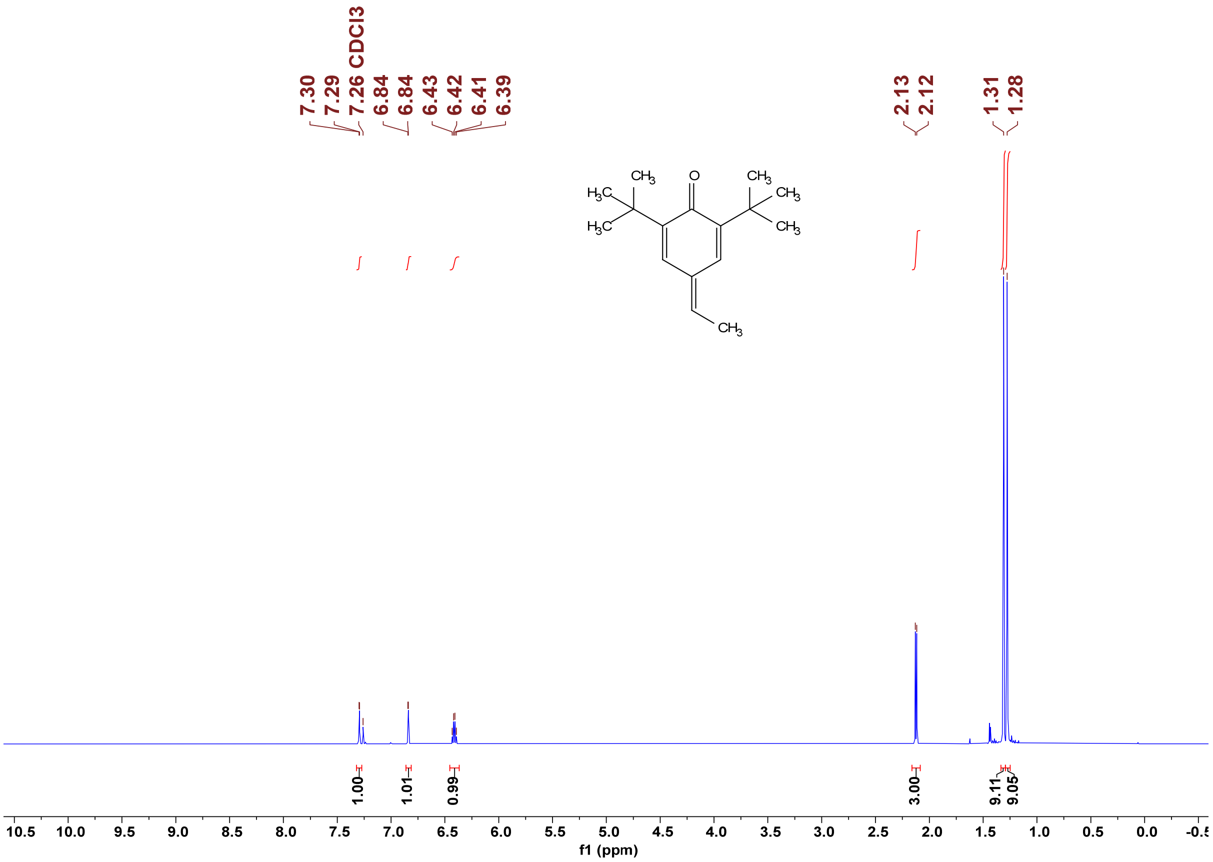

Figure S18. ^{1}H NMR spectrum of **1e** in $CDCl_3$ (600 MHz) TR01

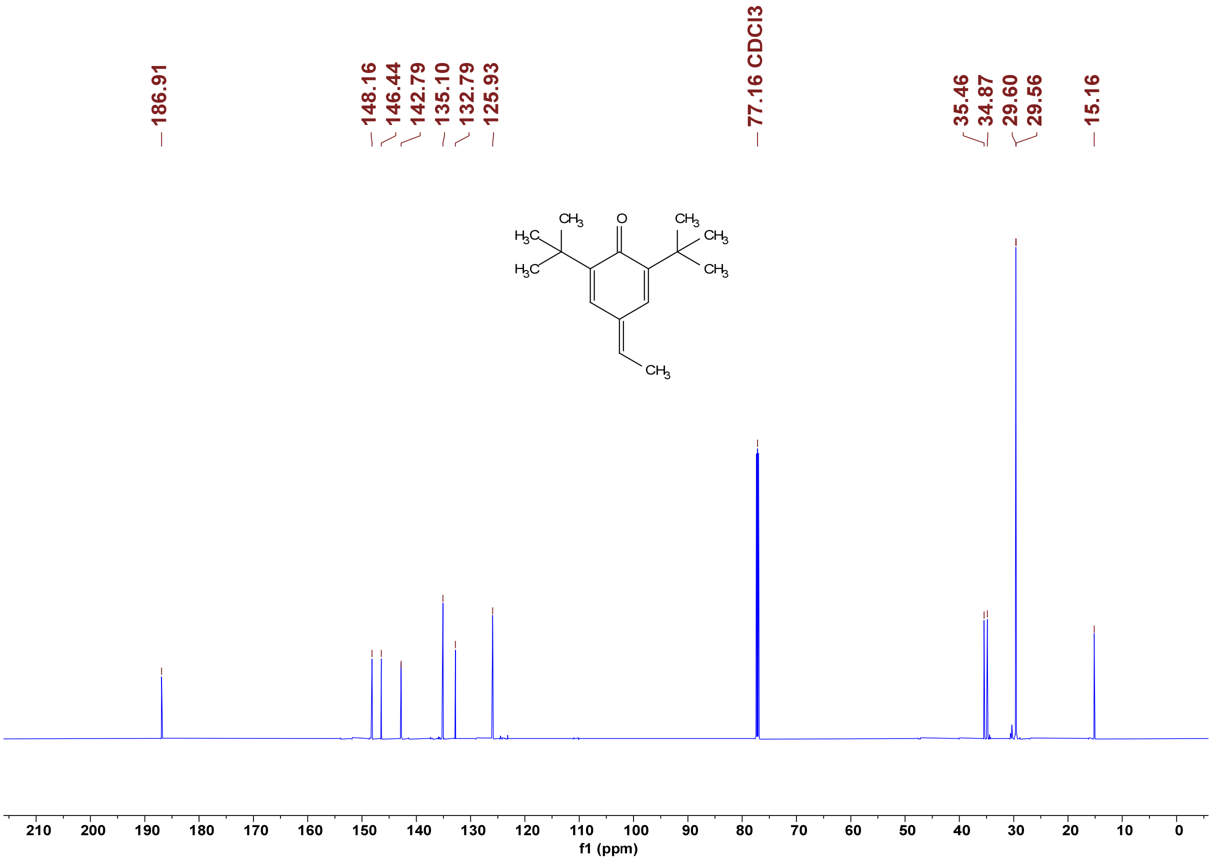

Figure S19. ^{13}C NMR spectrum of **1e** in $CDCl_3$ (151 MHz) TR01

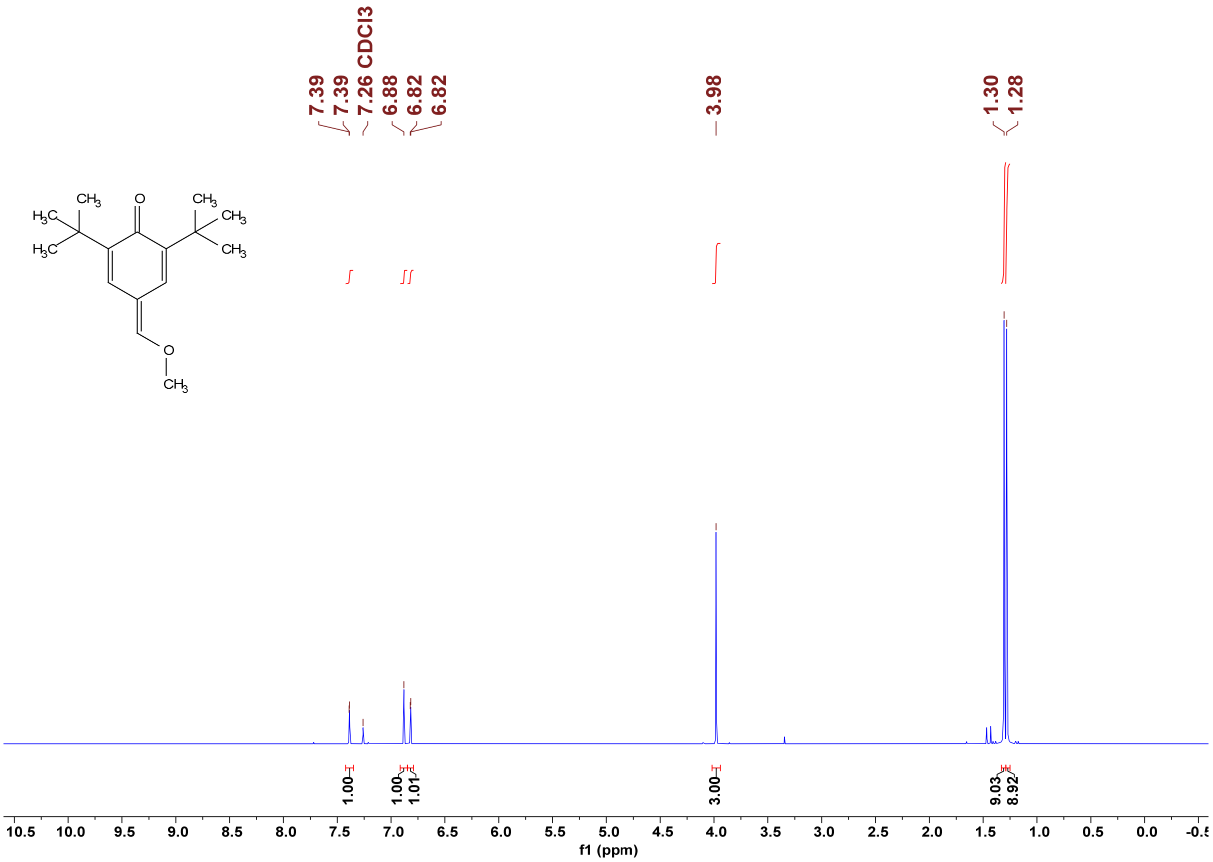

Figure S20. ^{1}H NMR spectrum of **1f** in $CDCl_3$ (600 MHz) CG476_CDCl3

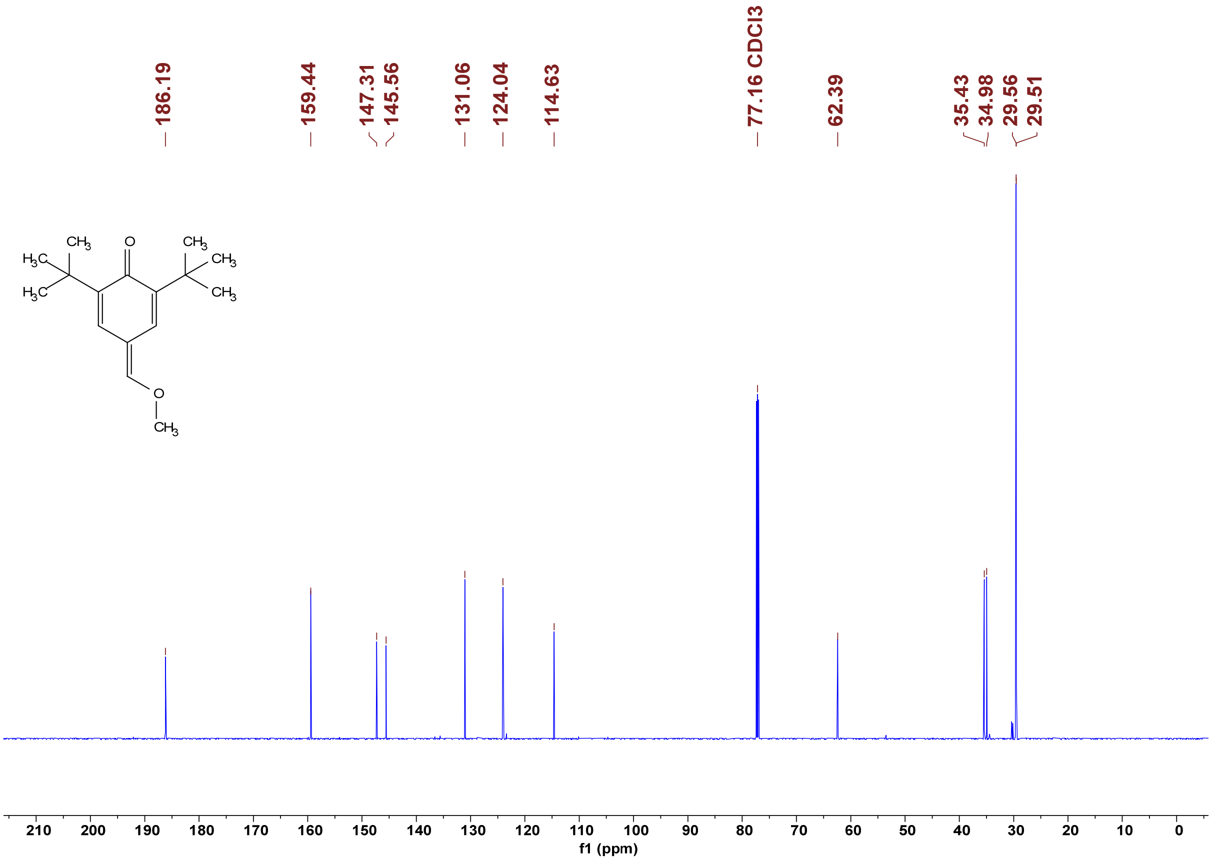

Figure S21. ^{13}C NMR spectrum of **1f** in $CDCl_3$ (151 MHz) CG476_CDCl3

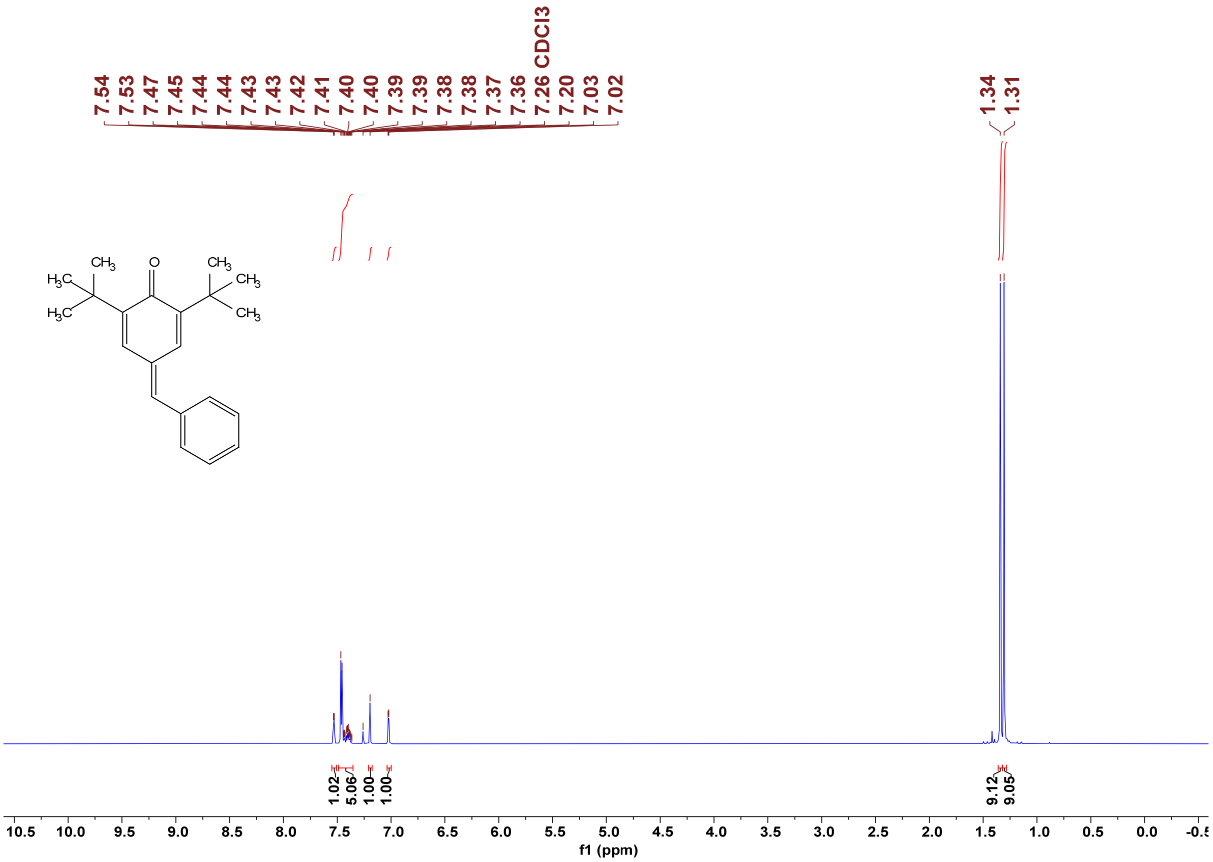

Figure S22. ^{1}H NMR spectrum of **1g** in $CDCl_3$ (400 MHz) CG095

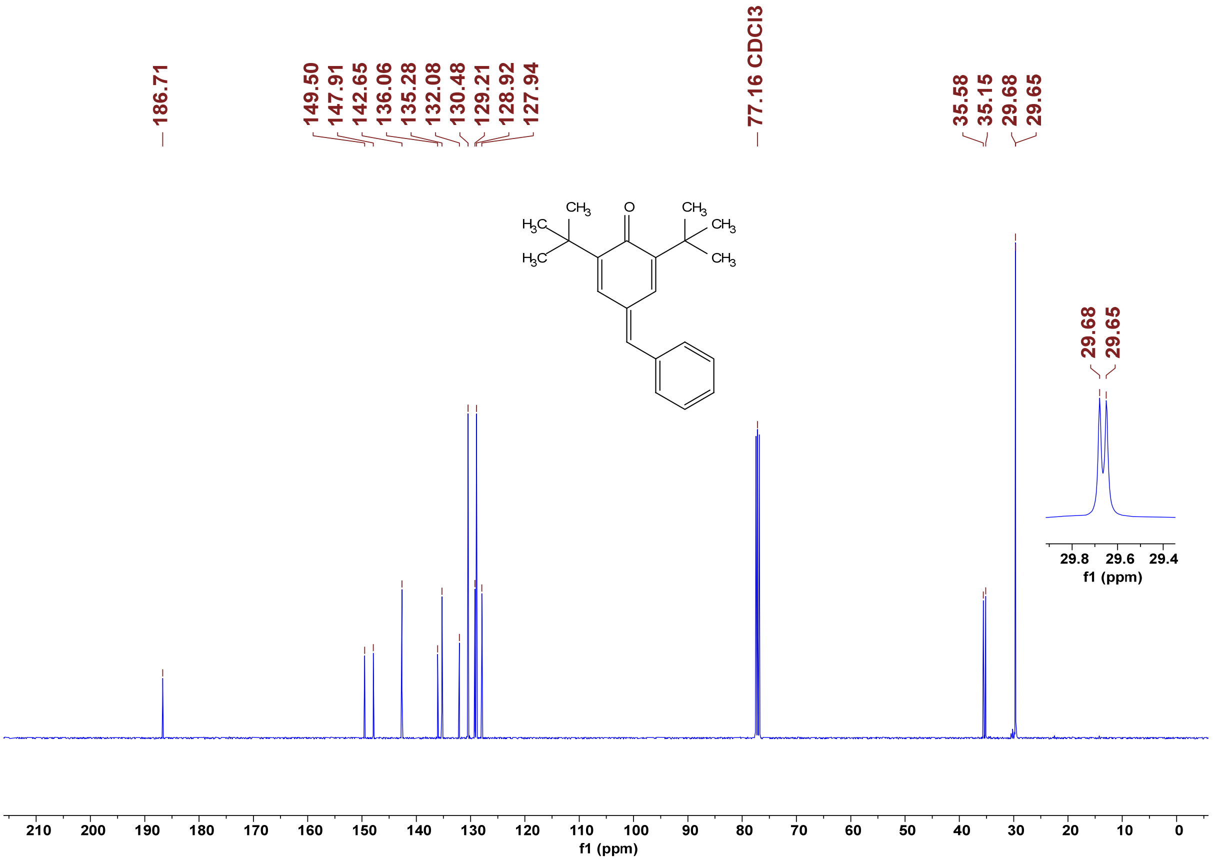

Figure S23. ^{13}C NMR spectrum of **1g** in $CDCl_3$ (101 MHz) CG095

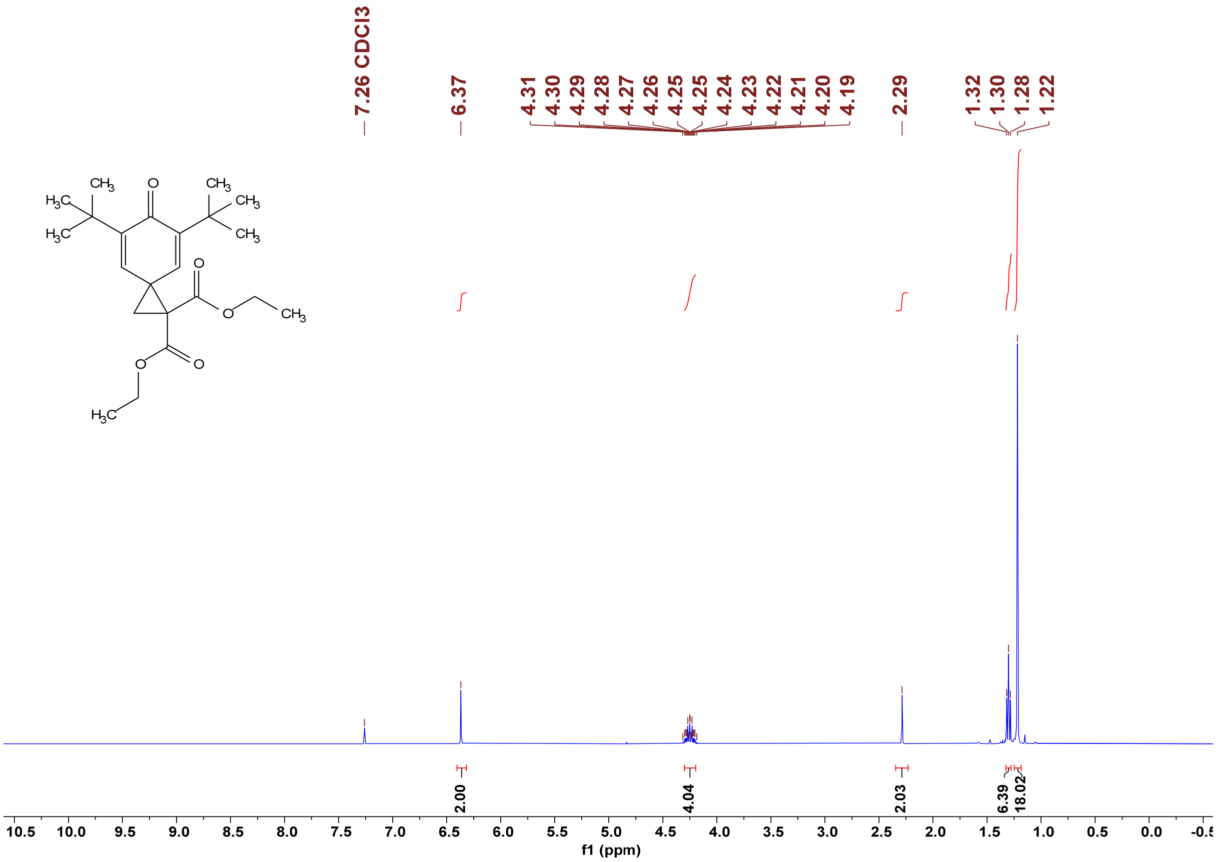

Figure S24. ^{1}H NMR spectrum of **3** in $CDCl_3$ (400 MHz) CG536_1F20-29

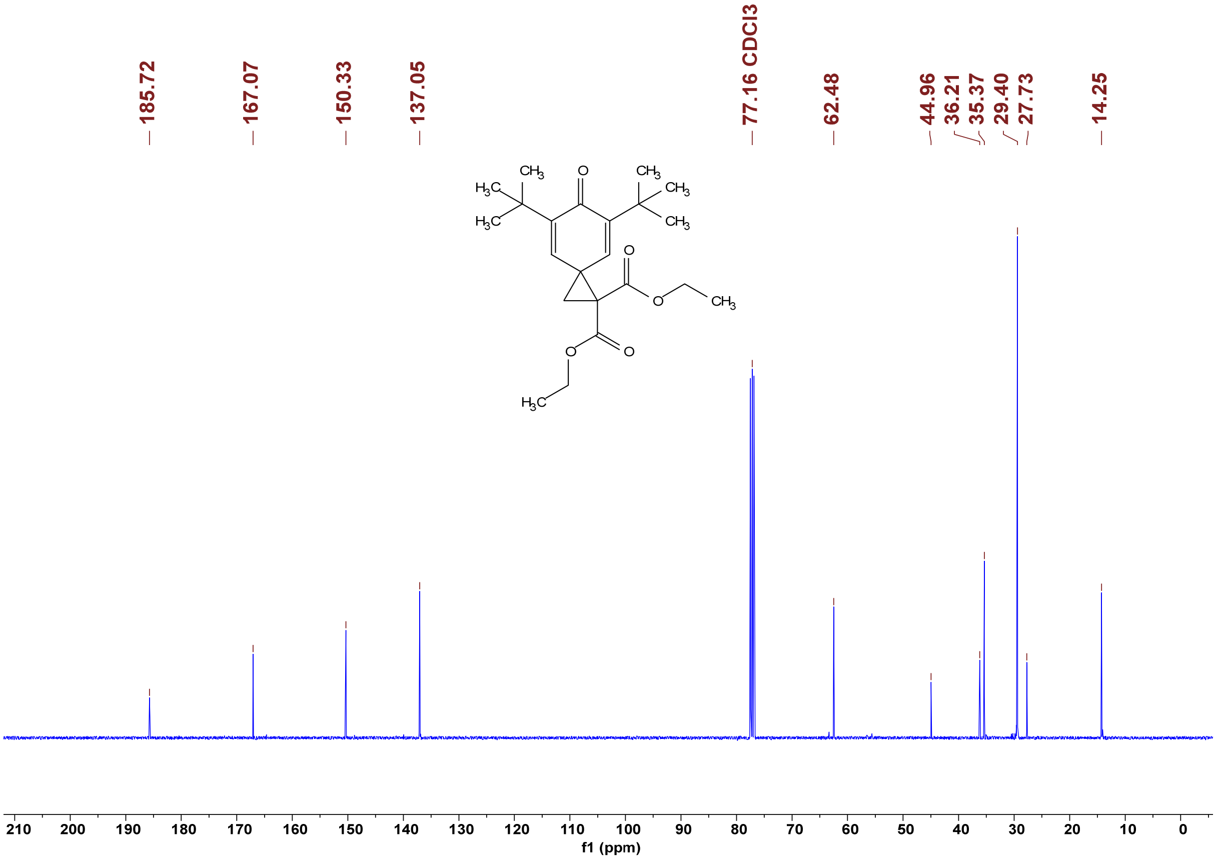

Figure S25. ^{13}C NMR spectrum of **3** in $CDCl_3$ (101 MHz) CG536_1F20-29

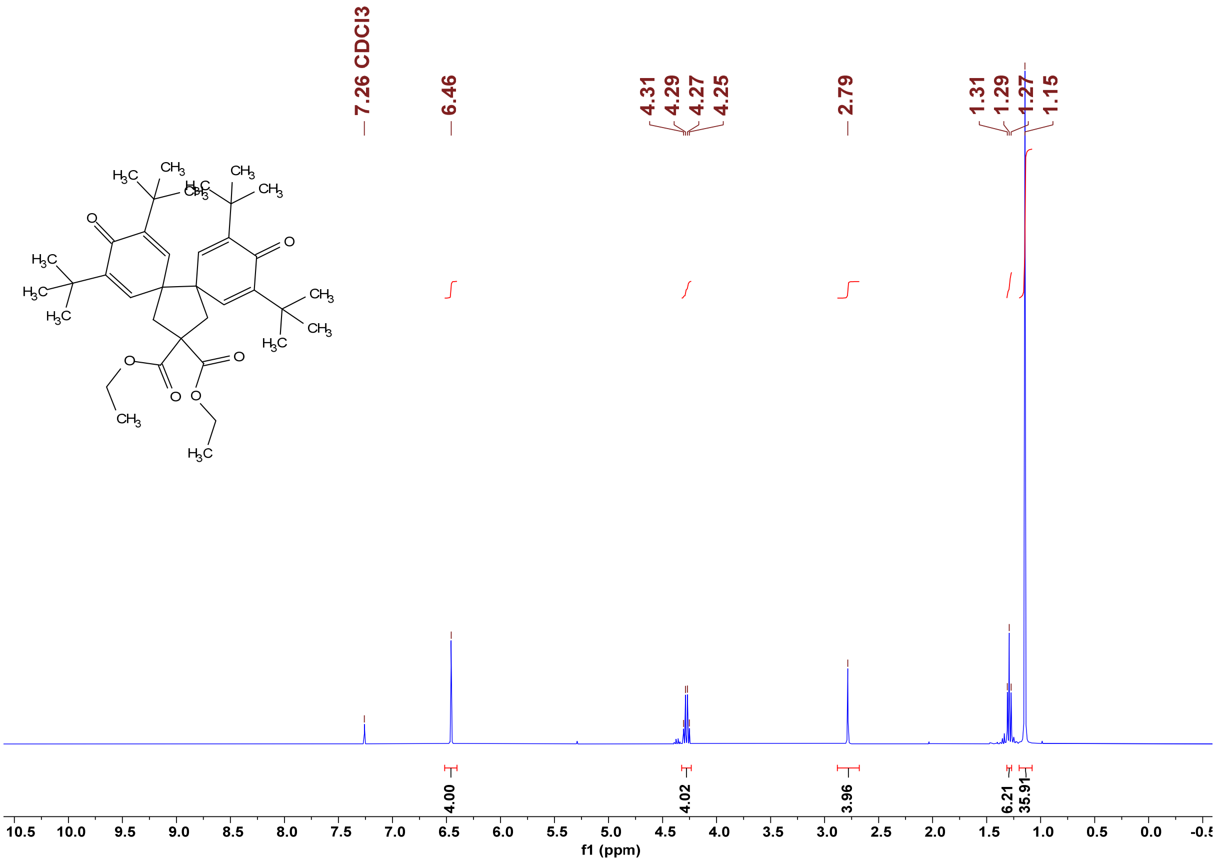

Figure S26. ^{1}H NMR spectrum of **4** in $CDCl_3$ (400 MHz) CG536_1F10-16

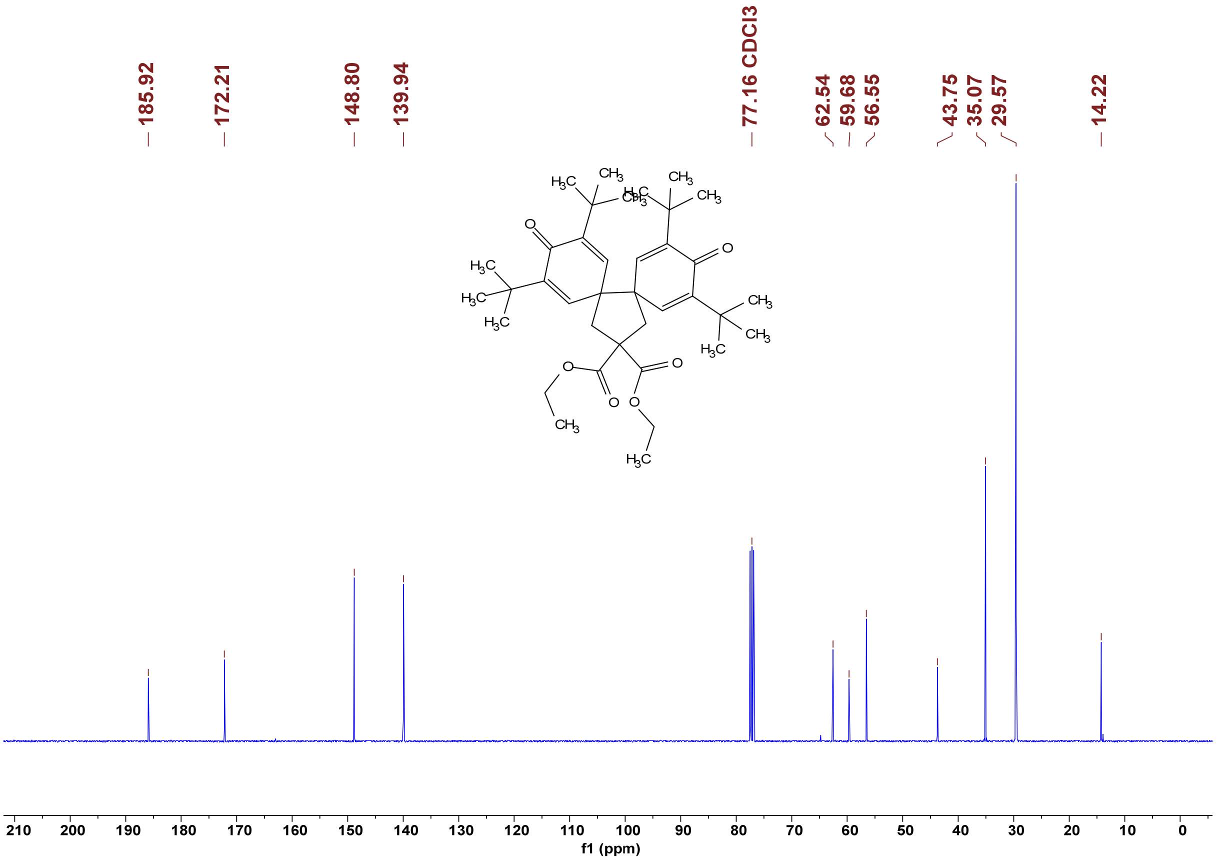

Figure S27. ^{13}C NMR spectrum of **4** in $CDCl_3$ (101 MHz) CG536_1F10-16

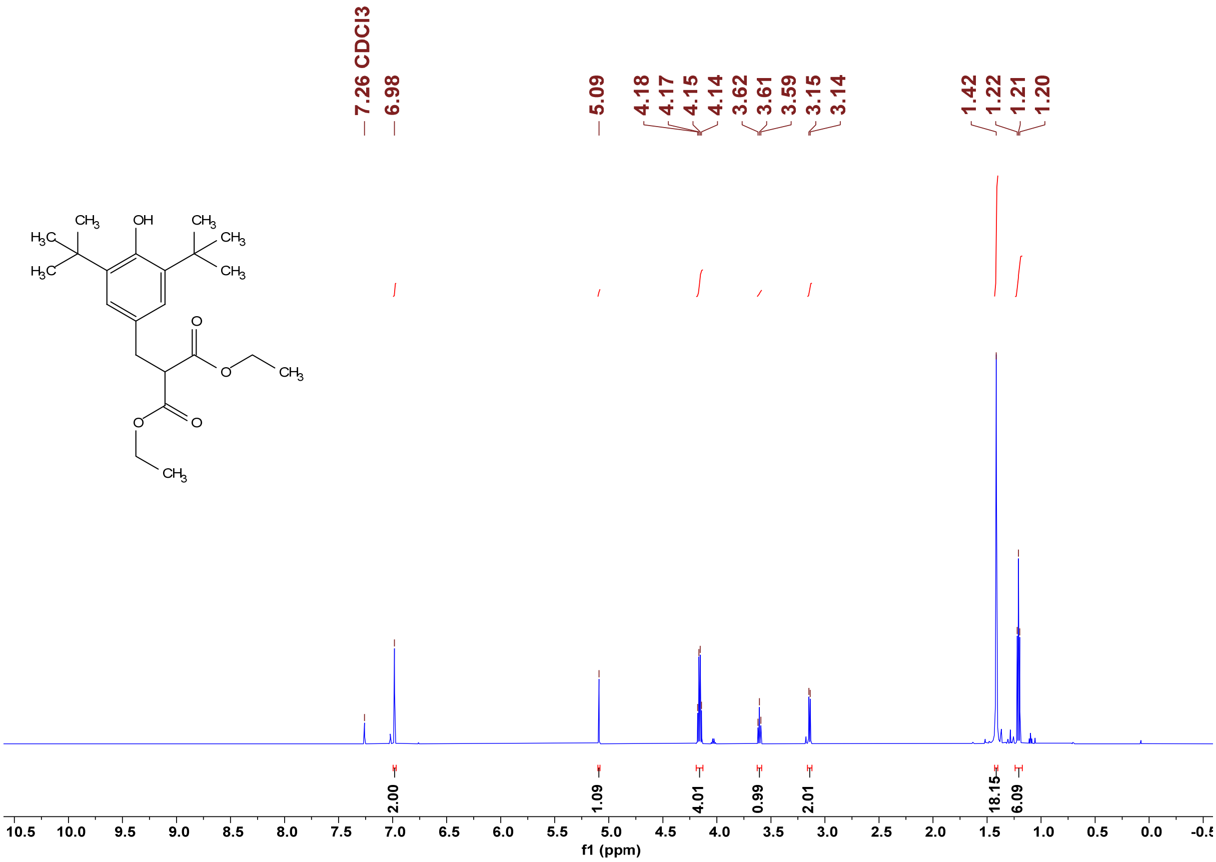

Figure S28. ^{1}H NMR spectrum of **5** in $CDCl_3$ (600 MHz) CG536_4_2

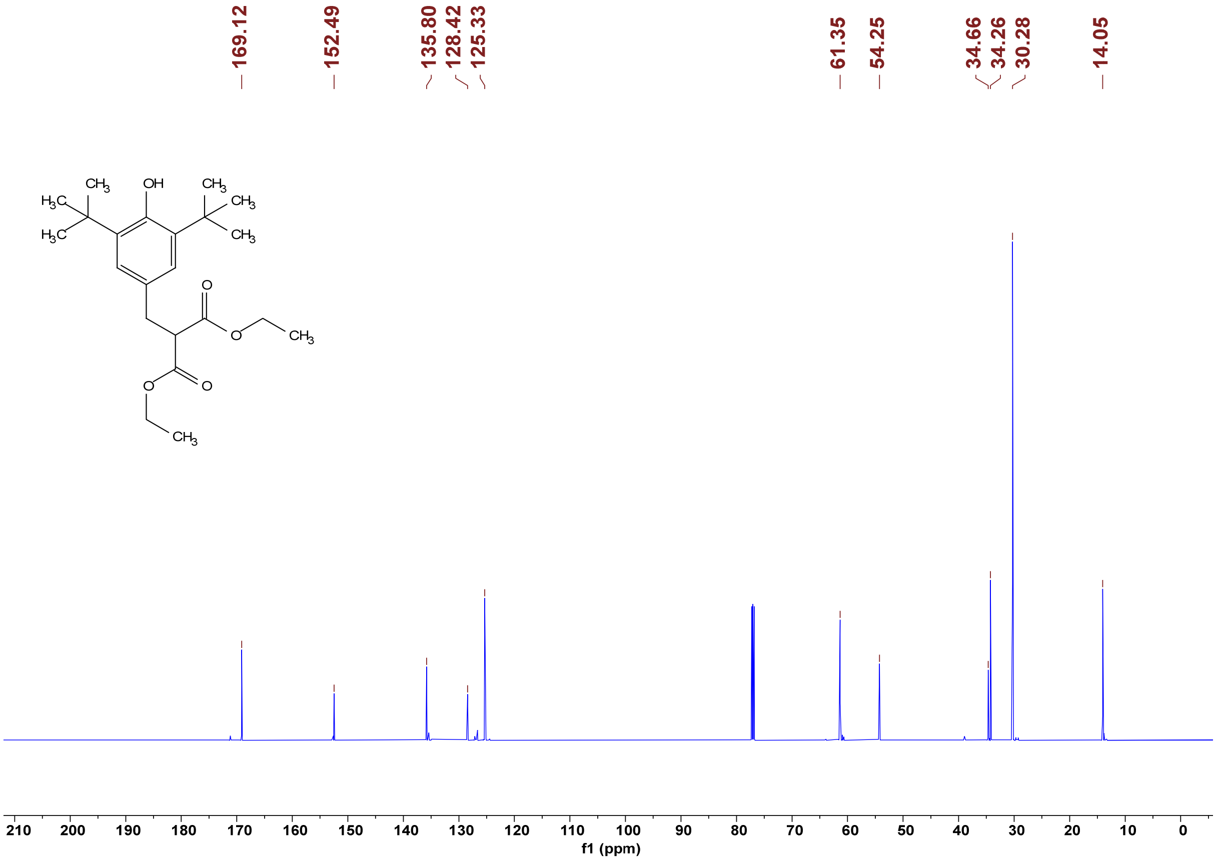

Figure S29. ^{13}C NMR spectrum of **5** in $CDCl_3$ (151 MHz) CG536_4_2

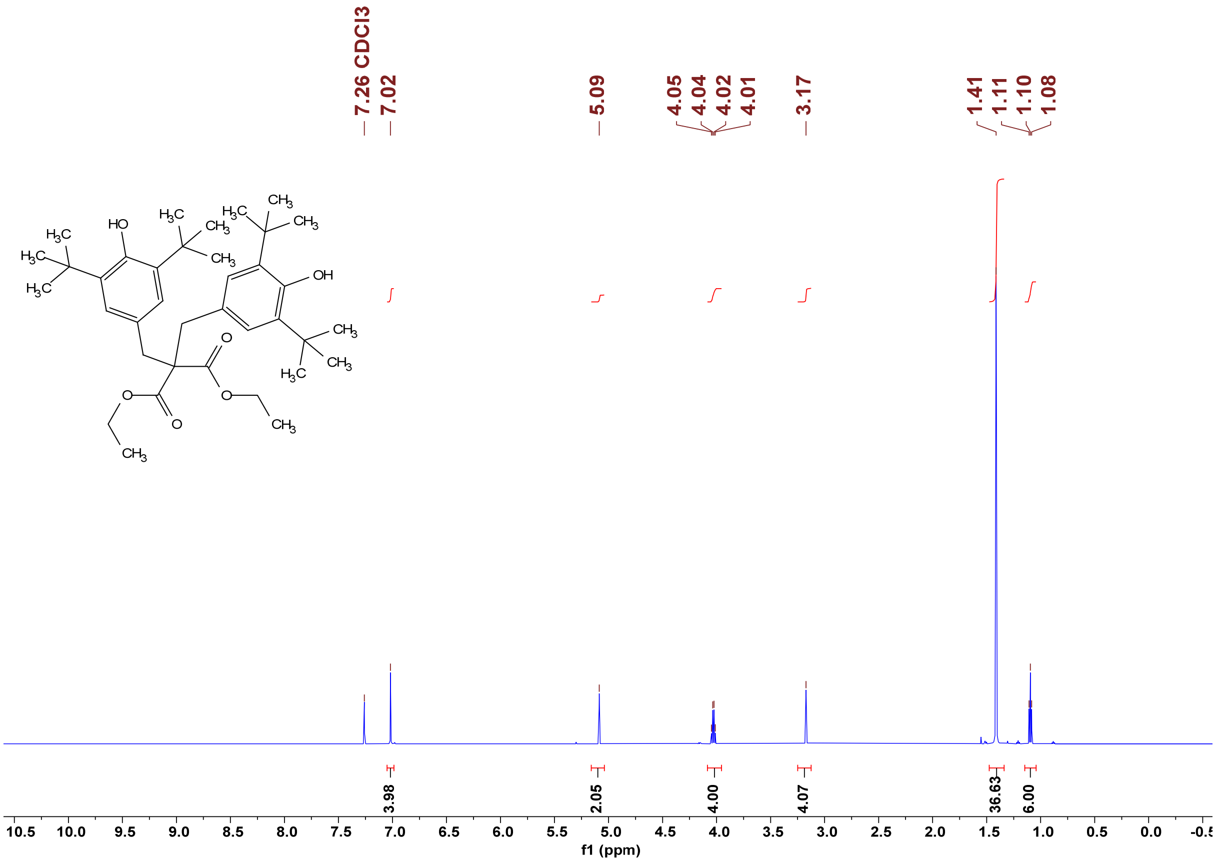

Figure S30. ^{1}H NMR spectrum of **6** in $CDCl_3$ (600 MHz) CG536_4_1

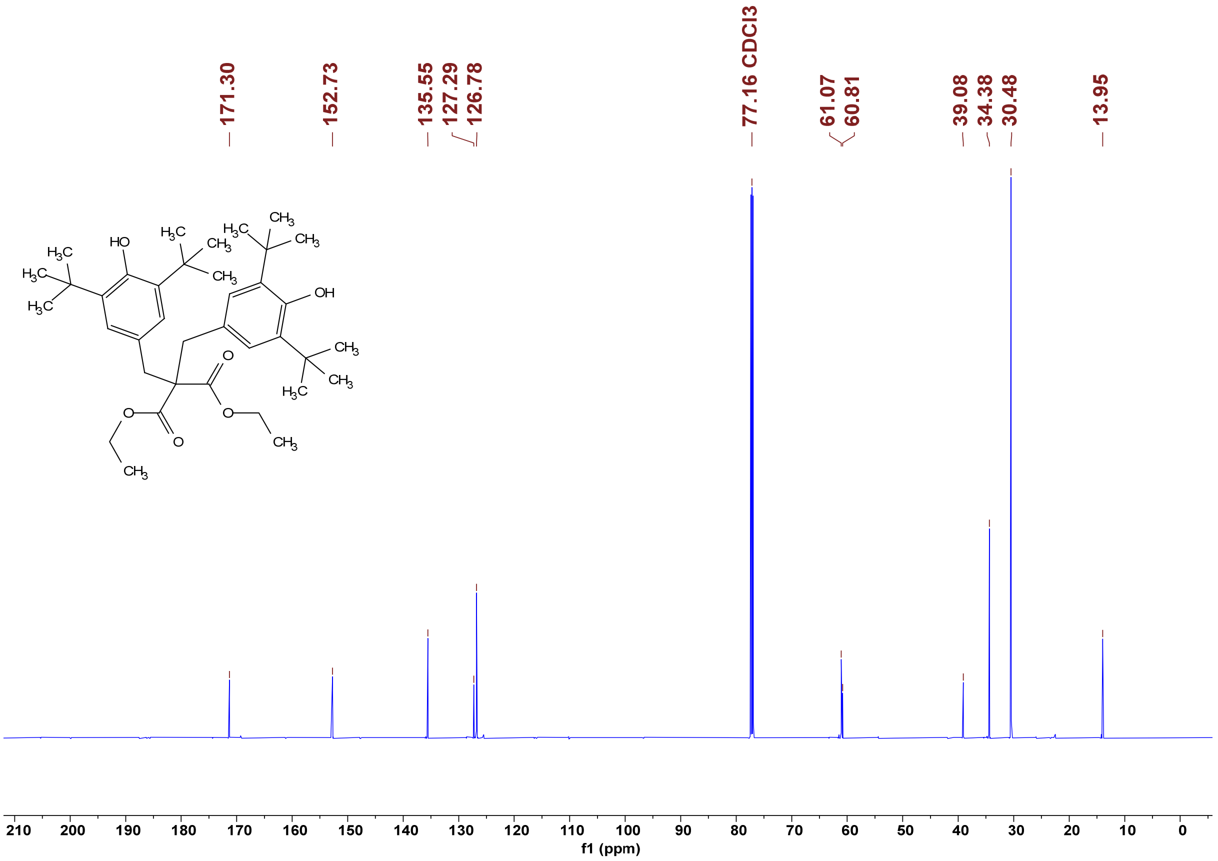

Figure S31. ^{13}C NMR spectrum of **6** in $CDCl_3$ (151 MHz) CG536_4_1

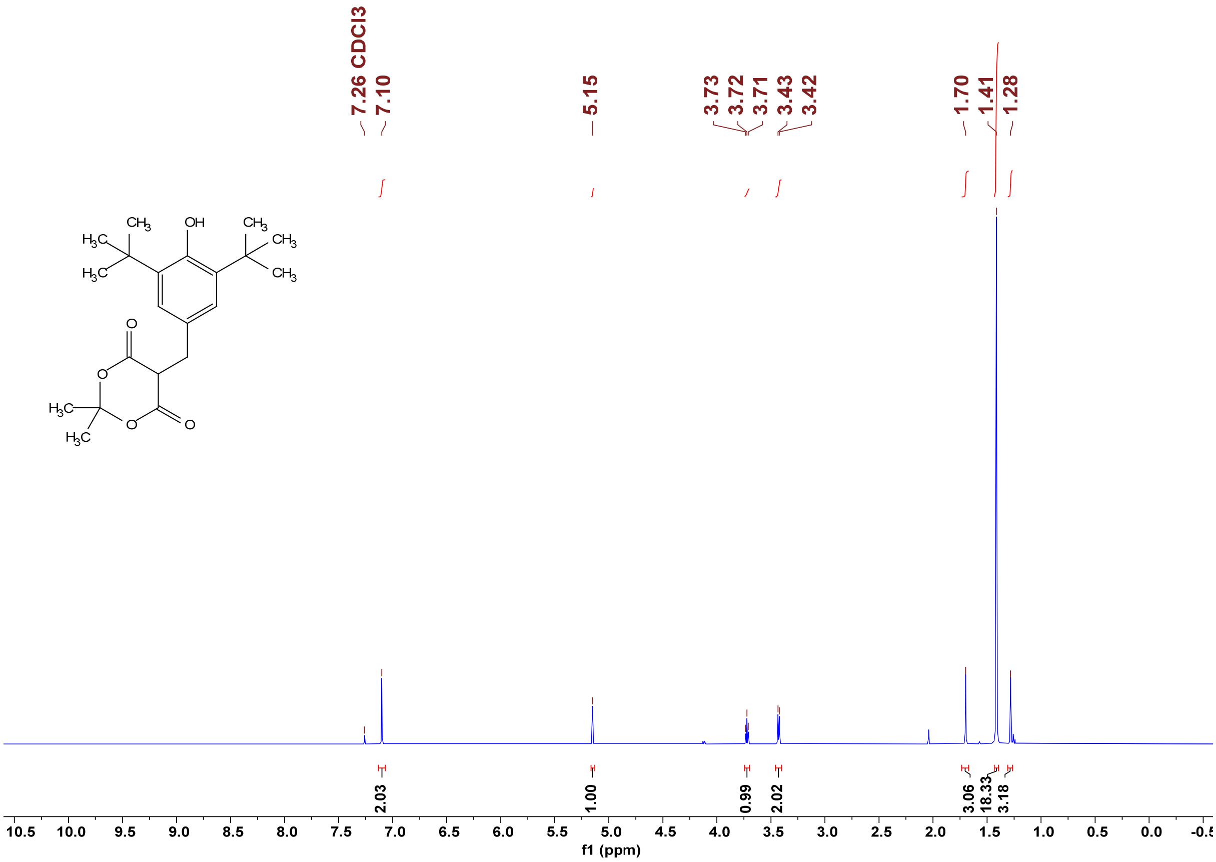

Figure S32. ^{1}H NMR spectrum of **7** in $CDCl_3$ (400 MHz) CG542

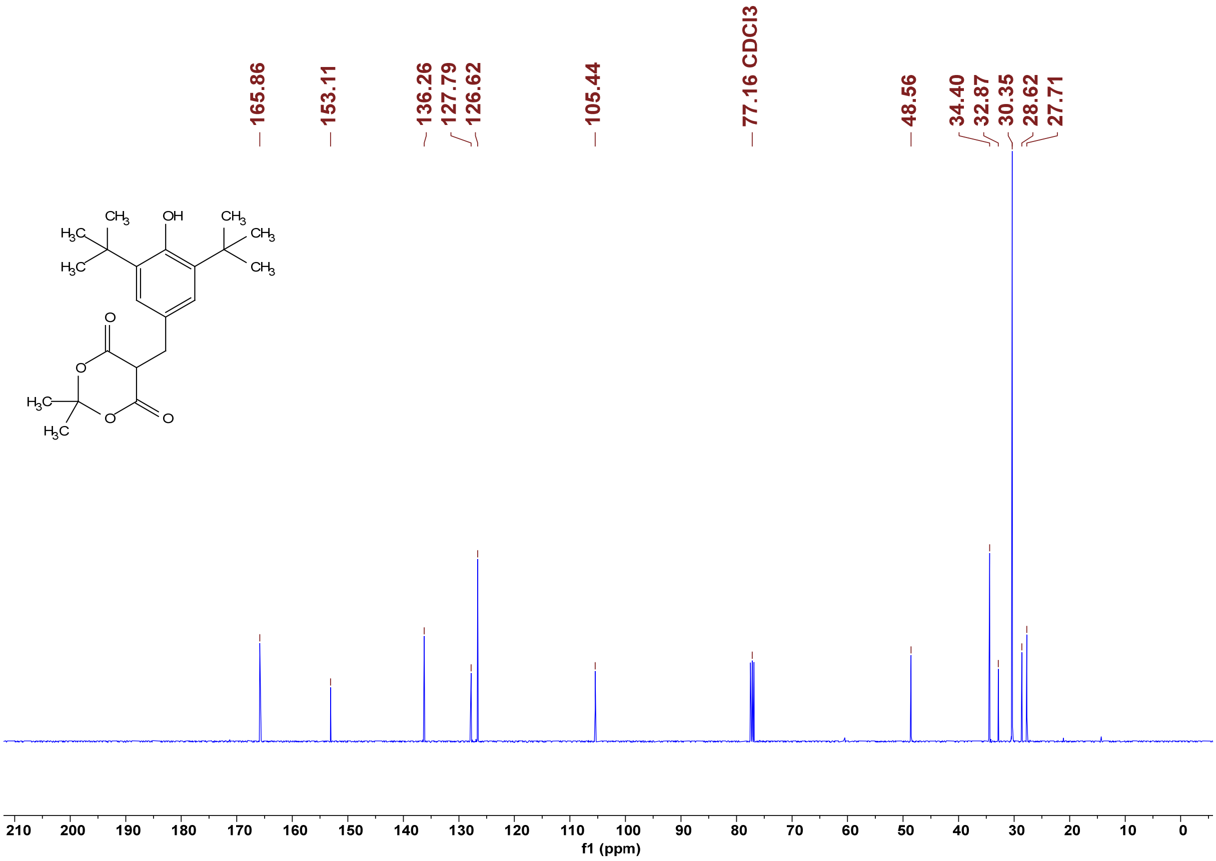

Figure S33. ^{13}C NMR spectrum of **7** in $CDCl_3$ (101 MHz) CG542

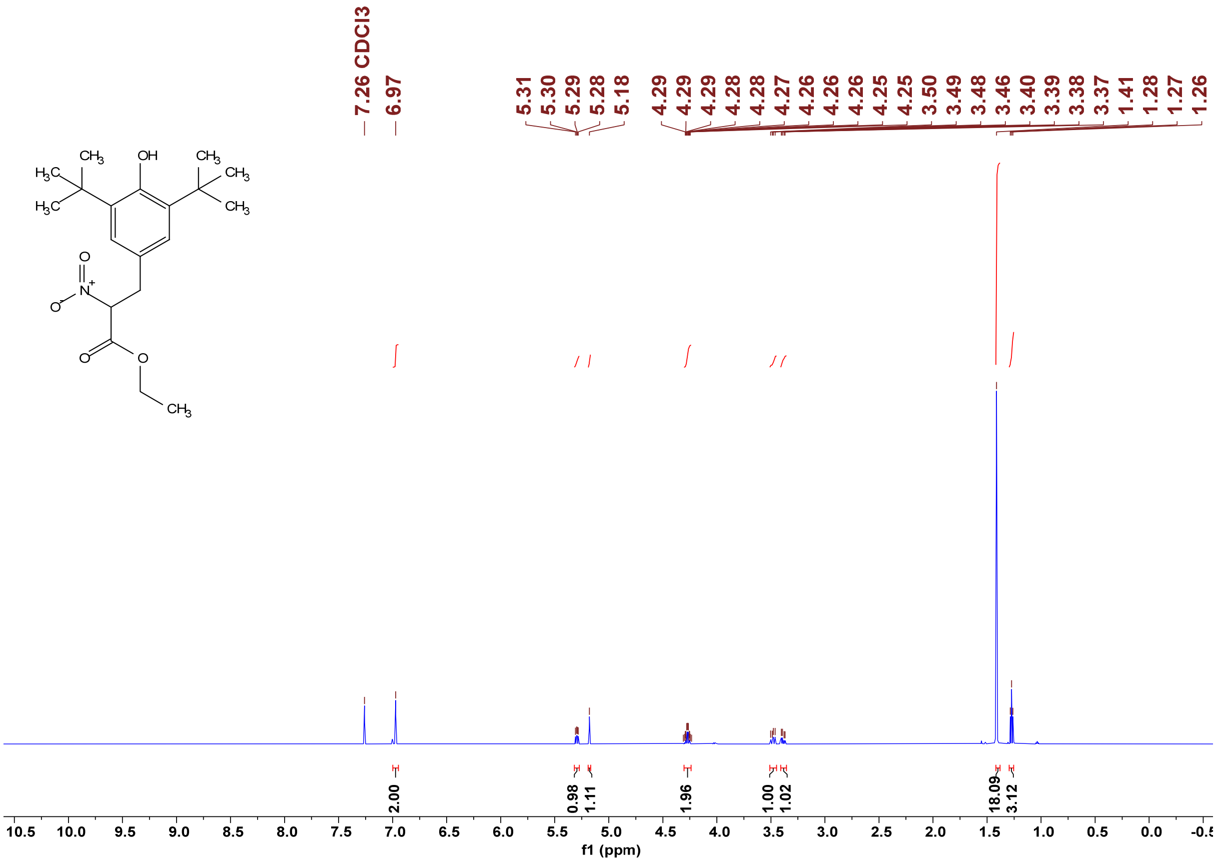

Figure S34. ^{1}H NMR spectrum of **8** in $CDCl_3$ (600 MHz) CG543

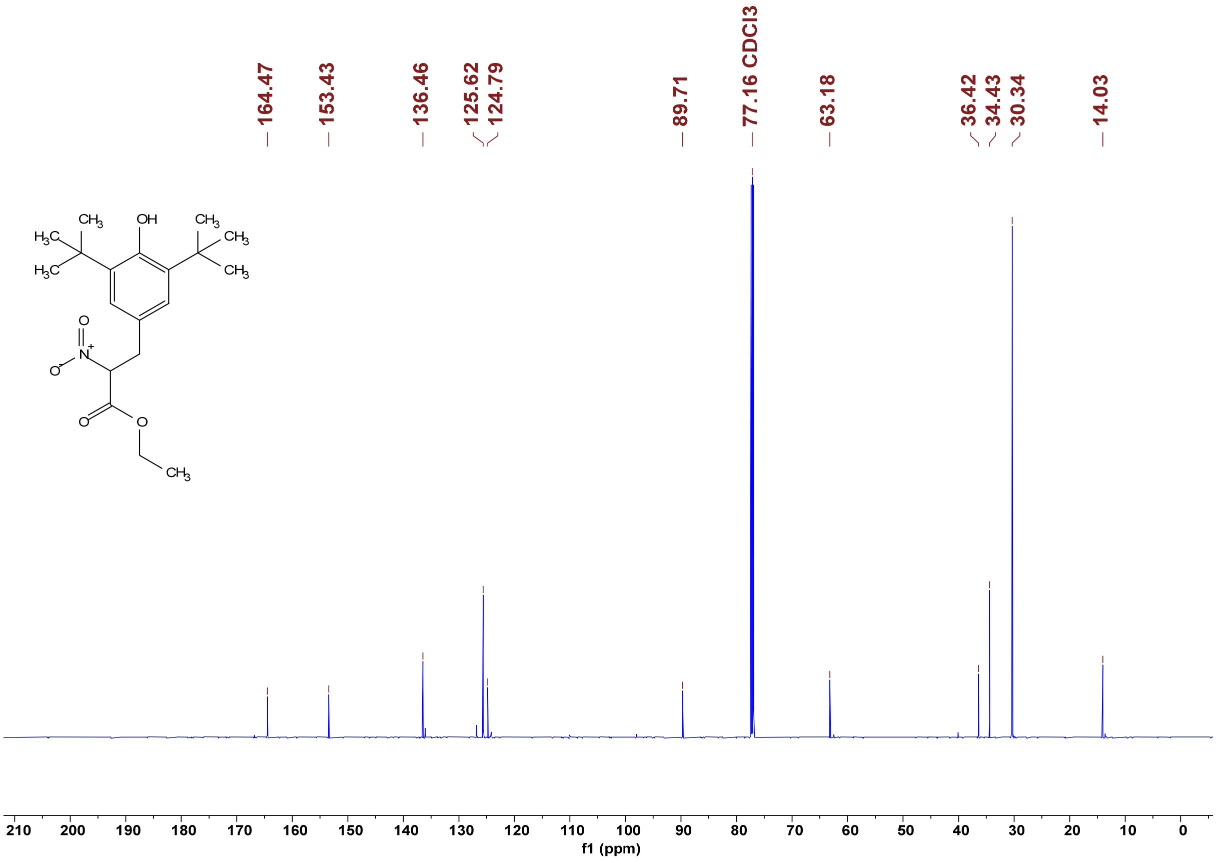

Figure S35. ^{13}C NMR spectrum of **8** in $CDCl_3$ (151 MHz) CG543

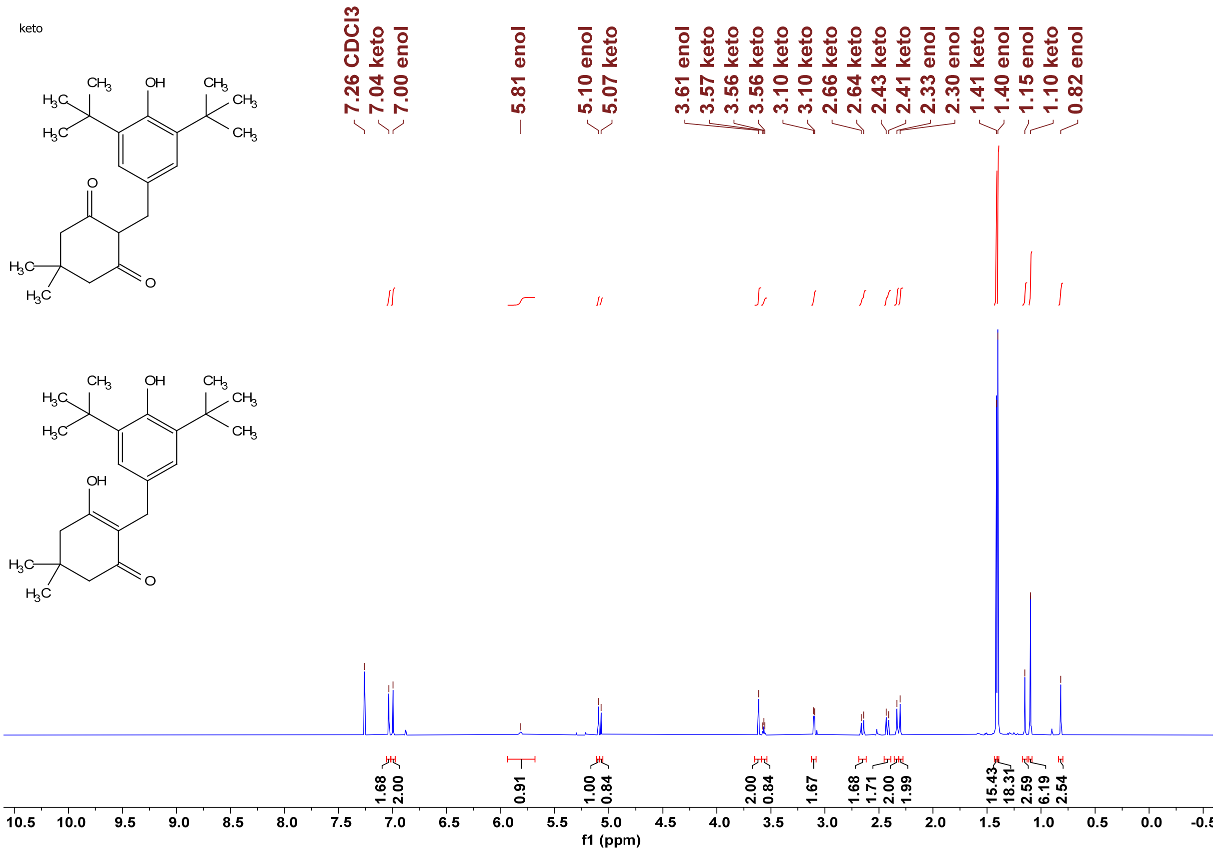

Figure S36. ^{1}H NMR spectrum of 3ab in $CDCl_3$ (600 MHz) CG538_1F2

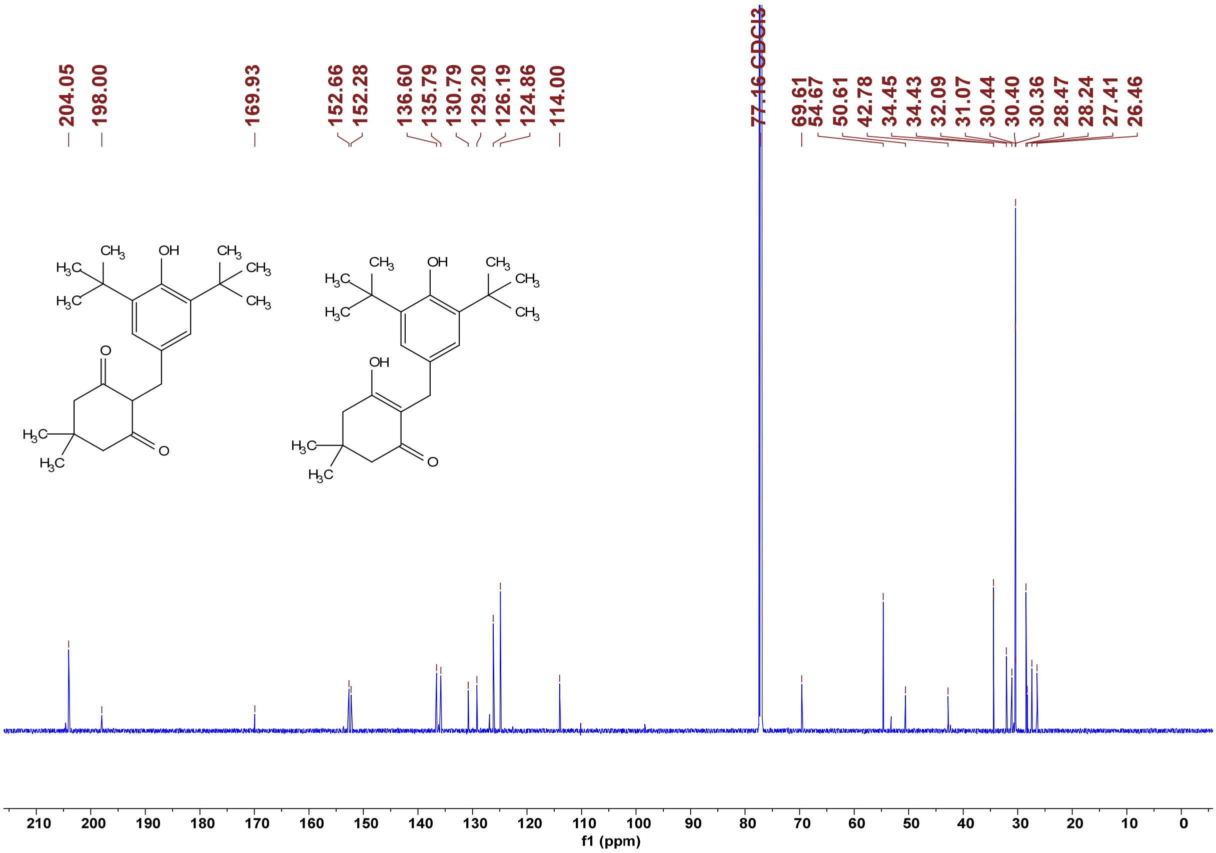

Figure S37. ^{13}C NMR spectrum of **3ab** in $CDCl_3$ (151 MHz) CG538_1F2

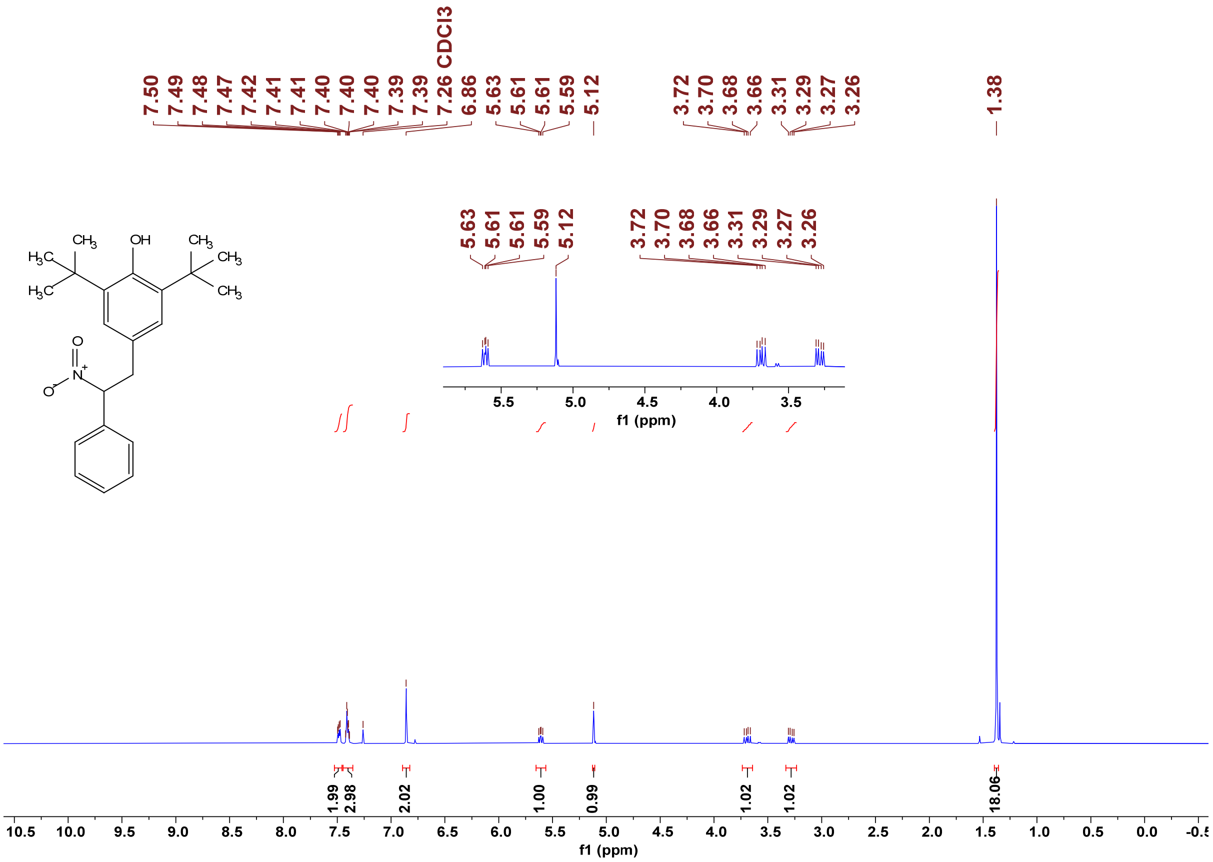

Figure S38. ^{1}H NMR spectrum of **10** in $CDCl_3$ (400 MHz) CG654

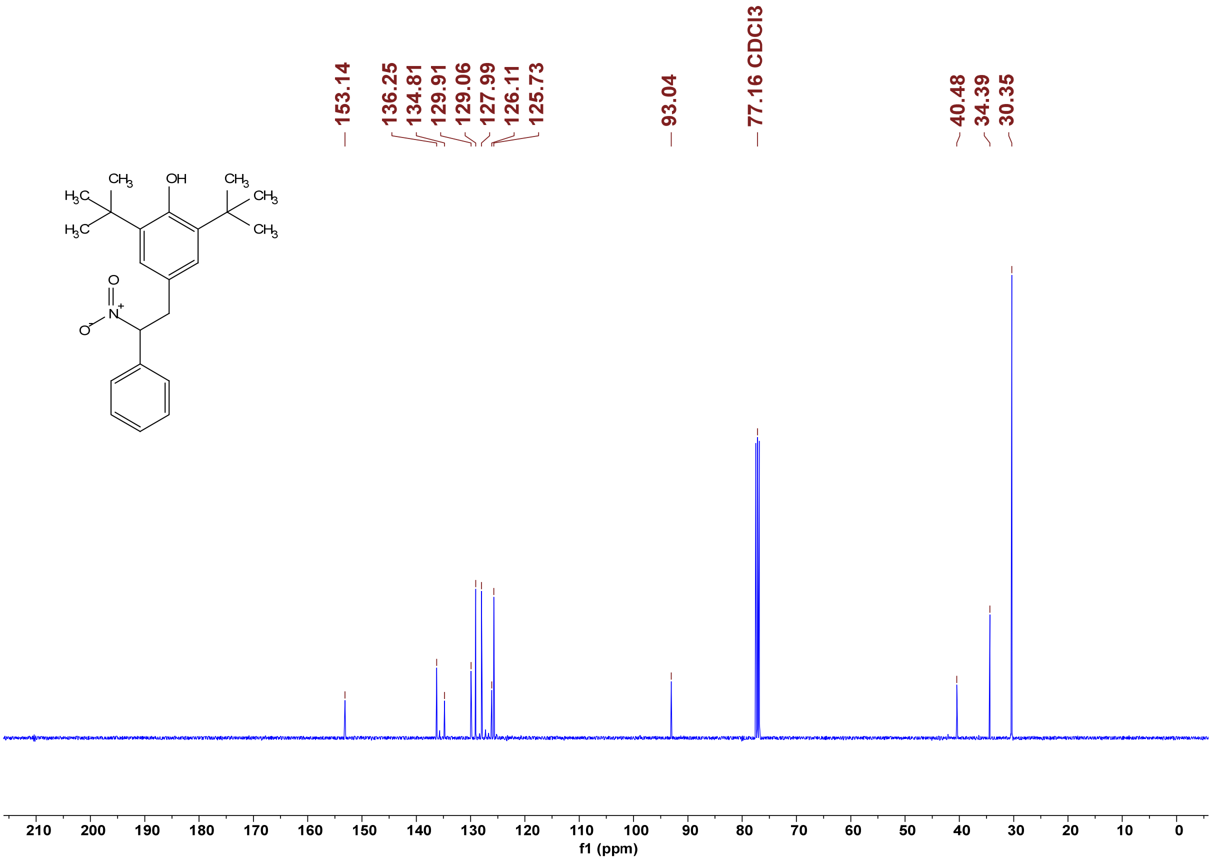

Figure S39. ^{13}C NMR spectrum of **10** in $CDCl_3$ (101 MHz) CG654

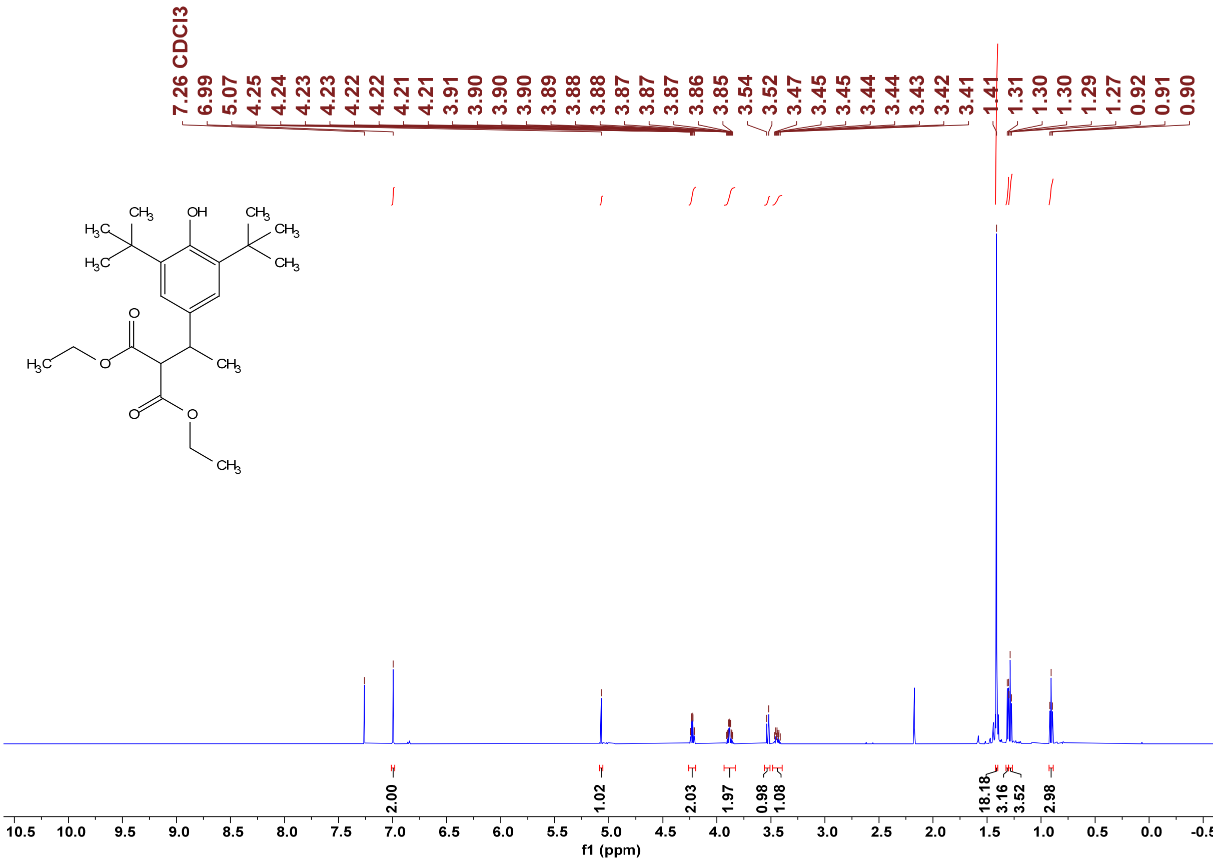

Figure S40. ^{1}H NMR spectrum of **11** in $CDCl_3$ (600 MHz) CG546

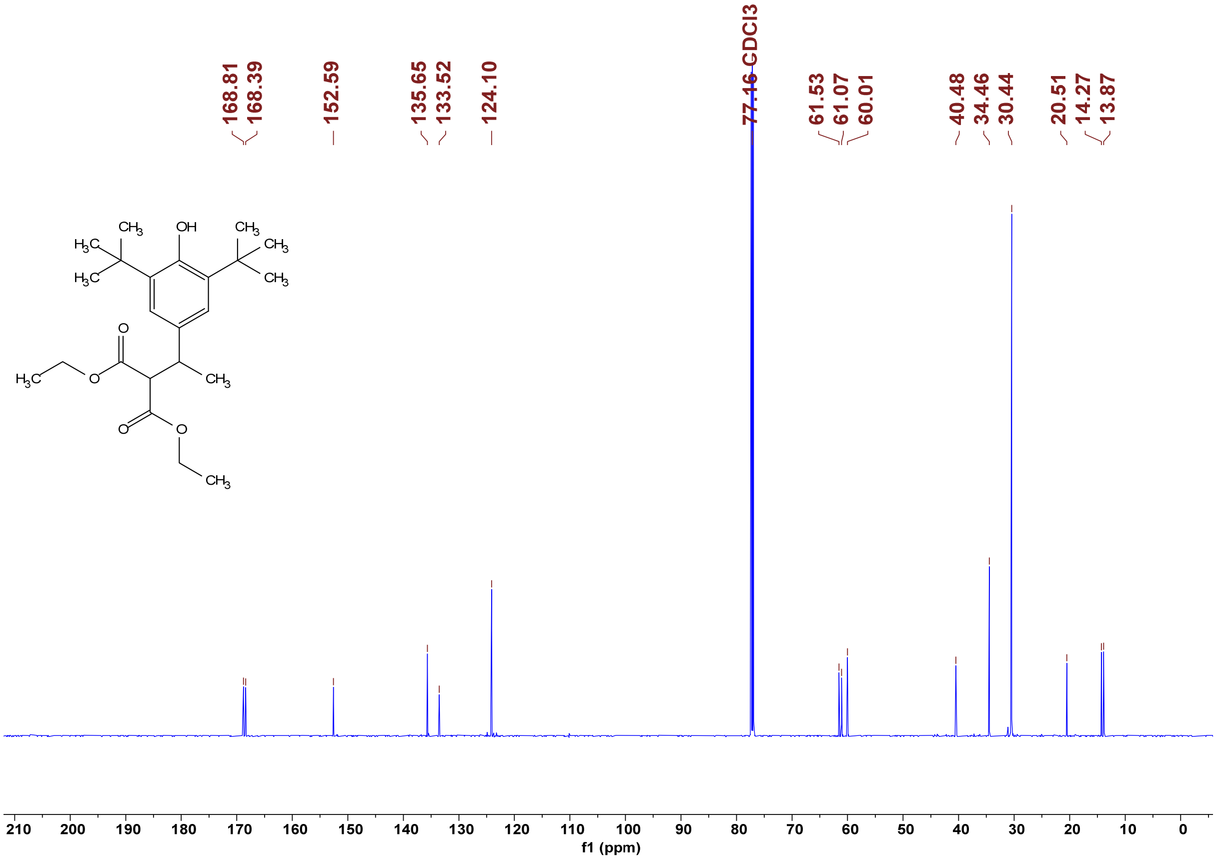

Figure S41. ^{13}C NMR spectrum of **11** in $CDCl_3$ (151 MHz) CG546

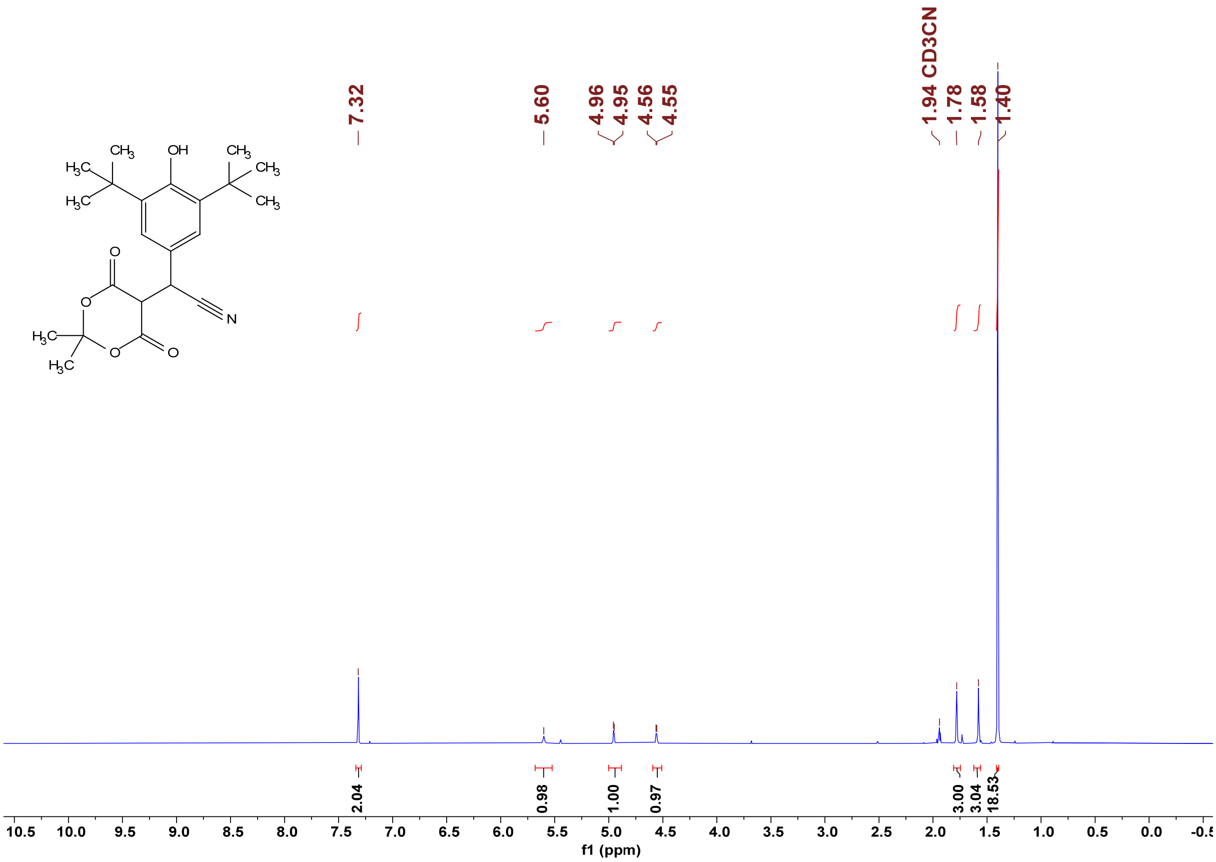

Figure S42. ^{1}H NMR spectrum of **12** in CD_3CN (400 MHz) CG651_1

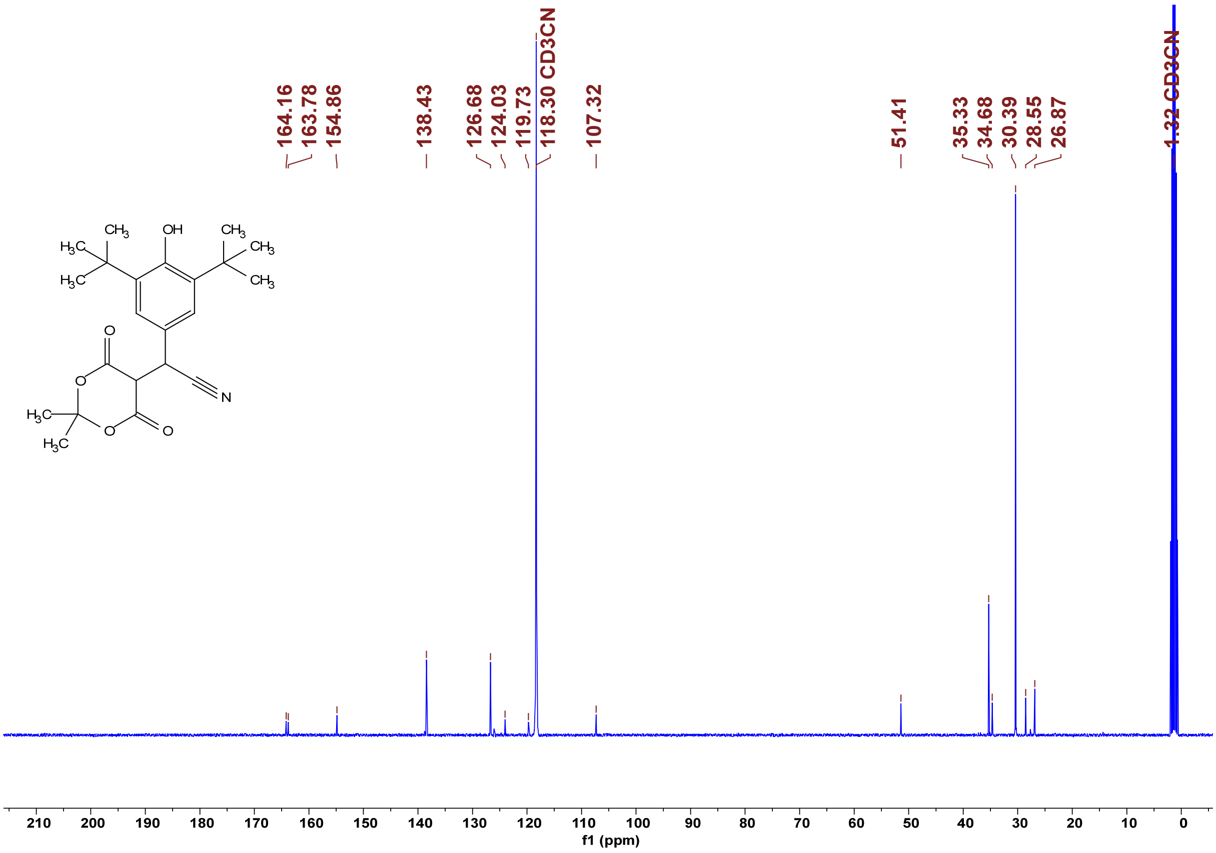

Figure S43. ^{13}C NMR spectrum of **12** in CD_3CN (101 MHz) CG651_1

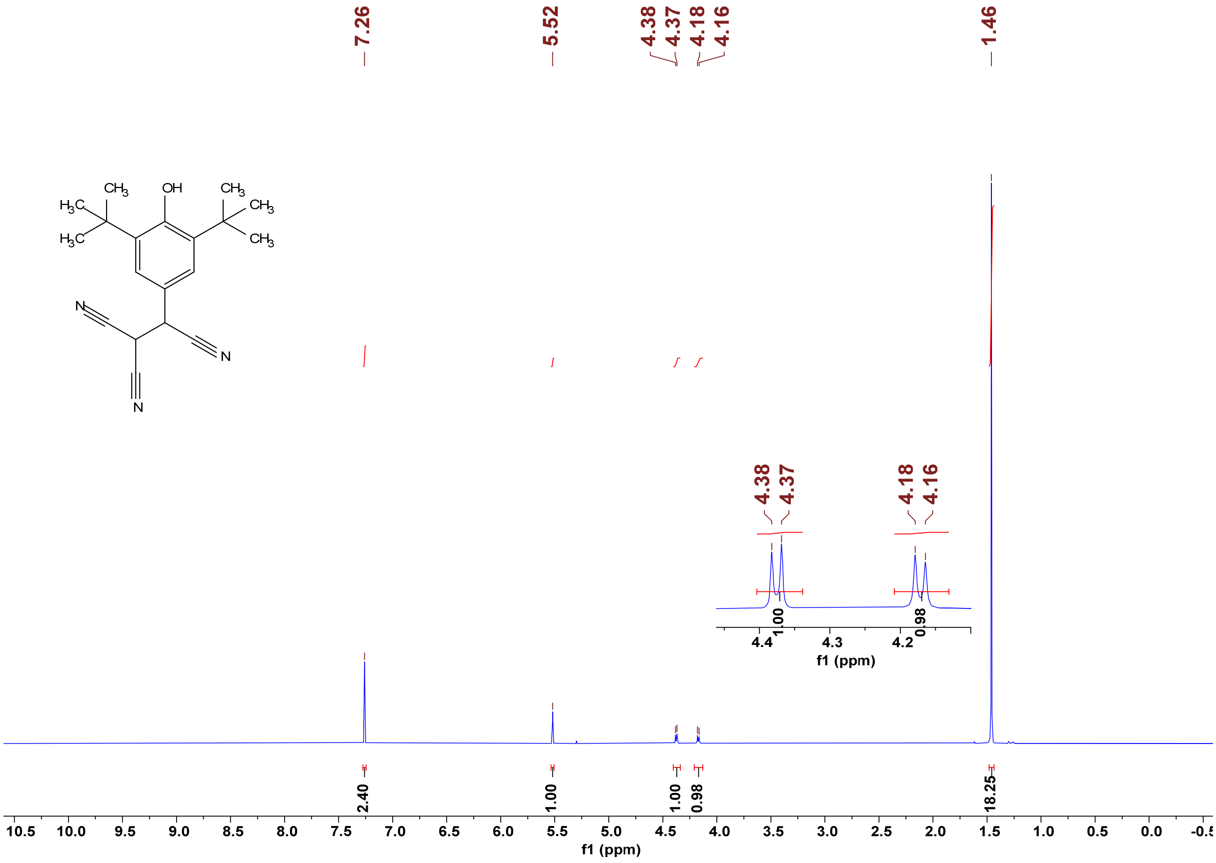

Figure S44. ^{1}H NMR spectrum of **13** in $CDCl_3$ (400 MHz) CG553

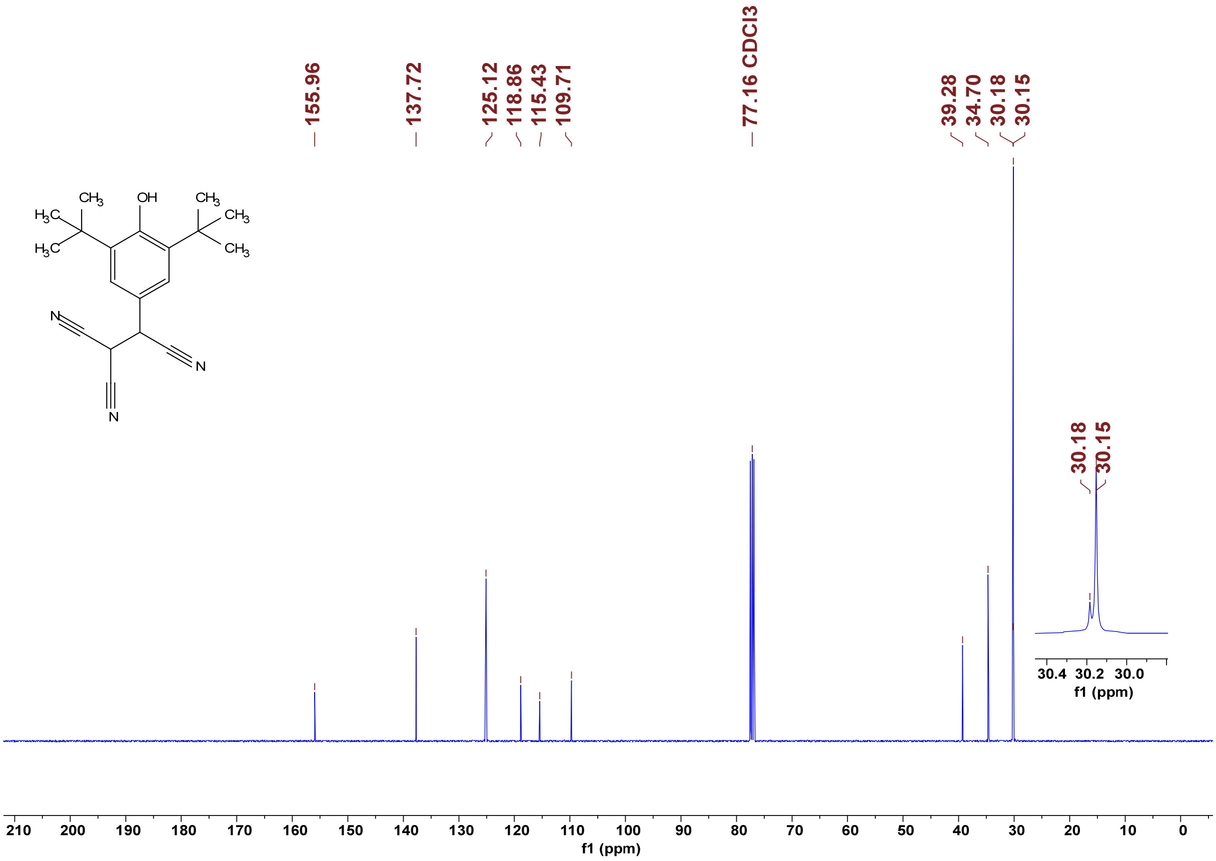

Figure S45. ^{13}C NMR spectrum of **13** in $CDCl_3$ (101 MHz) CG553

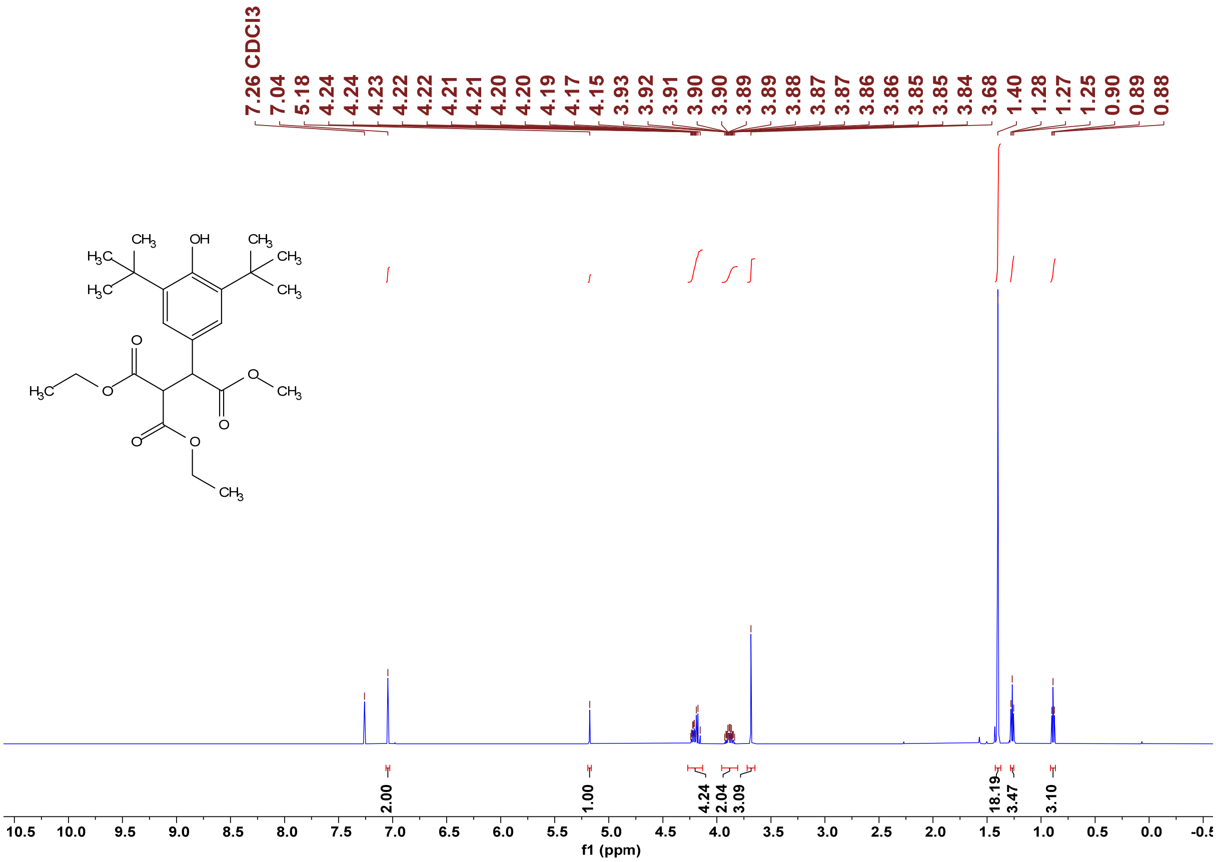

Figure S46. ^{1}H NMR spectrum of **14** in $CDCl_3$ (600 MHz) CG524_1

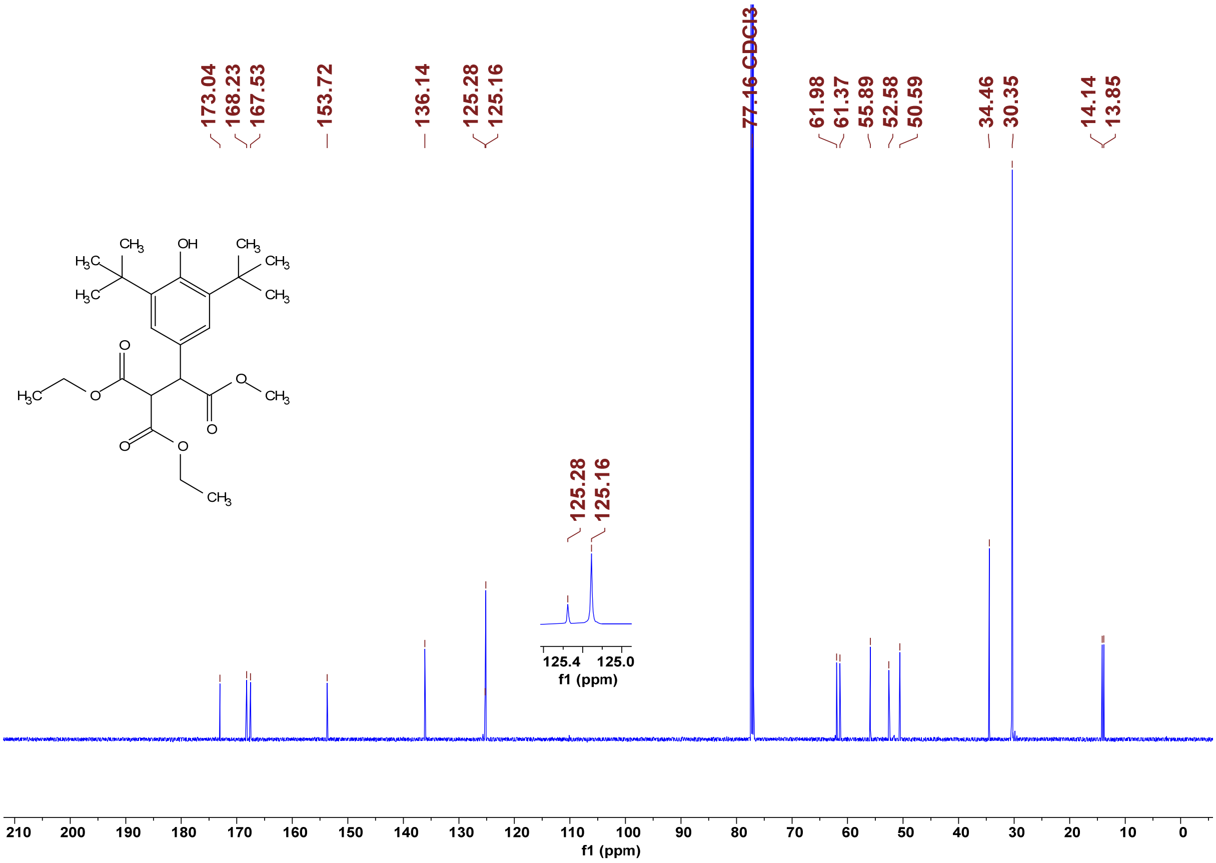

Figure S47. ^{13}C NMR spectrum of **14** in $CDCl_3$ (151 MHz) CG524_1

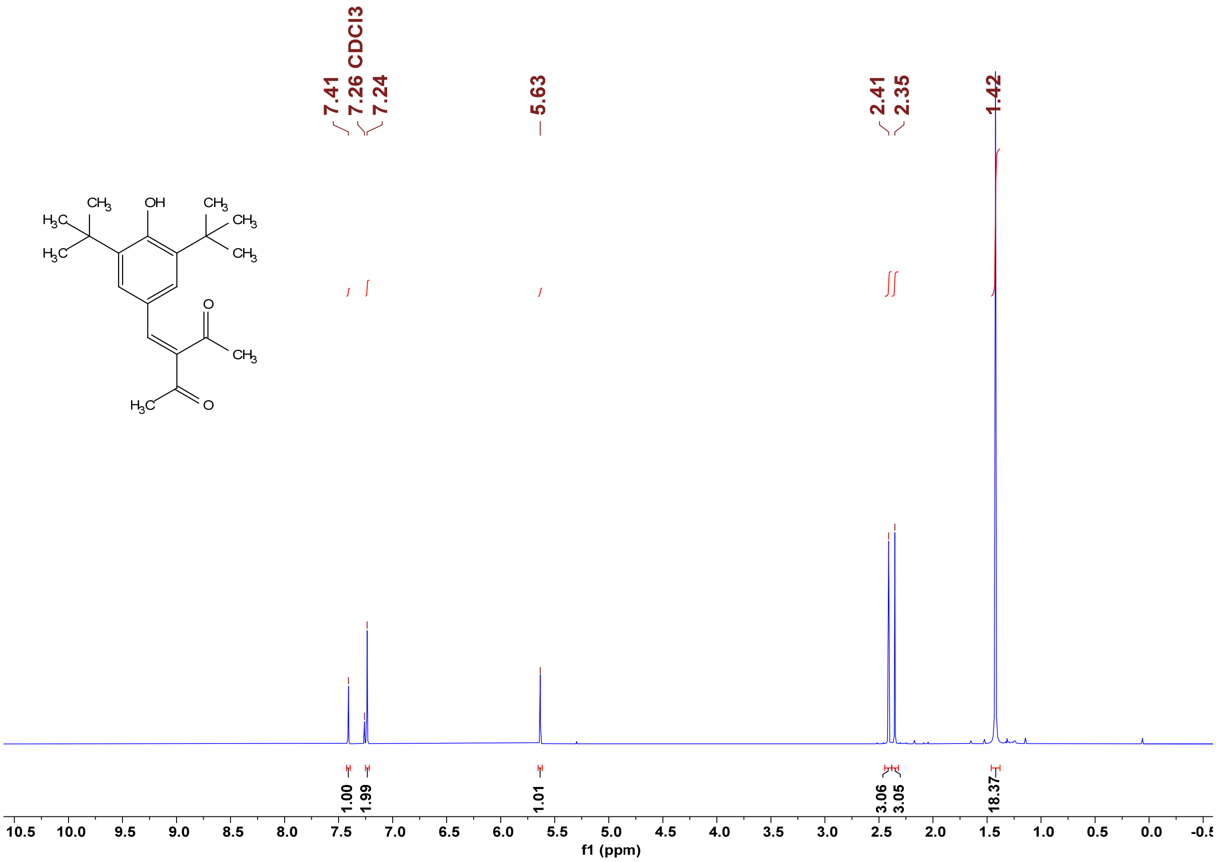

Figure S48. ^{1}H NMR spectrum of **15** in $CDCl_3$ (600 MHz) CG652

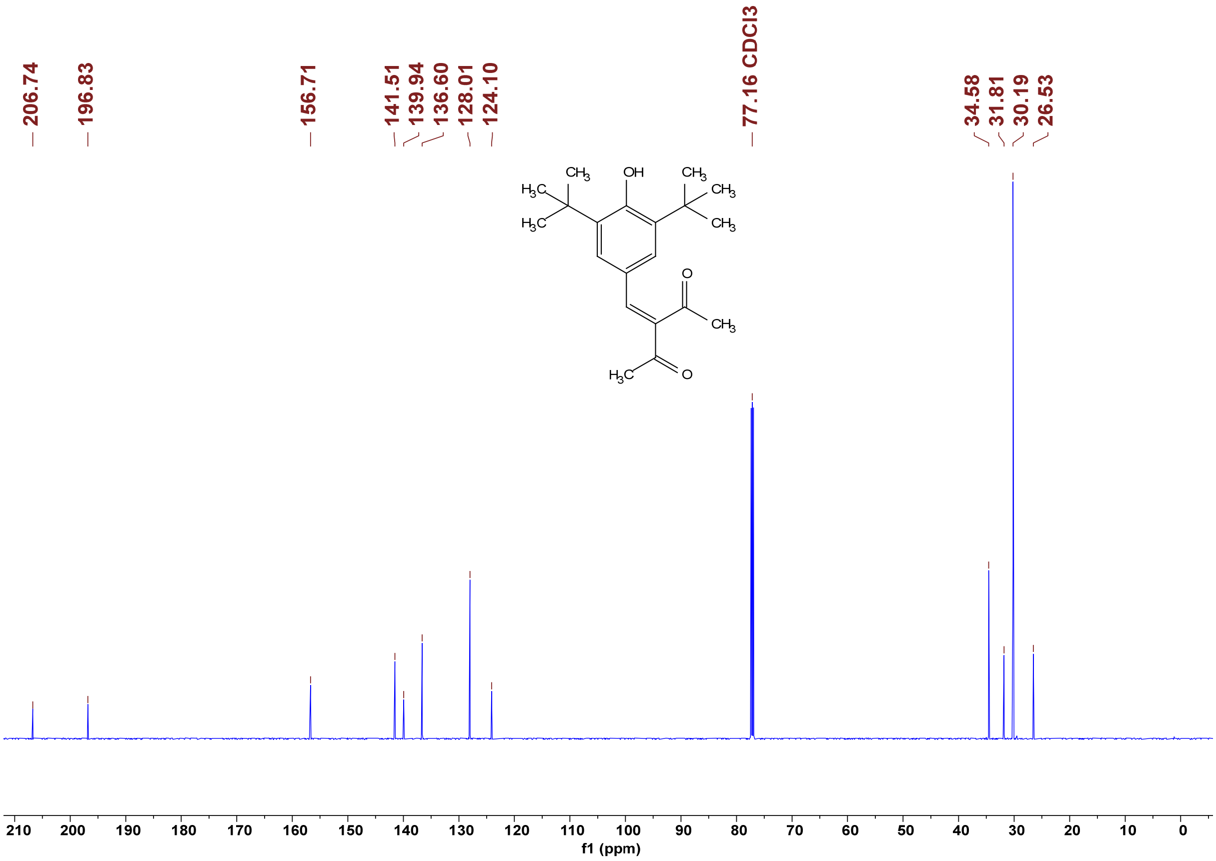

Figure S49. ^{13}C NMR spectrum of **15** in $CDCl_3$ (151 MHz) CG652

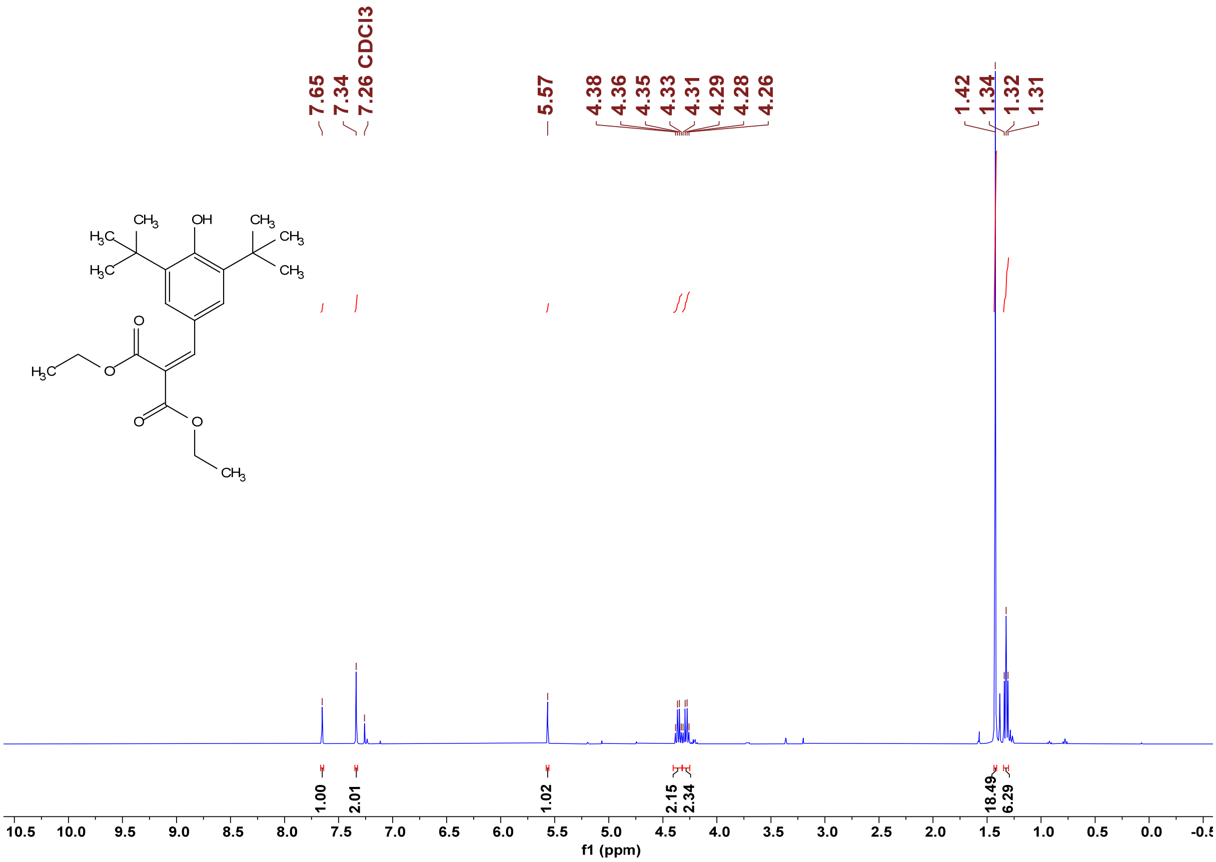

Figure S50. ^{1}H NMR spectrum of **16** in $CDCl_3$ (400 MHz) CG547_1

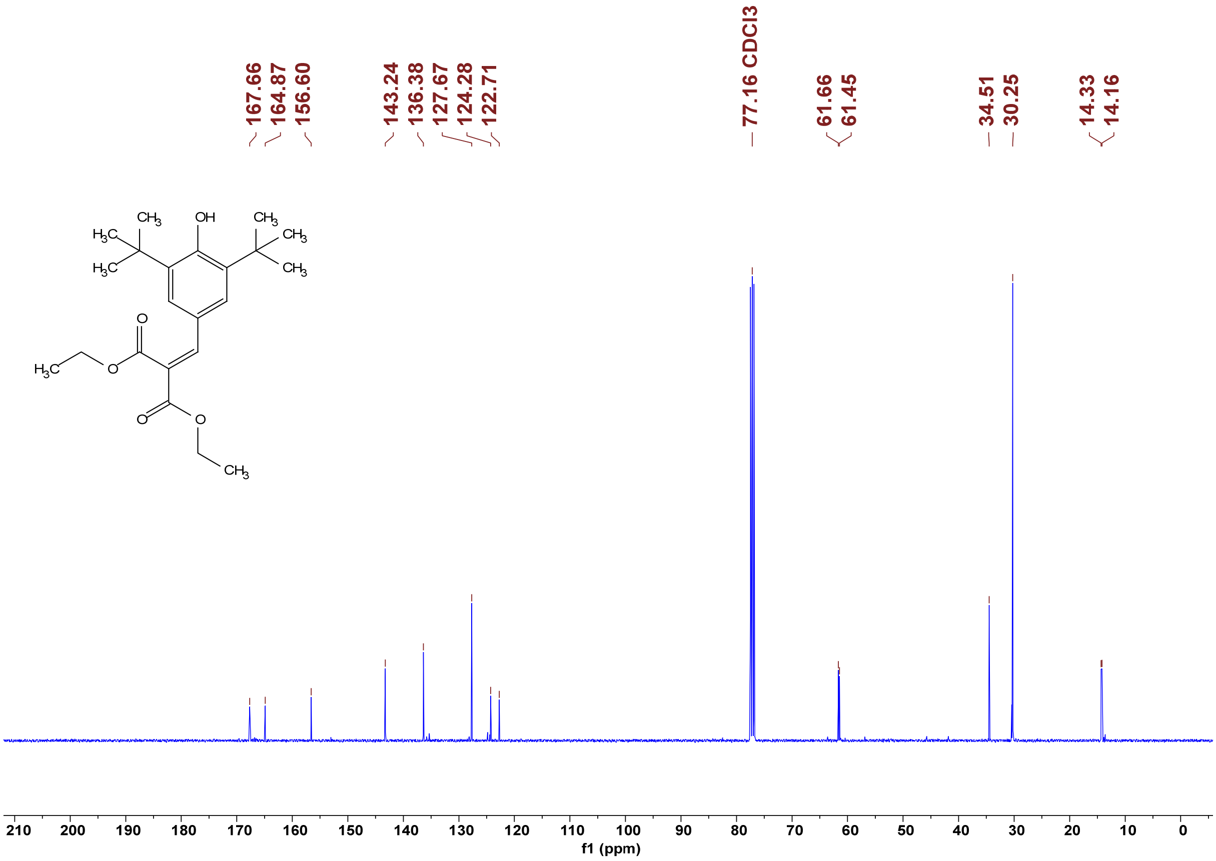

Figure S51. ^{13}C NMR spectrum of **16** in $CDCl_3$ (101 MHz) CG547_1

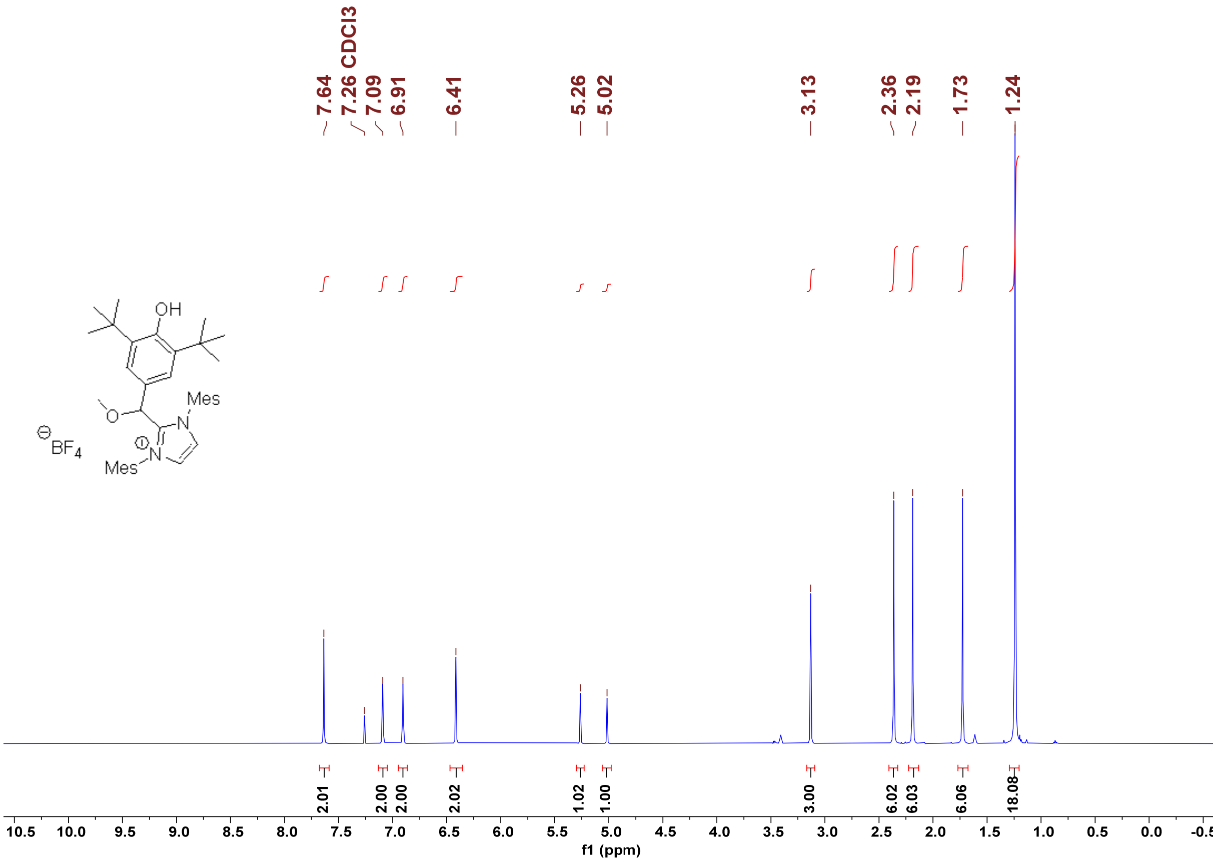

Figure S52. ^{1}H NMR spectrum of **18** in $CDCl_3$ (600 MHz) CG688

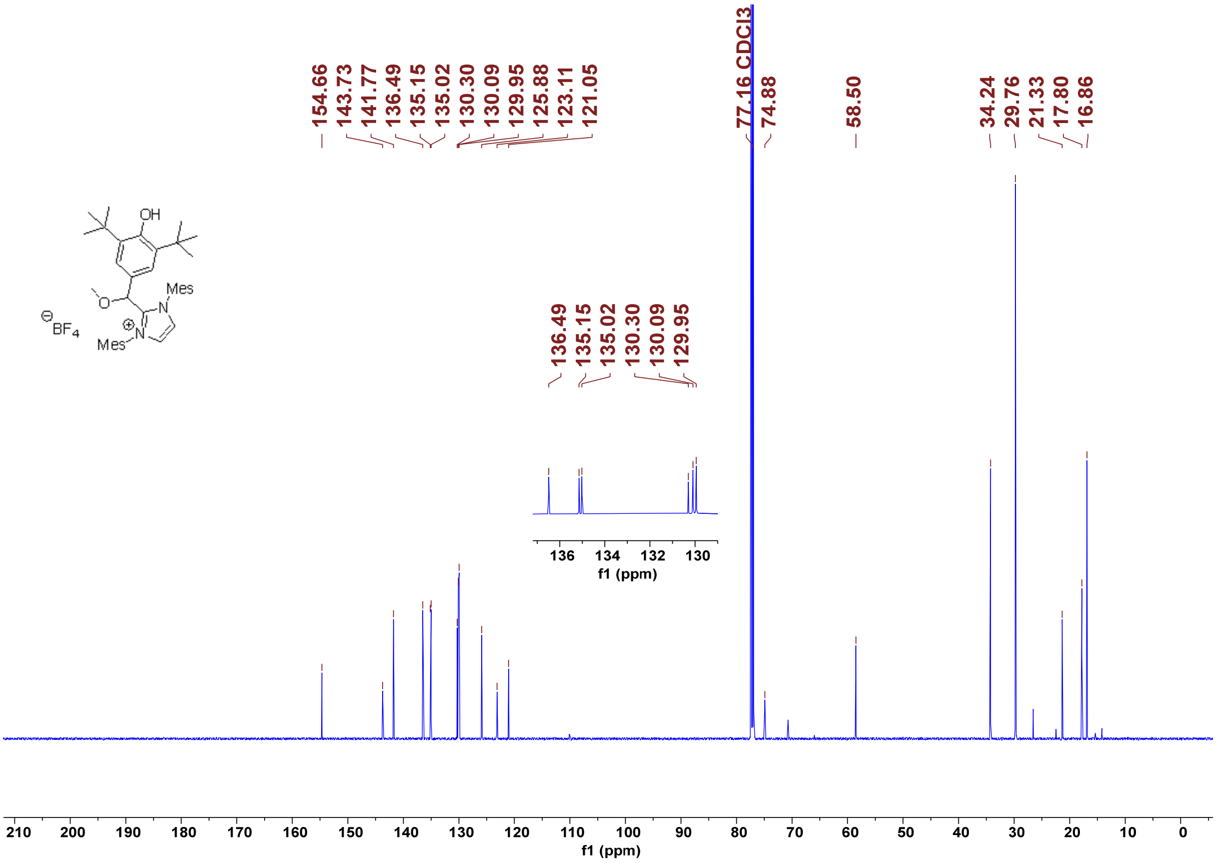

Figure S53. ^{13}C NMR spectrum of **18** in $CDCl_3$ (151 MHz) CG688

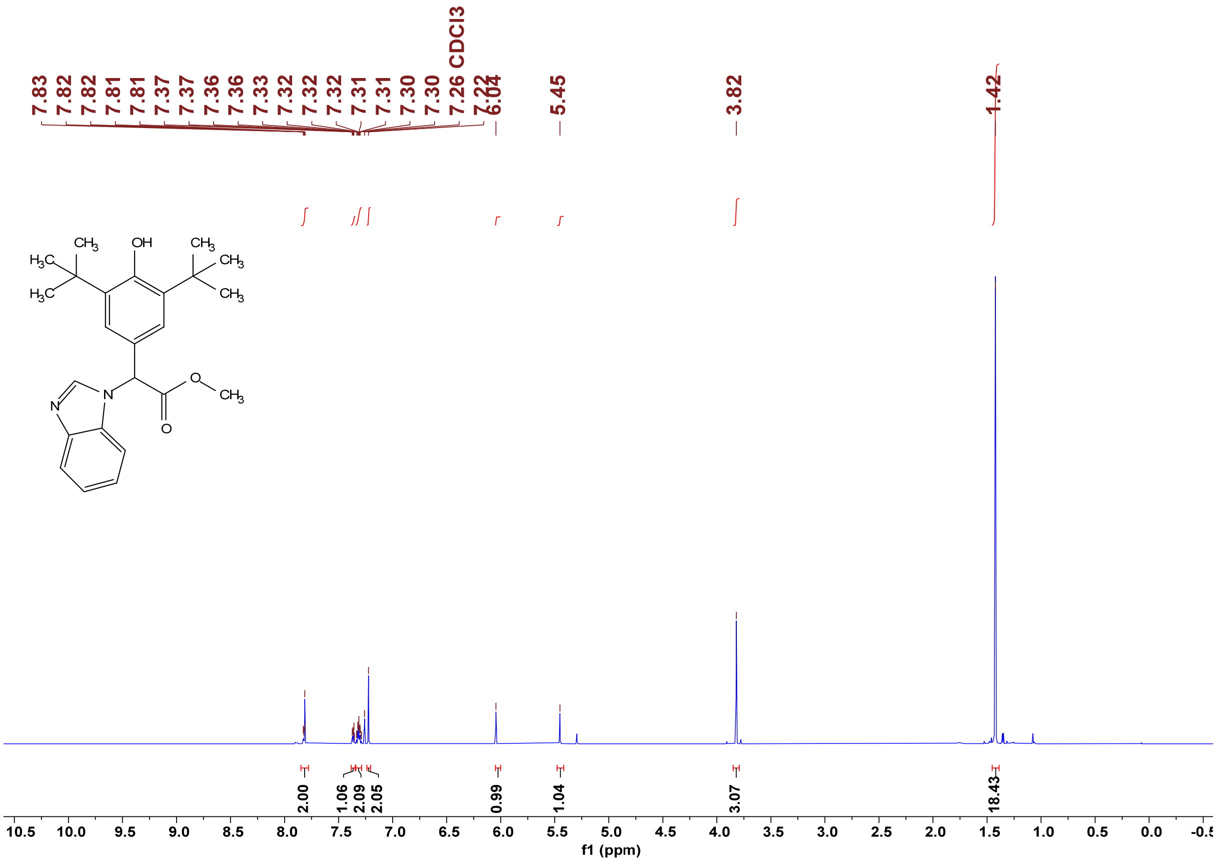

Figure S54. ^{1}H NMR spectrum of **20** in $CDCl_3$ (600 MHz) CG690

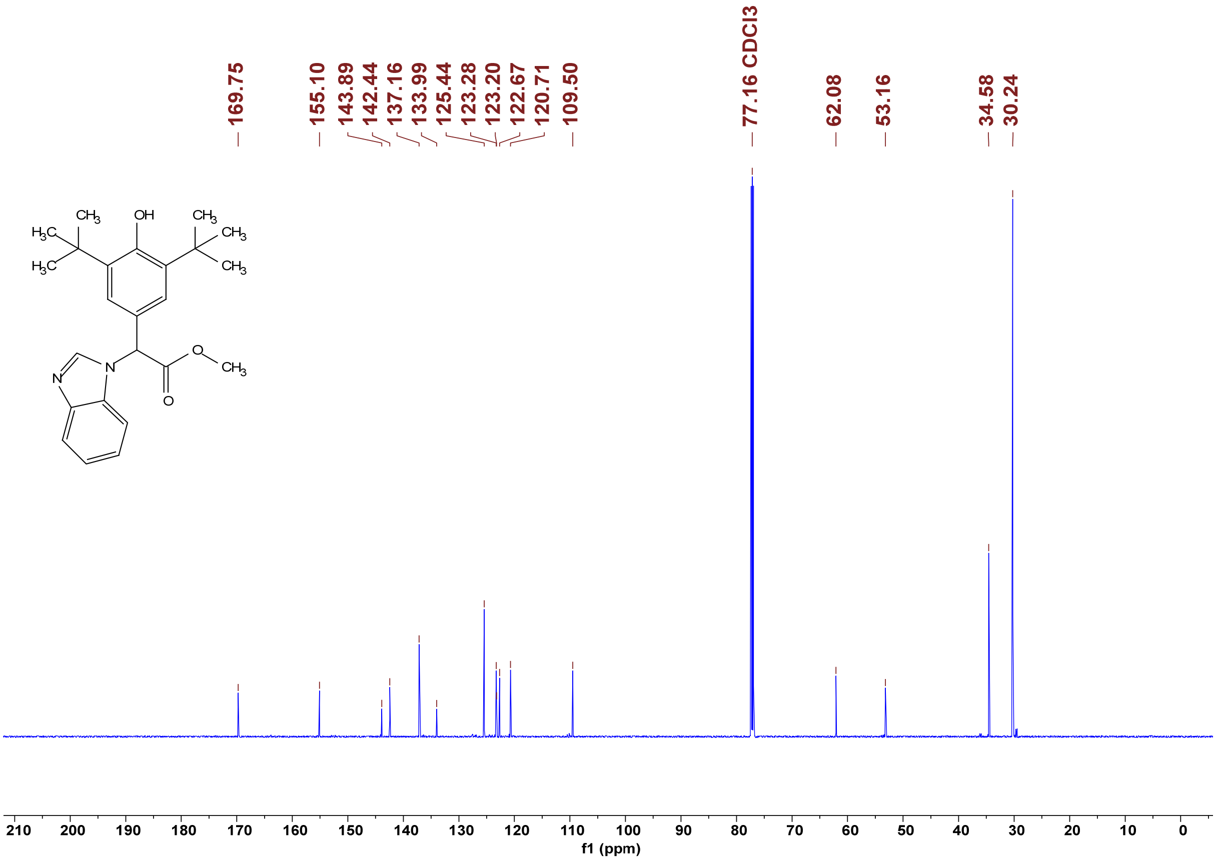

Figure S55. ^{13}C NMR spectrum of **20** in $CDCl_3$ (151 MHz) CG690

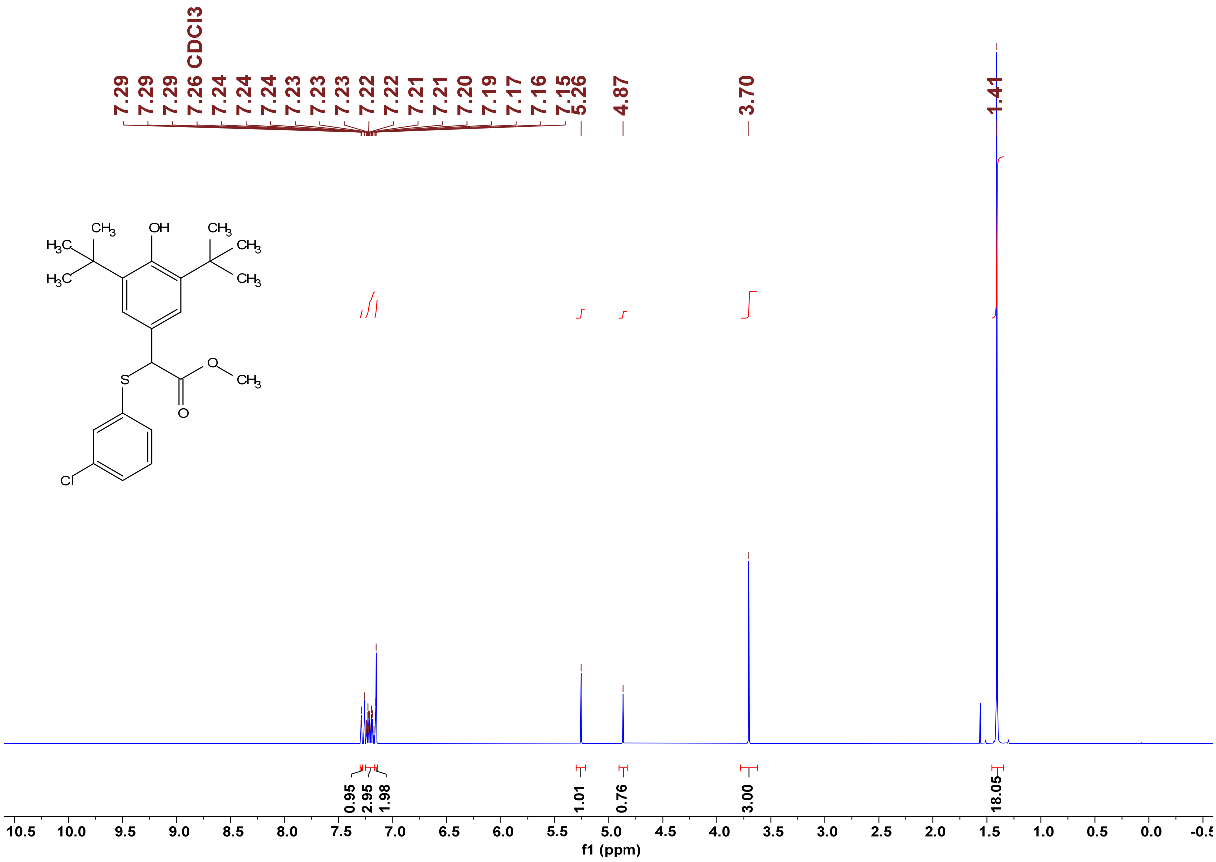

Figure S56. ^{1}H NMR spectrum of **22** in $CDCl_3$ (600 MHz) CG693F2

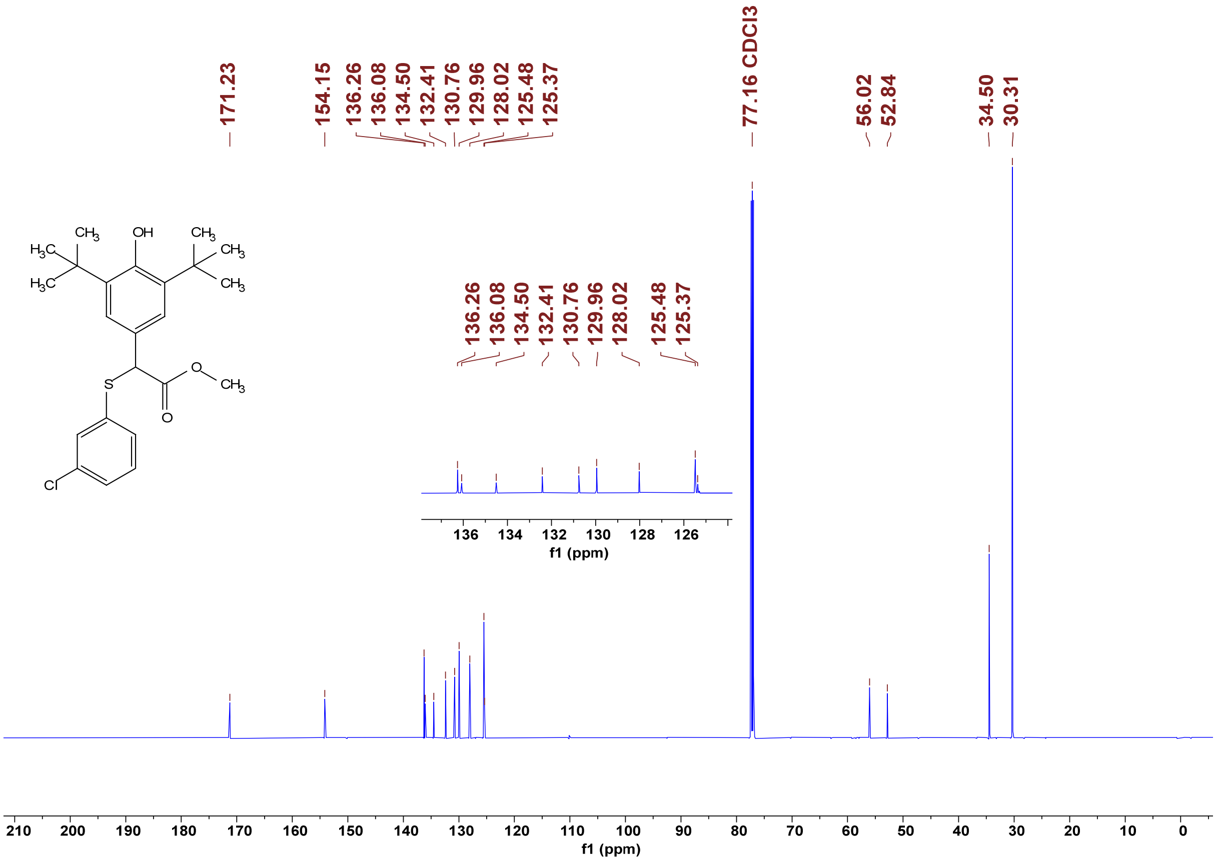

Figure S57. ^{13}C NMR spectrum of **22** in $CDCl_3$ (151 MHz) CG693F2

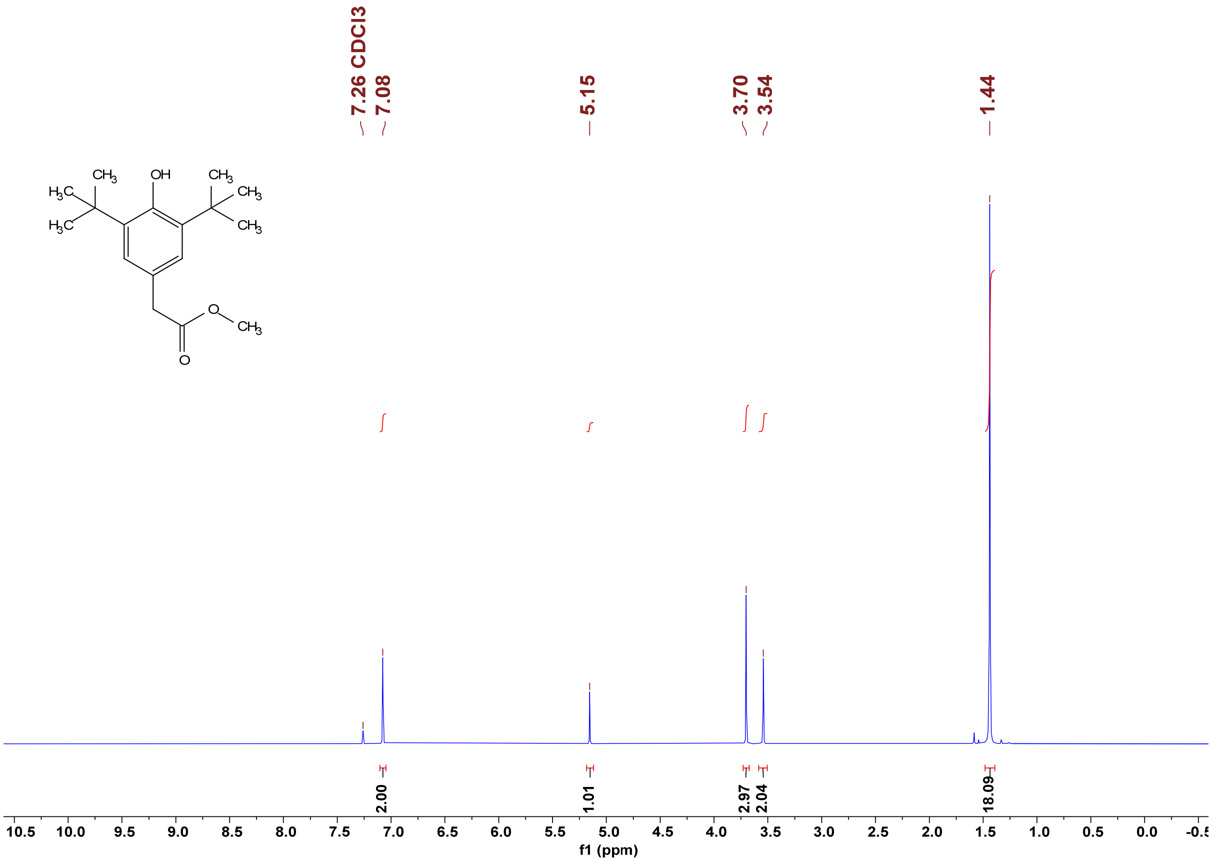

Figure S58. ^{1}H NMR spectrum of **24** in $CDCl_3$ (600 MHz) CG699

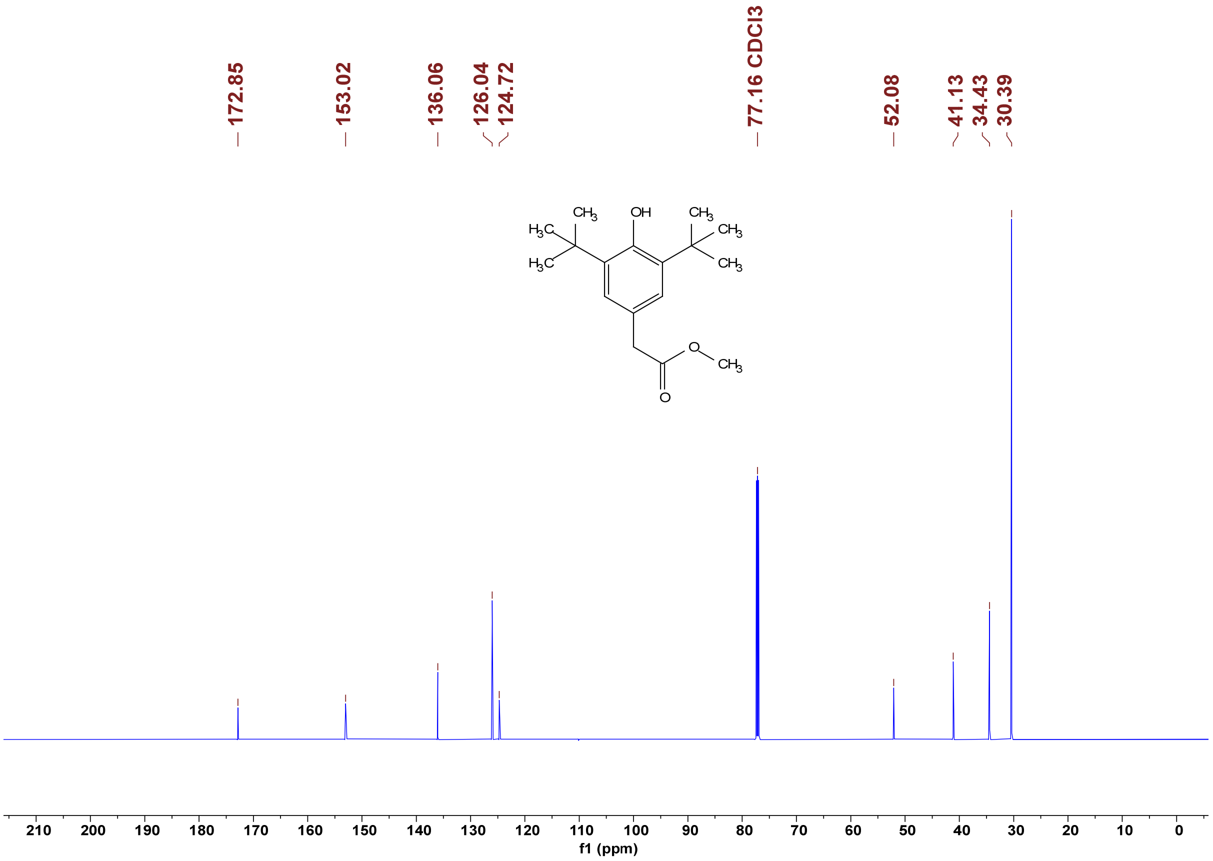

Figure S59. ^{13}C NMR spectrum of **24** in $CDCl_3$ (151 MHz) CG699

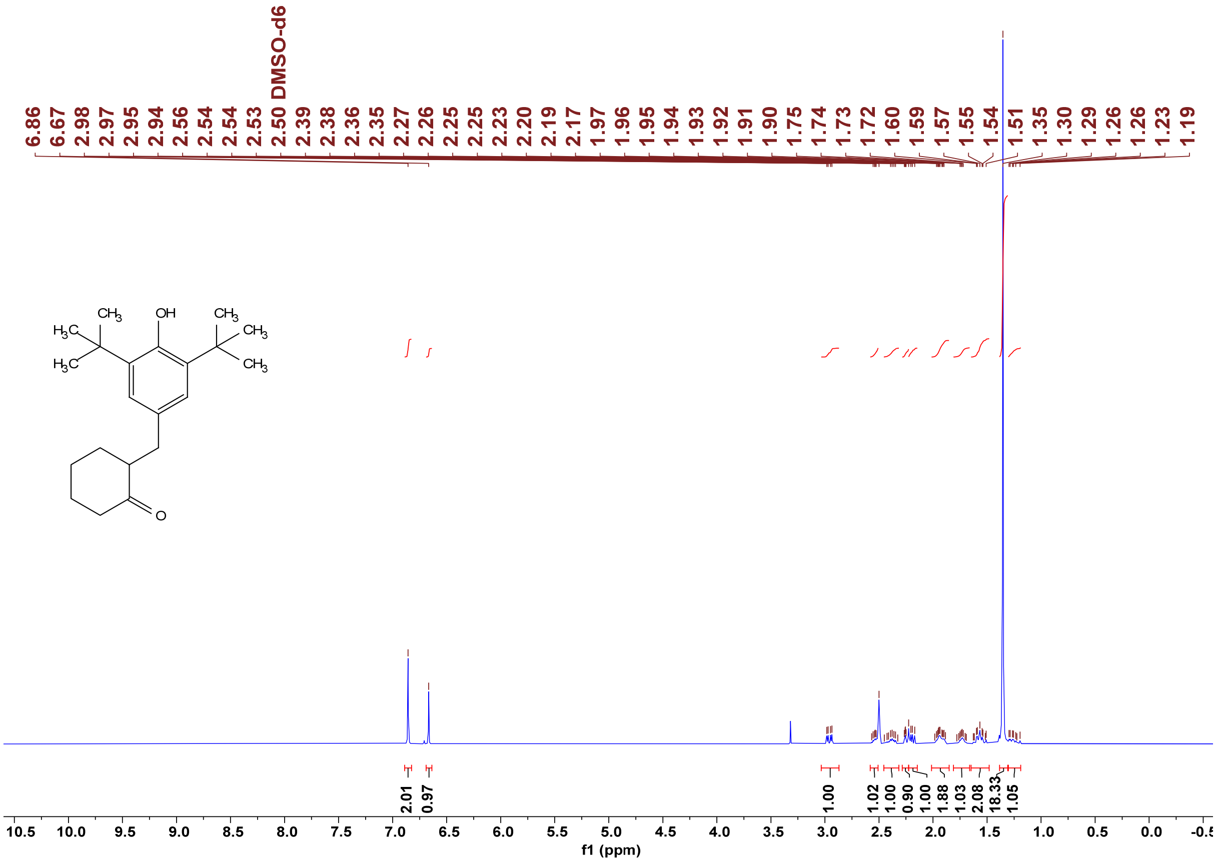

Figure S60. ^{1}H NMR spectrum of **26** in DMSO-d$_6$ (400 MHz) CG695

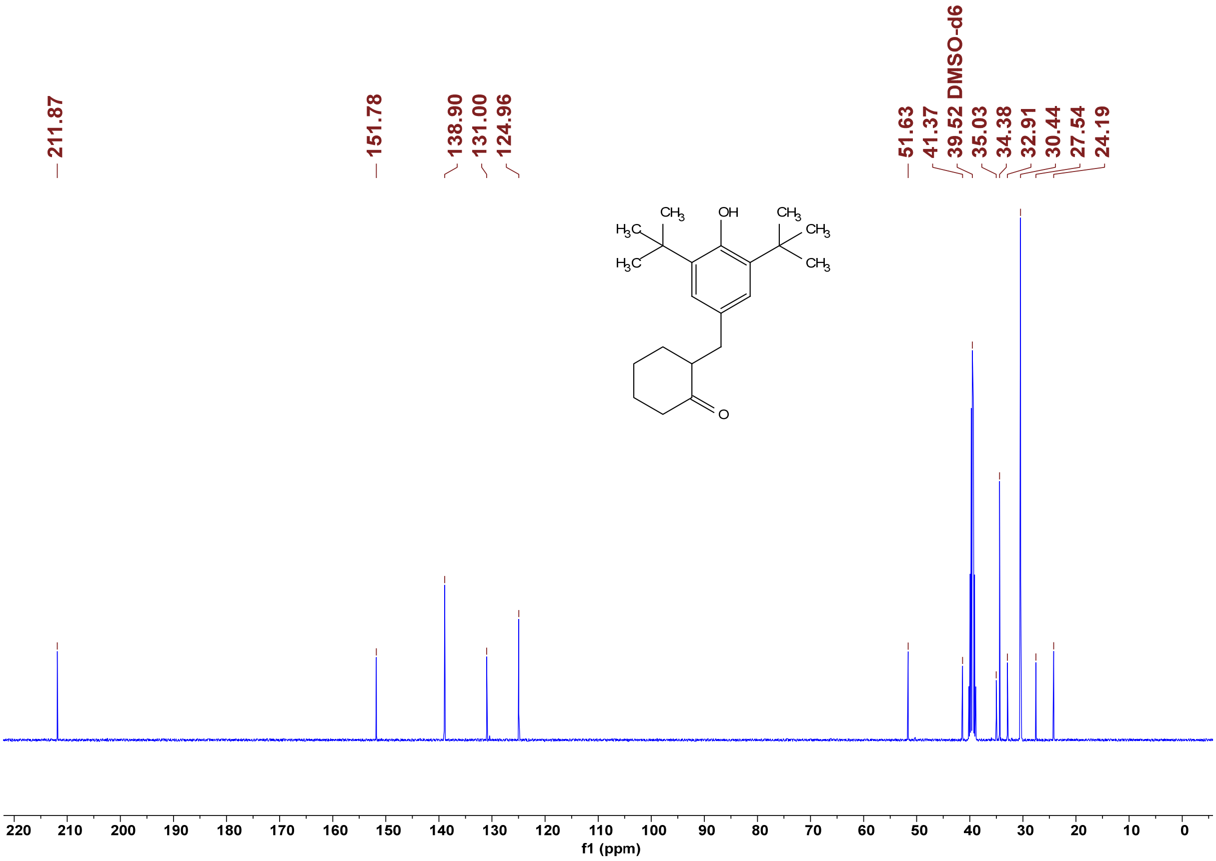

Figure S61. ^{13}C NMR spectrum of **26** in DMSO-d$_6$ (101 MHz) CG695

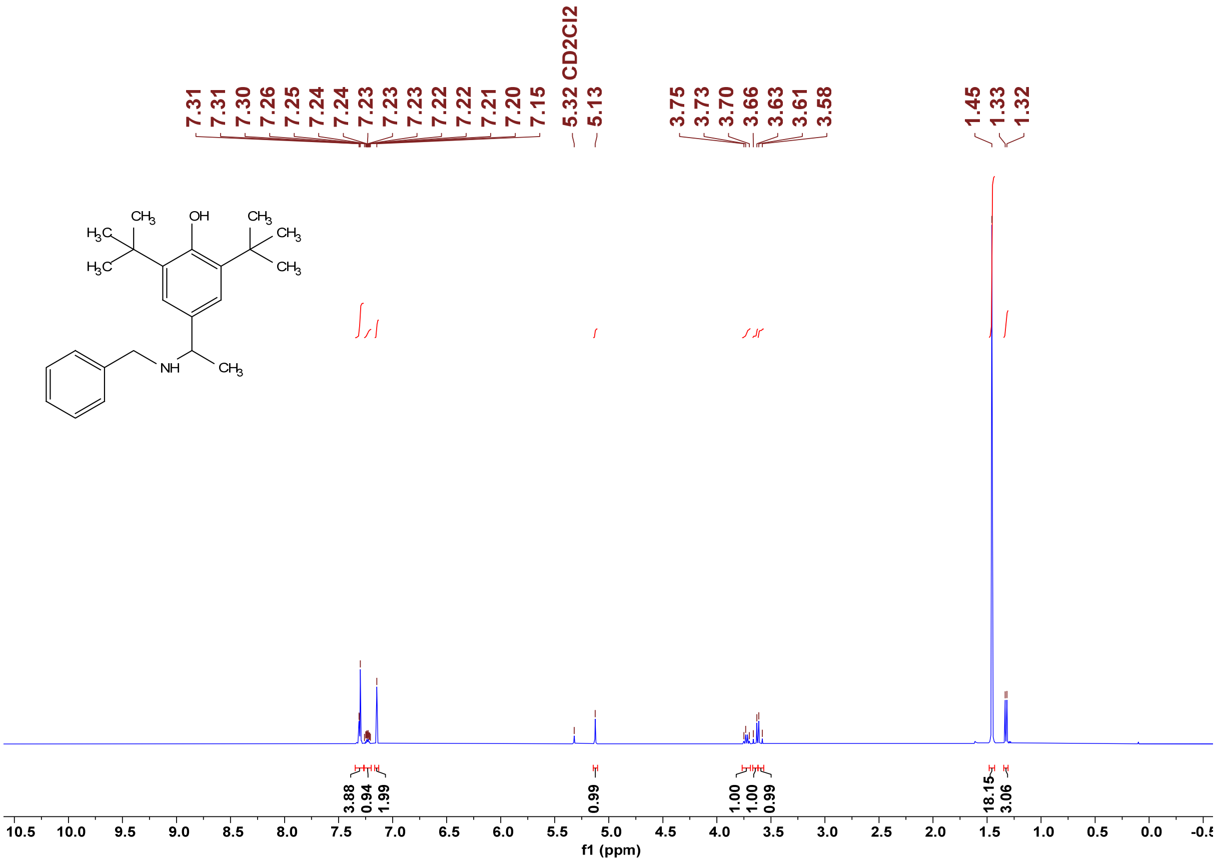

Figure S62. ^{1}H NMR spectrum of **28** in $CDCl_3$ (400 MHz) CG698

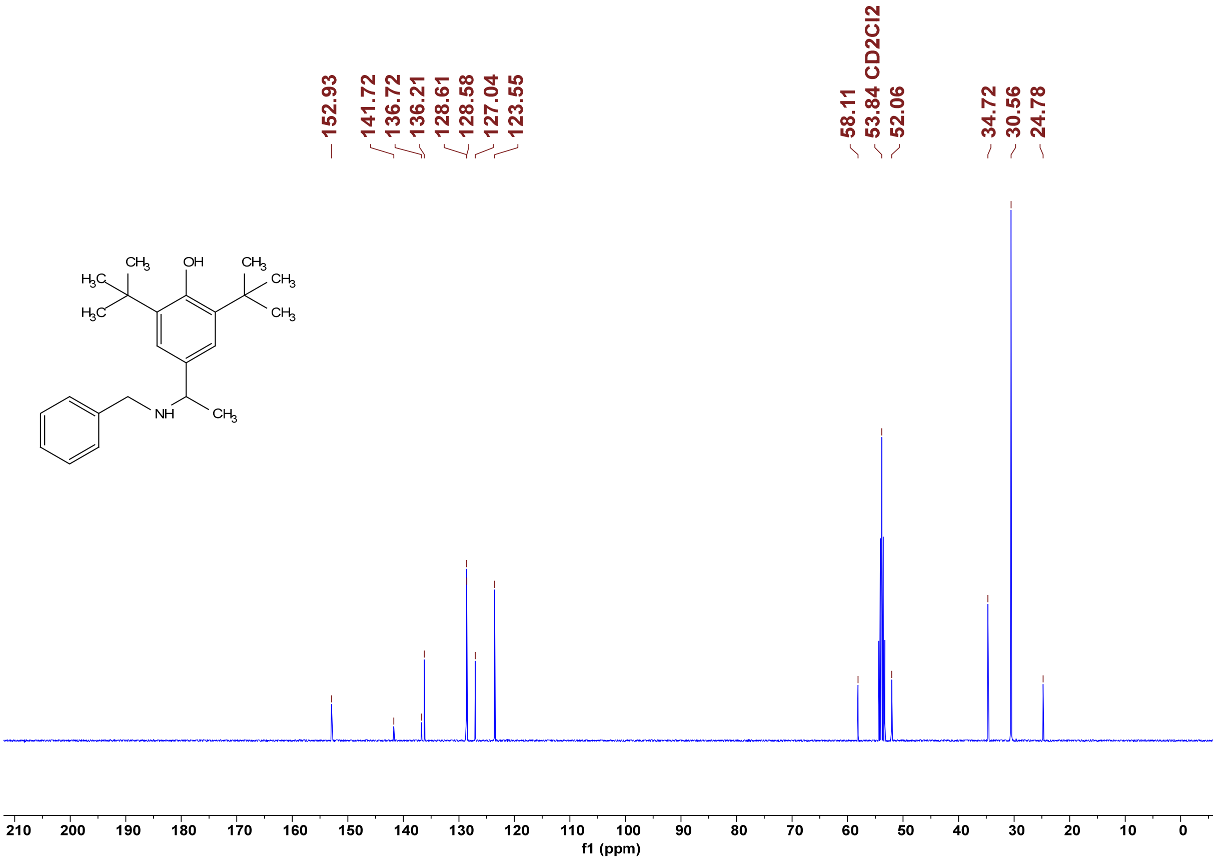

Figure S63. ^{13}C NMR spectrum of **28** in CD_2Cl_2 (101 MHz) CG698

11. Quantum-Chemical Calculations

11.1 Methyl Anion Affinities (MAAs) and Buried Volumes (%V_{bur}) of *para*-Quinone Methides 1

Methyl anion affinities (MAAs), defined as the negative free energies (–ΔG_R) of the addition of a methyl anion to an electrophile at 298.15 K (see scheme below), were calculated using established methodology.[56]

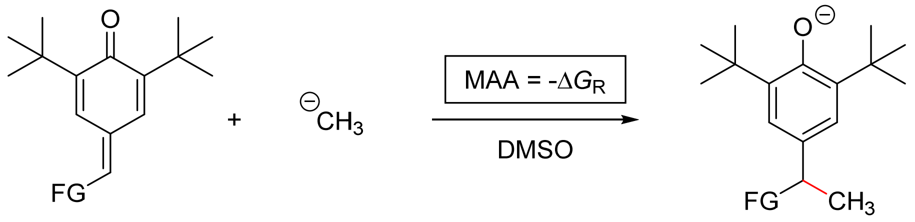

1 **1_M**

Initial conformer searches were performed using the OPLS4 force field as implemented in MacroModel (release 2021-4).[58] The Gaussian 16 software package was used to carry out DFT calculations (Rev. B.01).[59] Geometry optimizations at the DFT level were performed using the B3LYP hybrid functional in combination with the 6-31G(d,p) basis set in the gas phase.[61, 62, 63] Frequency calculations with the same method were carried out to obtain thermochemical corrections to Gibbs free energies (DG_{corr}). All structures obtained were confirmed to be true minima on the PES (Nimag=0). Using the same combination of functional and basis set, single point calculations were performed with consideration of solvation by application of the SMD solvation model for DMSO.[60] Using the SMD(DMSO)/B3LYP/6-31G(d,p) and the B3LYP/6-311++G(3df,2pd) functional/basis set combinations, single point solvation energies (ΔG_{solv}) were obtained directly. Gibbs energies (ΔG_R) were then calculated by addition of ΔG_{corr} and ΔG_{solv} to the total electronic energies obtained from single point calculations using B3LYP/6-311++G(3df,2pd).

Figure S64 depicts the *p*QMs investigated in this computational study. Energies, thermochemical corrections to energies and frontier orbital energies are given as the Boltzmann-weighted averages of conformers with at least 1 % contribution to ΔG_{298} and are summarized in Table S1.

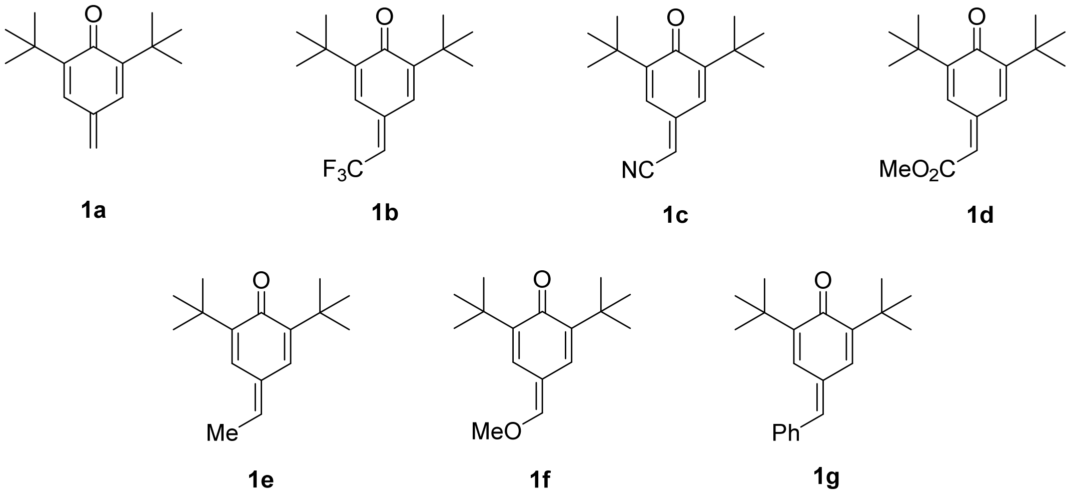

Figure S64. *p*-Quinone methides **1a**–**1g** investigated in this computational study.

Buried volumes (%V_{bur} = 100·V_{bur}/V_{sphere}) were calculated with the *SambVca 2.1* web interface using the DFT-optimized geometries as input.[64, 65, 66] The center of the sphere (r = 3.5 Å) was placed onto the reactive electrophilic carbon atom with the viewing direction along the exocyclic double bond, facing the quinone core. These two atoms, together with the (*E*)-standing adjacent carbon define the xz-plane (marked blue below). No atoms were deleted, atomic radii were scaled by 1.17 (default setting) and the mesh space value was left unchanged (0.1 Å). Hydrogen atoms were not considered in the calculation of %V_{bur}. The results incorporated in Table S1 are again given as Boltzmann-weighted averages of different conformers. The viewing direction is illustrated in Figure S65. Topographic steric maps are depicted in Figure S66.

Table S1. Summary of Boltzmann-weighted properties of *p*QMs **1** obtained from DFT calculations; (a) [SMD(DMSO)//B3LYP/6-31G(d,p)//B3LYP/6-31G(d,p)]. (b) The value for *E* of **1b** was taken from ref.[32].

*p*QM (FG)	*E*	MAA (kJ mol^{-1})	ε_{HOMO} (eV)^a	ε_{LUMO} (eV)^a	%V_{bur}
**1a** (H)	−11.17	176.04	−6.234	−2.173	38.4
**1b** (CF_3)	−11.68	196.86	−6.518	−2.640	58.1
**1c** (CN)	−11.88	175.66	−6.567	−2.970	49.4
**1d** (CO_2Me)	−12.01	172.33	−6.489	−2.839	54.8
**1e** (CH_3)	−13.68	152.99	−5.972	−2.005	48.1
**1f** (OCH_3)	−15.10	137.46	−5.559	−1.774	51.3
**1g** (Ph)	−15.58	148.58	−5.755	−2.358	57.3

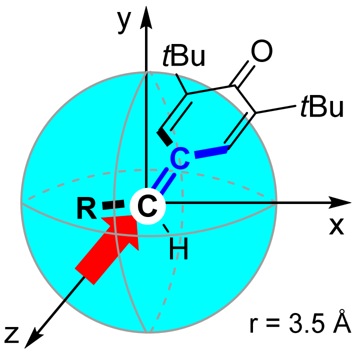

Figure S65. Viewing direction for mapping the buried volume %V_{bur}.

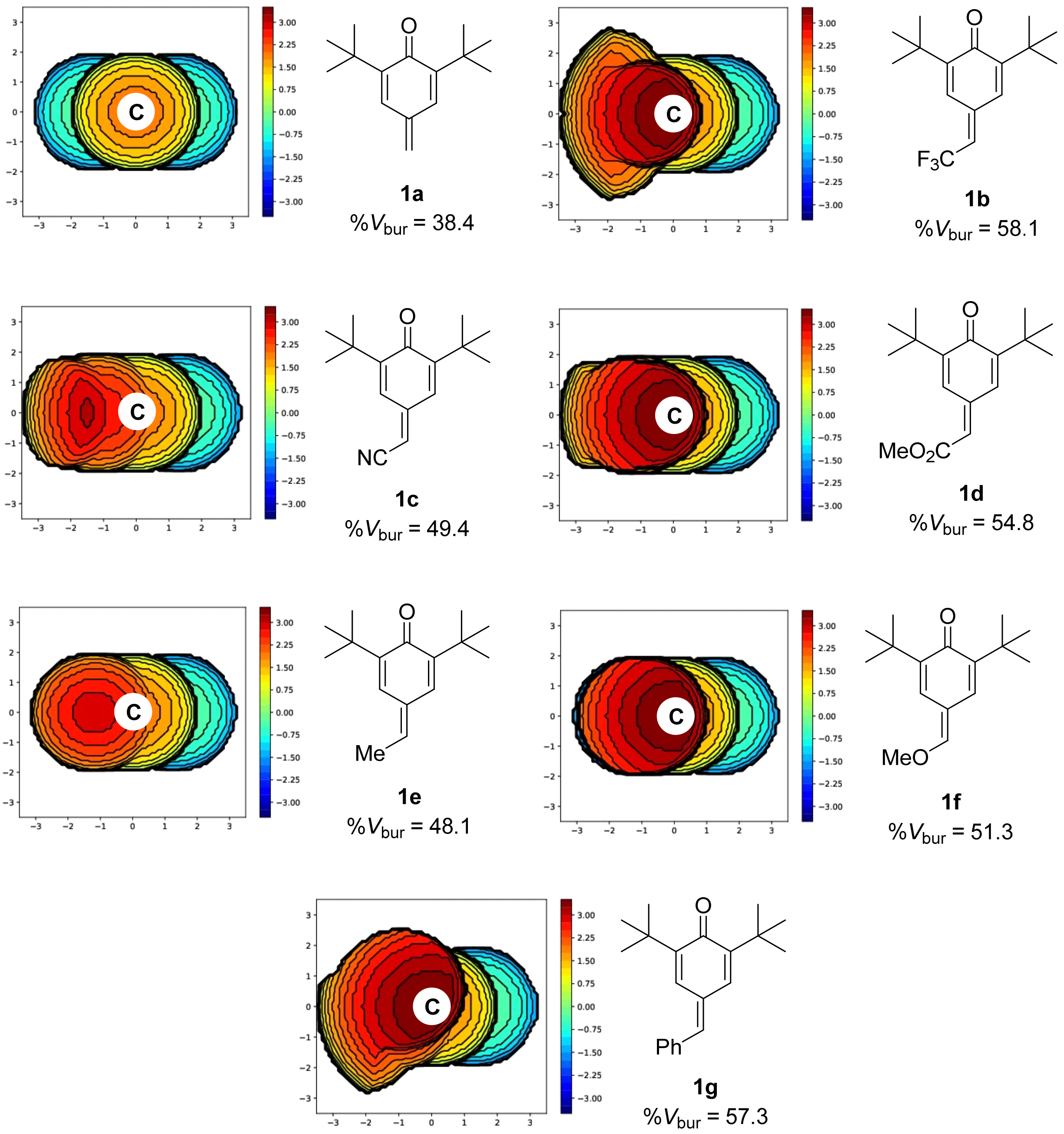

Figure S66. Topographic steric maps for the description of the vicinity of the electrophilic carbon atom in *p*QMs **1** (best conformer shown). Images were generated with the *SambVca 2.1* web interface based on geometries optimized at B3LYP/6-31G(d,p).[64, 65, 66]

11.2 Correlation Analysis

The correlation of electrophilicity *E* with MAA of *p*QMs **1** is shown below (Figure S67, with data from Table S1).

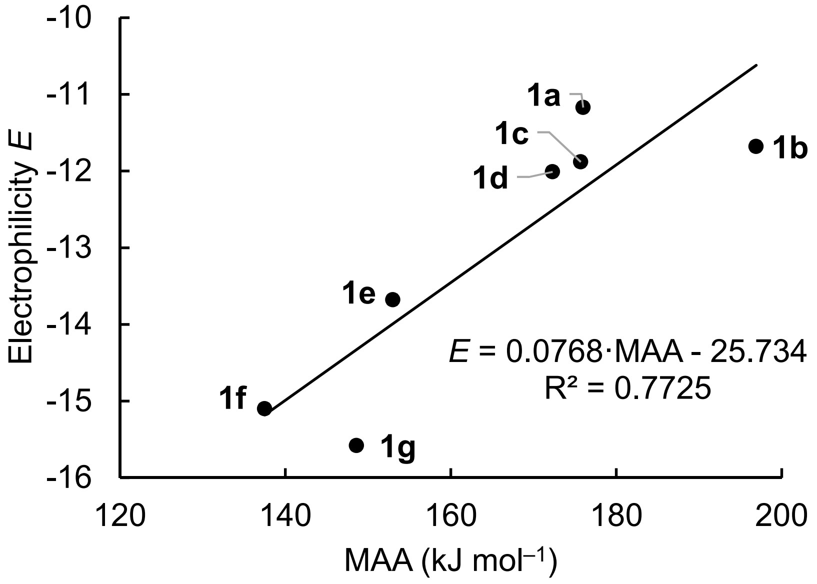

Figure S67. A plot of electrophilicity *E* vs MAA for *p*QMs **1a**–**1g**.

To enhance the predictive quality, Equation (S1) was used for a multivariate linear regression, which comprised a linear combination of MAA and $\%V_{\mathrm{bur}}$ as descriptors for the electrophilicity *E* of *p*QMs **1**.

$$E^{\mathrm{eqS1}} = a \cdot \mathrm{MAA} + b \cdot \%V_{\mathrm{bur}} + c \qquad \text{(S1)}$$

Values of the coefficients *a*, *b* and *c* were obtained through least-squares fitting with the Solver Add-in in MS Excel (MS Office Professional Plus 2016). The results are listed in Table S2 and visualized in Figure 6 (main text).

Table S2. Least-squares fitting for the description of electrophilicity *E* of *p*QMs **1** by the linear combination of MAA and $\%V_{\mathrm{bur}}$ [SumSq = $\Sigma(E^{\mathrm{eqS1}} - E)^2$].

*p*QM	*E*	MAA	$\%V_{\mathrm{bur}}$	E^{eqS1}	E^{eqS1}–*E*	$E = a \cdot \mathrm{MAA} + b \cdot \%V_{\mathrm{bur}} + \cdot c$
**1a** (H)	−11.17	176.0	38.4	−10.91	0.26	
**1b** (CF_3)	−11.68	196.9	58.1	−11.32	0.36	
**1c** (CN)	−11.88	175.7	49.4	−12.07	-0.19	*a* = 0.07763541
**1d** (CO_2Me)	−12.01	172.3	54.8	−12.89	-0.88	*b* = –0.10311350
**1e** (CH_3)	−13.68	153.0	48.1	−13.70	-0.02	*c* = –20. 61486824
**1f** (OCH_3)	−15.10	137.5	51.3	−15.23	-0.13	
**1g** (Ph)	−15.58	148.6	57.3	−14.99	0.59	$\Sigma(E^{\mathrm{eqS1}}–E)^2$ = 1.37439241

11.3 Visualization of LUMOs]

LUMOs were visualized with the program IboView (v20211019-RevA).[79] Outputs of single point calculations of *p*QM structures on the SMD(DMSO)/B3LYP/def2-TZVP//B3LYP/6-31G(d,p) level were used as inputs for IboView.[80] Figure S68 depicts the LUMOs of *p*QMs **1** (best conformer shown).

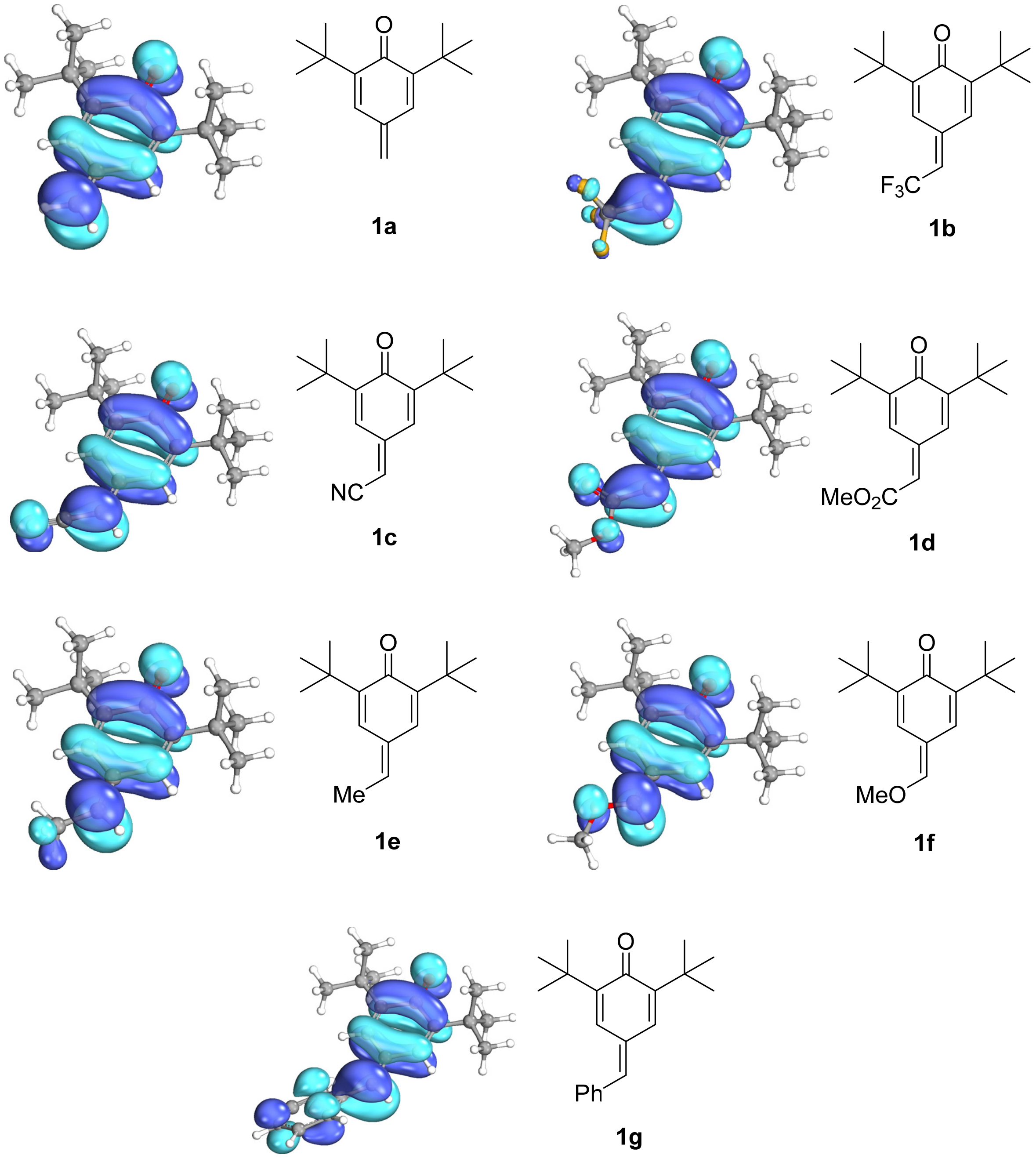

Figure S68. LUMOs of *p*QMs **1**, visualized with IboView.[79]

11.4 Summarized Data from DFT Calculations

Table S3. Summary of total electronic energies, thermochemical corrections and frontier orbital energies of gas phase-optimized structures of relevant conformers of *p*QMs **1** and methylated products **1_M**. (a) [B3LYP/6-311++G(3df,2pd)//B3LYP/6-31G(d,p)]; (b) [B3LYP/6-31G(d,p)]; (c) Single point solvation energies ΔG_{solv} were directly obtained from two subsequent single point calculations: [SMD(DMSO)//B3LYP/6-31G(d,p)//B3LYP/6-31G(d,p)] and [(a)]; (d) [SMD(DMSO)//B3LYP/6-31G(d,p)//B3LYP/6-31G(d,p)].

Identifier	Label	E_{tot} (hartree)[a]	ZPE (hartree)	Corr. ΔE (hartree)[b]	Corr. ΔH (hartree)[b]	Corr. ΔG (hartree)[b]	ΔG_{sol} (kcal mol^{-1})[c]	$-\Delta G_R$ (kJ mol^{-1})	e HOMO (hartree)[d]	e LUMO (hartree)[d]
**M**	ch3_anion	-39.85663831	0.027842	0.030708	0.031652	0.009690	-78.79	-39.97250946		
**1a**	QM_H	-660.27544112	0.335078	0.352260	0.353204	0.292118	-3.96	-659.98963385	-0.22909	-0.07986
**1b**	QM_CF3	-997.44213239	0.339842	0.360815	0.361759	0.289875	-3.77	-997.15826533	-0.23953	-0.09700
**1c**	QM_CN	-752.54913255	0.333981	0.353011	0.353955	0.288265	-6.58	-752.27135356	-0.24135	-0.10916
**1d**	QM_E_1	-888.23728576	0.378283	0.400125	0.401069	0.328094	-5.92	-887.91862598	-0.23846	-0.10435
	QM_E_2	-888.23413681	0.378346	0.400207	0.401151	0.328060	-6.30	-887.91611660	-0.23828	-0.10412
**1e**	QM_Me	-699.60736436	0.363113	0.381985	0.382929	0.318167	-5.21	-699.29750011	-0.21947	-0.07368
**1f**	QM_OMe	-774.84752368	0.368072	0.388129	0.389073	0.320668	-7.28	-774.53845722	-0.20430	-0.06521
**1g**	QM_Ph	-891.40577303	0.416823	0.438593	0.439537	0.366976	-7.34	-891.05049419	-0.21150	-0.08665
**1a_M**	QM_H_M	-700.28681211	0.372468	0.391215	0.392159	0.327580	-43.90	-700.02919193		
**1b_M**	QM_CF3_M	-1037.46848285	0.378057	0.400164	0.401109	0.328417	-41.22	-1037.20575478		
**1c_M**	QM_CN_M	-792.56689714	0.371512	0.391958	0.392902	0.324114	-42.66	-792.31076688		
**1d_M**	QM_E_M_1	-928.25002550	0.415617	0.438869	0.439814	0.364275	-44.68	-927.95695335		
	QM_E_M_2	-928.24846916	0.415628	0.438885	0.439830	0.364122	-44.64	-927.95548626		
**1e_M**	QM_Me_M	-739.61320382	0.400539	0.420657	0.421601	0.354133	-43.43	-739.32828164		
**1f_M**	QM_OMe_M_1	-814.85325410	0.405158	0.426415	0.427359	0.357092	-42.18	-814.56338090		
	QM_OMe_M_2	-814.84896155	0.405359	0.426646	0.427590	0.356900	-42.28	-814.55943971		
**1g_M**	QM_Ph_M_1	-931.40953777	0.453796	0.476962	0.477906	0.402038	-44.84	-931.07895760		
	QM_Ph_M_2	-931.40988617	0.453483	0.476824	0.477768	0.401147	-44.57	-931.07976672		

11.5 Geometries of Optimized Structures

Filenames (cf. Table S3), electronic energies E (“SCF done energy”), enthalpies H, Gibbs free energies G (in Hartree), number of imaginary frequencies (NImag) and Cartesian coordinates of atoms (in Å) for all stationary points (minima) found at the B3LYP/6-31G(d,p) level are listed below (alphabetically; by label).

ch3_anion.log

```
E =       -39.765320
H =       -39.764376
G =       -39.786338
NImag=0
C        0.0000000        0.0000000        0.1774510
H        0.0000000        0.9989880       -0.3549010
H        0.8651490       -0.4994940       -0.3549010
H       -0.8651490       -0.4994940       -0.3549010
```

QM_H.log

```
E =       -660.084316
H =       -659.731111
G =       -659.792198
NImag=0
C       -1.2932910        0.2235531        0.0000065
C       -1.2457945        1.5761389        0.0000165
C        0.0000021        2.3257213        0.0000208
C        1.2457987        1.5761392        0.0000154
C        1.2932954        0.2235534        0.0000053
C        0.0000024       -0.5349528       -0.0000017
O        0.0000027       -1.7704793       -0.0000277
C        2.6173544       -0.5596719       -0.0000016
C        2.7081052       -1.4459928       -1.2674046
C        3.8347846        0.3860321       -0.0000056
C        2.7081168       -1.4459963        1.2673981
C       -2.6173499       -0.5596721        0.0000009
C       -2.7080979       -1.4460000       -1.2673974
C       -2.7081157       -1.4459892        1.2674057
C       -3.8347797        0.3860324       -0.0000112
C        0.0000019        3.6788742        0.0000297
H       -2.1578107        2.1631630        0.0000218
H        2.1578148        2.1631634        0.0000199
H        2.6656738       -0.8330390       -2.1746372
H        1.8978065       -2.1738271       -1.2992556
H        3.6619433       -1.9853758       -1.2726138
H        3.8594501        1.0265404        0.8883208
H        3.8594443        1.0265405       -0.8883321
H        4.7530982       -0.2090903       -0.0000088
H        1.8978198       -2.1738320        1.2992537
H        2.6656919       -0.8330450        2.1746326
H        3.6619560       -1.9853776        1.2725980
H       -1.8978002       -2.1738355       -1.2992418
H       -2.6656630       -0.8330513       -2.1746333
H       -3.6619367       -1.9853816       -1.2726064
H       -1.8978201       -2.1738261        1.2992667
H       -3.6619557       -1.9853686        1.2726073
H       -2.6656909       -0.8330327        2.1746368
H       -3.8594466        1.0265463        0.8883112
H       -4.7530936       -0.2090895       -0.0000125
H       -3.8594374        1.0265356       -0.8883416
H       -0.9255957        4.2459593        0.0000337
H        0.9255995        4.2459594        0.0000329
```

QM_CF3.log

```
E =       -997.115784
H =       -996.754025
G =       -996.825909
NImag=0
C        1.5227900       -0.9470850       -0.0000020
C        0.3439440       -1.6091360       -0.0000080
C       -0.9478340       -0.9379620       -0.0000090
C       -0.9647760        0.5175600       -0.0000080
C        0.1668660        1.2594200       -0.0000020
C        1.4919970        0.5529500        0.0000030
O        2.5430930        1.1975590        0.0000100
C        0.1422330        2.7967460       -0.0000010
C        0.8504810        3.3361060       -1.2685590
C       -1.3002790        3.3406740       -0.0000080
C        0.8504680        3.3361040        1.2685660
C        2.8802010       -1.6691970        0.0000000
C        3.6833860       -1.2843370        1.2680850
```

C	2.7055760	-3.2004370	-0.0000060
C	3.6833950	-1.2843280	-1.2680760
C	-2.0749610	-1.6934120	-0.0000090
C	-3.4755560	-1.1600060	0.0000020
F	-3.7373410	-0.3917910	1.0855310
F	-3.7373250	-0.3916880	-1.0854600
F	-4.3642490	-2.1741560	-0.0000540
H	0.3180150	-2.6930690	-0.0000110
H	-1.9319630	1.0002400	-0.0000140
H	0.8051830	4.4307190	-1.2753590
H	0.3518900	2.9760940	-2.1752270
H	1.8966820	3.0330160	-1.3007280
H	-1.8602090	3.0288220	-0.8880460
H	-1.2691500	4.4343090	-0.0000070
H	-1.8602190	3.0288200	0.8880230
H	0.8051710	4.4307170	1.2753660
H	1.8966670	3.0330120	1.3007460
H	0.3518650	2.9760920	2.1752280
H	3.1379550	-1.5668730	2.1752920
H	3.8862810	-0.2141780	1.3011500
H	4.6392140	-1.8196930	1.2727140
H	3.6914430	-3.6748780	-0.0000050
H	2.1720960	-3.5549080	0.8885870
H	2.1721000	-3.5549010	-0.8886040
H	4.6392240	-1.8196820	-1.2727010
H	3.8862890	-0.2141680	-1.3011340
H	3.1379710	-1.5668600	-2.1752890
H	-2.0086010	-2.7763450	-0.0000060

QM_CN.log

E = -752.327373
H = -751.973418
G = -752.039108
NImag=0

C	-1.3989530	0.4363400	0.0000010
C	-0.7556200	1.6272020	0.0000040
C	0.6905110	1.7394420	0.0000020
C	1.4845340	0.5255430	0.0000030
C	0.9278970	-0.7086690	0.0000010
C	-0.5705980	-0.8167540	-0.0000080
O	-1.1163230	-1.9221500	-0.0000240
C	1.7699770	-1.9939190	0.0000030
C	1.4579350	-2.8277040	-1.2685780
C	3.2795860	-1.6819540	0.0000180
C	1.4579130	-2.8277120	1.2685730
C	-2.9310770	0.3216090	0.0000020
C	-3.4042130	-0.4327430	1.2684280
C	-3.6026710	1.7091060	0.0000190
C	-3.4042180	-0.4327150	-1.2684380
C	1.2630220	2.9815960	-0.0000010
C	2.6636980	3.2114190	-0.0000030
N	3.8113370	3.4171840	-0.0000050
H	-1.3116600	2.5581380	0.0000070
H	2.5607620	0.6507900	0.0000060
H	0.4117860	-3.1311160	-1.2992410
H	1.6856030	-2.2568670	-2.1754480
H	2.0813690	-3.7283900	-1.2763170
H	3.8385850	-2.6224020	0.0000190
H	3.5863640	-1.1192640	0.8880280
H	3.5863800	-1.1192570	-0.8879830
H	2.0813470	-3.7283990	1.2763170
H	0.4117630	-3.1311260	1.2992150
H	1.6855630	-2.2568810	2.1754520
H	-3.0917270	0.0959410	2.1756480
H	-4.4982230	-0.4878960	1.2743210
H	-3.0068690	-1.4468400	1.3002440
H	-3.3430320	2.2944090	0.8888230
H	-4.6891530	1.5809700	0.0000210
H	-3.3430380	2.2944280	-0.8887740
H	-3.0917390	0.0959900	-2.1756470
H	-4.4982280	-0.4878710	-1.2743270
H	-3.0068710	-1.4468100	-1.3002790
H	0.6359040	3.8688450	-0.0000020

QM_E_1.log

E = -887.965101
H = -887.564032
G = -887.637007
NImag=0

C	1.4534720	-1.0983890	0.0000020
C	0.1893960	-1.5798550	-0.0000080
C	-0.9901240	-0.7269910	-0.0000040
C	-0.7957300	0.7139300	-0.0000100
C	0.4347530	1.2808770	-0.0000010
C	1.6422320	0.3909560	0.0000260
O	2.7774760	0.8743370	0.0000670

C	0.6332280	2.8054920	-0.0000100
C	1.4124860	3.2377320	-1.2678810
C	-0.7163850	3.5503620	-0.0000560
C	1.4124120	3.2377570	1.2678990
C	2.6910350	-2.0096340	-0.0000060
C	3.5410850	-1.7441330	-1.2680400
C	3.5410600	-1.7441950	1.2680580
C	2.2986780	-3.5003720	-0.0000460
C	-2.2161650	-1.3286920	0.0000020
C	-3.5309500	-0.6597470	0.0000060
O	-3.7621260	0.5370050	0.0000170
O	-4.5135860	-1.5956100	-0.0000040
C	-5.8520020	-1.0763090	0.0000040
H	0.0060420	-2.6486030	-0.0000180
H	-1.6938380	1.3141000	-0.0000200
H	1.5245690	4.3275480	-1.2750730
H	0.8680710	2.9525140	-2.1748530
H	2.4045510	2.7878130	-1.2991320
H	-1.3159840	3.3197380	-0.8866180
H	-0.5303680	4.6288870	-0.0000630
H	-1.3160330	3.3197590	0.8864780
H	1.5245000	4.3275740	1.2750740
H	2.4044720	2.7878330	1.2992190
H	0.8679400	2.9525610	2.1748430
H	2.9582240	-1.9386660	-2.1751160
H	4.4074900	-2.4147870	-1.2774100
H	3.8990500	-0.7153750	-1.2970870
H	2.9581820	-1.9387730	2.1751130
H	3.8990230	-0.7154380	1.2971630
H	4.4074650	-2.4148490	1.2774110
H	1.7199710	-3.7753090	-0.8885470
H	1.7199610	-3.7753550	0.8884330
H	3.2068490	-4.1107490	-0.0000570
H	-2.2641990	-2.4132580	0.0000030
H	-6.5056370	-1.9480830	0.0000350
H	-6.0276550	-0.4638510	-0.8879830
H	-6.0276280	-0.4638040	0.8879630

QM_E_2.log

E = -887.961559
H = -887.560408
G = -887.633498
NImag=0

C	1.6311730	-0.8895500	0.0000000
C	0.4962860	-1.6256760	0.0000000
C	-0.8406900	-1.0459630	0.0000000
C	-0.9489890	0.4040930	0.0000000
C	0.1345340	1.2176280	0.0000000
C	1.5031470	0.6036070	0.0000010
O	2.5098420	1.3173830	0.0000030
C	0.0081880	2.7501250	-0.0000020
C	-1.4668420	3.1974440	-0.0000040
C	0.6793940	3.3361980	1.2681090
C	0.6793970	3.3361950	-1.2681130
C	3.0325640	-1.5212330	0.0000010
C	3.8088580	-1.0840370	-1.2679660
C	3.8088560	-1.0840370	1.2679690
C	2.9609980	-3.0608940	0.0000010
C	-1.8957170	-1.9147160	-0.0000010
C	-3.3562450	-1.6948820	-0.0000020
O	-4.1422460	-2.6250540	-0.0000110
O	-3.7549130	-0.4003970	0.0000090
C	-5.1796310	-0.2031330	0.0000080
H	0.5438820	-2.7088940	-0.0000010
H	-1.9471690	0.8127180	-0.0000020
H	-2.0040570	2.8468090	0.8876040
H	-2.0040550	2.8468070	-0.8876110
H	-1.5102540	4.2909190	-0.0000050
H	1.7429180	3.1011750	1.2999850
H	0.2055120	2.9450130	2.1751780
H	0.5632610	4.4256530	1.2740920
H	0.5632620	4.4256500	-1.2740990
H	0.2055170	2.9450060	-2.1751810
H	1.7429200	3.1011730	-1.2999860
H	4.7979180	-1.5552180	-1.2728310
H	3.9402610	-0.0027650	-1.3005230
H	3.2831990	-1.4016300	-2.1752900
H	3.9402610	-0.0027650	1.3005250
H	4.7979150	-1.5552200	1.2728360
H	3.2831960	-1.4016290	2.1752920
H	2.4523640	-3.4504060	-0.8884260
H	2.4523580	-3.4504060	0.8884240
H	3.9767000	-3.4678470	0.0000040
H	-1.6762760	-2.9781730	-0.0000030
H	-5.6292210	-0.6525910	0.8883890
H	-5.6292170	-0.6525680	-0.8883880
H	-5.3243430	0.8767270	0.0000210

QM_Me.log

E = -699.406269
H = -699.023339
G = -699.088102
NImag=0

C	-1.3506480	0.1776487	0.0000034
C	-1.0447317	1.4970017	0.0000027
C	0.3165361	2.0065181	-0.0000002
C	1.3935285	1.0331836	0.0000022
C	1.1802652	-0.3053722	0.0000011
C	-0.2296793	-0.8103446	-0.0000090
O	-0.4610854	-2.0258837	-0.0000202
C	2.3337061	-1.3245702	0.0000014
C	2.2544956	-2.2123707	1.2671852
C	2.2545129	-2.2123467	-1.2672005
C	3.7102224	-0.6309595	0.0000182
C	-2.8005034	-0.3379907	0.0000051
C	-3.0586510	-1.1910653	-1.2670967
C	-3.0586473	-1.1910858	1.2670937
C	-3.8163316	0.8207040	0.0000180
C	0.5163495	3.3536057	-0.0000034
C	1.8075344	4.1045043	-0.0000077
H	-1.8326566	2.2431977	0.0000059
H	2.4064475	1.4142185	0.0000047
H	1.3186152	-2.7691942	1.3005249
H	2.3334852	-1.6027222	2.1742868
H	3.0862450	-2.9258896	1.2702230
H	3.0862629	-2.9258650	-1.2702416
H	1.3186331	-2.7691701	-1.3005621
H	2.3335128	-1.6026805	-2.1742892
H	3.8590026	-0.0076695	0.8886133
H	4.4965250	-1.3922187	0.0000282
H	3.8590247	-0.0076702	-0.8885737
H	-2.9063910	-0.5952341	-2.1740754
H	-4.0959840	-1.5448274	-1.2698031
H	-2.3972371	-2.0564306	-1.3030042
H	-4.0959815	-1.5448443	1.2697983
H	-2.9063827	-0.5952696	2.1740816
H	-2.3972362	-2.0564534	1.3029854
H	-3.7188735	1.4541450	-0.8884046
H	-3.7188500	1.4541439	0.8884388
H	-4.8308981	0.4104504	0.0000309
H	-0.3766664	3.9786771	-0.0000050
H	1.8653814	4.7618660	0.8774047
H	1.8654027	4.7618190	-0.8774544
H	2.6893791	3.4623923	0.0000196

QM_OMe.log

E = -774.615296
H = -774.226223
G = -774.294629
NImag=0

C	1.2136660	-0.8000890	0.0000010
C	0.1512550	-1.6448940	0.0000020
C	-1.2153890	-1.1793560	0.0000020
C	-1.4649600	0.2424820	0.0000020
C	-0.4595040	1.1549000	0.0000010
C	0.9545920	0.6695020	0.0000000
O	1.8969710	1.4760750	-0.0000010
C	-0.7263280	2.6703350	0.0000000
C	-0.1104900	3.3151480	-1.2668650
C	-2.2353380	2.9851310	0.0000010
C	-0.1104880	3.3151500	1.2668630
C	2.6680450	-1.3032540	0.0000000
C	3.4026080	-0.7977020	1.2664240
C	2.7397490	-2.8429970	0.0000010
C	3.4026050	-0.7977050	-1.2664280
C	-2.2235430	-2.0982420	0.0000020
O	-3.5192020	-1.7482430	0.0000050
C	-4.4644230	-2.8198770	-0.0000050
H	0.3026100	-2.7197410	0.0000020
H	-2.5017330	0.5546820	0.0000020
H	-0.5559430	2.8909860	-2.1738130
H	0.9675310	3.1605110	-1.3015210
H	-0.3103040	4.3928030	-1.2684040
H	-2.7398610	2.5887610	-0.8879180
H	-2.3768050	4.0704950	0.0000000
H	-2.7398590	2.5887620	0.8879220
H	0.9675330	3.1605130	1.3015180
H	-0.5559390	2.8909890	2.1738120
H	-0.3103020	4.3928050	1.2684000
H	2.9126720	-1.1688350	2.1737080
H	3.4207440	0.2912930	1.3000690
H	4.4351430	-1.1657570	1.2688190
H	3.7888720	-3.1549510	0.0000010

H	2.2690540	-3.2781910	0.8885940
H	2.2690530	-3.2781930	-0.8885890
H	3.4207400	0.2912910	-1.3000740
H	2.9126670	-1.1688400	-2.1737100
H	4.4351410	-1.1657580	-1.2688230
H	-2.0095820	-3.1670960	0.0000020
H	-5.4516880	-2.3582620	0.0000090
H	-4.3521160	-3.4410730	0.8958550
H	-4.3521280	-3.4410460	-0.8958860

QM_Ph.log

E = -891.149880
H = -890.710343
G = -890.782904
NImag=0

C	1.9062670	-1.0404210	-0.0234880
C	0.6745100	-1.6032240	0.0381650
C	-0.5579360	-0.8372800	0.0560680
C	-0.4438560	0.6070470	0.1074530
C	0.7477580	1.2554200	0.0682300
C	2.0030140	0.4496750	-0.0505840
O	3.1032250	1.0089670	-0.1478990
C	0.8539950	2.7880720	0.1543250
C	1.7137310	3.1930500	1.3778850
C	1.4957110	3.3435750	-1.1424230
C	-0.5288840	3.4494360	0.3147310
C	3.1975530	-1.8750290	-0.0759010
C	3.9566850	-1.5906560	-1.3960390
C	4.1006380	-1.5258690	1.1333680
C	2.9027850	-3.3869010	-0.0195770
C	-1.7460110	-1.5217800	0.0623780
C	-3.1204400	-1.0341730	-0.0228400
C	-3.5040560	0.1067570	-0.7577530
C	-4.8367010	0.5077920	-0.8060530
C	-5.8190170	-0.2193370	-0.1305110
C	-5.4617190	-1.3682390	0.5795390
C	-4.1327880	-1.7767650	0.6206740
H	0.5648470	-2.6826070	0.0459700
H	-1.3600330	1.1684090	0.2266320
H	2.7256730	2.7967670	1.2984570
H	1.2643500	2.8262840	2.3075240
H	1.7714630	4.2853870	1.4428660
H	1.5522890	4.4367220	-1.0873550
H	2.5012510	2.9486400	-1.2840630
H	0.8899340	3.0838420	-2.0178700
H	-1.0380190	3.1258270	1.2289470
H	-1.1865350	3.2444950	-0.5371170
H	-0.4037050	4.5349760	0.3755960
H	3.3396990	-1.8527640	-2.2627940
H	4.2355270	-0.5399850	-1.4710730
H	4.8675560	-2.1986970	-1.4373960
H	3.5885420	-1.7456260	2.0768760
H	4.3813880	-0.4729270	1.1252390
H	5.0140790	-2.1304850	1.0999450
H	2.2994770	-3.7225420	-0.8701280
H	2.3874270	-3.6728590	0.9039000
H	3.8474270	-3.9385810	-0.0516100
H	-1.6655700	-2.6039780	0.1636070
H	-2.7608700	0.6524080	-1.3276950
H	-5.1115290	1.3841400	-1.3855720
H	-6.8570140	0.0966730	-0.1713800
H	-6.2209610	-1.9486890	1.0951490
H	-3.8599270	-2.6734630	1.1706620

QM_H_M.log

E = -700.074412
H = -699.682253
G = -699.746832
NImag=0

C	1.2396320	-0.1171382	-0.0816651
C	1.1966703	1.2616420	-0.2790025
C	0.0000235	1.9844209	-0.3785345
C	-1.1966429	1.2616746	-0.2790012
C	-1.2396424	-0.1171040	-0.0816627
C	-0.0000151	-0.8727188	0.0334005
O	-0.0000327	-2.1449713	0.2186068
C	-2.5784823	-0.8766290	0.0111629
C	-3.8046722	0.0448364	-0.1351718
C	-2.6520815	-1.9409305	-1.1114739
C	-2.6805446	-1.5845359	1.3846789
C	2.5784514	-0.8766999	0.0111565
C	2.6805003	-1.5846064	1.3846735
C	2.6520165	-1.9410056	-1.1114785
C	3.8046667	0.0447309	-0.1351857
C	0.0000444	3.4881920	-0.5431314
C	0.0001053	4.2647365	0.7882786

```
H        2.1276152     1.8194418    -0.3654317
H       -2.1275725     1.8195002    -0.3654293
H       -3.8427327     0.8064465     0.6524790
H       -4.7235073    -0.5512537    -0.0641129
H       -3.8177337     0.5600407    -1.1027978
H       -1.7846332    -2.5988397    -1.0362922
H       -2.6497875    -1.4589260    -2.0970941
H       -3.5737807    -2.5353118    -1.0276555
H       -1.8142459    -2.2351773     1.5151074
H       -3.6026192    -2.1805913     1.4507690
H       -2.6969966    -0.8459608     2.1958270
H        3.6025557    -2.1806924     1.4507587
H        1.8141811    -2.2352190     1.5151097
H        2.6969826    -0.8460299     2.1958197
H        3.5737022    -2.5354090    -1.0276658
H        2.6497265    -1.4590034    -2.0970998
H        1.7845533    -2.5988942    -1.0362887
H        4.7234850    -0.5513865    -0.0641406
H        3.8427581     0.8063350     0.6524698
H        3.8177330     0.5599414    -1.1028083
H       -0.8782152     3.8009231    -1.1268561
H        0.8782717     3.8008958    -1.1269201
H        0.8821636     4.0064033     1.3846627
H       -0.8819208     4.0064347     1.3847238
H        0.0001197     5.3529956     0.6318898
```

QM_CF3_M.log

```
E =      -1037.120044
H =      -1036.718936
G =      -1036.791627
NImag=0
C       -0.7021537     1.2937682     0.0842115
C        0.6177092     0.9099876     0.2969066
C        1.0100494    -0.4336927     0.4066479
C        0.0122503    -1.4116275     0.2931727
C       -1.3293529    -1.1084130     0.0848844
C       -1.7478245     0.2827687    -0.0284548
O       -2.9748668     0.6026436    -0.2156998
C       -2.3976809    -2.2145427    -0.0236189
C       -3.4538013    -2.0291500     1.0937256
C       -3.0998386    -2.1274230    -1.4011557
C       -1.8101276    -3.6322393     0.1151690
C       -1.0959074     2.7799674    -0.0339063
C       -2.1087385     3.1401151     1.0810540
C        0.1079966     3.7325049     0.1005553
C       -1.7503602     3.0372651    -1.4134190
C        2.4441457    -0.8416353     0.6868464
C        3.0108474    -0.2615846     1.9987446
C        3.4003194    -0.5355992    -0.4589992
F        3.6034238     0.7930153    -0.6451562
F        4.6403675    -1.0724831    -0.2310857
F        2.9845175    -1.0438928    -1.6374036
H        1.3853487     1.6739777     0.3730124
H        0.3187005    -2.4522642     0.3791313
H       -3.8809369    -1.0278736     1.0199404
H       -2.9917775    -2.1473555     2.0816745
H       -4.2538946    -2.7778611     1.0023925
H       -3.5166008    -1.1271034    -1.5283441
H       -3.9048857    -2.8727590    -1.4767714
H       -2.3838724    -2.3202853    -2.2094318
H       -1.3225976    -3.7820899     1.0857386
H       -1.0757077    -3.8513687    -0.6687013
H       -2.6149380    -4.3733307     0.0315870
H       -1.6461352     3.0295160     2.0696870
H       -2.9685239     2.4716770     1.0149914
H       -2.4454555     4.1820443     0.9802412
H        0.6057464     3.6310352     1.0720904
H       -0.2349448     4.7710357     0.0116596
H        0.8572698     3.5630686    -0.6813100
H       -2.6049720     2.3698916    -1.5340318
H       -1.0325105     2.8457808    -2.2203208
H       -2.0845434     4.0814123    -1.4977173
H        2.4721850    -1.9365945     0.7596443
H        4.0360437    -0.5999734     2.1856771
H        2.3769103    -0.5762401     2.8312002
H        3.0090719     0.8313545     1.9720198
```

QM_CN_M_1.log

```
E =      -792.322395
H =      -791.929493
G =      -791.998281
NImag=0
C        0.3445620     1.1711940     0.0403740
C        1.3854620     0.2519950     0.0980840
C        1.1692720    -1.1339790     0.1051630
C       -0.1515330    -1.5956400     0.0412540
```

C	-1.2464000	-0.7393010	-0.0148620
C	-1.0377760	0.7037320	-0.0143150
O	-2.0206610	1.5229990	-0.0620210
C	-2.6886820	-1.2798230	-0.0766570
C	-3.3766970	-0.7844530	-1.3724480
C	-2.7526120	-2.8196050	-0.0714140
C	-3.4883060	-0.7702710	1.1476210
C	0.6125830	2.6894620	0.0312960
C	2.1133200	3.0337220	0.0934600
C	0.0392460	3.3107480	-1.2661710
C	-0.0742640	3.3463870	1.2540540
C	2.3181200	-2.1216080	0.2410590
C	2.9994490	-2.0891910	1.6322050
C	3.3444850	-1.9537200	-0.8098030
N	4.1668370	-1.8293410	-1.6228420
H	2.4130290	0.6064030	0.1294970
H	-0.3104810	-2.6722610	0.0414730
H	-2.8632410	-1.1835150	-2.2557280
H	-3.3409030	0.3053950	-1.4047420
H	-4.4236430	-1.1183080	-1.4094900
H	-2.2343390	-3.2556130	-0.9334360
H	-3.8000330	-3.1429160	-0.1168100
H	-2.3143670	-3.2466470	0.8383700
H	-3.4498630	0.3197630	1.1731570
H	-3.0589760	-1.1636150	2.0774400
H	-4.5360220	-1.0993410	1.0932840
H	2.6646220	2.6293730	-0.7630170
H	2.5847570	2.6549480	1.0079830
H	2.2397520	4.1234830	0.0843130
H	0.5553060	2.9059490	-2.1451620
H	0.1739700	4.4018050	-1.2670880
H	-1.0235400	3.0757940	-1.3395000
H	0.3601270	2.9679680	2.1877060
H	-1.1394810	3.1111240	1.2381320
H	0.0614100	4.4372460	1.2361990
H	1.9098730	-3.1307190	0.0928360
H	3.4169200	-1.0980420	1.8283550
H	3.8052270	-2.8287710	1.7119470
H	2.2459470	-2.2960100	2.3969410

QM_E_M_1.log

E = -927.954915

H = -927.515101

G = -927.590639

NImag=0

C	1.3544520	-1.0581760	-0.1164670
C	0.0587140	-1.4210280	-0.4707660
C	-0.9683090	-0.4867230	-0.6601330
C	-0.6659250	0.8688910	-0.4572440
C	0.6043750	1.3096860	-0.1000440
C	1.6853600	0.3476590	0.0819840
O	2.8694260	0.7198070	0.4034770
C	0.9036960	2.8071490	0.1141000
C	1.4006290	3.0369990	1.5627060
C	2.0023920	3.2653020	-0.8765140
C	-0.3311780	3.7018100	-0.1061520
C	2.4622940	-2.1133770	0.0735800
C	1.9664960	-3.5533770	-0.1619770
C	3.6164320	-1.8426740	-0.9227050
C	3.0123170	-2.0351450	1.5190420
C	-2.3665370	-0.9404940	-1.0708060
C	-2.8065750	-0.4211810	-2.4495630
C	-3.3606730	-0.5129130	-0.0052610
O	-4.0408160	0.4958430	-0.0159850
O	-3.3906930	-1.3932590	1.0345450
C	-4.1858570	-0.9842100	2.1485050
H	-0.1850610	-2.4711820	-0.6168260
H	-1.4698300	1.5898390	-0.5785890
H	2.2707860	2.4058000	1.7490710
H	0.6135710	2.7802130	2.2824240
H	1.6711790	4.0912040	1.7194210
H	2.8870550	2.6398330	-0.7474110
H	2.2686890	4.3181340	-0.7038290
H	1.6483310	3.1722940	-1.9106760
H	-1.1449600	3.4574260	0.5859690
H	-0.7222710	3.6185930	-1.1267530
H	-0.0581070	4.7516940	0.0591940
H	2.7951310	-4.2563590	-0.0094120
H	1.5938980	-3.6984640	-1.1827860
H	1.1650030	-3.8304010	0.5326470
H	3.2656340	-1.9567840	-1.9559060
H	4.4421420	-2.5518050	-0.7658650
H	3.9796780	-0.8233850	-0.7826460
H	2.2273630	-2.2898370	2.2418390
H	3.3592930	-1.0196070	1.7158770
H	3.8436990	-2.7411190	1.6591490
H	-2.3654070	-2.0366740	-1.0791380

H	-2.0823490	-0.7360850	-3.2062020
H	-2.8538480	0.6702320	-2.4506420
H	-3.7978090	-0.7997990	-2.7302000
H	-4.1040120	-1.7859520	2.8844370
H	-5.2310020	-0.8399890	1.8572180
H	-3.8127490	-0.0461560	2.5696290

QM_E_M_2.log

E = -927.953300
H = -927.513470
G = -927.589178
NImag=0

C	-0.4321260	1.2896880	0.1420440
C	0.7594080	0.6303270	0.4249540
C	0.8480080	-0.7673730	0.5270200
C	-0.3220140	-1.5104800	0.3152060
C	-1.5516720	-0.9240370	0.0330520
C	-1.6577350	0.5260820	-0.0655220
O	-2.7736760	1.1030610	-0.3212320
C	-2.8204950	-1.7753060	-0.1733890
C	-3.3921830	-1.5230380	-1.5904250
C	-2.5571200	-3.2872090	-0.0353560
C	-3.8869540	-1.3837110	0.8788120
C	-0.4940340	2.8272350	0.0405820
C	-1.4833530	3.3787610	1.0966850
C	0.8712430	3.5002960	0.2797400
C	-0.9820160	3.2361460	-1.3710310
C	2.1574960	-1.4757920	0.8644710
C	2.7555960	-1.0500810	2.2190540
C	3.1433620	-1.3198590	-0.2835040
O	3.3251800	-2.1186090	-1.1813500
O	3.8171060	-0.1333130	-0.2313710
C	4.6611580	0.1319100	-1.3530970
H	1.6680260	1.2072430	0.5680720
H	-0.2471680	-2.5932410	0.3860140
H	-2.6776170	-1.8501200	-2.3556150
H	-3.5820420	-0.4559940	-1.7164280
H	-4.3272640	-2.0823420	-1.7390880
H	-2.1804540	-3.5487180	0.9604420
H	-1.8334540	-3.6477450	-0.7754190
H	-3.4930010	-3.8387690	-0.1902940
H	-4.0848630	-0.3131060	0.8077430
H	-3.5300160	-1.6131940	1.8904870
H	-4.8202480	-1.9415850	0.7145900
H	-1.5850540	4.4697730	1.0041620
H	-1.1248340	3.1580790	2.1097450
H	-2.4574690	2.9082760	0.9547910
H	0.7633860	4.5890210	0.1945540
H	1.6209830	3.1831770	-0.4543860
H	1.2663440	3.2835820	1.2790150
H	-0.2643350	2.9100210	-2.1339040
H	-1.0846240	4.3283910	-1.4476980
H	-1.9462340	2.7647720	-1.5676150
H	1.9518030	-2.5510870	0.8921150
H	3.6905170	-1.5818920	2.4399360
H	2.0356220	-1.2677980	3.0136700
H	2.9640530	0.0219380	2.2365650
H	5.1225320	1.1013920	-1.1574850
H	4.0787260	0.1704290	-2.2780140
H	5.4283960	-0.6409580	-1.4601100

QM_Me_M_1.log

E = -739.390744
H = -738.969143
G = -739.036612
NImag=0

C	0.8022340	-0.9044230	0.0000000
C	1.4930440	0.3050520	-0.0000030
C	0.8533070	1.5539360	-0.0000070
C	-0.5473960	1.5531690	-0.0000070
C	-1.3121030	0.3876520	-0.0000030
C	-0.6548210	-0.9104380	0.0000000
O	-1.3246790	-2.0074760	0.0000020
C	-2.8534090	0.4345580	-0.0000020
C	-3.3955130	-0.2825210	1.2610870
C	-3.4122860	1.8705960	-0.0000030
C	-3.3955150	-0.2825250	-1.2610880
C	1.5461050	-2.2557950	0.0000020
C	1.1537310	-3.0650910	1.2607050
C	1.1537310	-3.0650950	-1.2606980
C	3.0786440	-2.0985410	0.0000020
C	1.6368520	2.8580300	-0.0000070
C	2.5019590	3.0305780	1.2653000
C	2.5020020	3.0305550	-1.2652880
H	2.5809090	0.2912000	-0.0000020
H	-1.0469840	2.5204700	-0.0000100

H	-3.0824000	0.2490560	2.1683320
H	-2.9994400	-1.2989630	1.2908730
H	-4.4948840	-0.3158190	1.2510740
H	-3.0976720	2.4340950	0.8862200
H	-4.5092600	1.8375750	0.0000010
H	-3.0976790	2.4340900	-0.8862320
H	-2.9994410	-1.2989660	-1.2908720
H	-3.0824050	0.2490500	-2.1683350
H	-4.4948860	-0.3158240	-1.2510720
H	0.0686650	-3.1780710	1.2875160
H	1.4805830	-2.5430180	2.1685970
H	1.6275620	-4.0576160	1.2516800
H	0.0686650	-3.1780760	-1.2875070
H	1.6275620	-4.0576190	-1.2516700
H	1.4805820	-2.5430240	-2.1685910
H	3.4361660	-1.5613870	0.8863220
H	3.4361660	-1.5613910	-0.8863190
H	3.5508860	-3.0892340	0.0000040
H	0.9005210	3.6754710	-0.0000270
H	3.2593220	2.2408090	1.3309300
H	1.8848780	2.9673510	2.1673630
H	3.0261310	3.9967010	1.2720340
H	3.2593680	2.2407860	-1.3308780
H	3.0261740	3.9966780	-1.2720200
H	1.8849530	2.9673120	-2.1673710

QM_OMe_M_1.log

E = -814.599388
H = -814.172029
G = -814.242296
NImag=0

C	1.1714399	-0.8352013	-0.0734256
C	-0.0277192	-1.5308962	-0.2015247
C	-1.2754055	-0.8978606	-0.2799332
C	-1.2984755	0.5046928	-0.2036829
C	-0.1469237	1.2709269	-0.0645159
C	1.1584883	0.6197256	-0.0011399
O	2.2408369	1.2971081	0.1184569
C	-0.2090551	2.8091724	0.0253581
C	0.4065888	3.2785838	1.3666301
C	0.5924349	3.4320343	-1.1442307
C	-1.6478905	3.3554955	-0.0470661
C	2.5259412	-1.5683605	0.0011300
C	3.2289593	-1.2321372	1.3394249
C	2.3831016	-3.1000747	-0.0833026
C	3.4283615	-1.1126983	-1.1720411
C	-2.5430338	-1.6975974	-0.4619924
C	-3.2584858	-1.4349582	-1.7892200
O	-3.5502961	-1.4445001	0.5479502
C	-3.1200087	-1.7491701	1.8542442
H	-0.0154359	-2.6183178	-0.2513660
H	-2.2696505	0.9917394	-0.2398389
H	0.4203318	4.3765666	1.4265754
H	1.4252014	2.8959998	1.4477455
H	-0.1834597	2.9022463	2.2115023
H	1.6150080	3.0520266	-1.1214870
H	0.6064473	4.5289710	-1.0672183
H	0.1354443	3.1672038	-2.1057962
H	-1.6287622	4.4504240	0.0232982
H	-2.2710087	2.9807404	0.7730350
H	-2.1398501	3.0926007	-0.9906141
H	2.6402933	-1.6059191	2.1863733
H	3.3294275	-0.1493024	1.4285665
H	4.2236122	-1.6986373	1.3868250
H	1.9214959	-3.4178410	-1.0254995
H	1.7804557	-3.5022485	0.7394855
H	3.3746975	-3.5667592	-0.0265329
H	4.4206995	-1.5824053	-1.1082886
H	3.5358610	-0.0273145	-1.1400886
H	2.9809557	-1.3978227	-2.1323159
H	-2.2786456	-2.7704449	-0.4148251
H	-3.5251065	-0.3770168	-1.8725669
H	-4.1761655	-2.0304780	-1.8636444
H	-2.5994614	-1.6860733	-2.6254307
H	-3.9415571	-1.4954230	2.5329227
H	-2.2253015	-1.1800225	2.1375517
H	-2.8897468	-2.8234120	1.9720776

QM_OMe_M_2.log

E = -814.594966
H = -814.167376
G = -814.238066
NImag=0

C	-0.1286364	1.2869261	-0.0844097
C	-1.2844714	0.5403549	-0.2968378
C	-1.2802069	-0.8571565	-0.4311374

C	-0.0368053	-1.5057430	-0.3355919
C	1.1605794	-0.8350288	-0.1218832
C	1.1622080	0.6178420	0.0147057
O	2.2471477	1.2725869	0.2065991
C	2.5029881	-1.5884878	-0.0312510
C	3.4576139	-1.0895113	-1.1438368
C	3.1541231	-1.3261435	1.3492434
C	2.3455349	-3.1123158	-0.1944730
C	-0.1755062	2.8228762	0.0485486
C	0.6839850	3.4685932	-1.0662627
C	-1.6020785	3.3933250	-0.0697659
C	0.3869118	3.2446553	1.4284652
C	-2.5242569	-1.6861929	-0.6373675
C	-3.7060536	-0.9553522	-1.2857507
O	-2.9933083	-2.3833148	0.5618876
C	-3.1460176	-1.5779324	1.7089568
H	-2.2357064	1.0592169	-0.3694936
H	-0.0361663	-2.5889922	-0.4356342
H	3.0465791	-1.3217950	-2.1341007
H	4.4400936	-1.5767523	-1.0628631
H	3.5783514	-0.0087039	-1.0556109
H	4.1393056	-1.8101100	1.4140462
H	2.5258197	-1.7311033	2.1521303
H	3.2662809	-0.2506993	1.4953510
H	1.9199451	-3.3788148	-1.1689102
H	1.7042554	-3.5441982	0.5823744
H	3.3285756	-3.5943224	-0.1195991
H	0.2663258	3.2378494	-2.0541338
H	1.6996446	3.0738440	-1.0105134
H	0.7092111	4.5625262	-0.9567019
H	-2.2661859	3.0047379	0.7111498
H	-2.0554881	3.1670168	-1.0418205
H	-1.5705226	4.4851058	0.0351669
H	1.3965021	2.8466513	1.5416306
H	-0.2429808	2.8504688	2.2356140
H	0.4109289	4.3401299	1.5225886
H	-2.2659323	-2.5343970	-1.2848631
H	-4.0645596	-0.1236186	-0.6714425
H	-4.5395593	-1.6513227	-1.4274465
H	-3.4168148	-0.5450719	-2.2585421
H	-3.4226511	-2.2502654	2.5288852
H	-3.9454681	-0.8244467	1.6047994
H	-2.2199961	-1.0514079	1.9718932

QM_Ph_M_1.log

E = -931.131297

H = -930.653391

G = -930.729258

NImag=0

C	-0.8900335	1.3026822	0.1533217
C	0.3252223	0.7390319	0.5290639
C	0.5114117	-0.6448959	0.6714443
C	-0.5870807	-1.4750907	0.4124003
C	-1.8357033	-0.9886845	0.0305130
C	-2.0405097	0.4456749	-0.1126724
O	-3.1760351	0.9326411	-0.4595753
C	-3.0186938	-1.9372667	-0.2488030
C	-4.1861582	-1.6105111	0.7146272
C	-3.5011771	-1.7505233	-1.7084115
C	-2.6548602	-3.4225241	-0.0602495
C	-1.0564351	2.8285232	0.0039796
C	-1.4776452	3.1662222	-1.4473597
C	-2.1511286	3.3298040	0.9780301
C	0.2378325	3.6065401	0.3102991
C	1.8379546	-1.2317372	1.1407687
C	2.0854580	-0.9607786	2.6375621
C	3.0138297	-0.8316312	0.2427488
C	2.9280663	-1.0894433	-1.1367716
C	3.9747239	-0.7794804	-1.9994593
C	5.1419987	-0.1849491	-1.5076679
C	5.2400436	0.0889268	-0.1461149
C	4.1881415	-0.2351609	0.7183638
H	1.1794474	1.3857855	0.7158995
H	-0.4405969	-2.5480732	0.5239158
H	-4.4542408	-0.5583628	0.6062475
H	-3.8892054	-1.7969941	1.7541603
H	-5.0623184	-2.2379796	0.4953340
H	-2.7108085	-2.0378540	-2.4129346
H	-3.7546258	-0.7014336	-1.8691999
H	-4.3812158	-2.3774412	-1.9129634
H	-3.5325484	-4.0462813	-0.2719293
H	-2.3343831	-3.6397341	0.9654305
H	-1.8531066	-3.7385884	-0.7379703
H	-2.3898556	2.6198364	-1.6921158
H	-0.6883861	2.8766168	-2.1519389
H	-1.6537063	4.2457348	-1.5616994
H	-1.8455564	3.1607963	2.0181143

H	-3.0773394	2.7851649	0.7877969
H	-2.3250633	4.4077786	0.8478627
H	1.0545837	3.3276682	-0.3652421
H	0.5807331	3.4445394	1.3388797
H	0.0573744	4.6819036	0.1870194
H	1.7455031	-2.3234296	1.0342129
H	2.1711057	0.1127093	2.8382075
H	2.9924941	-1.4545786	3.0110646
H	1.2330936	-1.3310876	3.2136622
H	2.0104762	-1.5227478	-1.5249485
H	3.8787333	-0.9929826	-3.0613654
H	5.9580765	0.0655293	-2.1808102
H	6.1367204	0.5581562	0.2525842
H	4.2913789	-0.0140716	1.7756265

QM_Ph_M_2.log

E = -931.131609
H = -930.653842
G = -930.730462
NImag=0

C	-1.3739080	-1.2892810	0.1444200
C	-0.0777730	-1.2210240	0.6449640
C	0.5919660	-0.0127910	0.8915930
C	-0.1043660	1.1726190	0.6099830
C	-1.4032790	1.1894610	0.1036900
C	-2.1009480	-0.0621820	-0.1554330
O	-3.2980890	-0.0840260	-0.6170590
C	-2.1365840	2.5157200	-0.1830330
C	-2.5224410	2.5837180	-1.6815080
C	-3.4232410	2.5938050	0.6747970
C	-1.2825930	3.7579730	0.1373100
C	-2.0754710	-2.6414800	-0.0943910
C	-1.1908330	-3.8500840	0.2682200
C	-3.3592270	-2.7194940	0.7676410
C	-2.4605100	-2.7709810	-1.5888240
C	2.0207500	-0.0375730	1.4149530
C	2.3341960	1.1030920	2.4106530
C	3.1041070	-0.0652780	0.3274460
C	2.9277500	0.5568950	-0.9157610
C	3.9456530	0.5543790	-1.8706980
C	5.1618720	-0.0799170	-1.6091930
C	5.3490930	-0.7134240	-0.3794680
C	4.3299100	-0.7019890	0.5736170
H	0.4600970	-2.1413860	0.8644830
H	0.3933660	2.1185800	0.8036140
H	-3.1001560	3.4947310	-1.8951530
H	-3.1174160	1.7058660	-1.9381990
H	-1.6224760	2.6004530	-2.3088730
H	-4.0425340	1.7192550	0.4696570
H	-3.9907120	3.5079870	0.4475400
H	-3.1709940	2.6094620	1.7423300
H	-1.8544110	4.6659860	-0.0920630
H	-0.3618750	3.7887760	-0.4571480
H	-1.0021690	3.8029440	1.1961900
H	-0.2710180	-3.8795140	-0.3272820
H	-1.7411850	-4.7793560	0.0737100
H	-0.9074870	-3.8494050	1.3273040
H	-3.1061280	-2.6939690	1.8348210
H	-3.9057900	-3.6534330	0.5717090
H	-3.9987670	-1.8667580	0.5344920
H	-3.0149540	-3.7033920	-1.7692020
H	-1.5609660	-2.7863440	-2.2164860
H	-3.0782100	-1.9181880	-1.8744830
H	2.1390720	-0.9765490	1.9753270
H	2.3020840	2.0804030	1.9188830
H	3.3363650	0.9874180	2.8400270
H	1.6013410	1.1105790	3.2237800
H	1.9727680	1.0256560	-1.1320220
H	3.7829570	1.0422070	-2.8288820
H	5.9503180	-0.0888170	-2.3577330
H	6.2862310	-1.2222680	-0.1645280
H	4.4826270	-1.2016950	1.5287870

--

End of file.
